# Mechanistic Investigation of the Rhodium-Catalyzed Transfer Hydroarylation Reaction Involving Reversible C–C Bond Activation

**DOI:** 10.1021/jacs.3c07780

**Published:** 2023-11-30

**Authors:** Marius D. R. Lutz, Sven Roediger, Miguel A. Rivero-Crespo, Bill Morandi

**Affiliations:** ETH Zürich, Vladimir-Prelog-Weg 3, HCI, 8093 Zürich, Switzerland

## Abstract

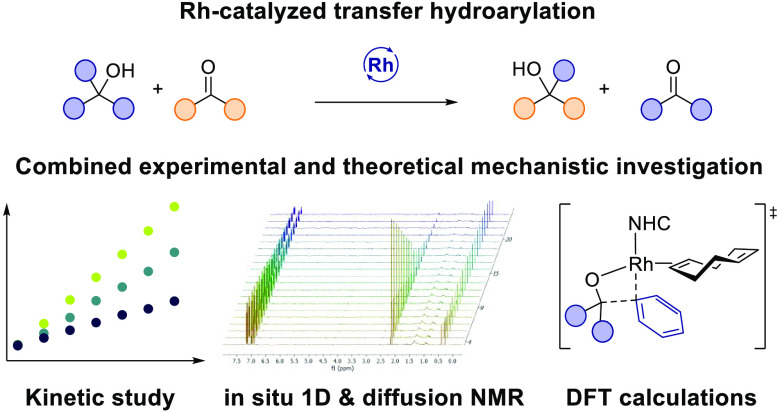

Carbon–carbon (C–C) bonds are ubiquitous but are among the least reactive bonds in organic chemistry. Recently, catalytic approaches to activate C–C bonds by transition metals have demonstrated the synthetic potential of directly reorganizing the skeleton of small molecules. However, these approaches are usually restricted to strained molecules or rely on directing groups, limiting their broader impact. We report a detailed mechanistic study of a rare example of catalytic C–C bond cleavage of unstrained alcohols that enables reversible ketone transfer hydroarylation under Rh-catalysis. Combined insight from kinetic analysis, in situ nuclear magnetic resonance (NMR) monitoring, and density functional theory (DFT) calculations supports a symmetric catalytic cycle, including a key reversible β-carbon elimination event. In addition, we provide evidence regarding the turnover-limiting step, the catalyst resting state, and the role of the sterically encumbered NHC ligand. The study further led to an improved catalytic system with the discovery of two air-stable precatalysts that showed higher activity for the transformation in comparison to the original conditions.

## Introduction

Carbon–carbon (C–C) bonds form the backbone of organic molecules. While forging C–C bonds in an efficient manner has been a longstanding goal of organic synthesis en route to ever more structurally complex functional molecules, their cleavage has only recently become an important target for synthetic chemistry. Despite the challenges associated with cleaving such strong bonds, recent developments in catalytic activation of C–C single bonds have tremendously impacted the field of skeletal editing approaches,^1^ small molecule functionalization,^2^ polymer synthesis,^3^ and target-oriented synthesis.^4^ Alcohols have served as remarkably versatile handles for skeletal reorganization through catalytic β-carbon elimination, one of the most applied mechanistic manifolds for catalytic C–C bond cleavage.^2b,2d,5^ Among the different possible substrates amenable to β-carbon elimination, the activation of unstrained alcohols without an additional directing group is particularly attractive as it allows for a more general application of β-carbon elimination across diverse substrates.^6^

**Scheme 1:**
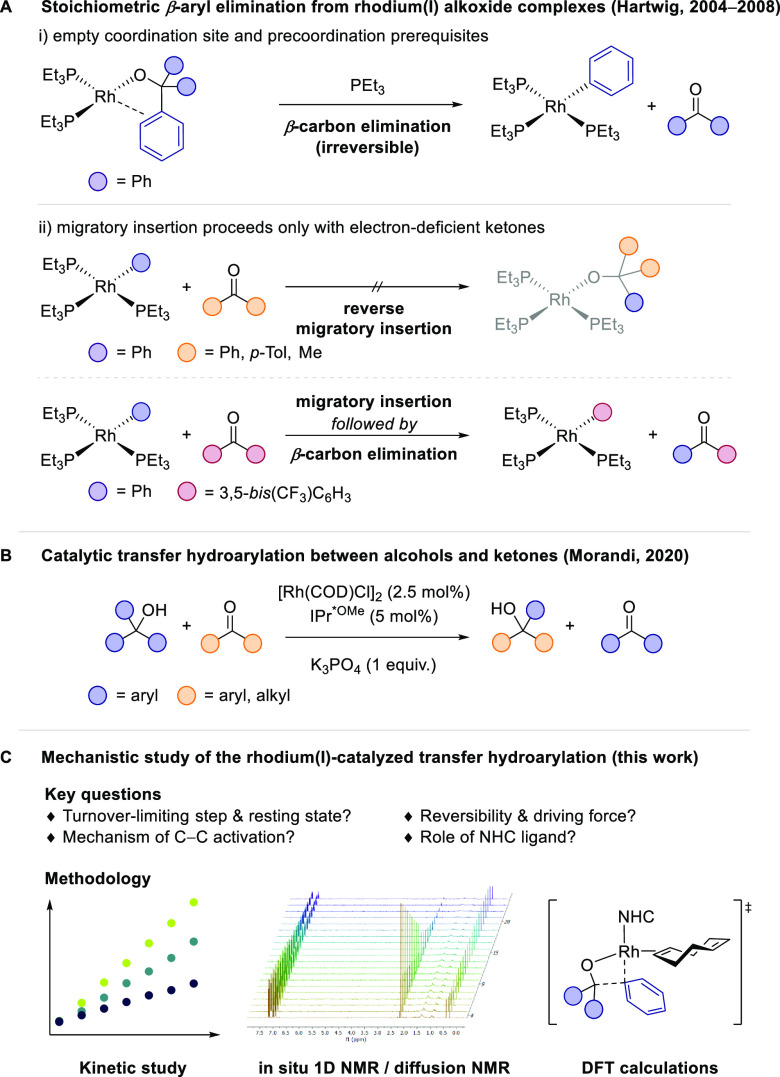
Context of This Work

While catalytic β-carbon elimination has been explored in many instances with late transition metals, the mechanistic details of such reactions are not well understood. However, some insightful stoichiometric organometallic studies, primarily focused on alkyl metal species, have provided key information regarding the β-carbon elimination step.^7,8^ For example, in a seminal study, Hartwig and co-workers investigated the stoichiometric β-carbon elimination step from isolated rhodium alkoxide complexes and the related migratory insertion of ketones into rhodium(I) aryl complexes (Scheme 1A).^8,9^ In contrast, in-depth mechanistic studies of catalytic reactions involving a key β-carbon elimination step remain particularly rare.^10^ Given the potential to improve organic reactions in terms of efficiency and scope by mechanistically driven design,^11^ detailed mechanistic studies of catalytic reactions involving β-carbon elimination are in high demand to expand their scope and synthetic utility.

As part of our continuing interest in developing shuttle catalysis reactions,^12,13^ we recently developed a rhodium-catalyzed transfer hydrohydroarylation reaction between unstrained tertiary alcohols and unactivated ketones that extends the concept of transfer hydrogenation to hydrocarbyl groups in tertiary alcohols and, at the same time, leverages alcohols as latent aryl nucleophiles (Scheme 1B).^14^ In our previous report we studied the reversibility of the overall reaction and conducted a free-energy relationship analysis, but information regarding the catalytic intermediates, the identity of the resting state, and how the C–C bond cleavage steps proceed was unclear.

Herein, we discuss in-depth experimental (intermediate synthesis, kinetic analysis, in situ NMR, diffusion NMR) and theoretical (DFT) mechanistic studies on this reaction involving the catalytic reversible cleavage of an unactivated C–C bond (Scheme 1C). Systematic mechanistic studies indicated that the symmetric catalytic cycle proceeds through four major steps: dissociative alcohol exchange, turnover-limiting β-carbon elimination, dissociative ketone exchange, and migratory insertion. Further, identifying an off-cycle resting state allowed us to develop improved conditions with air-stable precatalysts for the catalytic reaction.

## Results and Discussion

### Mechanistic Hypothesis

**Scheme 2:**
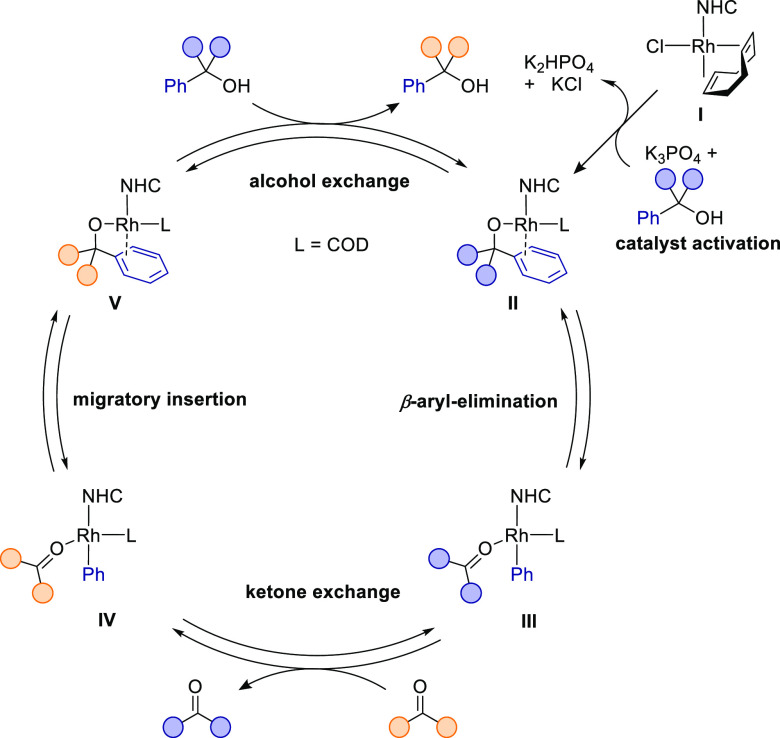
Originally Proposed Catalytic Cycle

In our initial report, we proposed a catalytic cycle based on literature precedent and preliminary mechanistic experiments (Scheme 2).^14^ Because of the isofunctional nature of the reaction, the catalytic cycle is symmetric and all elementary steps are reversible. The precatalyst [Rh(IPr*^OMe^)(COD)Cl] (**I**) is believed to undergo base-assisted ligand exchange with the alcohol substrate to form catalytically active alkoxide complex **II**. Partial or full decoordination of the COD ligand would create an open coordination site for β-carbon elimination. Coordination of the phenyl group is expected to facilitate the cleavage of the strong C(sp^2^)–C(sp^3^) bond.^8^ This is followed by ketone exchange from species **III**, releasing the ketone product and generating complex **IV**. Migratory insertion of the ketone into the rhodium–phenyl bond then is predicted to give rise to alkoxide complex **V**, which would release the second product and close the cycle by alcohol exchange.

### Synthesis of Organometallic Complexes

**Scheme 3:**
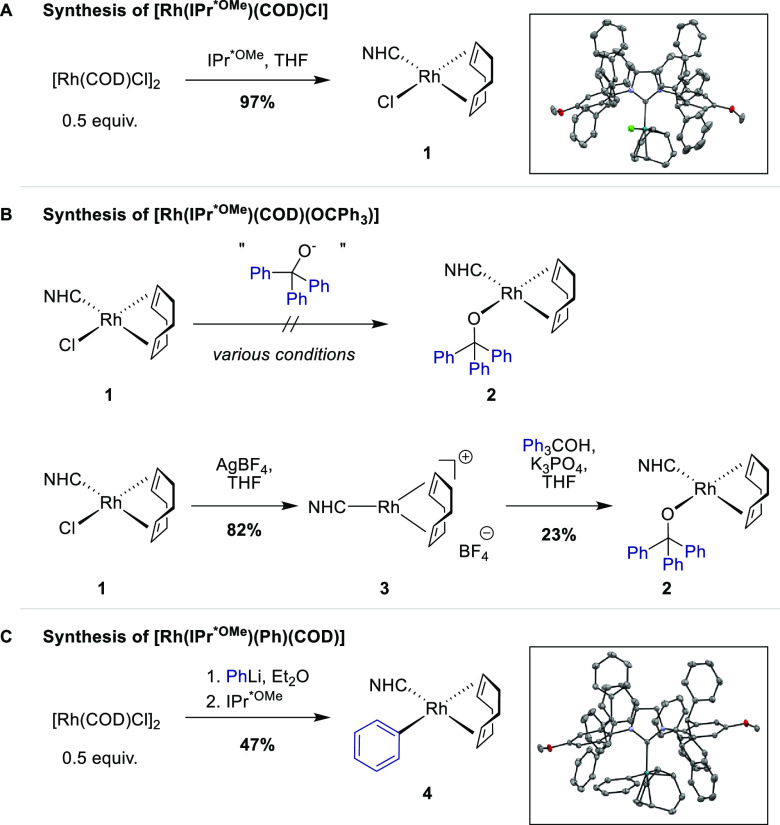
Synthesis of Organometallic Complexes

We commenced our investigation by synthesizing several rhodium complexes to identify potential on-cycle intermediates. The catalytically active complex [Rh(IPr*^OMe^)(COD)Cl] (**1**) was synthesized as described in our initial report from [Rh(COD)Cl]_2_ and IPr*^OMe^ in 97% yield (Scheme 3A).^14^

We next turned our attention to the synthesis of alkoxide complex **2**, which was envisioned to be formed by transmetalation from **1** (Scheme 3B). The attempted conditions and outcomes to synthesize **2** are summarized in Table S1 in the Supporting Information (SI). The direct salt exchange between **1** and alkoxide salts of triphenylmethanol did not result in any conversion. This is consistent with calculations showing that the ligand exchange of **1** with triphenylmethanolate is endergonic, thus preventing a productive transmetalation in a stoichiometric experiment (+10.8 kcal/mol; see SI, section 8). Therefore, we changed our synthetic approach and sought to abstract the chloride ion and replace it with a non-coordinating anion to ease the coordination of the alcohol. We prepared the cationic complex [Rh(IPr*^OMe^)(COD)]BF_4_ (**3**) by halide abstraction from **1**, as previously described.^15^ Mimicking the catalytic reaction conditions, complex **3** was reacted with triphenylmethanol and K_3_PO_4_, which afforded the desired alkoxide complex **2** in 23% yield (Scheme 3B).

We then targeted the putative aryl intermediate [Rh(IPr*^OMe^)(Ph)(COD)] (**4**), which might be generated after β-carbon elimination. Transmetalation from [Rh(COD)Cl]_2_, followed by reaction with the NHC ligand, afforded the novel phenyl complex **4** in 49% yield (Scheme 3C). Lastly, we attempted to synthesize complexes with bound ketone substrates to study the insertion of the ketone into the Rh–aryl bond. However, two different routes from cationic complex **3** and phenyl complex **4** failed to afford the ketone complexes (see SI, section 3).

### Kinetic Analysis of the Catalytic Reaction

**Figure 1:**
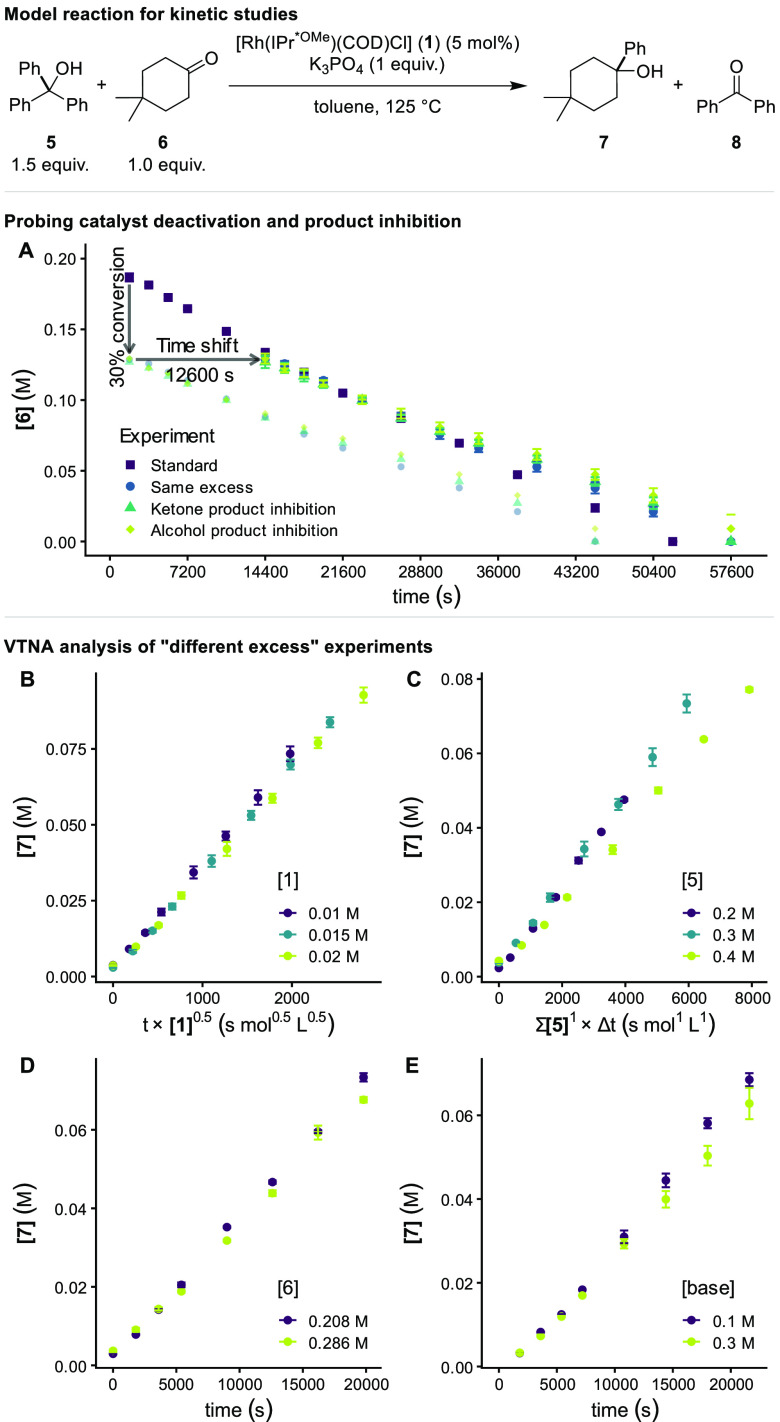
Kinetic studies. Concentrations determined vs *n*-dodecane as internal standard using GC-FID. Each data point is the average of three experiments with standard errors of the mean, including error bars. (A) “Same excess” experiments to test for catalyst deactivation and product inhibition. The three concentration profiles of the “same excess” and “product inhibition” experiments were shifted by 12600 s (210 min) to take into account the time required to complete six turnovers (30% conversion). (B–E) Visual kinetic analysis of “different excess” experiments is performed using VTNA plots. The time scale was normalized according to the VTNA method. For the equivalent plots for product **8** see SI, section 6. (B) Order in catalyst **1**. (C) Order in alcohol substrate **5**. (D) Order in ketone substrate **6**. (E) Order in base.

With a range of catalyst precursors and potential catalytic intermediates in hand, the kinetics of the transfer hydroarylation reaction were studied to identify the resting state and the turnover-limiting step in the system. Additionally, the kinetic analysis was anticipated to reveal potential deactivation or product inhibition pathways, allowing for improved reaction conditions to be developed. For the kinetic and NMR monitoring studies, we selected the reaction of triphenylmethanol (**5**) and 4,4-dimethylcyclohexanone (**6**) we previously used for the reaction development,^14^ because of their clean and high conversion toward alcohol product **7** and ketone product **8** with no detected side reactions, as well as their easy identification by NMR and GC (Figure 1). Temporal concentrations were determined by GC-FID analysis of a series of samples taken at regular intervals from the reaction mixtures using *n-*dodecane as an internal standard. To ensure that in situ formation of the rhodium NHC complex does not introduce a source of variability that could potentially affect the kinetic analysis, isolated complex **1** was used throughout our kinetic studies.^16^

Initially, we assessed the catalytic and kinetic competence of the synthesized complexes. Complexes **3** and **4** were found to be catalytically and kinetically competent and displayed a higher initial rate compared to **1**. This suggests that these complexes are either on-cycle themselves or can immediately form such intermediates under the reaction conditions. The rate of formation for ketone product **8** was slightly higher than that of alcohol product **7**, independently of the employed precatalyst. This result is consistent with the generally higher measured yields of **8** and the formation of benzene as a side product in the catalytic reaction. This observation supports the hypothesis that the catalyst can turn over through an undesired pathway after β-carbon elimination. Instead of undergoing ketone insertion, protodemetalation can occur by proton transfer from an alcohol (substrate or product), releasing benzene and forming the putative alkoxide complex, thus prematurely regenerating the catalyst (SI, Scheme S1). The comparably more challenging migratory insertion into unactivated carbonyls compared to β-carbon elimination has also been noted in stoichiometric studies (vide supra).^8^

We continued our studies by interrogating the system by reaction progress kinetic analysis (RPKA).^17,18^ Before the rate law of the reaction was determined, “same excess” experiments were carried out to probe catalyst deactivation and product inhibition (Figure 1A). A good overlap between the standard and the “same excess” concentration profiles was found during the first 6 h of the reaction, which corresponded to roughly 70% conversion. Also, both product inhibition experiments overlapped with the “same excess” experiment over the whole course of the reaction profile, supporting that product inhibition is not occurring, for neither the ketone nor alcohol products.

We next employed Variable Time Normalization Analysis (VTNA) to study the rate dependence on catalyst and reactant concentration.^19,20^ In a series of “different excess” experiments, the concentrations of products **7** and **8** were followed over the course of the reaction and plotted against a normalized time scale, including the order in the reagent in question, allowing for visual identification of the order in the measured species. The order in catalyst **1** was determined to be approximately 0.5 by graphical analysis (Figure 1B) of the concentration profiles of the alcohol product **7**. A first-order dependence of the rate on the concentration of alcohol substrate **5** (Figure 1C), and a zero-order dependence on both ketone **6** (Figure 1D) and inorganic base (Figure 1E) were observed for both products (see SI, section 6 for analogous analysis for product **8**). Plotting product formation against the VTNA normalized time scale with the concentrations of all reagents raised to their correct order linearized the reaction profiles and allowed determination of the observed rate constant (for **7**: *k*_obs_ = (1.21 ± 0.02) × 10^–4^ M s^–1.5^; for **8**: *k*_obs_ = (1.59 ± 0.06) × 10^–4^ M s^–1.5^) as the slope of the fitted line (see SI, section 6 for details).^21^

**Scheme 4:**
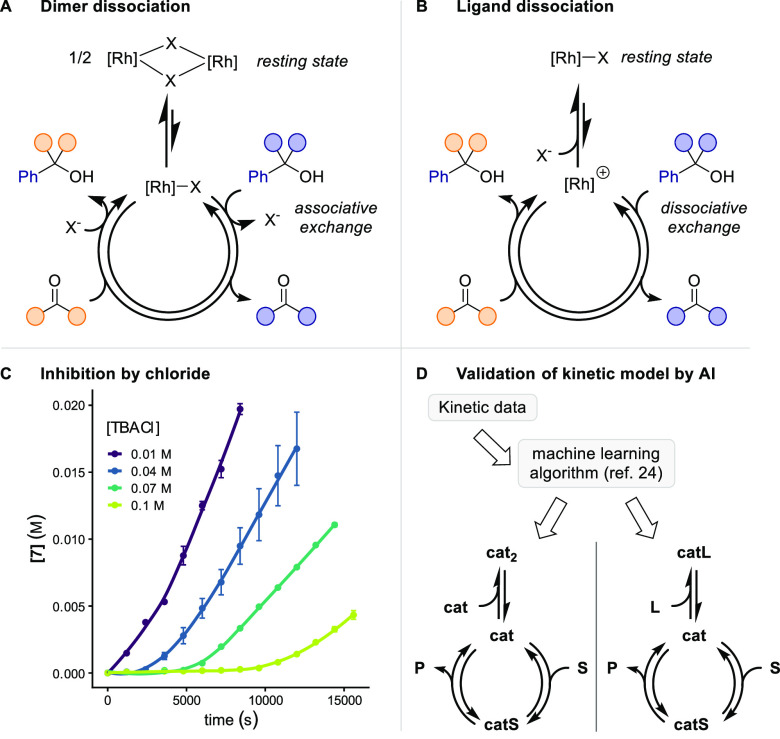
Two Proposed Mechanisms Involving a Reversible Activation Step from an Off-Cycle Resting State

The rate law obtained by the VTNA approach was further confirmed using the method of initial rates, which afforded comparable results. The order in complex **1** was found to be 0.52 ± 0.05 for monitoring of **7** and 0.48 ± 0.05 for monitoring of **8**, and the rate-dependence in alcohol **5** was close to first-order (0.78 ± 0.11 for monitoring of **7** and 1.11 ± 0.01 for monitoring of **8**). Ketone **6** exhibited a small fractional order for the alcohol product (0.19 ± 0.06) but did not affect the ketone product formation rate (0.01 ± 0.05). The former may be indicative of reversibility of the ketone insertion step,^22^ or the ketone insertion step lying after the turnover-limiting step. Different initial concentrations of the inorganic base showed a negligible influence on the reaction rate (0.07 ± 0.03 for monitoring of **7** and 0.09 ± 0.07 for monitoring of **8**). This observed rate law (eqs 1–2) is consistent with the VTNA analysis and with a mechanism in which alcohol **5** is involved at or before the turnover-limiting step. This is in line with the dependence of the reaction rate on the substitution of the alcohol substrate, as revealed by a Hammett study we conducted previously.^14^
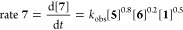
1
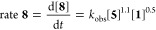
2The half order in **1** points toward an equilibrium with an off-cycle resting state. Two scenarios could result in a half-order in catalyst (Scheme 4, top): (A) The active (monomeric) catalyst is in equilibrium with an inactive dimer; (B) The active catalyst arises from reversible dissociation of a ligand from an off-cycle resting state.^11b,23^ Both scenarios are plausible with our mechanistic hypothesis that involves dissociation from a likely stabilized intermediate to free up a coordination site for the key β-carbon elimination step. We conducted additional kinetic experiments under saturation conditions, indicating that COD does not significantly inhibit the reaction (see SI, section 6 for details). Likewise, we measured the influence of added exogenous chloride, which inhibited the reaction (Scheme 4C). We could not precisely determine the order, because of the limited solubility of tetrabutylammonium chloride (TBACl) as the chloride source in the reaction medium. However, the inverse order in chloride indicates a kinetically relevant transmetalation step between the chloride ligand and the alcohol substrate. This result suggested that the dissociation of a chloride ligand might give rise to the fractional catalyst order.^23b^

Notably, our conclusions from the kinetic data were validated by the machine learning algorithm recently introduced by Burés and Larrosa that assigns one or multiple probable reaction mechanism classes based on experimental time–concentration data with high confidence.^24^ The algorithm predicts one of the two scenarios depicted in Scheme 4D from our kinetic data. Still, more data, e.g., the identity of the resting state, are required to validate this hypothesis. We thus turned to NMR spectroscopy to elucidate the identity of the resting state.

### NMR Analysis of the Reaction

**Figure 2:**
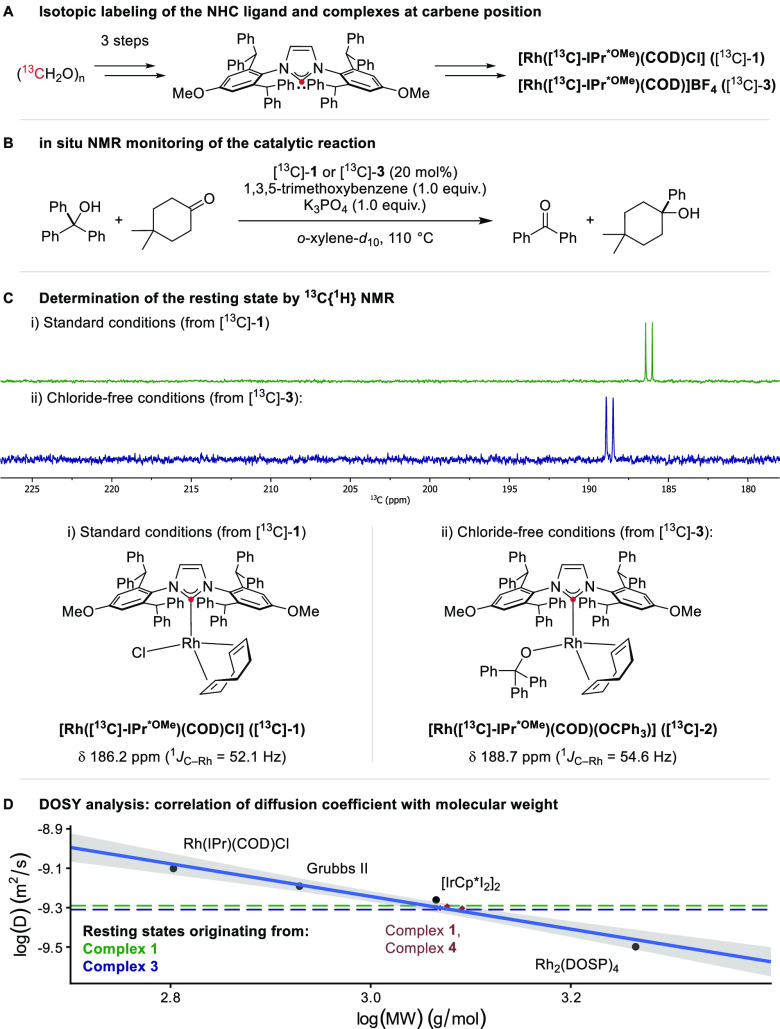
Elucidation of the resting states was performed by NMR spectroscopy. (A) Isotopic labeling of the NHC ligand for resting state analysis by ^13^C NMR. (B) Reaction conditions of the model reaction. (C) ^13^C{^1^H} NMR spectra of the catalytic reaction with [^13^C]-**1** and [^13^C]-**3** showing the signals of the detected catalyst resting states **RS-1** and **RS-3**. (D) Elucidation of resting state mass by correlation of diffusion coefficient (DOSY experiments) to molecular weight. The blue line represents the calibration curve using known organometallic complexes (black and brown points). The horizontal dashed lines represent the measured diffusion coefficients of observed resting states **RS-1** and **RS-3**.

We continued our mechanistic investigation of the transfer hydroarylation by monitoring the reaction by NMR spectroscopy to exclude one of the two kinetically plausible resting states. In contrast to systems with phosphine ligands where the catalyst speciation can be readily monitored by ^31^P NMR thanks to its broad chemical shift range and high sensitivity,^25^ changes in the chemical environment of the coordinating carbon atom in NHC ligands are more challenging to monitor due to the lower abundance of ^13^C isotopes. To circumvent this apparent shortcoming without changing the ligand, we turned to isotopic enrichment of the ligand as a seldomly employed yet highly diagnostic mechanistic tool in homogeneous catalysis,^26^ and synthesized ^13^C-labeled analogs at the carbene position of IPr^*OMe^ and complexes **1** and **3** (Figure 2A). Because the carbene signal splits into a doublet when coordinated to rhodium (I(^103^Rh) = 1/2), the speciation of the catalyst, as well as the discrimination between bound and free ligand, is possible by measuring the ^13^C{^1^H}-NMR spectrum, akin to what is possible with a phosphine ligand.

The monitoring studies were performed under the same conditions as those described for the catalytic experiments. The standard reaction catalyzed by 20 mol % [^13^C]-**1** was monitored by ^1^H- and ^13^C{^1^H}-NMR in situ at 110 °C in *o*-xylene-*d*_10_ (Figure 2B; see SI, section 7 for details and spectra). Over the course of the measurement, the only catalyst species in the ^13^C{^1^H}-NMR spectrum was detected at 186.2 ppm (^1^*J*_C–Rh_ = 52.1 Hz) (**RS-1**), matching the shift of isolated **1** under identical conditions (Figure 2C, green spectrum). Because the reaction temperature might have been insufficient, the reaction was repeated ex situ in toluene-*d*_8_ at 125 °C outside the spectrometer for technical reasons. Again, no evolution in speciation of the Rh-containing species was observed. We, therefore, conclude that **1** is the resting state under the standard reaction conditions.

By contrast, a different resting state was formed when the cationic complex [^13^C]-**3** was employed as the precatalyst. In the ^13^C{^1^H}-NMR spectrum, a single species with a doublet at 188.7 ppm (^1^*J*_C–Rh_ = 54.6 Hz) (**RS-3**) was observed at 110 °C (Figure 2C, blue spectrum). When the reaction was then carried out ex situ in toluene-*d*_8_ at 125 °C, the same species was observed at δ 188.6 ppm (d,^1^*J*_C–Rh_ = 54.3 Hz), confirming that the same species exists at ambient temperature. The major species generated from **3** matches alkoxide complex **2**, pointing toward the alkoxide complex being the resting state under chloride-free conditions. When **RS-3** was treated with tetrabutylammonium chloride (TBACl), it converted into **RS-1** (see SI, section 7), further validating that complex **1** is the resting state under the standard conditions.

### DOSY Analysis of the Resting States

Diffusion-Ordered NMR SpectroscopY (DOSY) has increasingly been used to study the speciation of organometallic complexes in solution and in mixtures.^27,28^ Although the 1D NMR spectra suggested monomeric resting states, the possibility of generating a dimeric species under catalytic conditions with similar chemical shift values could not be completely ruled out. We wondered whether we could distinguish between a monomeric and a dimeric resting state by employing ^1^H-DOSY-NMR. A calibration curve using well-defined organometallic complexes, including [Au(PPh_3_)Cl], Grubbs’ second generation catalyst, [Ir(Cp*)I_2_]_2_, [Rh(IPr)(COD)Cl], [Rh_2_(DOSP)_4_], as well as **1** and **4** was established, showing a linear correlation between diffusion coefficient and molecular weight (MW) (Figure 2D). The reaction mixtures from the NMR monitoring experiments were subjected to a DOSY analysis, and the diffusion coefficients of the resting states were determined and plotted as horizontal lines. Both resting states (**RS-1:** 5.128 × 10^–10^ m^2^/s, **RS-3**: 4.896 × 10^–10^ m^2^/s) lie in the range of the characterized monomeric complexes **1**–**4**, thus excluding dimeric resting states.

### DFT Calculations

**Figure 3:**
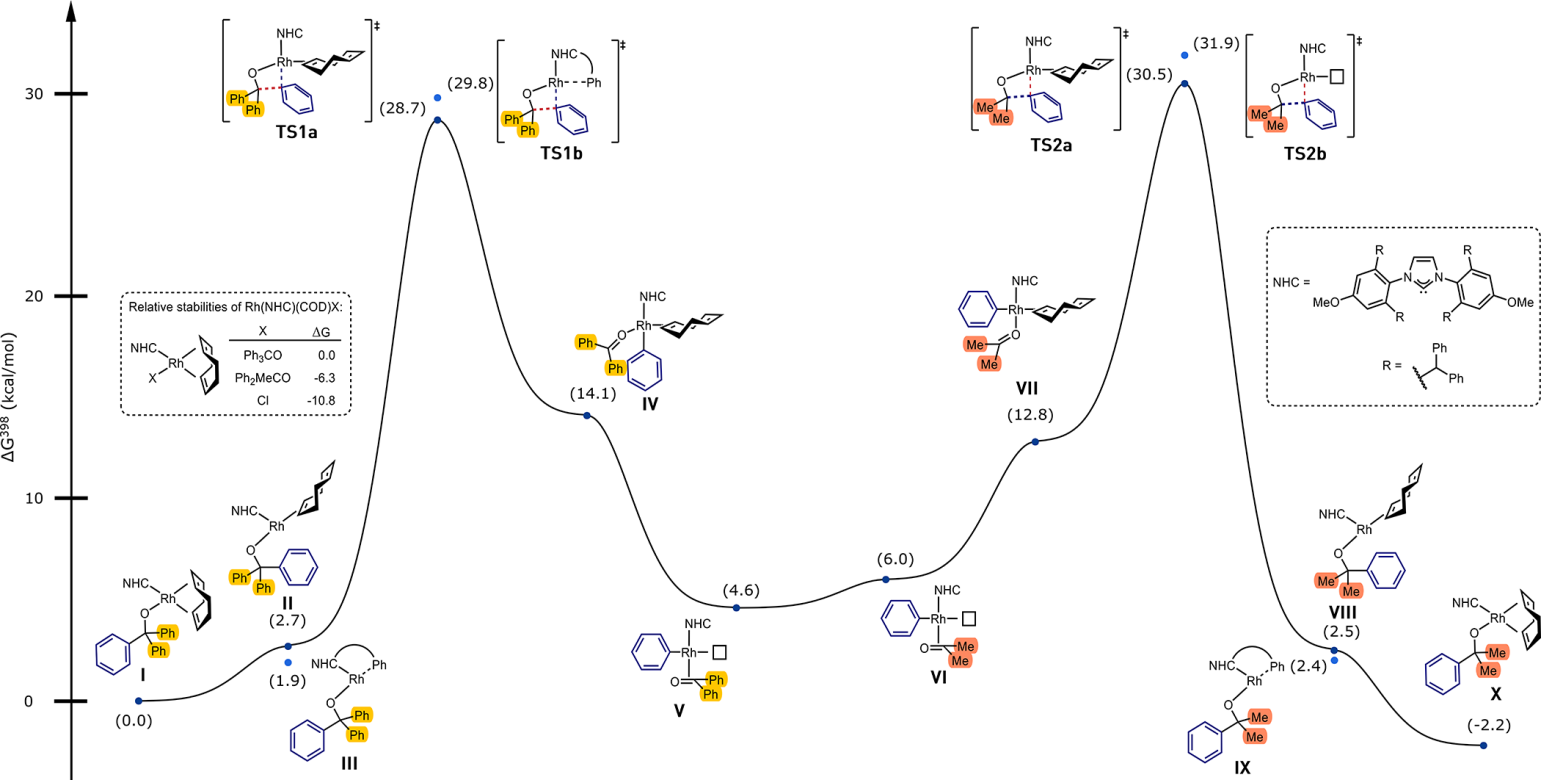
Free energy profile for the rhodium-catalyzed transfer hydroarylation between alcohol **5** and acetone at the PBE0-D3(BJ)/SMD(toluene)-def2tzvp//PBE0-D3(BJ)/def2svp-def2tzvp(Rh) level of theory. NHC = IPr^*OMe^. Energy diagram created with EveRplot.^29^

To complement our experimental results and gain further information on the kinetically invisible steps of the reaction, we performed computational studies on the transformation of alcohol **5** and acetone as a model reaction (Figure 3).

We used alkoxide complex **2** (labeled as complex **I** in Figure 3) as a starting point for our investigation. Partial or full decoordination of COD from **I** poses a low energy penalty of 2.7 or 1.9 kcal/mol, respectively (complexes **II**–**III**). The key β-carbon elimination transition state was examined for the COD-containing and COD-free structures. A slight preference for the transition state containing monodentate COD (**TS1a**, 28.7 kcal/mol) over the one containing no COD (**TS1b**, 29.8 kcal/mol) was found. Associative and dissociative ketone exchanges of the resulting phenyl complex were examined, revealing a preference for the latter (see SI, section 8 for details). The insertion of acetone into the phenyl–metal bond (**TS2a**, 30.5 kcal/mol) is, as expected, similar in energy to the β-carbon elimination transition state **TS1a**. Like **TS1a**, the favored transition state for the β-carbon elimination step, **TS2a** also contains a monodentate COD ligand. **TS2b**, the corresponding COD-free transition state, is 1.4 kcal/mol higher in energy than **TS2a**. The product alkoxide complex **X** is 2.2 kcal/mol lower in energy compared to the starting complex **I**, making the reaction slightly exergonic. As all energy barriers can be passed in both directions, the reaction is reversible, with a thermodynamic preference for the product side. This conforms with the experimental results in our previous report.^14^

While the flexibility of the NHC ligand family IPr* has been observed several times in the literature,^30,31^ it has not been studied in detail in a catalytic context. We noticed that the NHC in the COD-free alkoxide complexes (structures **III** and **IX**) not only forms dispersive interactions with the alkoxide fragment but also is directly coordinated to the rhodium with a peripheral phenyl group of the ligand in η^2^-fashion. The flexibility of the ligand is also reflected in changes of the buried volume. Whereas the NHC ligand has a very compact conformation in the sterically encumbered COD-containing complex **I** (%V_bur_ = 33.5%), it wraps around the metal in the low-coordinate COD-free complex **III**, resulting in a %V_bur_ value of 63.7% (see SI, section 8 for details). By this means, the large NHC ligand can not only enforce steric bulk but also stabilize low-coordinate intermediates. Notably, we could also experimentally detect η^6^-coordinated complexes with IPr^*OMe^ in cationic Rh(I) complexes.^15^

### Proposed Catalytic Cycle

**Scheme 5:**
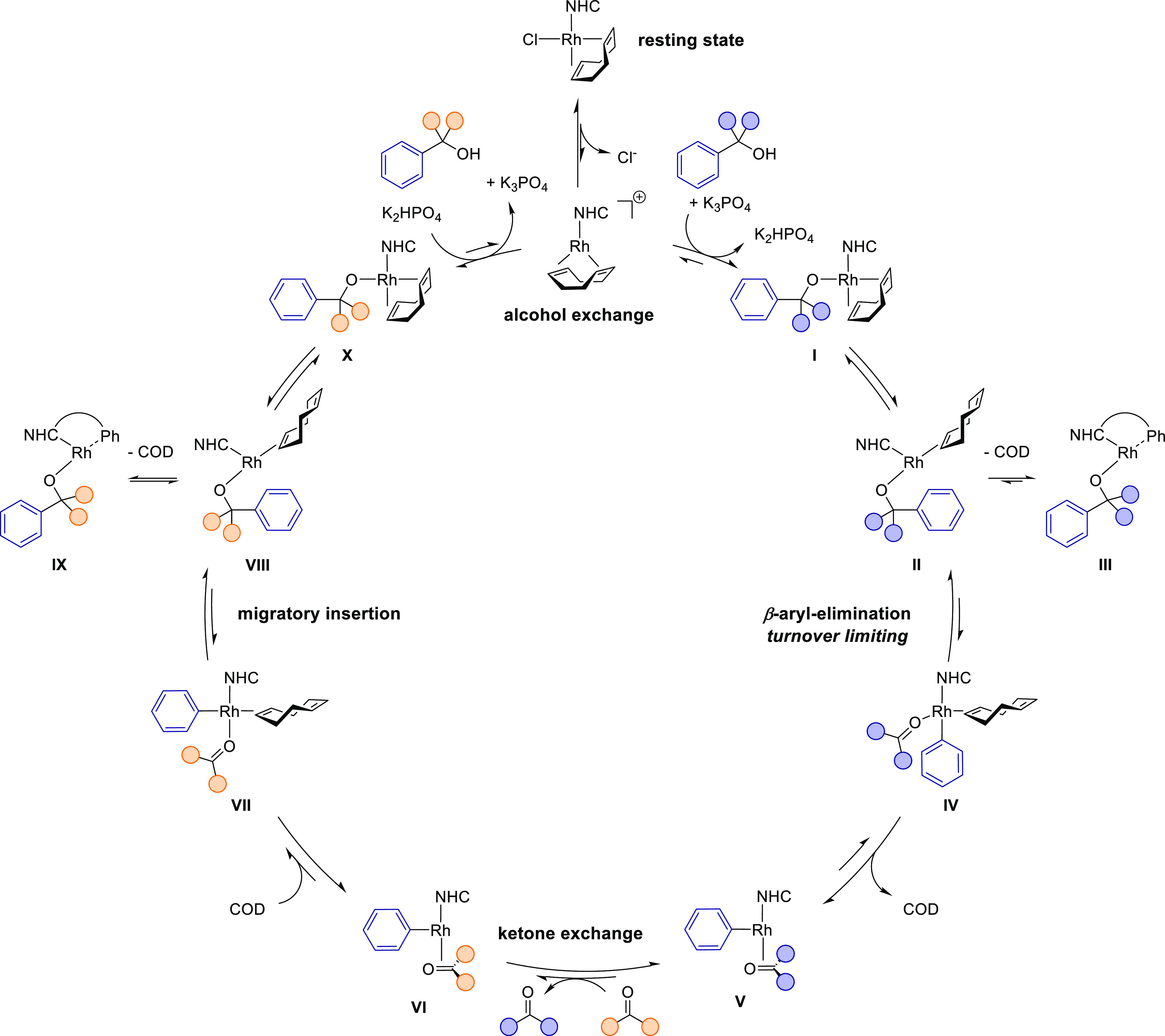
Proposed Mechanism of the Rhodium-Catalyzed Transfer Hydroarylation

Based on the combined results from the stoichiometric experiments, kinetic analysis, in situ NMR studies, and theoretical calculations, we propose a revised catalytic cycle for the transfer hydroarylation reaction (Scheme 5). Under the standard reaction conditions employing complex **1**, it is an off-cycle resting state. Reversible transmetalation with the alcohol substrate via a dissociative mechanism is the entryway into the catalytic cycle. Coordination and deprotonation of the alcohol, assisted by the base, lead to alkoxide complex **I** that serves as the resting state when employing cationic complex **3**. Partial decoordination of COD opens up a coordination site for β-carbon elimination (**II**). Full decoordination is also feasible (**III**) but results in a higher barrier for C–C bond cleavage. β-Aryl elimination takes place from **II**, which affords ketone–aryl complex **IV**. After β-carbon elimination, decoordination of the COD ligand and subsequent ketone exchange lead to intermediate **VI**. This intermediate can reassociate COD (**VII**), stabilizing a more favorable transition state for migratory insertion of the ketone substrate into the Rh–aryl bond to afford intermediate **VIII**. Isomerization affords complex **X**, which can transmetalate with another alcohol to close the cycle. In summary, our mechanistic experiments and the DFT calculations support that the catalytic cycle is symmetric and fully reversible at the reaction temperature.

The mechanistic proposal was further supported by kinetic modeling (see SI, section 6.8 for the derivation of the theoretical rate law). The experimental concentration profiles could be satisfactorily simulated with our model, which accounts for the observed half-order dependence at the evaluated reaction conditions. Although the model is a simplification of the real reaction system, the experimental data fit within the prediction interval of the theoretical model (*R*^2^ = 0.96).

### Improved Protocols for the Transfer Hydrorylation

**Scheme 6:**
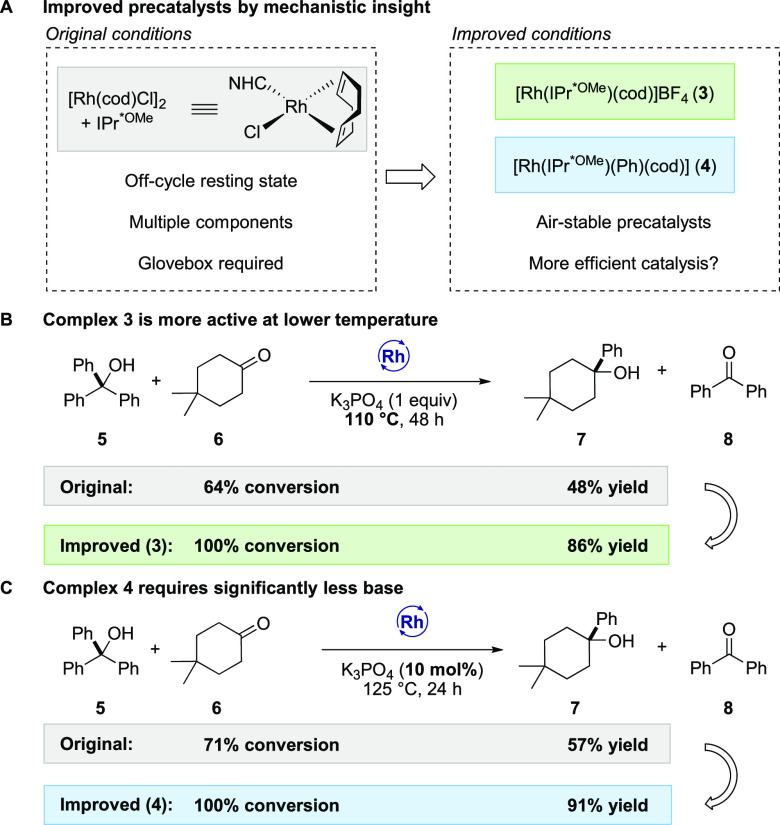
Improved Conditions for the Transfer Hydroarylation Reaction

After elucidating several aspects of the mechanism of the reaction, including concentration–rate dependences and resting state analysis, we reasoned that cationic complex **3** and phenyl complex **4** should be more active in the catalytic reaction, since they do not introduce inhibitory X-type ligands and showed a higher rate of product formation. Therefore, we benchmarked our previously reported conditions with the new conditions based on our analysis (Scheme 6A).

Direct comparison of chloride complex **1** with cationic complex **3** and phenyl complex **4** under the standard conditions showed that all complexes performed comparably, affording alcohol product **7** in 75–94% yield (see SI, section 9 for details). While complex **4** displayed the highest initial rate, complex **3** delivered the highest yield of the alcohol product after 24 h. Complex **3** exhibits high activity at a lower temperature, delivering **7** in 86% yield at 110 °C within 48 h (Scheme 6B). Reasoning that the chloride-free complexes might not require a stoichiometric base, we re-evaluated the required amount of K_3_PO_4_. When the base was omitted, no conversion was detected in the case of **1**. However, the reaction occurred in the case of complexes **3** and **4**, albeit with a lower efficiency. Adding a catalytic amount of base (10 mol %) to the reaction catalyzed by **3** and **4** increased the yield to 52% and 91%, respectively (Scheme 6C). We reason that the base plays a dual role: it not only facilitates transmetalation of the chloride ligand in the reaction using **1** but also might assist in deprotonation of the bound alcohol substrates.

Next, we were interested in assessing the air stability of the newly found precatalysts for transfer hydroarylation. The applicability of catalytic protocols involving organometallic complexes, especially those employed in β-carbon elimination catalysis, can often be hampered by the necessity of a glovebox to set up reactions under inert conditions.^32,33^ Complexes **3** and **4** were stored under air at room temperature for seven months to assess their long-term stability. Gratifyingly, both precatalysts performed the reaction without any erosion of their activity with respect to the inert conditions (see SI, section 9 for details). These complexes can thus be used as bench-stable catalysts for ketone arylation, which traditionally requires air-sensitive and pyrophoric reagents, i.e., organolithium and organomagnesium compounds.

## Conclusion

In this work, we investigated the mechanism of the rhodium-catalyzed transfer arylation using a combination of experimental and computational methods. The reaction proceeds via a symmetric and reversible redox-neutral catalytic cycle that is driven by the thermodynamic stability of the products. According to the kinetic studies, β-carbon elimination is the turnover-limiting step. Spectroscopic studies revealed the resting state to be an off-cycle intermediate with a chloride ligand. We further identified two air-stable precatalysts that translate to milder reaction conditions by circumventing the off-cycle resting state. In addition, we elucidated the role of the privileged sterically encumbered NHC ligand IPr^*OMe^. We believe that the conclusions drawn from this study will inspire future reaction development of reversible reactions in the context of C–C bond activation.

## Supporting Information Available

Cartesian Coordinates of the optimized structures (XYZ)
Full details of synthesis, characterization (including single crystal X-ray determinations), kinetic measurement protocols, computational details (PDF)

## Supplementary Material

ja3c07780_si_001.xyz
