# Supplementary material for: Mechanistic Investigation of the Rhodium-Catalyzed Transfer Hydroarylation Reaction Involving Reversible C–C Bond Activation
Source: J Am Chem Soc. 2023 Nov 30;145(49):26657–66. doi: 10.1021/jacs.3c07780 (PMC10722515; doi:10.1021/jacs.3c07780)
Supplement: Supplementary file 2 — ja3c07780_si_002.pdf [file ja3c07780_si_002.pdf]

Supporting Information

Mechanistic Investigation of the Rhodium-Catalyzed Transfer Hydroarylation Reaction Involving Reversible C–C Bond Activation

Marius D. R. Lutz, Sven Roediger, Miguel A. Rivero Crespo, Bill Morandi*

Laboratory of Organic Chemistry, Department of Chemistry and Applied Biosciences, ETH Zürich, Vladimir-Prelog-Weg 3, 8093 Zürich, Switzerland
*Corresponding author. Email: bill.morandi@org.chem.ethz.ch

Contents

1 General Experimental Details **S8**

2 Synthesis of NHC Ligands **S9**
2.1 Preparation of **L1**·HBF_4 . . . S9
2.2 Preparation of [^{13}C]-**L1**·HBF_4 . . . S9
2.3 Preparation of free **L1** . . . S9
2.4 Preparation of free [^{13}C]-**L1** . . . S10

3 Synthesis of Organometallic Compounds **S10**
3.1 Preparation of Li-**5** . . . S10
3.2 Preparation of Na-**5** . . . S10
3.3 Preparation of K-**5** . . . S11
3.4 Preparation of aryllithiums . . . S11
3.5 Preparation of complex **1** . . . S11
3.6 Preparation of complex [^{13}C]-**1** . . . S12
3.7 Preparation of complex **3** . . . S12
3.8 Preparation of complex [^{13}C]-**3** . . . S12
3.9 Preparation of complex **4** . . . S12
3.10 Preparation of complex [^{13}C]-**4** . . . S13
3.11 Preparation of complex **S1** . . . S13
3.12 Preparation of complex **2** . . . S14
3.12.1 Screening of conditions to transmetalate to alkoxide complex **2** . . . S14
3.12.2 Preparation of complex **2** . . . S15
3.12.3 Further attempts to transmetalate **1** . . . S16
3.12.4 Alcohol exchange with hydroxy complex . . . S16
3.13 Ketone coordination complexes . . . S17
3.14 Preparation of complex [Rh(IPr)(COD)Cl] (**S2**) . . . S20

4 X-ray data **S21**
4.1 **L1** · HBF_4 . . . S22
4.2 **L1** . . . S23
4.3 Complex **4** . . . S24
4.4 Complex **S1** . . . S25

5 Influence of the Base **S26**

6 Kinetic Measurements and Analysis **S27**
6.1 Catalytic Competence of Complexes . . . S27
6.2 Kinetic Competence of Complexes . . . S27
6.3 Explanation for The Higher Rate of Formation of Ketone Product Versus Alcohol Product . . . S28
6.4 RPKA Kinetics . . . S29
6.4.1 Catalyst Deactivation (Same Excess) and Product Inhibition (Different Excess) . . . S29
6.5 VTNA Kinetics . . . S31
6.5.1 Order in complex **1** . . . S31
6.5.2 Order in alcohol **5** . . . S32
6.5.3 Order in ketone **6** . . . S32
6.5.4 Order in base . . . S33
6.5.5 Observed Rate Constant . . . S33
6.6 Initial Rates Kinetics . . . S34
6.6.1 Order in complex **1** . . . S34
6.6.2 Order in phenyl complex **4** . . . S36
6.6.3 Order in alcohol **5** . . . S38
6.6.4 Order in ketone **6** . . . S40
6.6.5 Order in base . . . S42
6.6.6 Addition of COD . . . S44
6.6.7 Order in catalyst with added COD . . . S46
6.6.8 Addition of TBACl . . . S48
6.6.9 Order in catalyst with added chloride . . . S51
6.6.10 Observed rate constant . . . S53
6.7 Validation of the Kinetic Analysis With a Machine Learning Model . . . S54
6.8 Rationalization for the Fractional Order in [Rh] . . . S56
6.8.1 Derivation for a Monomer-dimer Equilibrium . . . S56
6.8.2 Ligand Dissociation . . . S57
6.8.3 Discussion . . . S59
6.9 Derivation of Theoretical Rate Law . . . S59
6.10 Determination of Elasticity Coefficient – Order in Catalyst . . . S63

7 NMR Experiments **S65**
7.1 Reaction Monitoring . . . S65
7.1.1 Complex **1** . . . S65
7.1.2 Complex **3** . . . S69
7.1.3 Complex **3** + Chloride Source . . . S77
7.2 DOSY Experiments . . . S77
7.2.1 General Considerations . . . S77
7.2.2 Molecular Weight Range of the Resting States . . . S78

8 Computational Studies **S80**
8.1 Computational Details . . . S80
8.2 Free energy profile . . . S81
8.3 Ligand flexibility analysis . . . S83
8.4 Thermodynamics of Ion Exchange . . . S85

8.5 Summary of Calculated Energy Values S86

9 Improved Protocol for the Shuttle Arylation **S88**

Supplemental References **S89**

DFT Calculations: Cartesian Coordinates of Structures **S91**

NMR Spectra **S128**

List of Figures

S1 Stacked ^{1}H-NMR-spectra of the reaction at 25–110 °C in toluene-d_8 measured at 25 °C. S17
S2 Stacked ^{1}H-NMR-spectra of the reaction of **3** with ketones acetone, 4,4′-difluorobenzophenone and **6**. S19
S3 Stacked ^{19}F-NMR-spectra of the reaction of **3** with ketones acetone, 4,4′-difluorobenzophenone and **6**. S20
S4 Asymmetric unit of compound IPr^{*OMe} · HBF_4. . . . . S22
S5 Crystallographic structure of compound IPr^{*OMe} · HBF_4. . . . . S22
S6 Asymmetric unit of compound IPr^{*OMe}. S23
S7 Crystallographic structure of compound IPr^{*OMe}. S23
S8 Asymmetric unit of compound **4**. S24
S9 Crystallographic structure of compound **4**. S24
S10 Asymmetric unit of compound **S1**. S25
S11 Crystallographic structure of the major species, **S1**. S25
S12 Overlay of the concentration profiles of the ketone starting material (**6**) to study catalyst deactivation and product inhibition. S30
S13 Plot of the concentration profiles of **7** and **8** with varying amounts of the catalyst (**1**). S31
S14 Plot of the concentration profiles of **7** and **8** with varying amounts of alcohol substrate (**5**). S32
S15 Plot of the concentration profiles of **7** and **8** with varying amounts of ketone substrate (**6**). S32
S16 Plot of the concentration profiles of **7** and **8** with varying amounts of K_3PO_4. . . . . S33
S17 Plot of the concentration profiles of **7** and **8** in dependence of the concentrations of the catalyst (**1**), alcohol substrate (**5**), and ketone substrate (**6**). The time scale was normalized according to the VTNA method. S34
S18 Plot of the concentration profiles of **7** and **8** with varying amounts of the catalyst (**1**) S35
S19 Plot of the initial reaction rate in dependence of the concentration of the catalyst (**1**). S35
S20 Plot of the concentration profiles of **7** and **8** with varying amounts of the catalyst (**1**). S36
S21 Plot of the concentration profiles of **7** and **8** with varying amounts of the catalyst (**4**). S37
S22 Plot of the initial reaction rate in dependence of the concentration of the catalyst (**4**). S37
S23 Plot of the concentration profiles of **7** and **8** with varying amounts of the catalyst (**4**). S38
S24 Plot of the concentration profiles of **7** and **8** with varying amounts of the alcohol substrate (**5**). . . . S39
S25 Plot of the initial reaction rate in dependence of the concentration of the alcohol substrate (**5**). S39
S26 Plot of the concentration profiles of **7** and **8** with varying amounts of the alcohol substrate (**5**). . . . S40
S27 Plot of the concentration profiles of **7** and **8** with varying amounts of the ketone substrate (**6**). S41
S28 Plot of the initial reaction rate in dependence of the concentration of the ketone substrate (**6**). S41
S29 Plot of the concentration profiles of **7** and **8** with varying amounts of the ketone substrate (**6**). S42
S30 Plot of the concentration profiles of **7** and **8** with varying amounts of the base (K_3PO_4). S43
S31 Plot of the initial reaction rate in dependence of the concentration of the base (K_3PO_4). S43
S32 Plot of the concentration profiles of **7** and **8** with varying amounts of the base (K_3PO_4). S44
S33 Plot of the concentration profiles of **7** and **8** with varying amounts of COD. S45
S34 Plot of the initial reaction rate in dependence of the concentration of COD. S45
S35 Plot of the concentration profiles of **7** and **8** with varying amounts of COD. S46
S36 Plot of the concentration profiles of **7** and **8** with varying amounts of the catalyst (**1**) in presence of an excess of COD. S47
S37 Plot of the initial reaction rate in dependence of the concentration of the catalyst (**1**) in presence of an excess of COD. S47
S38 Plot of the concentration profiles of **7** and **8** with varying amounts of the catalyst (**1**) in presence of an excess of COD. S48
S39 Plot of the concentration profiles of **7** and **8** with varying amounts of the TBACl, including early time points. S49
S40 Plot of the concentration profiles of **7** and **8** with varying amounts of TBACl. S50
S41 Plot of the initial reaction rate in dependence of the concentration of TBACl. S50
S42 Plot of the concentration profiles of **7** and **8** with varying amounts of TBACl. S51

S43 Plot of the concentration profiles of **7** and **8** with varying amounts of the catalyst (**1**) in presence of an excess of TBACl. S52
S44 Plot of the initial reaction rate in dependence of the concentration of catalyst (**1**) in presence of an excess of TBACl. S52
S45 Plot of the concentration profiles of **7** and **8** with varying amounts of the catalyst (**1**) in presence of an excess of TBACl. S53
S46 Plot of the concentration profiles of **7** and **8** in dependence of the concentrations of **1**, **5** and **6**. The time scale was normalized according to the VTNA method. S54
S47 Kinetic data used for machine learning algorithm and prediction for four substrate to product pairs. . S55
S47 Kinetic data used for machine learning algorithm and prediction for four substrate to product pairs. . S56
S48 Correlation of predicted and experimental concentration data points of **7 / PA**. S63
S49 Order in [Rh] as a function of the chloride dissociation equilibrium constant K_1. . . . . S64
S50 Stacked ^{1}H-NMR-spectra of the in situ catalytic reaction with [^{13}C]-**1** in *o*-xylene-d_{10} measured at 110 °C. S66
S51 Stacked ^{13}C{^{1}H}-NMR-spectra of the in situ catalytic reaction with [^{13}C]-**1** in *o*-xylene-d_{10} measured at 110 °C. S67
S52 Stacked ^{1}H-NMR-spectra of the ex situ catalytic reaction at 125 °C with [^{13}C]-**1** in toluene-d_8 measured at 25 °C. S68
S53 Stacked ^{13}C{^{1}H}-NMR-spectra of the ex situ catalytic reaction with [^{13}C]-**1** in toluene-d_8 measured at 25 °C. S69
S54 Stacked ^{1}H-NMR-spectra of the in situ catalytic reaction with [^{13}C]-**3** in *o*-xylene-d_{10} measured at 110 °C. S70
S55 Stacked ^{13}C{^{1}H}-NMR-spectra of the in situ catalytic reaction with [^{13}C]-**3** in *o*-xylene-d_{10} measured at 110 °C. S71
S56 Stacked ^{1}H-NMR-spectra of the in situ catalytic reaction with [^{13}C]-**3** in *o*-xylene-d_{10} measured at 110 °C. S72
S57 Stacked ^{13}C{^{1}H}-NMR-spectra of the in situ catalytic reaction with [^{13}C]-**3** in *o*-xylene-d_{10} measured at 110 °C. S73
S58 Stacked ^{1}H-NMR-spectra of the ex situ catalytic reaction at 125 °C with [^{13}C]-**3** in toluene-d_8 measured at 25 °C. S74
S59 Stacked ^{13}C{^{1}H}-NMR-spectra of the ex situ catalytic reaction at 125 °C with [^{13}C]-**3** in toluene-d_8 measured at 25 °C. S75
S60 Stacked ^{1}H-NMR-spectra of the in situ catalytic reaction with [^{13}C]-**3** in *o*-xylene-d_{10} measured at 25 °C after premixing and after 8 h monitoring at 110 °C. S75
S61 Stacked ^{13}C{^{1}H}-NMR-spectra of the in situ catalytic reaction with [^{13}C]-**3** in *o*-xylene-d_{10} measured at 25 °C after premixing and after 8 h monitoring at 110 °C. S76
S62 Stacked ^{1}H-NMR-spectra of the ex situ catalytic reaction at 125 °C with [^{13}C]-**3** in toluene-d_8 measured at 25 °C and isolated alkoxide complex **2** S76
S63 Stacked ^{13}C{^{1}H}-NMR-spectra of the ex situ catalytic reaction at 125 °C with [^{13}C]-**3** in toluene-d_8 measured at 25 °C and isolated alkoxide complex **2** S76
S64 Stacked ^{1}H-NMR-spectra of the ex situ catalytic reaction at 125 °C with [^{12}C]-**3** in toluene-d_8 measured at 25 °C. S77
S65 Calibration curve for correlating the diffusion coefficient (D) from DOSY measurements. S78
S66 Plot of log molecular weight (MW) versus log diffusion coefficient (D). S79
S67 Free energy profile for the rhodium-catalyzed transfer hydroarylation between alcohol **5** and acetone. S81
S68 Free energy profile for the rhodium-catalyzed transfer hydroarylation between alcohol **5** and acetone including the ketone exchange step. S82
S69 Buried volume analysis for different complexes. S83
S70 Investigation of the effect of the peripheral phenyl groups of the NHC ligand. S84
S71 Dispersive interactions between a peripheral ligand phenyl group and an alkoxide phenyl group in complex **III**. S85
S72 Thermodynamics of ion exchange processes. S85
S73 ^{1}H-NMR spectrum of **L1** (500 MHz, toluene-d_8). S129

S74 $^{13}C\{^1H\}$-NMR spectrum of **L1** (126 MHz, toluene-d_8). . . . S130
S75 ^1H-NMR spectrum of Li-**5** (400 MHz, DMSO-d_6). . . . S131
S76 $^{13}C\{^1H\}$-NMR spectrum of Li-**5** (101 MHz, DMSO-d_6). . . . S132
S77 ^1H-NMR spectrum of Na-**5** (400 MHz, THF-d_8). . . . S133
S78 $^{13}C\{^1H\}$-NMR spectrum of Na-**5** (126 MHz, THF-d_8). . . . S134
S79 ^1H-NMR spectrum of K-**5** (400 MHz, THF-d_8). . . . S135
S80 $^{13}C\{^1H\}$-NMR spectrum of K-**5** (101 MHz, THF-d_8). . . . S136
S81 ^1H-NMR spectrum of **S1** (600 MHz, toluene-d_8). . . . S137
S82 $^{13}C\{^1H\}$-NMR spectrum of **S1** (150 MHz, toluene-d_8). . . . S138
S83 ^1H-^{103}Rh HMBC spectrum of **S1** (16 MHz, toluene-d_8). . . . S139
S84 ^1H-NMR spectrum of **2** (500 MHz, toluene-d_8). . . . S140
S85 $^{13}C\{^1H\}$-NMR spectrum of **2** (126 MHz, toluene-d_8). . . . S141
S86 ^1H-^{103}Rh HMBC spectrum of **2** (16 MHz, toluene-d_8). . . . S142
S87 ^1H-NMR spectrum of **4** (600 MHz, toluene-d_8). . . . S143
S88 $^{13}C\{^1H\}$-NMR spectrum of **4** (151 MHz, toluene-d_8). . . . S144
S89 ^1H-NMR spectrum of **4** (500 MHz, THF-d_8). . . . S145
S90 $^{13}C\{^1H\}$-NMR spectrum of **4** (126 MHz, THF-d_8). . . . S146
S91 ^1H-^{103}Rh HMBC spectrum of **4** (16 MHz, THF-d_8). . . . S147
S92 DOSY-^1H-NMR spectrum of Au(PPh_3)Cl (500 MHz, toluene-d_8). . . . S148
S93 DOSY-^1H-NMR spectrum of complex Rh(IPr)(COD)Cl (**S2**) (500 MHz, toluene-d_8). . . . S149
S94 DOSY-^1H-NMR spectrum of Grubbs 2nd generation catalyst (500 MHz, toluene-d_8). . . . S150
S95 DOSY-^1H-NMR spectrum of complex Ir(C_5Me_5)I_2 (500 MHz, toluene-d_8). . . . S151
S96 DOSY-^1H-NMR spectrum of complex **1** (500 MHz, toluene-d_8). . . . S152
S97 DOSY-^1H-NMR spectrum of complex **S1** (500 MHz, toluene-d_8). . . . S153
S98 DOSY-^1H-NMR spectrum of complex **4** (500 MHz, toluene-d_8). . . . S154
S99 DOSY-^1H-NMR spectrum of complex $Rh_2(DOSP)_4$ (500 MHz, toluene-d_8). . . . S155
S100 DOSY-^1H-NMR spectrum of the resting state **RS-1** in a catalytic reaction (500 MHz, toluene-d_8). . . S156
S101 DOSY-^1H-NMR spectrum of the resting state **RS-3** in a catalytic reaction (500 MHz, toluene-d_8). . . S157

List of Schemes

S1 Rational for protodemetalation side reaction. . . . S29
S2 Kinetic model. . . . S59

List of Tables

S1 Summary of experiments attempting to synthesize complex **2**. . . . S15
S2 Catalytic competence of Rh complexes in the absence of base. . . . S26
S3 Using alkoxides instead of alcohols. . . . S26
S4 Assessment of impact of moisture in inorganic base. . . . S26
S5 Catalytic competence of Rh complexes with reduced amount of base. . . . S27
S6 Catalytic competence of Rh complexes. . . . S27
S7 Kinetic competence of Rh complexes. . . . S28
S8 Observed rate constant (k_{obs}). . . . S33
S9 Initial rates. . . . S34
S10 Initial rates. . . . S36
S11 Initial rates. . . . S38
S12 Initial rates. . . . S40
S13 Initial rates. . . . S42

S14 Initial rates. S44
S15 Initial rates. S46
S16 Initial rates. S49
S17 Initial rates. S51
S18 Observed rate constant (k_{obs}). S53
S19 Kinetic data used as input for machine learning algorithm. S54
S20 Summary of calculated energy values. S86
S21 Summary of calculated energy values (continued). S87
S22 Catalytic competence of Rh complexes at 110 °C. S88
S23 Assessment of long term catalyst stability. S88

1 General Experimental Details

General methods All air- and moisture-sensitive manipulations were carried out using Schlenk techniques under nitrogen or in an *MBraun LABmaster Pro SP* glovebox under an argon atmosphere with a Teflon-coated magnetic stirring bar unless otherwise noted. Nitrogen was dried using a drying tube equipped with Drierite™ unless otherwise noted. All glassware was cleaned by immersion in a base-bath ($KOH_{(sat.)}$ in $^{i}PrOH_{(aq.)}$), rinsing with water, acetone and subsequently dried in a 80 °C oven prior to use. For the reactions carried out at elevated temperature, an aluminum heating block, or a oil bath (for ex situ NMR reactions) was used. Yields refer to chromatographically and spectroscopically (^{1}H-NMR) homogeneous material, unless otherwise stated. Reactions were monitored by NMR and GC.

Chemicals Chemicals were purchased from common suppliers and used without further purification, unless noted else. Chloro(1,5-cyclooctadiene)rhodium(I) dimer ($[Rh(COD)Cl]_2$) and hydroxy(1,5-cyclooctadiene)rhodium(I) dimer ($[Rh(COD)(OH)]_2$) were purchased from ABCR and was stored in a glovebox. Silver tetrafluoroborate ($AgBF_4$) was purchased from Apollo and was stored in a glovebox. Anhydrous potassium phosphate tribasic (K_3PO_4) was purchased from Sigma-Aldrich and stored in a glovebox.

Solvents The solvents used for air- and moisture-sensitive manipulations were dried and deoxygenated using literature procedures.[1] Toluene (PhMe), tetrahydrofuran (THF), and hexane for synthetic reactions were obtained by passing the previously degassed solvents through an activated alumina column (LC Technology Solutions solvent purification system) and stored over activated molecular sieves under nitrogen. Anhydrous dichloromethane (CH_2Cl_2) was purchased from Acros over activated molecular sieves.

Deuterated organic solvents were distilled over potassium (toluene-d_8, THF-d_8), or distilled over CaH_2 and degassed by a freeze-pump-thaw cycle (CD_2Cl_2), and stored over molecular sieves before use.

Nuclear magnetic resonance (NMR) spectroscopy NMR spectra were acquired on commercial instruments (*Bruker Avance III 400 MHz*, *Bruker Neo 400 MHz*, *Bruker Avance III 500 MHz*, all equipped with a BBFO probe; *Bruker Avance III 600 MHz*, equipped with a DCH probe; *Bruker Avance III 600 MHz*, equipped with a Prodigy TCI triple resonance probe with z-gradients) at the NMR facility of ETH Zürich. All spectra were acquired at 298 K unless stated otherwise. ^{1}H-NMR chemical shifts are reported relative to the residual non-deuterated solvent signal: chloroform-d ($CDCl_3$, δ = 7.26 ppm), toluene-d_8 (δ = 2.08 ppm), and CD_2Cl_2 (δ = 5.32 ppm), respectively.[2] ^{13}C{^{1}H}-NMR chemical shifts are reported relative to the solvent signal: chloroform-d ($CDCl_3$, δ = 77.16 ppm), toluene-d_8 (δ = 20.43 ppm), and CD_2Cl_2 (δ = 53.84 ppm), respectively.[2] ^{13}C and ^{19}F-NMR spectra were acquired proton-decoupled and signals are singlets unless stated otherwise. ^{103}Rh signals were detected indirectly in a ^{1}H-^{103}Rh HMBC experiment (pulse sequence: `hmbcgpndqf`). Spectral data are reported as followed: chemical shift δ/ppm, multiplicity (s = singlet, d = doublet, t = triplet, q = quartet, br = broad, m = multiplet, app = apparent; or combinations thereof.

Gas chromatography (GC) GC measurements were conducted on a *Shimadzu GC-2025 Series* GC system. A quartz capillary column *Macherey-Nagel OPTIMA 5* (30.0 m × 0.25 mm × 0.25 µm, carrier gas: hydrogen) was used. The carrier gas rate was 68.6 cm s^{-1} and the injection temperature 250 °C. A flame ionization detector (FID) with an inlet temperature of 250 °C was used. To determine GC yields, calibration curves were generated using n-dodecane as an internal standard.

High resolution mass spectrometry (HRMS) HRMS data was obtained using electron ionisation (EI) on a *Thermo scientific Q Exactive GC Orbitrap* with direct Probe, electrospray ionisation (ESI) on a *Bruker maXis – ESI-Qq-TOF-MS* or matrix-assisted laser desorption/ionisation (MALDI) on *Bruker solariX – MALDI-FTICR-MS* and are reported in m/z. All published data are within a range of m/z ± 3 ppm of theoretical values.

Infrared (IR) spectroscopy ATR-FTIR spectra were recorded in an argon-filled glovebox on a Bruker ALPHA II FT-IR spectrometer. The absorption bands are reported in cm^{-1} and are described as follows: strong (s), medium (m), weak (w), broad (br), and shoulder (sh).

2 Synthesis of NHC Ligands

2.1 Preparation of L1 · HBF_4

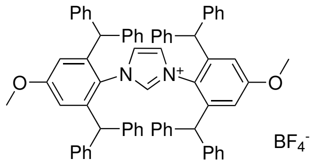

IPr*OMe · HBF_4 (L1 · HBF4)

NHC ligand IPr*OMe · HBF_4 (**L1** · HBF_4) was prepared as previously reported.[3]

Single crystals suitable for X-ray diffraction analysis were obtained by vapor diffusion of hexane into a toluene solution at rt (cf. Section 4.1).

2.2 Preparation of [^{13}C]-L1 · HBF_4

^{13}C-labeled IPr*OMe · HBF_4 ([^{13}C]-**L1** · HBF_4) was prepared through a procedure identical to that for the synthesis of natural abundance **L1** · HBF_4, except that ^{13}C-enriched paraformaldehyde was used in the cyclization step.

The ^{1}H and ^{13}C{^{1}H}-NMR spectral data were identical to those of unlabeled **L1** · HBF_4.[3]

NMR shifts of the labeled carbon:

^{1}H-NMR (500 MHz, $CDCl_3$): δ 10.13 (d, J = 227.3 Hz, 1 H).
^{13}C{^{1}H}-NMR (126 MHz, $CDCl_3$): δ 141.0.

2.3 Preparation of free L1

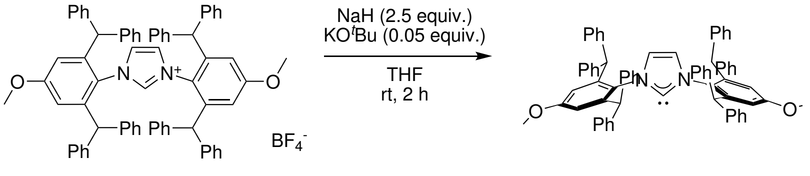

IPr*OMe · HBF_4 **IPr*OMe (L1)**

NHC ligand IPr*OMe (**L1**) was prepared as previously reported.[3]

The ^{1}H and ^{13}C{^{1}H}-NMR spectral data in THF-d_8 were identical to those of unlabeled **L1**.[3]

^{1}H-NMR (500 MHz, toluene-d_8): δ 7.32 – 7.27 (m, 8 H), 7.10 – 7.03 (m, 12 H), 7.02 – 6.89 (m, 24 H), 6.82 (s, 4 H), 5.95 (s, 4 H), 5.75 (s, 2 H), 3.14 (s, 6 H).
^{13}C{^{1}H}-NMR (126 MHz, toluene-d_8): δ 221.0, 159.6, 144.9, 143.7, 143.5, 137.5, 133.9, 130.2, 129.9, 129.1, 128.9, 128.7, 128.6, 128.6, 128.5, 128.5, 128.3, 128.3, 128.3, 128.2, 128.2, 128.2, 128.0, 128.0, 128.0, 127.8, 127.8, 127.8, 126.5, 125.3, 125.1, 125.1, 125.1, 124.9, 122.6, 114.8, 54.5, 51.7.
Single crystals suitable for X-ray diffraction analysis were obtained by vapor diffusion of hexane into a toluene solution at rt (cf. Section 4.2).

2.4 Preparation of free [^{13}C]-L1

^{13}C-labeled IPr^{*OMe} ([^{13}C]-**L1**) was prepared through a procedure identical to that for the synthesis of natural abundance **L1**.[3]

The ^{1}H and ^{13}C{^{1}H}-NMR spectral data were identical to those of unlabeled **L1**.[3]

NMR shift of the labeled carbon:

^{13}C{^{1}H}-NMR (126 MHz, THF-d_8): δ 221.9.

3 Synthesis of Organometallic Compounds

3.1 Preparation of Li-5

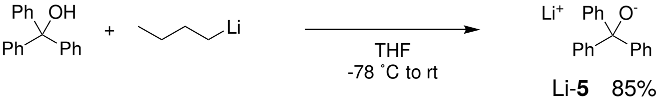

Li-**5** 85%

An oven-dried 20 mL Schlenk tube equipped with a magnetic stirring bar was placed under nitrogen and was charged with triphenylmethanol (521 mg, 2.00 mmol, 1.0 equiv.). The apparatus was evacuated and back-filled with nitrogen three times. Then, THF (10 mL) was added. The resulting solution was cooled to −78 °C and *n*-butyllithium solution (1.25 mL, 1.6 M in hexane, 2.00 mmol, 1.0 equiv.) was added dropwise via syringe at −78 °C. The mixture was stirred at 25 °C for 75 min. The volatiles were removed on the Schlenk line and the crude material was dried to give a white solid. The solid was washed with hexane (7 mL). The supernatant was removed by cannula, and the solid was washed with hexane (1×7 mL) and dried under high vacuum to yield 452 mg (85%) of lithium triphenylmethanolate (Li-**5**) as an off-white solid.

^{1}H-NMR (400 MHz, DMSO-d_6): δ 7.31 – 7.12 (m, 15 H).
^{13}C{^{1}H}-NMR (101 MHz, DMSO-d_6): δ 149.8, 127.9, 127.2, 126.0, 81.2.

3.2 Preparation of Na-5

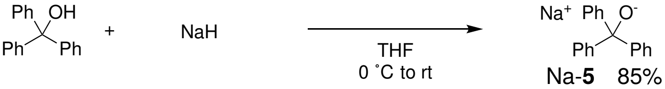

Na-**5** 85%

An oven-dried 50 mL Schlenk round-bottom flask equipped with a magnetic stirring bar was placed under nitrogen and was charged with NaH (323 mg, 60weight%, 8.07 mmol, 1.05 equiv.) and hexane (10 mL). After stirring for 5 min, the solvent was removed via syringe. The resulting grey solid was suspended in THF (20 mL) to afford a light grey suspension. Another oven-dried 25 mL Schlenk round-bottom flask equipped with a magnetic stirring bar was placed under nitrogen and was charged with triphenylmethanol (2.00 g, 7.68 mmol, 1.0 equiv.). The apparatus was evacuated and back-filled with nitrogen three times. Then, THF (15 mL) was added. The NaH-containing suspension was cooled to 0 °C and the alcohol solution was added dropwise via cannula at 0 °C. The mixture was stirred at 25 °C for 5 h. The volatiles were removed on the Schlenk line and the crude material was dried to give a white solid. The solid was washed with hexane (5×2 mL) and the supernatant was removed by decantation. The white solid was dried under high vacuum to yield 1.99 g (83%) of sodium triphenylmethanolate (Na-**5**) as a white solid.

^{1}H-NMR (400 MHz, THF-d_8): δ 7.36 – 7.30 (m, 6 H), 7.15 – 7.07 (m, 6 H), 7.07 – 6.98 (m, 3 H).
^{13}C{^{1}H}-NMR (126 MHz, THF-d_8): δ 156.5, 129.0, 127.3, 125.3, 84.2.

3.3 Preparation of K-5

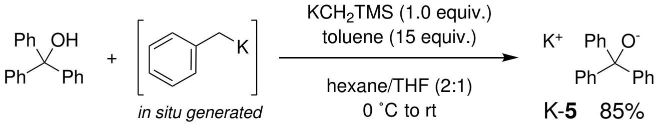

In a glovebox a 50 mL Schlenk tube equipped with a magnetic stirring bar was charged with ((trimethylsilyl)methyl)-potassium[4,5] (400 mg, 3.17 mmol, 1.0 equiv.). The flask was cycled on the line and hexane (10 mL) was added to give a light grey suspension. After stirring for 5 min, the solvent was removed via syringe. The resulting grey solid was suspended in THF (20 mL) to afford a light grey suspension. The mixture was cooled to 0 °C and toluene (5.0 mL, 47.5 mmol, 15 equiv.) was added dropwise. The resulting mixture was warmed to rt and stirred for 1 h. The color slowly changed to an intense orange. Another oven-dried 25 mL Schlenk round-bottom flask equipped with a magnetic stirring bar was placed under nitrogen and was charged with triphenylmethanol (824 mg, 3.17 mmol, 1.0 equiv.). The apparatus was evacuated and back-filled with nitrogen three times. Then, THF (5 mL) was added. The alcohol solution was added dropwise via cannula at 0 °C. The Schlenk was washed with THF (1×1 mL). The mixture was stirred at 25 °C for 1 h. The volatiles were removed on the Schlenk line to give a white solid, which was dried under high vacuum to yield 0.856 g (91%) of potassium triphenylmethanolate (K-**5**) as a white solid.

^{1}H-NMR (400 MHz, THF-d_8): δ 7.33 – 7.19 (m, 6 H), 7.13 – 6.90 (m, 9 H).
^{13}C{^{1}H}-NMR (151 MHz, THF-d_8): δ 157.7, 129.0, 127.9, 125.3, 86.3.

3.4 Preparation of aryllithiums

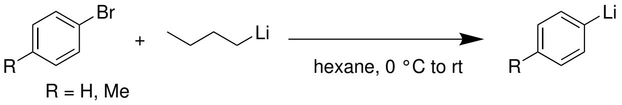

Phenyllithium and *p*-tolyllithium were prepared according to literature procedures.[6,7]

3.5 Preparation of complex 1

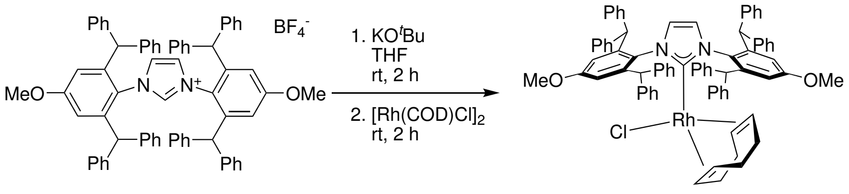

L1 · HBF$_4$

1
97%

[Rh(IPr^{*OMe})(COD)Cl] (**1**) was prepared as previously reported.[8]

3.6 Preparation of complex [^{13}C]-1

^{13}C-labeled [Rh(IPr^{*OMe})(COD)Cl] ([^{13}C]-**1**) was prepared through a procedure identical to that for the synthesis of natural abundance [Rh(IPr^{*OMe})(COD)Cl] (**1**), except that the free carbene [^{13}C]-**L1** was used in the synthesis.

The ^{1}H and ^{13}C{^{1}H}-NMR spectral data were identical to those of unlabeled **1**.

NMR shift of the labeled carbon:

^{13}C{^{1}H}-NMR (101 MHz, toluene-d_8): δ 186.3 (d, J = 51.9 Hz).
^{13}C{^{1}H}-NMR (75 MHz, CDCl$_3$): δ 184.7 (d, J = 51.9 Hz).

3.7 Preparation of complex 3

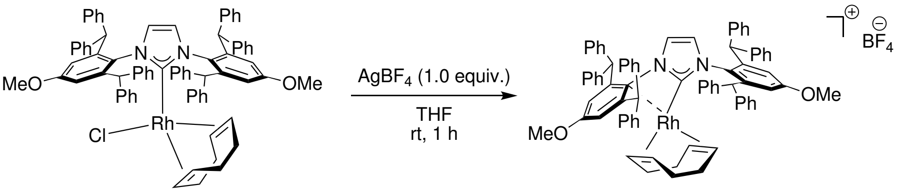

1 **3** **82%**

[Rh(IPr^{*OMe})(COD)][BF$_4$] (**3**) was prepared as previously reported.[9]

3.8 Preparation of complex [^{13}C]-3

^{13}C-labeled [Rh(IPr^{*OMe})(COD)][BF$_4$] ([^{13}C]-**3**) was prepared through a procedure identical to that for the synthesis of natural abundance [Rh(IPr^{*OMe})(COD)][BF$_4$] (**3**), except that [^{13}C]-**1** was used in the synthesis. The ^{1}H and ^{13}C{^{1}H}-NMR spectral data were identical to those of unlabeled **3**.

NMR shift of the labeled carbon:

^{13}C{^{1}H}-NMR (101 MHz, CD$_2$Cl$_2$): δ 184.8–184.0 (br s, $\nu_{1/2}$ = 33 Hz), 181.8–180.6 (br s, $\nu_{1/2}$ = 33 Hz).

3.9 Preparation of complex 4

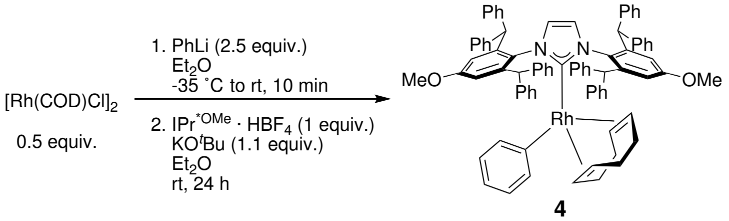

4 **47%**

In a glovebox, a 8 mL vial equipped with a magnetic stirring bar under argon was charged with [Rh(COD)Cl]$_2$ (100.0 mg, 202.8 µmol, 0.5 equiv.) and Et$_2$O (5.0 mL). The resulting yellow suspension was cooled to −35 °C in a glovebox freezer for 20 min. Likewise, a 4 mL vial equipped with a magnetic stirring bar under argon was charged with phenyllithium (85.2 mg, 1.01 mmol, 2.5 equiv.) and Et$_2$O (2.0 mL). The resulting colorless solution was cooled to −35 °C in a glovebox freezer for 20 min. In a glovebox, a 8 mL vial equipped with a magnetic stirring bar under argon was charged with **L1** · HBF$_4$ (419.0 mg, 405.6 µmol, 1.0 equiv.) and KO^{t}Bu (50.1 mg, 446.2 µmol, 1.10 equiv.). Et$_2$O (5.0 mL) was

added and the off white suspension was stirred at 25 °C for 20 min. The cold phenyllithium solution was added to the rhodium-containing vial and the mixture was returned to the freezer for 2 min, then stirred at 25 °C for 5 min. The reaction mixture was added to the vial containing the NHC suspension, and the resulting orange mixture was stirred at 25 °C for 24 h.

The reaction mixture was transferred into a 50 mL Schlenk round-bottom flask, and the residue washed over with hexane (2×2 mL). The supernatant was removed by cannula filtration and discarded. The orange solid was washed with hexane (2×6 mL), then dissolved in toluene (9 mL) and cannula filtered into another 50 mL Schlenk round-bottom flask. The solution was concentrated until a solid started to form, and was returned to a glovebox. The saturated solution was filtered and layered with hexane to afford orange crystals. Decantation of the supernatant and drying afforded [Rh(IPr^{*OMe})(Ph)(COD)] (**4**) as orange crystals (247.2 mg, 47%).

^{1}H-NMR (500 MHz, toluene-d_8): δ 7.44 (s, 4 H), 7.19 – 6.64 (m, 47 H), 6.52 (s, 2 H), 5.19 (s, 2 H), 4.52 (s, 2 H), 4.34 (br s, 2 H), 3.15 (s, 6 H), 2.64 (br , 2 H), 2.26 (br s, 2 H), 1.97 (br s, 2 H), 1.77 (br s, 2 H).
^{13}C{^{1}H}-NMR (151 MHz, toluene-d_8): δ 188.4 (d, J = 59.1 Hz), 177.1 (d, J = 34.9 Hz), 159.3, 129.8, 126.7, 126.5, 126.2, 125.6, 124.1, 120.9, 116.5, 116.1, 89.0 (d, J = 10.0 Hz), 85.2, 54.6, 52.0.
^{1}H-NMR (600 MHz, THF-d_8): δ 7.30 – 6.66 (m, 41 H), 6.59 – 6.38 (m, 10 H), 6.25 (s, 2 H), 4.97 (s, 2 H), 4.08 (s, 4 H), 3.51 (s, 6 H), 2.48 (br s, 2 H), 2.08 (s, 2 H), 1.89 (s, 2 H), 1.71 (s, 2 H).
^{13}C{^{1}H}-NMR (151 MHz, THF-d_8): δ 188.6 (d, J = 59.1 Hz), 178.0 (d, J = 35.2 Hz), 159.9, 145.7, 145.5, 145.1, 144.7, 144.3, 143.9, 138.6, 134.4, 131.6, 131.3, 130.4, 129.3, 128.9, 128.8, 127.5, 127.4, 127.1, 126.9, 126.3, 124.7, 120.9, 116.8, 116.6, 88.9 (d, J = 9.8 Hz), 86.0, 55.5, 52.7, 52.5, 31.6 (d, J = 34.2 Hz).
^{103}Rh-NMR (16 MHz, THF-d_8): δ -7772.
IR (ATR) $\tilde{\nu}$ = 3026 (w), 2939 (w), 2837 (w), 1597 (m), 1493 (m), 1462 (m), 1445 (m), 1299 (m), 1269 (m), 1230 (m), 1146 (w), 1082 (m), 1046 (w), 1031 (w), 961 (w), 914 (w), 879 (w), 854 (w), 763 (m), 730 (w), 698 (s), 647 (w), 622 (w), 602 (m), 583 (w), 502 (w) cm^{-1}.
HRMS (*m/z*): [M−COD]$^+$ calcd for $C_{75}H_{61}N_2O_2Rh$ 1124.3788, found 1124.3852.
Anal. Calcd for $C_{83}H_{73}N_2O_2Rh$: C, 80.83; H, 5.97; N, 2.27. Found: C, 80.90; H, 6.16; N, 2.57.
Single crystals suitable for X-ray diffraction analysis were obtained by slow diffusion of hexane into a toluene solution (cf. Section 4.3).

3.10 Preparation of complex [^{13}C]-4

^{13}C-labeled [Rh(IPr^{*OMe})(Ph)(COD)] ([^{13}C]-**4**) was prepared through a procedure identical to that for the synthesis of natural abundance [Rh(IPr^{*OMe})(Ph)(COD)] (**4**), except that the free carbene [^{13}C]-IPr^{*OMe} was used in the synthesis. The ^{1}H and ^{13}C{^{1}H}-NMR spectral data were identical to those of unlabeled **4**.

NMR shift of the labeled carbon:

^{13}C{^{1}H}-NMR (101 MHz, toluene-d_8): δ 188.4 (d, J = 59.1 Hz)

3.11 Preparation of complex S1

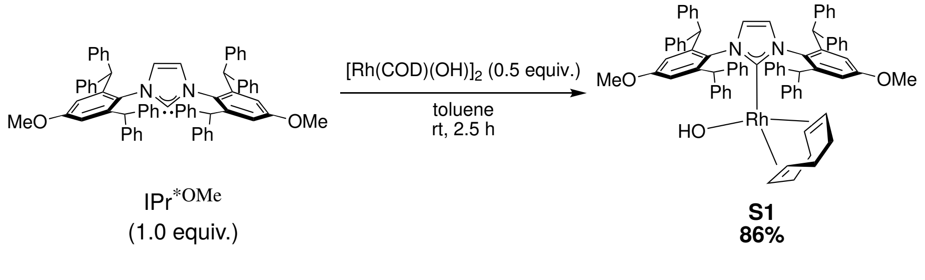

In a glovebox, a 8 mL-vial equipped with a magnetic stirring bar under argon was charged with $[Rh(COD)(OH)]_2$ (50.0 mg, 109.6 µmol, 0.5 equiv.), **L1** · 0.7 THF (218.3 mg, 219.2 µmol, 1.0 equiv.), and toluene (4.0 mL). The yellow reaction mixture was stirred at 25 °C for 2.5 h to give a yellow cloudy solution. The reaction mixture was transferred into a Schlenk round-bottom flask, cycled onto the Schlenk line, and concentrated there until a yellow solid started to precipitate. Hexane (5 mL) was added, inducing precipitation of a yellow solid. The supernatant was removed by cannula, and the solid was washed with pentane (2×2 mL) and dried under high vacuum to yield $[Rh(IPr^{*OMe})(COD)(OH)]$ (**S1**) (221.3 mg, 86%) as a yellow solid.

^{1}H-NMR (500 MHz, toluene-d_8): δ 7.58 (br s, 8 H), 7.16 (t, J = 7.6 Hz, 8 H), 7.11 – 6.81 (m, 33 H), 4.90 (s, 2 H), 4.58 – 4.54 (m, 2 H), 3.59 – 3.54 (m, 2 H), 3.20 (s, 6 H), 2.13 – 2.08 (m, 2 H), 2.02 – 1.94 (m, 2 H), 1.73 – 1.62 (m, 4 H).
^{13}C{^{1}H}-NMR (151 MHz, toluene-d_8): δ 190.8 (d, J = 55.8 Hz), 159.8, 145.3, 132.5, 131.0, 130.1, 128.3, 128.2, 126.6, 126.4, 123.4, 114.9, 91.9 (d, J = 8.1 Hz), 64.7 (d, J = 11.8 Hz), 54.6, 51.7, 33.5, 29.3.
^{103}Rh-NMR (16 MHz, toluene-d_8): δ -7460.
IR (ATR) $\tilde{\nu}$ = 3024 (w), 2836 (w), 1597 (m), 1493 (m), 1463 (m), 1440 (m), 1300 (m), 1236 (m), 1146 (w), 1086 (m), 1050 (w), 1031 (w), 961 (w), 916 (w), 853 (w), 762 (m), 738 (m), 698 (s), 651 (w), 622 (w), 603 (m), 584 (w), 501 (w) cm^{-1}.
Anal. Calcd for $C_{77}H_{69}N_2O_3Rh$: C, 78.82; H, 5.93; N, 2.39. Found: C, 80.44; H, 6.05; N, 2.56.
Single crystals suitable for X-ray diffraction analysis were obtained by slow diffusion of hexane into a toluene solution (Section 4.4).

3.12 Preparation of complex 2

3.12.1 Screening of conditions to transmetalate to alkoxide complex 2

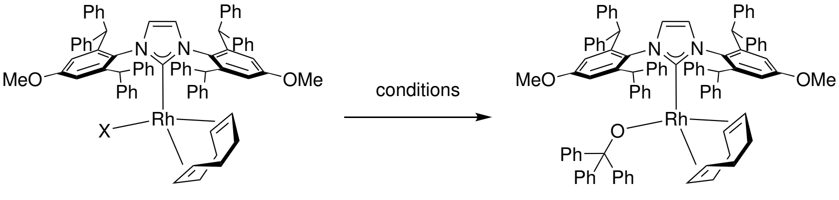

X = Cl, OH, BF_4, Ph **2**

Table S1: Summary of experiments attempting to synthesize complex **2**.

Conditions	Temp.	Result
Starting from complex $[Rh(COD)Cl]_2$		
Na-**5**, THF, then IPr^{*OMe}	−78–25 °C	**1**
Starting from complex $[Rh(IPr^{*OMe})(COD)Cl]$ (**1**)		
Li-**5**, toluene	rt	no reaction
Na-**5**, toluene	rt	no reaction
K-**5**, toluene	rt	no reaction
K-**5**, toluene	110 °C	decomposition to multiple products, including **S1**, **8**
K-**5**, THF	rt	no reaction
**5**, KHMDS, toluene	rt	no reaction
**5**, KHMDS, toluene	rt, then 80 °C	no reaction
**5**, KHMDS, THF	rt	no reaction
**5**, NaH, toluene	25–110 °C	no reaction
$AgBF_4$, then Na-**5**, THF	rt	complex mixture
$AgBF_4$ (2 equiv.), then Na-**5** (2 equiv.), THF	rt	**product observed**, mixture with **1** and other species
$AgBF_4$ (1.5 equiv.), Na-**5** (2 equiv.)[a], THF	rt	**product observed**, mixture with sm
Na-**5** (1.1 equiv.), then $AgBF_4$ (2 equiv.), toluene	rt	**product observed (major)**
Na-**5**, then AgOTf, THF	rt	no reaction
Starting from complex $[Rh(IPr^{*OMe})(COD)(OH)]$ (**S1**)		
**5**, toluene	25–110 °C	no reaction, formation of **8** and benzene (*vide infra*)
$[Ph_3C][BF_4]$, THF	25 °C	**product observed**, mixture with sm
Starting from complex $[Rh(IPr^{*OMe})(COD)]BF_4$ (**3**)		
Li-**5**, Et_2O	rt	no reaction
Na-**5**, THF	rt	complex mixture, including sm, **S1**
K-**5**, THF	rt	**S1**
**5**, KHMDS, toluene	rt	complex mixture
**5**, KHMDS, $AgBF_4$ (0.1 equiv.)[a], THF	rt	**product observed (major)**
**5**, KHMDS, THF	rt	complex mixture
**5**, K_3PO_4 (5 equiv.), toluene	25–80 °C	**product observed (major)**, but low conversion during scale-up
**5**, K_3PO_4 (5 equiv.), toluene	125 °C	**product observed (major)**, formation of **8**
**5**, K_3PO_4 (5 equiv.), THF	25 °C	**product observed (major)**, scale-up successful
Starting from complex $[Rh(IPr^{*OMe})(Ph)(COD)]$ (**4**)		
**5**, toluene	25–80 °C	complex mixture
**5**, K_3PO_4, toluene	25–125 °C	complex mixture

[a] One pot – added alkoxide before filtering off silver salts

3.12.2 Preparation of complex 2

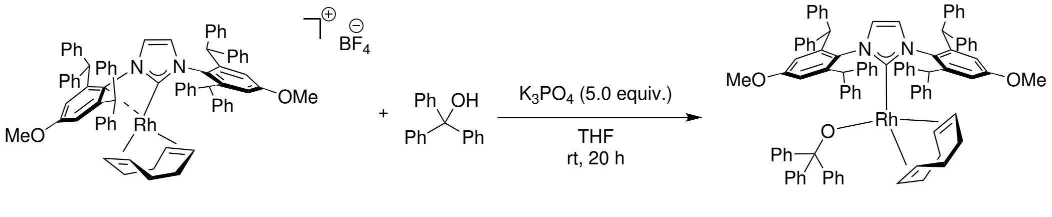

3

2
23%

In a glovebox, a 50 mL Schlenk round-bottom flask was charged with **3** (58.5 mg, 47.1 µmol, 1.0 equiv.), **5** (12.3 mg,

47.1 µmol, 1.0 equiv.) and K_3PO_4 (49.9 mg, 235 µmol, 5.0 equiv.). THF (2 mL) was added to afford a faintly yellow solution with yellow/white precipitate. The reaction mixture was stirred at 25 °C for 20 h. After this time a yellow solution with small amounts of a white precipitate was obtained. The reaction was cannula filtered into another Schlenk flask. The residual solid was washed with THF (2 mL). The resulting yellow solution was concentrated to give a yellow gel which was triturated with hexane (5 mL) to precipitate a yellow solid. The solid was washed with hexane (2×1 mL) and the solid dried to afford **2** as a light yellow solid (15.5 mg, 23%).

^{1}H-NMR (500 MHz, toluene-d_8): δ 7.70 (s, 8 H), 7.18 (dd, J = 8.3, 7.2 Hz, 8 H), 7.11 – 6.99 (m, 9 H), 6.97 (dd, J = 1.6, 0.8 Hz, 2 H), 6.93 (s, 4 H), 6.89 – 6.85 (m, 15 H), 6.86 – 6.77 (m, 6 H), 5.13 (br s, 2 H), 4.73 (br s, 2 H), 3.51 (s, 2 H), 3.24 (s, 6 H), 2.02 – 1.83 (m, 4 H), 1.65 – 1.50 (m, 4 H). The aromatic region shows a smaller integral than expected.
^{13}C{^{1}H}-NMR (126 MHz, toluene-d_8): δ 188.7 (d, J = 54.9 Hz), 160.1, 145.6, 144.3, 131.7, 131.3, 130.0, 128.5, 126.8, 126.4, 123.2, 114.7, 96.7 (d, J = 7.5 Hz), 82.1, 62.3 (d, J = 14.2 Hz), 54.7, 51.9, 33.5, 28.4. One carbon was not observed.
^{103}Rh-NMR (16 MHz, toluene-d_8): δ -7279.
IR (ATR) $\tilde{\nu}$ = 2875, 1595, 1493, 1463, 1441, 1301, 1213, 1145 ,1086, 1048, 1031, 951, 853, 760,697, 621, 603, 582, 543, 442 cm^{-1}.

3.12.3 Further attempts to transmetalate 1

Conditions	Result
LiHMDS, THF, 60 °C	no reaction
KHMDS, THF, 25 °C	no reaction
K_3PO_4, THF, 50 °C	no reaction
KO^{t}Bu, toluene, 25–80 °C	decomposition
pyridine, toluene, 25–110 °C	no reaction

3.12.4 Alcohol exchange with hydroxy complex

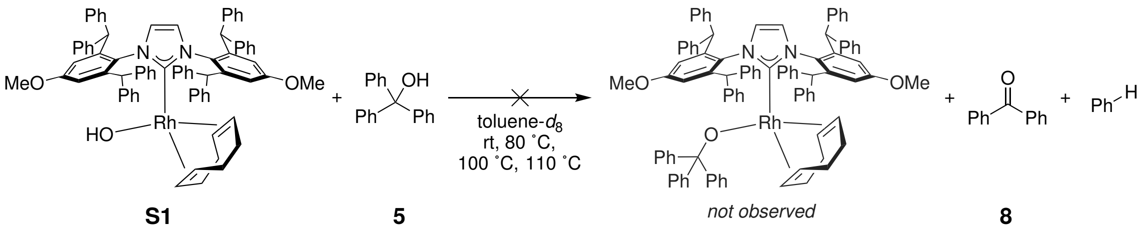

In a glovebox, a 4 mL-vial was charged with **S1** (10.0 mg, 8.52 µmol, 1.0 equiv.), **5** (2.2 mg, 8.52 µmol, 1.0 equiv.) and toluene-d_8 (0.5 mL), affording a yellow solution.

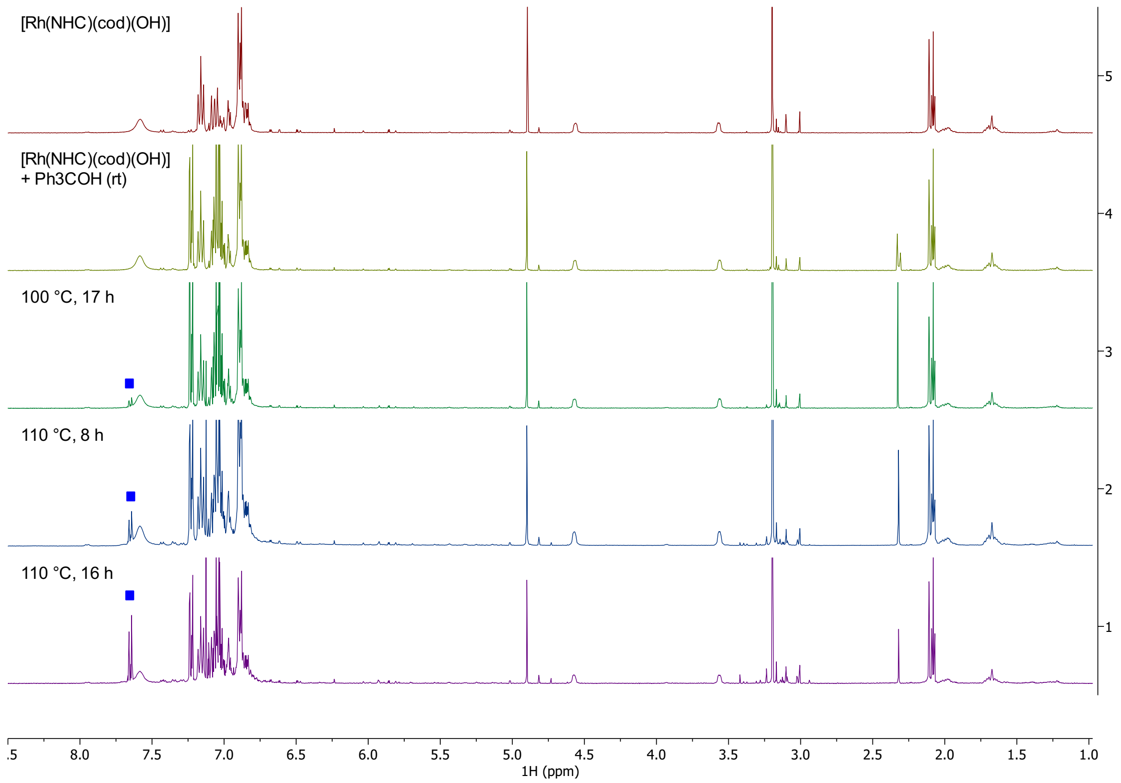

Figure S1: Stacked ^{1}H-NMR-spectra of the reaction at 25–110 °C in toluene-d_8 measured at 25 °C. No reaction occurred until 100 °C, at 110 °C benzophenone (**8**) (blue squares) slowly forms with no new product being formed.

No reaction occurred at either room temperature or up to 100 °C. After prolonged heating at 110 °C benzophenone (**8**) and benzene formed, however, the signals of the complex did not change. Possible explanations are that hydroxy complex **S1** is more stable than the corresponding alkoxide, or the association of the alcohol is rate-determining and subsequent β-carbon elimination is fast.

3.13 Ketone coordination complexes

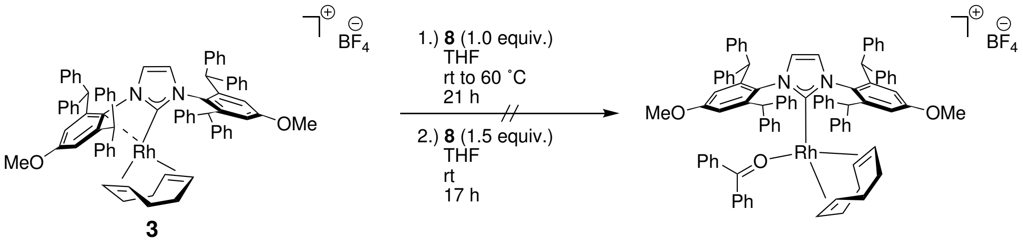

In a glovebox, a 25 mL Schlenk round-bottom flask equipped with a magnetic stirring bar under argon was charged with **3** (50.4 mg, 40.5 µmol, 1.0 equiv.), **8** (7.4 mg, 40.5 µmol, 1.0 equiv.), was brought out of a glovebox, and cycled onto the Schlenk line. THF (5.0 mL) was added to, affording a light yellow solution. The reaction mixture was stirred at 60 °C for 21 h, after which the volatiles were removed to afford a yellow solid. The solid was washed with hexane and dried.

NMR analysis showed the signals of free benzophenone (**8**) and **3**.

The solid was dissolved again in THF (1.5 mL) and **8** (11.1 mg, 60.8 µmol, 1.5 equiv.) was added. The reaction mixture was stirred at 25 °C for 17 h, after which the volatiles were removed to afford a yellow oily residue. It was taken up in toluene and hexane (2:1, 2 + 1 mL). After brief stirring a yellow solid precipitated. The supernatant was removed by cannula filtration and the solid was washed with toluene and hexane (2:1, 2 + 1 mL) and hexane (2 mL) affording a yellow solid which was dried.

NMR analysis of the solid revealed that the peaks of **8** were gone and the peaks of **3** were unchanged. Therefore, the starting complex was reisolated.

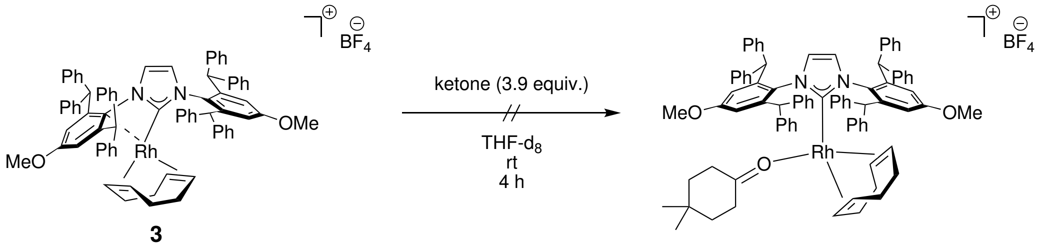

In a glovebox, a 4 mL vial was charged with **3** (11.0 mg, 8.85 µmol, 1.0 equiv.), **6** (4.4 mg, 35 µmol, 3.9 equiv.) and THF-d_8 (0.5 mL), affording a yellow suspension (complex partially dissolved).

NMR analysis after 30 min and 4 h showed no significant changes, except small shifts in most peaks which were generally observed with this complex based on the concentration.

After 4 h the NMR sample was brought back into glovebox, and poured into 2 mL hexane. The cloudy suspension was agitated, and then allowed to settle. The cloudy supernatant was removed and the solid was washed with hexane and dried.

NMR analysis of the solid revealed that the peaks of **6** disappeared and the peaks of **3** were unchanged. Therefore, the starting complex was reisolated.

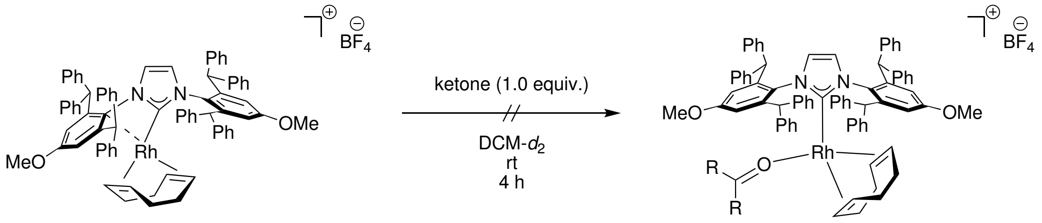

In a glovebox, a 4 mL-vial was charged with **3** (10.0 mg, 8.0 µmol, 1.0 equiv.), ketone (**6**: 1.0 mg, 8.0 µmol, 1.0 equiv.; 4,4′-difluorobenzophenone: 1.8 mg, 8.0 µmol, 1.0 equiv.; acetone: 3 drops, >24 µmol, >5.0 equiv.) and CD_2Cl_2 (0.5 mL), affording a yellow solution. The reaction mixture was closed and left for 4 h at 25 °C without stirring, before being concentrated. The resulting solid was washed with hexane (2×1 mL) and dried. The obtained yellow solid was analyzed by ^{1}H-NMR- and ^{19}F-NMR spectroscopy.

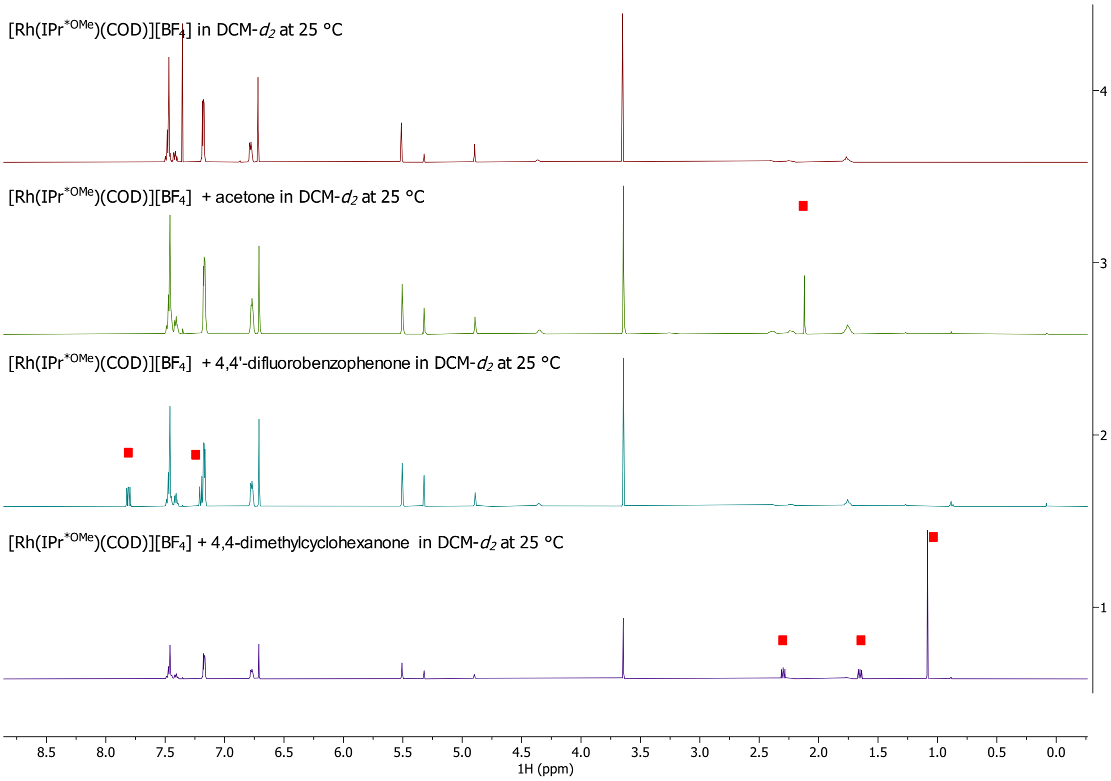

Figure S2: Stacked ^{1}H-NMR-spectra of the reaction of **3** with ketones acetone, 4,4′-difluorobenzophenone and **6** in CD_2Cl_2. The signals of the non-coordinated ketones are indicated with red squares.

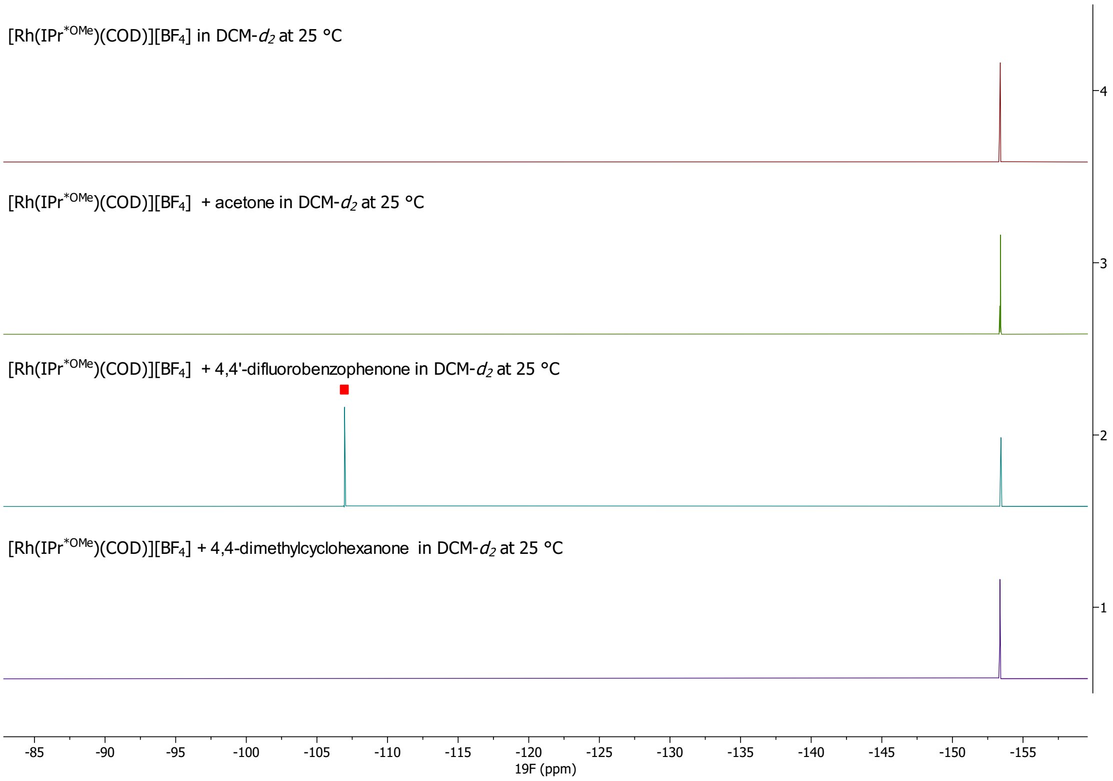

Figure S3: Stacked ^{19}F-NMR-spectra of the reaction of **3** with ketones acetone, 4,4′-difluorobenzophenone and **6** in CD_2Cl_2. The signals of the non-coordinated ketones are indicated with red squares.

NMR analysis of the isolated products indicated that no reaction took place in all cases, as all peaks of **3** were unchanged.

3.14 Preparation of complex [Rh(IPr)(COD)Cl] (S2)

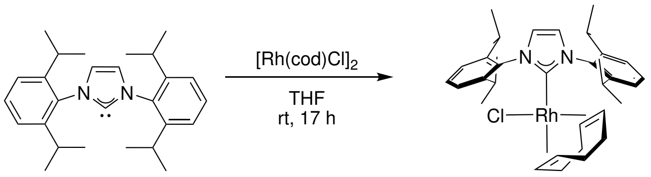

IPr **[Rh(IPr)(cod)Cl]**

[Rh(IPr)(COD)Cl] (**S2**) was prepared as previously reported.[10]

4 X-ray data

General information

Single crystalline samples were measured on the following instruments:

- *Bruker/Nonius Kappa APEX-II* diffractometer with microfocus sealed tube Mo-Kα radiation using mirror optics (λ = 0.71073 Å).
- *Rigaku Oxford Diffraction XtaLAB Synergy-S Dualflex kappa* diffractometer equipped with a *Dectris Pilatus 300 HPAD* detector and using microfocus sealed tube Cu-Kα radiation with mirror optics (λ = 1.54178 Å).

All measurements were carried out at 100 K (unless otherwise noted) using an *Oxford Cryosystems Cryostream 800* sample cryostat. Data collected on *Bruker* instruments were integrated using *SAINT* from the *Bruker Apex-II* program suite and corrected for absorption effects using the multi-scan method (*SADABS*).[11] Data collected on the Rigaku instrument were integrated using *CrysAlisPro* and corrected for absorption effects using a combination of empirical (*ABSPACK*) and numerical corrections.[i] The structures were solved using *SHELXT*[12] or *SHELXS*[13] and refined by full-matrix least-squares analysis (*SHELXL*),[14] using the program package *OLEX2*.[15] Unless otherwise indicated below, all non-hydrogen atoms were refined anisotropically and hydrogen atoms were constrained to ideal geometries and refined with fixed isotropic displacement parameters (in terms of a riding model).

These data can be obtained free of charge from The Cambridge Crystallographic Data Centre, 12 Union Road, Cambridge CB2 1EZ, UK (fax: +44(1223)-336-033; e-mail: deposit@ccdc.cam.ac.uk), or via https://www.ccdc.cam.ac.uk/getstructures.

[i] CrysAlisPro and ABSPACK. Rigaku Oxford Diffraction (2016).

4.1 L1 · HBF_4

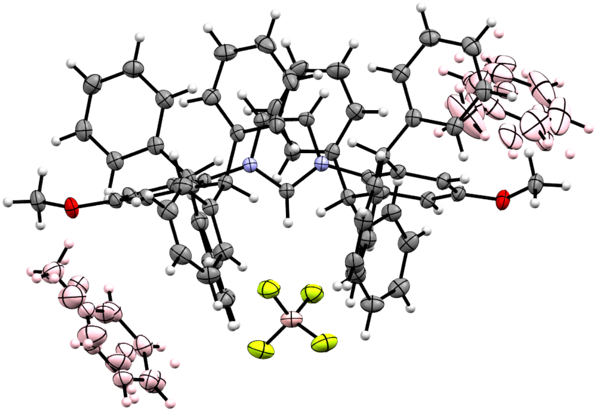

Figure S4: Asymmetric unit of compound IPr^{*OMe} · HBF_4. Ellipsoid are drawn at the 50% probability level. Disorder is shown in pink.

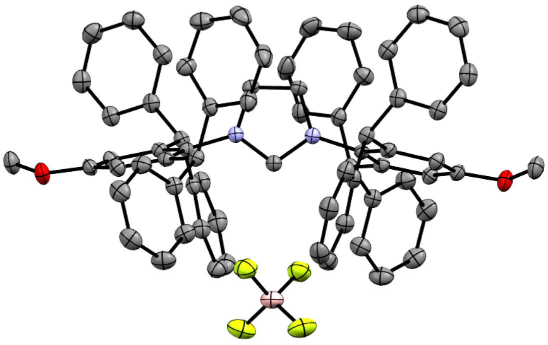

Figure S5: Crystallographic structure of compound IPr^{*OMe} · HBF_4. Hydrogens and lattice molecules (toluene) omitted for clarity. Ellipsoid are drawn at the 50% probability level. Disorder is shown in pink.

Experimental. Single crystals were isolated at 25 °C by slow evaporation of a concentrated toluene solution.

Note. There are four cocrystallized, disordered toluene molecules.

**Identification code**	CCDC **2250383**
Empirical formula	$C_{69}H_{57}BF_4N_2O_2$, 4 C_7H_8
Formula weight	1401.51
Temperature/K	100.0(1)
Crystal system	monoclinic
Space group	$I2/a$ (15)
a/Å	25.3296(2)
b/Å	9.7854(1)
c/Å	31.0012(2)
α/°	90
β/°	94.435(1)
γ/°	90
Volume/Å^3	7660.96(11)
Z	4
ρ_{calc}/g cm^{-3}	1.215
μ/mm^{-1}	0.614
F(000)	2968
Crystal size/mm^3	0.234 × 0.18 × 0.106
Crystal color	clear light yellow
Crystal shape	block
Radiation	Cu K$_\alpha$ (λ = 1.54178 Å)
2Θ range/°	5.72 to 160.37
Index ranges	-25 ≤ h ≤ 32
	-12 ≤ k ≤ 12
	-38 ≤ l ≤ 39
Reflections collected	47883
Independent reflections	8235
	R_{int} = 0.0327
	R_{sigma} = 0.0234
Data / Restraints / Param.	8235/642/611
Goodness-of-fit on F^2	1.058
Final R indexes	R_1 = 0.0399
[$I \geq 2\sigma(I)$]	wR_2 = 0.1049
Final R indexes	R_1 = 0.0440
[all data]	wR_2 = 0.1078
Largest peak/hole /e Å^3	0.51/-0.23

Note. A related structure of this ligand in the form of the aurate adduct ([**L1** · H][$AuCl_2$]) was reported by Nolan and co-workers (CCDC **1827215**).[16]

4.2 L1

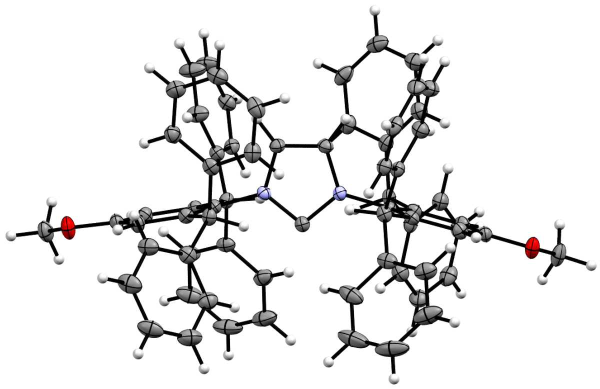

Figure S6: Asymmetric unit of compound IPr^{*OMe}. Ellipsoid are drawn at the 50% probability level.

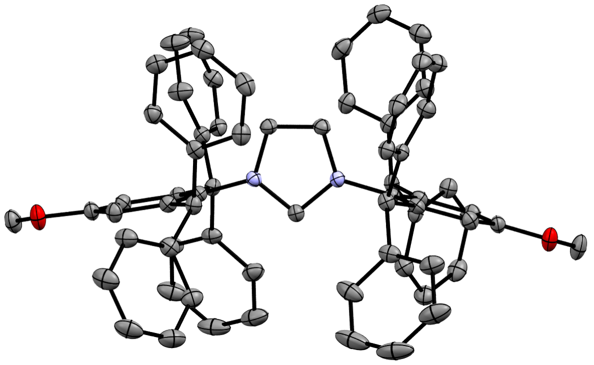

Figure S7: Crystallographic structure of compound IPr^{*OMe}. Hydrogens omitted for clarity.

Experimental. Single crystals were isolated at 25 °C by vapor diffusion of hexane into a concentrated toluene solution.

Note. About 5% of the carbene position is oxidized.

**Identification code**	CCDC **2250370**
Empirical formula	$C_{69}H_{56}N_2O_{2.04}$
Formula weight	945.79
Temperature/K	100.0(1)
Crystal system	triclinic
Space group	*P*-1 (2)
a/Å	12.4751(2)
b/Å	13.9259(2)
c/Å	17.1986(2)
α/°	70.621(1)
β/°	86.887(1)
γ/°	68.213(1)
Volume/Å^3	2609.15(7)
Z	2
ρ_{calc}/g cm^{-3}	1.204
μ/mm^{-1}	0.552
F(000)	1001
Crystal size/mm^3	0.20 × 0.145 × 0.11
Crystal color	clear colorless
Crystal shape	block
Radiation	Cu K$_\alpha$ (λ = 1.54178 Å)
2Θ range/°	7.26 to 159.63
Index ranges	-15 ≤ h ≤ 15
	-17 ≤ k ≤ 17
	-21 ≤ l ≤ 21
Reflections collected	89161
Independent reflections	11165
	R_{int} = 0.0486
	R_{sigma} = 0.0254
Data / Restraints / Param.	11165/18/670
Goodness-of-fit on F^2	1.053
Final R indexes	R_1 = 0.0373
[$I \geq 2\sigma(I)$]	wR_2 = 0.0933
Final R indexes	R_1 = 0.0409
[all data]	wR_2 = 0.0957
Largest peak/hole /e Å^3	0.26/-0.24

4.3 Complex 4

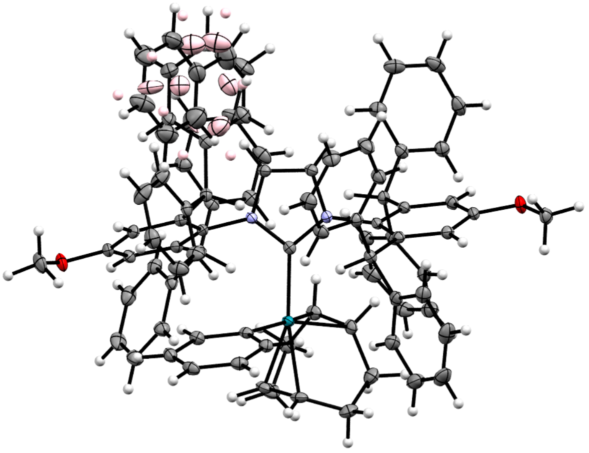

Figure S8: Asymmetric unit of compound **4**. Ellipsoid are drawn at the 50% probability level. Disorder is shown in pink.

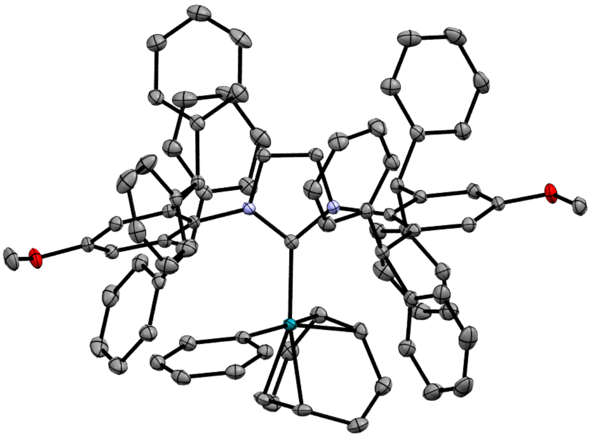

Figure S9: Crystallographic structure of compound **4**. Ellipsoid are drawn at the 50% probability level. Hydrogens and lattice molecules (toluene) omitted for clarity.

Experimental. Single crystals were isolated at 25 °C by slow diffusion of hexane into a saturated toluene solution.

Note. There is cocrystallized, disordered toluene.

**Identification code**	CCDC **2250373**
Empirical formula	$C_{83}H_{73}N_2O_2Rh$, C_7H_8
Formula weight	1325.47
Temperature/K	100.0(1)
Crystal system	monoclinic
Space group	$P2_1/n$ (14)
a/Å	10.83730(10)
b/Å	20.62350(10)
c/Å	30.2581(2)
α/°	90
β/°	90.3750(10)
γ/°	90
Volume/Å^3	6762.63(8)
Z	4
ρ_{calc}/g cm^{-3}	1.302
μ/mm^{-1}	2.449
F(000)	2784
Crystal size/mm^3	$0.18 \times 0.104 \times 0.073$
Crystal color	clear orange
Crystal shape	block
Radiation	Cu K$_\alpha$ (λ = 1.54178 Å)
2Θ range/°	5.19 to 144.25
Index ranges	$-13 \leq h \leq 13$
	$-25 \leq k \leq 25$
	$-37 \leq l \leq 37$
Reflections collected	179203
Independent reflections	13332
	R_{int} = 0.0519
	R_{sigma} = 0.0203
Data / Restraints / Param.	13332/163/924
Goodness-of-fit on F^2	1.054
Final R indexes	R_1 = 0.0247
[$I \geq 2\sigma(I)$]	wR_2 = 0.0624
Final R indexes	R_1 = 0.0262
[all data]	wR_2 = 0.0632
Largest peak/hole /e Å^3	0.53/-0.50

4.4 Complex S1

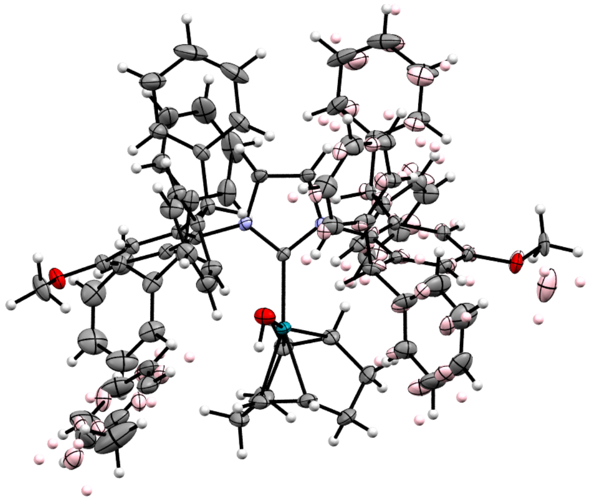

Figure S10: Asymmetric unit of compound **S1**. Ellipsoid are drawn at the 50% probability level. Disorder and minor species are shown in pink.

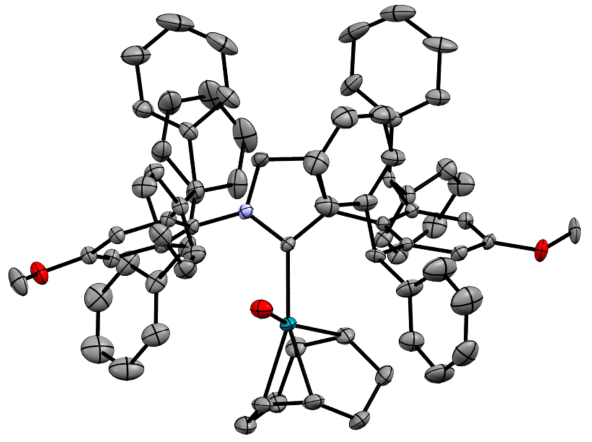

Figure S11: Crystallographic structure of the major species, **S1**. Ellipsoid are drawn at the 50% probability level. The minor species, hydrogens and lattice molecules (toluene) omitted for clarity.

**Identification code**	CCDC **2250372**
Empirical formula	$C_{77}H_{69}N_2O_3Rh$, C_7H_8
Formula weight	1265.38
Temperature/K	100.0(1)
Crystal system	orthorhombic
Space group	$P2_12_12_1$ (19)
a/Å	14.4063(3)
b/Å	21.0555(4)
c/Å	21.3613(4)
α/°	90
β/°	90
γ/°	90
Volume/Å^3	6479.6(2)
Z	4
ρ_{calc}/g cm^{-3}	1.297
μ/mm^{-1}	0.317
F(000)	2656
Crystal size/mm^3	0.235 × 0.126 × 0.035
Crystal color	clear light yellow
Crystal shape	plate
Radiation	Mo K$_\alpha$ (λ = 0.71073 Å)
2Θ range/°	4.79 to 69.60
Index ranges	-22 ≤ h ≤ 23
	-32 ≤ k ≤ 23
	-34 ≤ l ≤ 32
Reflections collected	108178
Independent reflections	24459
	R_{int} = 0.0456
	R_{sigma} = 0.0440
Data / Restraints / Param.	24459/3505/1183
Goodness-of-fit on F^2	1.026
Final R indexes	R_1 = 0.0428
$[I \geq 2\sigma(I)]$	wR_2 = 0.0952
Final R indexes	R_1 = 0.0535
[all data]	wR_2 = 0.0993
Largest peak/hole /e Å^3	3.07/-1.39
Flack x parameter	-0.019(6)

Experimental. Single crystals were isolated at 25 °C by slow diffusion of hexane into a saturated toluene solution.

Notes. There is disorder in half of the ligand and co-crystallized, disordered toluene. Also, there is a second species in 4% occupation that was not modeled. There is a remaining electron-density $3e^-/\text{Å}^3$ next to Rh; the exact nature could not be determined.

5 Influence of the Base

Table S2: Catalytic competence of Rh complexes in the absence of base.

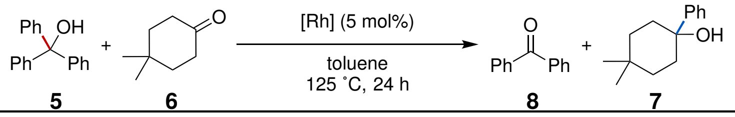

Entry[a]	Complex	Yield **8** (%)[b]	Yield **7** (%)[b]
1	[Rh(IPr^{*OMe})(COD)Cl] (**1**)	0	0
2	[Rh(IPr^{*OMe})(COD)(OH)] (**S1**)	13	13
3	[Rh(IPr^{*OMe})(COD)]BF_4 (**3**)	32	26
4	[Rh(IPr^{*OMe})(Ph)(COD)] (**4**)	64	73

[a] General conditions: **5** (0.150 M), **6** (0.100 M), [Rh] (0.005 M), *n*-dodecane (0.097 M), 125 °C, 24 h.
[b] Product yield was determined by GC analysis.

Table S3: Using alkoxides instead of alcohols.

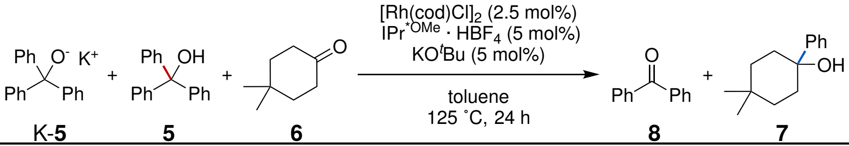

Entry[a]	**5** (equiv.)	K-**5** (equiv.)	Yield **8** (%[b])	Yield **7** (%)[b]
1	1.5	0	149	86
2	0	1.5	8	5
3	0.3	1.2	11	5
4	1.3	0.2	38	13

[a] General conditions: **6** (0.100 M), **1** (0.005 M), *n*-dodecane (0.097 M), 125 °C, 24 h.
[b] Product yield was determined by GC analysis.

Table S4: Assessment of impact of moisture in inorganic base.

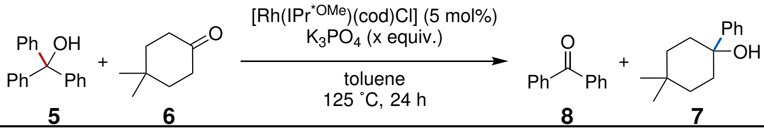

Entry[a]	Base	equiv.	Yield **8** (%[b])	Yield **7** (%)[b]
1	K_3PO_4 (regular)	1.0	119	93
2	K_3PO_4 (vacuum dried)	1.0	112	93
3	K_3PO_4 (regular)	0.1	14	16
4	K_3PO_4 (vacuum dried)	0.1	50	57

[a] General conditions: **5** (0.150 M), **6** (0.100 M), K_3PO_4 (0.010 M), **1** (0.005 M), *n*-dodecane (0.097 M), 125 °C, 24 h.
[b] Product yield was determined by GC analysis.

Table S5: Catalytic competence of Rh complexes with reduced amount of base.

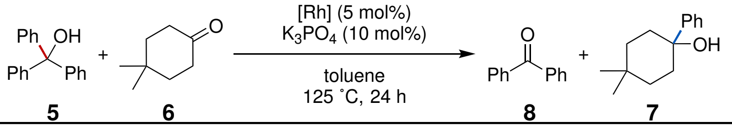

Entry[a]	Complex	Yield **8** (%)[b]	Yield **7** (%)[b]
1	[Rh(IPr^{*OMe})(COD)Cl] (**1**)	50	57
2	[Rh(IPr^{*OMe})(COD)]BF_4 (**3**)	54	52
3	[Rh(IPr^{*OMe})(Ph)(COD)] (**4**)	106	91

[a] General conditions: **5** (0.150 M), **6** (0.100 M), K_3PO_4 (0.010 M), [Rh] (0.005 M), *n*-dodecane (0.097 M), 125 °C, 24 h.
[b] Product yield was determined by GC analysis.

6 Kinetic Measurements and Analysis

6.1 Catalytic Competence of Complexes

General Procedure. A 4 mL-vial under air was charged with **5** (39.1 mg, 0.150 mmol, 1.5 equiv.), **6** (12.6 mg, 0.100 mmol, 1.0 equiv.) and a magnetic stirring bar, and the vial was transferred into a glovebox. In a glovebox, the vial was charged with K_3PO_4 (21.2 mg, 0.100 mmol, 1.0 equiv.), rhodium complex (5.0 µmol, 5 mol %), *n*-dodecane (22.0 µL, 0.0970 mmol) as internal standard and toluene (0.50 mL).

The vial was sealed with a screw cap, taken out of a glovebox and placed in a preheated heating block at 125 °C for 24 h. After cooling to room temperature again, an aliquot was removed, diluted with EtOAc (approx. 0.5 mL) and filtered over a short plug of celite, followed by GC analysis. The reaction was then continued with the same vials under the assumption that no conversion took place at ambient temperature.

Table S6: Catalytic competence of Rh complexes.

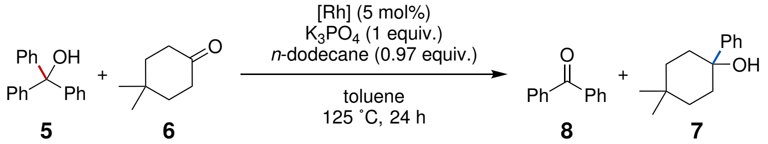

Entry[a]	Complex	Yield **8** (%)[b]	Yield **7** (%)[b]	Catal. competent?
	$[Rh(COD)Cl]_2$, IPr^{*OMe} · HBF_4, KO^{t}Bu	99	86	
1	[Rh(IPr^{*OMe})(COD)Cl] (**1**)	75	81	Yes
2	[Rh(IPr^{*OMe})(COD)(OH)] (**S1**)	69	77	Yes
3	[Rh(IPr^{*OMe})(COD)]BF_4 (**3**)	117	92	Yes
4	[Rh(IPr^{*OMe})(Ph)(COD)] (**4**)	83	80	Yes

[a] General conditions: **5** (0.150 M), **6** (0.100 M), K_3PO_4 (0.100 M), [Rh] (0.005 M), *n*-dodecane (0.097 M), 125 °C, 24 h.
[b] Product yield was determined by GC analysis.

6.2 Kinetic Competence of Complexes

General Procedure. The experiments were set up according to the general procedure in Section 6.1. At regular time intervals, the reaction was stopped by cooling to 0 °C in an ice-water bath and the vials were transferred into a glovebox. An aliquot of 30.0 µL was removed, diluted with EtOAc (approx. 0.5 mL) and filtered over a short plug of

celite, followed by GC analysis. The reaction was then continued with the same vials under the assumption that no conversion took place at ambient temperature.

Table S7: Kinetic competence of Rh complexes.

Entry[a]	Complex	rate [alcohol (**7**)] ($M\,s^{-1}$)[b]	rate [ketone (**8**)] ($M\,s^{-1}$)[b]
1[c]	[Rh(COD)Cl]$_2$, IPr^{*OMe}	1.60(17) × 10^{-6}	1.34(14) × 10^{-6}
2[c]	[Rh(IPr^{*OMe})(COD)Cl] (**1**)	2.97(8) × 10^{-6}	2.58(7) × 10^{-6}
3[c]	[Rh(IPr^{*OMe})(COD)]BF$_4$ (**3**)	5.04(14) × 10^{-6}	4.49(11) × 10^{-6}
4[d]	[Rh(IPr^{*OMe})(Ph)(COD)] (**4**)	6.22(12) × 10^{-6}	5.67(13) × 10^{-6}

[a] General conditions: **5** (0.300 M), **6** (0.200 M), K_3PO_4 (0.200 M), [Rh] (0.010 M), *n*-dodecane (0.097 M).
[b] Estimate from linear regression with standard error of the mean.
[c] Average of three independent measurements.
[d] Average of two independent measurements.

6.3 Explanation for The Higher Rate of Formation of Ketone Product Versus Alcohol Product

The rate of formation for the ketone product **8** was slightly higher than that of the alcohol product **7**, independently of the employed precatalyst. This result is consistent with the generally higher observed yields of **8** and the formation of benzene in the catalytic reaction.[8]

This observation supports the hypothesis that the catalyst can turn over through an unproductive pathway after β-carbon elimination. Instead of undergoing ketone insertion, protodemetalation by proton transfer from an alcohol (substrate or product) releases benzene and regenerates the putative alkoxide complex, thus regenerating the catalyst prematurely (Scheme S1).

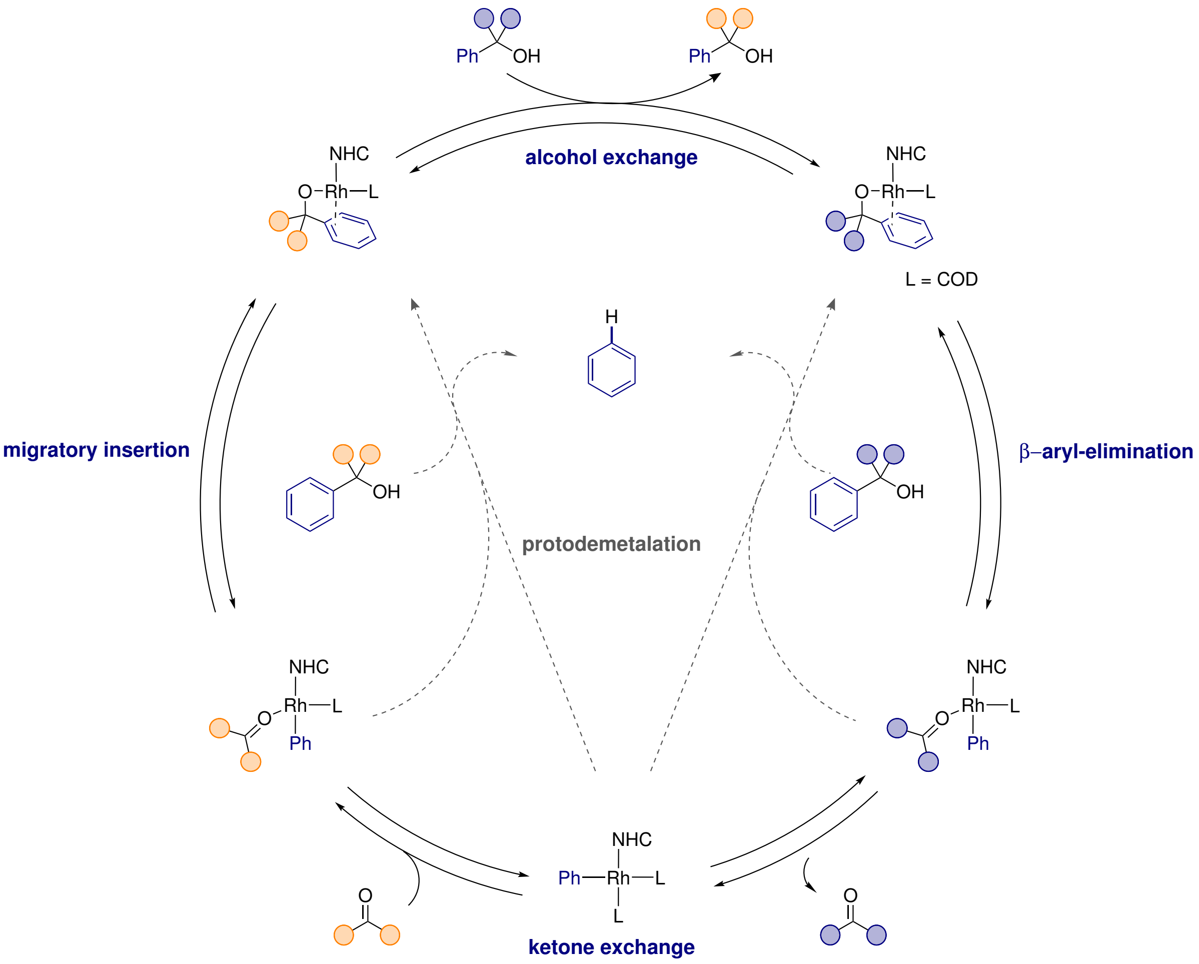

Scheme S1: Rational for protodemetalation side reaction.

6.4 RPKA Kinetics

6.4.1 Catalyst Deactivation (Same Excess) and Product Inhibition (Different Excess)

Catalyst deactivation and product inhibition was assessed using the RPKA methodology, as reported by Blackmond et al.[17,18] The "same excess" experiment simulates the reaction, where the Rh catalyst has already completed six turnovers (30% conversion). The three concentration profiles of the "same excess" and "product inhibition" experiments were shifted by 12600 s (210 min) to take into account the time required to complete six turnovers. In the two "product inhibition" experiments, additionally an equivalent amount of either product compared to "consumed" starting material was added.

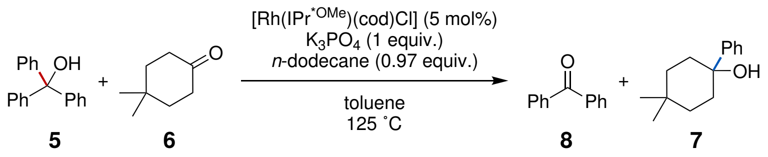

General Procedure. 4 mL-vials under air were charged with **5** (78.1 mg, 0.300 mmol, 1.5 equiv.) and a magnetic stirring bar and the vials were transferred into a glovebox. A 0.025 M stock solution of **1** in toluene was prepared inside a glovebox. Likewise, a 1.0 M stock solution of **6** in toluene was prepared. The substrate-containing vial was charged with K_3PO_4 (42.5 mg, 0.200 mmol), 1.0 equiv.), **6** stock solution (200.0 µL, 0.200 mmol), 1.0 equiv.), *n*-dodecane (22.0 µL, 0.0970 mmol) as internal standard, toluene (400.0 µL), and the catalyst stock solution (400.0 µL, 5 µmol, 5 mol %) (total volume: 1.00 mL). The vials were sealed with a screw cap, taken out of a glovebox and placed in a preheated heating block at 125 °C. At regular time intervals, the reaction was stopped by cooling to 0 °C in an ice-water bath and the vials were transferred into a glovebox. An aliquot of 30.0 µL was removed, diluted with EtOAc (approx. 0.5 mL) and filtered over a short plug of celite, followed by GC analysis. The reaction was then continued with the same vials under the assumption that no conversion took place at ambient temperature.

Data Analysis. Data analysis and plotting was done with *RStudio*. The concentration data were determined by GC measurements with an internal standard using an external calibration curve. The average of three independent measurements was calculated for each time point and plotted against the reaction time. The error bars represent the standard deviation of the mean (standard error).

Entry	Experiment type	[Rh] (**1**) (M)	[a. sm (**5**)] (M)	[k. sm (**6**)] (M)	[a. product (**7**)] (M)	[k. product (**8**)] (M)
1	Standard	0.010	0.300	0.200	0	0
2	Same excess	0.010	0.240	0.140	0	0
3	Product inhibition	0.010	0.240	0.140	0.060	0
4	Product inhibition	0.010	0.240	0.140	0	0.060

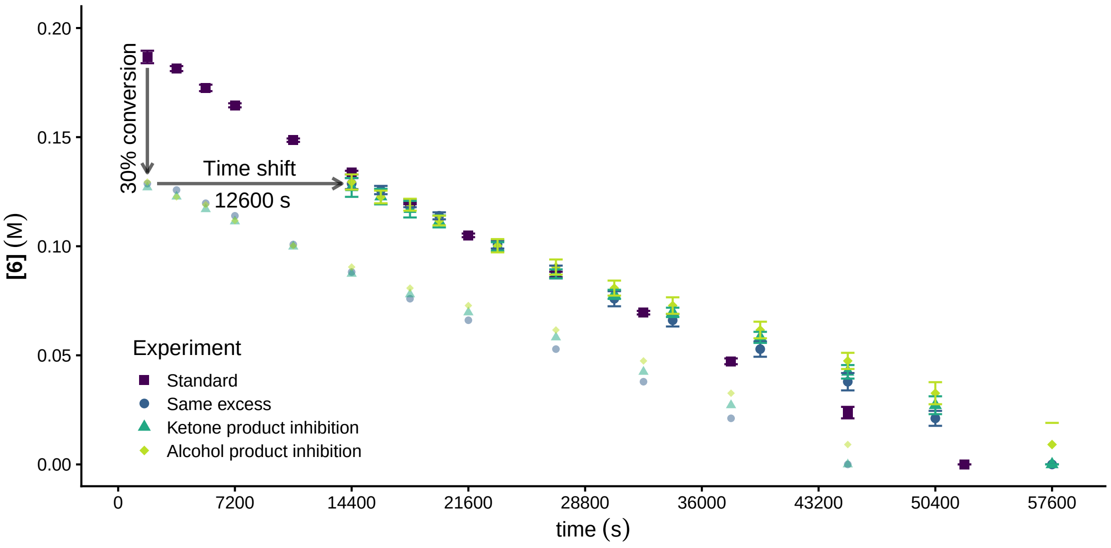

Figure S12: Overlay of the concentration profiles of the ketone starting material (**6**) to study catalyst deactivation and product inhibition. Black squares: standard reaction, blue circles: "same excess", dark green triangles: ketone product (**8**) inhibition, light green diamonds: alcohol product (**7**) inhibition. Each data point is the average of three experiments with standard deviation of the mean included as error bars.

Initially the curves overlap well, showing that there is no significant catalyst deactivation taking place. At high conversions (approx. 32400 s (540 min)) the standard reaction shows a higher consumption of starting material than the "same excess" experiment. Further, there is no significant product inhibition compared to the "same excess" experiment.

Since we observe deviation of the concentration profiles after 32400 s, the analysis for the VTNA method was restricted to the first 6 hours (21600 s).

6.5 VTNA Kinetics

General Procedure. The experiments were conducted according to the general procedure in Section 6.4.1.

Data Analysis. The analysis was conducted with *RStudio* according to the VTNA method described by Burés et al.[19] The concentration data were determined by GC measurements with an internal standard using an external calibration curve. The average of three independent measurements was calculated for each time point and plotted against the reaction time. The error bars represent the standard deviation of the mean (standard error).

6.5.1 Order in complex 1

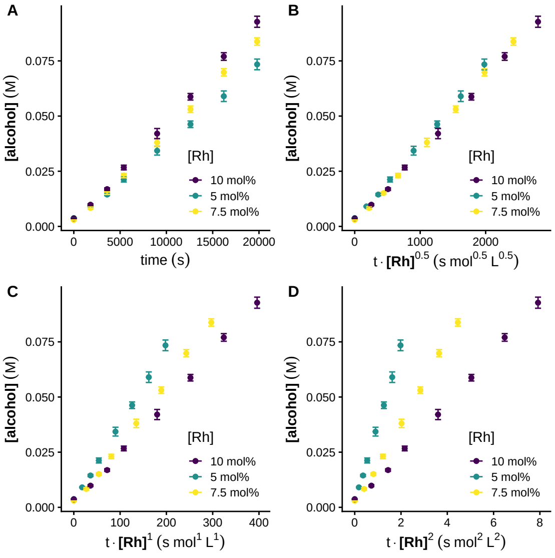

(a) Alcohol product (**7**)

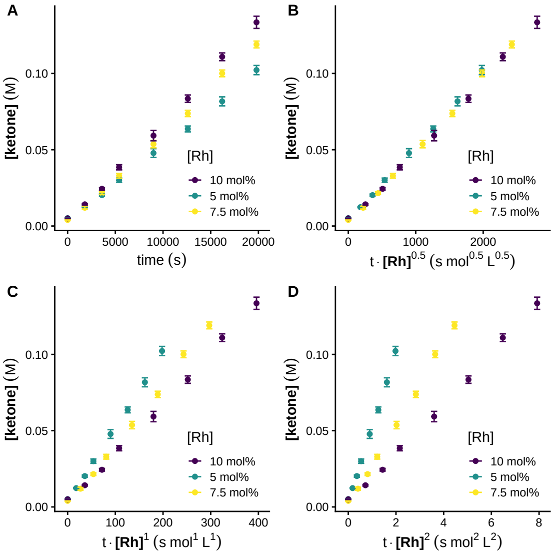

(b) Ketone product (**8**)

Figure S13: Plot of the concentration profiles of **7** and **8** in dependence of the concentration of the catalyst (**1**). The timescale was normalized according to the VTNA method. Each data point is the average of three experiments with standard deviation of the mean included as error bars. A) $n = 0$, B) $n = 0.5$, C) $n = 1$, D) $n = 2$. Graphical analysis determined that the curves overlap best for $n = 0.5$.

6.5.2 Order in alcohol 5

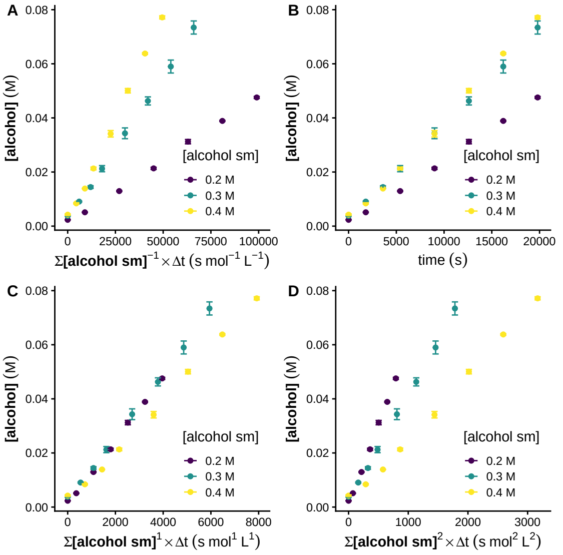

(a) Alcohol product (**7**)

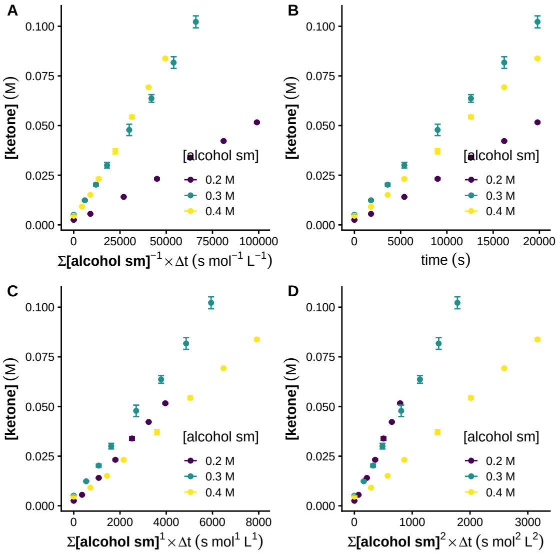

(b) Ketone product (**8**)

Figure S14: Plot of the concentration profiles of **7** and **8** in dependence of the concentration of alcohol substrate (**5**). Each data point is the average of three experiments with standard deviation of the mean included as error bars. The time scale was normalized according to the VTNA method. A) $n = -1$, B) $n = 0$, C) $n = 1$, D) $n = 2$. Graphical analysis determined that the curves overlap best for $n = 1$.

6.5.3 Order in ketone 6

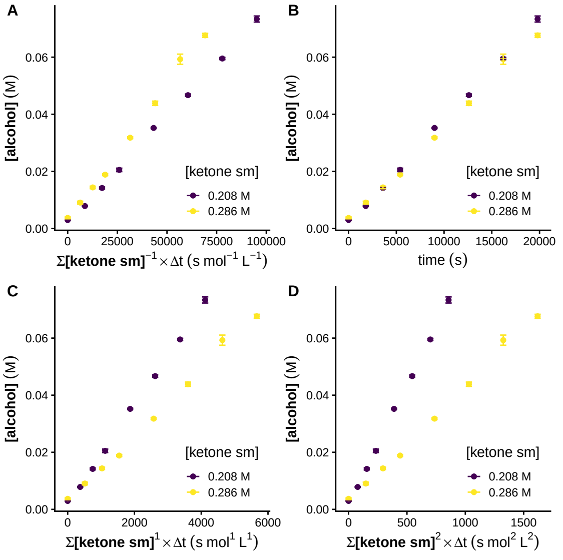

(a) Alcohol product (**7**)

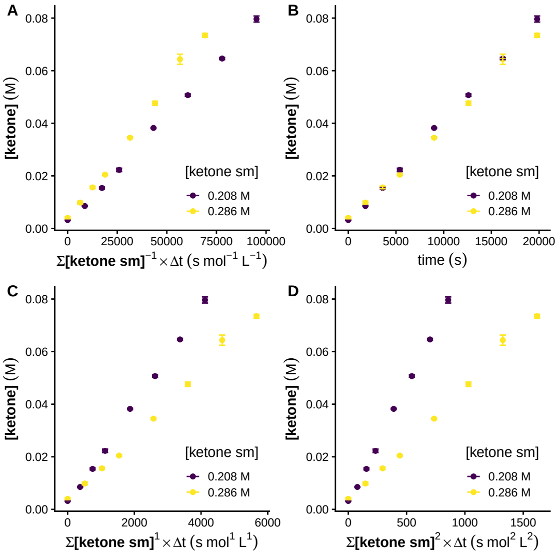

(b) Ketone product (**8**)

Figure S15: Plot of the concentration profiles of **7** and **8** in dependence of the concentration of ketone substrate (**6**). Each data point is the average of three experiments with standard deviation of the mean included as error bars. The time scale was normalized according to the VTNA method. A) $n = -1$, B) $n = 0$, C) $n = 1$, D) $n = 2$. Graphical analysis determined that the curves overlap best for $n = 0$.

6.5.4 Order in base

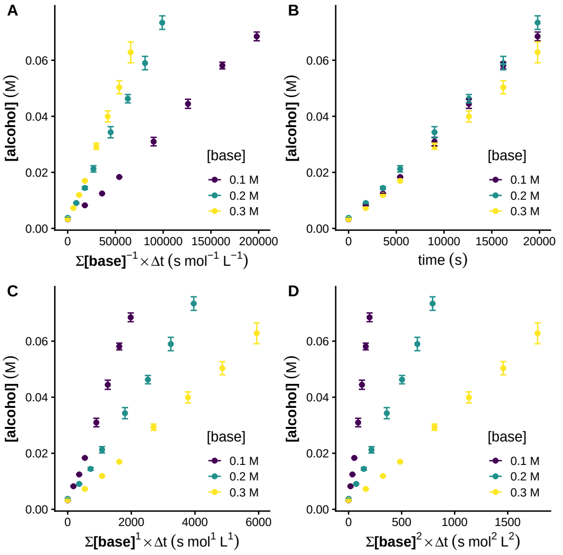

(a) Alcohol product (**7**)

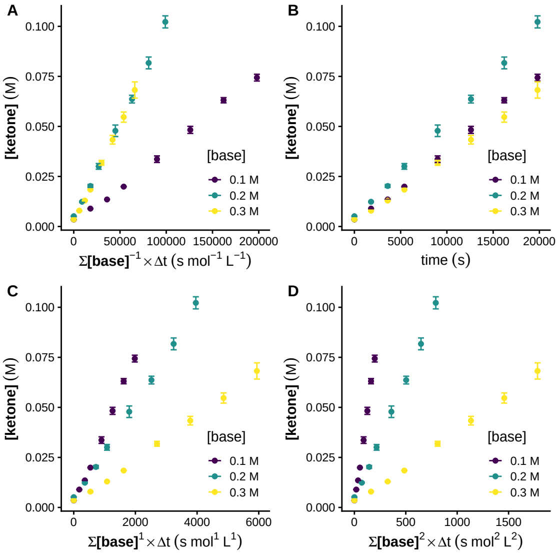

(b) Ketone product (**8**)

Figure S16: Plot of the concentration profiles of **7** and **8** in dependence of the concentration of K_3PO_4. Each data point is the average of three experiments with standard deviation of the mean included as error bars. The time scale was normalized according to the VTNA method. A) $n = -1$, B) $n = 0$, C) $n = 0.5$, D) $n = 1$. Each data point is the average of three experiments with standard deviation of the mean included as error bars. Graphical analysis determined that the curves overlap best for $n = 0$.

6.5.5 Observed Rate Constant

When plotting the concentration profiles against a VTNA time scale where the order of all reagents (**1**, **5** and **6**) is raised to their correct order, a straight line is obtained with its slope corresponding to the rate constant k_{obs}.[19]

Table S8: Observed rate constant (k_{obs}).

k_{obs} [alcohol (**7**)] (M $s^{-1.5}$)[a]	k_{obs} [ketone (**8**)] (M $s^{-1.5}$)[a]
1.21(2) × 10^{-4}	1.59(6) × 10^{-4}

[a] Estimate from linear regression with standard error of the mean.

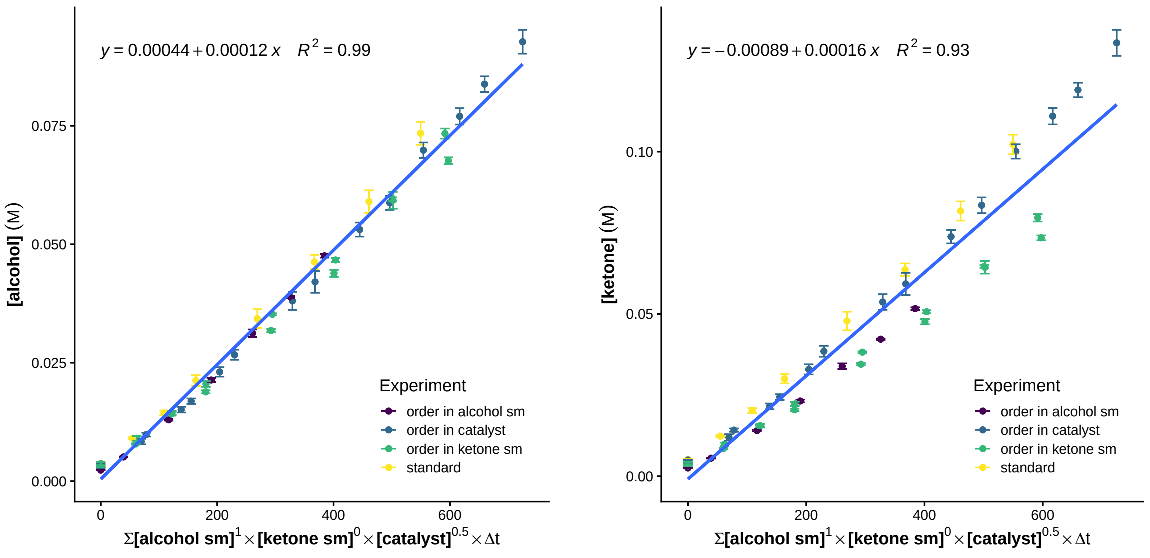

(a) Alcohol product (**7**) **(b)** Ketone product (**8**)

Figure S17: Plot of the concentration profiles of **7** and **8** in dependence of the concentrations of the catalyst (**1**), alcohol substrate (**5**), and ketone substrate (**6**). The time scale was normalized according to the VTNA method. The observed rate constant was extracted by linear regression of the linearized curve. Each data point is the average of three experiments with standard deviation of the mean included as error bars.

6.6 Initial Rates Kinetics

General Procedure. The experiments were conducted according to the general procedure in Section 6.4.1.

Data Analysis. Data analysis and plotting was done with *RStudio*. The concentration data were determined by GC measurements with an internal standard using an external calibration curve. The initial reaction rate was determined by plotting the concentrations of both products **7** and **8** over time, limiting the data to 10% conversion. This process was repeated in duplicate for each experiment in order to arrive at an average value of $\frac{d[\mathbf{7}]}{dt}$ and $\frac{d[\mathbf{8}]}{dt}$. The initial rates were then plotted against the concentration (x) of reactant in question and the data was fitted with an exponential equation of the form $y = a \cdot x^b$. The same data was then linearized using the found exponent b. As no induction period was observed for our standard reaction (see Fig. S12), the steady state approximation in the linear regime of the reaction is assumed.

The same data, except without limiting to 10% conversion, was also analyzed by the VTNA method.[19]

6.6.1 Order in complex 1

Table S9: Initial rates.

Entry[a]	[**1**] (M)	rate [alcohol (**7**)] (M s^{-1})[b]	rate [ketone (**8**)] (M s^{-1})[b]
1	0.0025	1.62(7) × 10^{-6}	2.10(10) × 10^{-6}
2	0.0050	2.27(10) × 10^{-6}	3.02(14) × 10^{-6}
3	0.010	2.89(12) × 10^{-6}	3.70(19) × 10^{-6}
4	0.020	4.58(22) × 10^{-6}	5.69(32) × 10^{-6}

[a] General conditions: **5** (0.300 M), **6** (0.200 M), K_3PO_4 (0.200 M), *n*-dodecane (0.097 M).
[b] Standard errors of the mean are reported in parentheses.

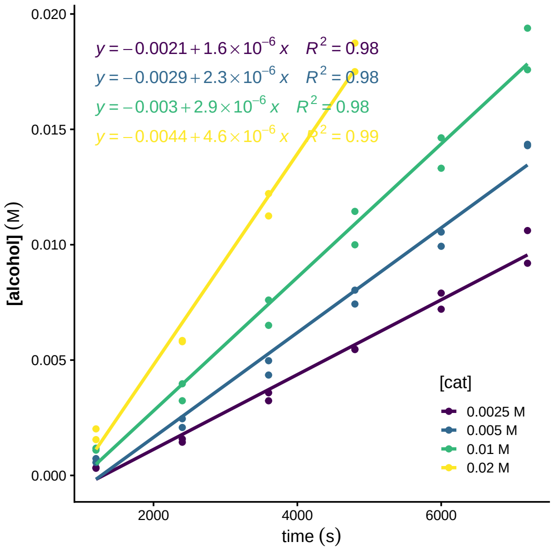

(a) Alcohol product (**7**)

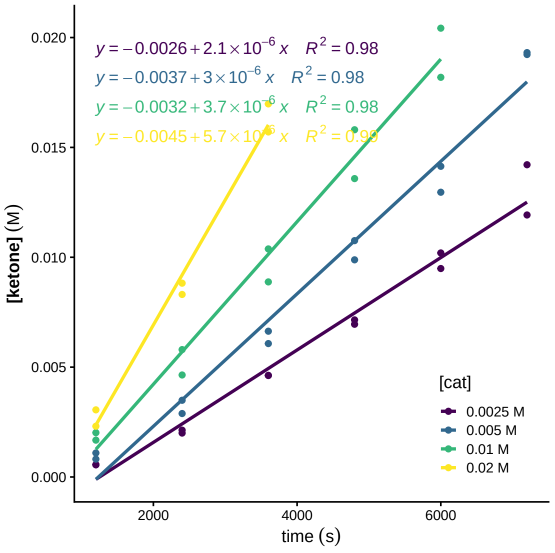

(b) Ketone product (**8**)

Figure S18: Plot of the concentration profiles of **7** and **8** in dependence of the concentration of the catalyst (**1**). Each reaction was run in duplicate in parallel.

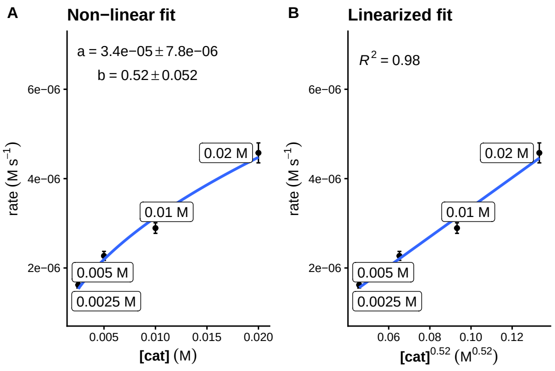

(a) Alcohol product (**7**)

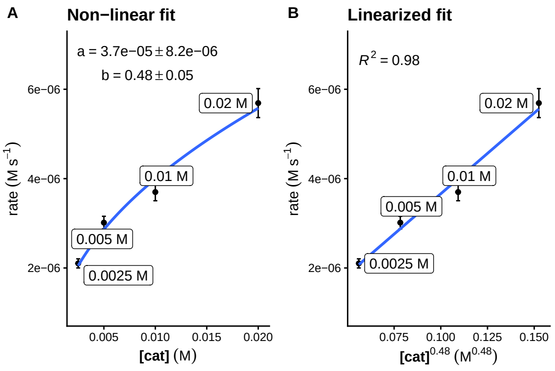

(b) Ketone product (**8**)

Figure S19: Plot of the initial reaction rate in dependence of the concentration of the catalyst (**1**). Each data point is the average of two experiments with standard deviation of the mean included as error bars. A) Non-linear exponential fit to determine order; B) Linearized fit with obtained order.

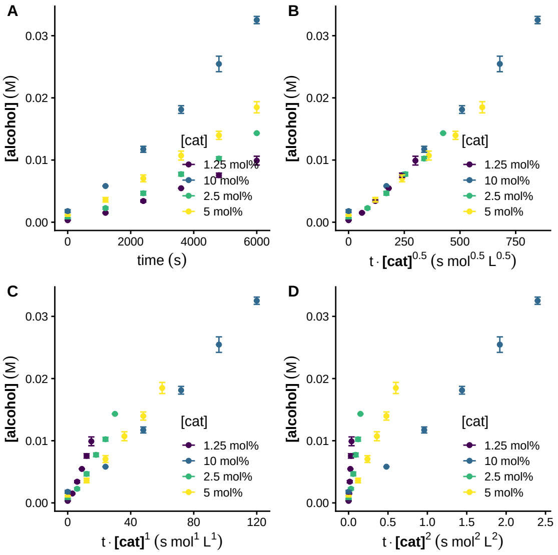

(a) Alcohol product (**7**)

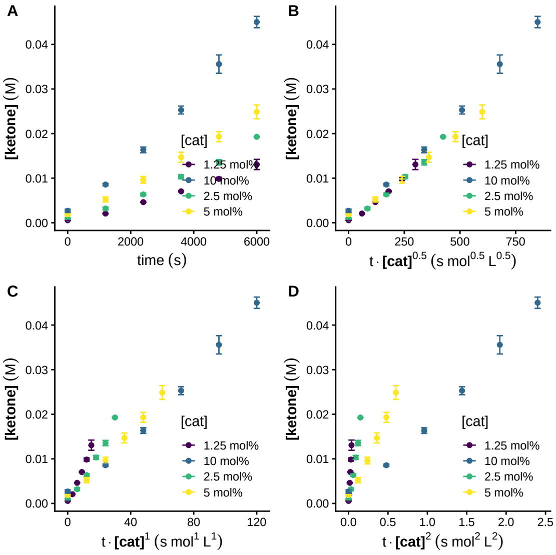

(b) Ketone product (**8**)

Figure S20: Plot of the concentration profiles of **7** and **8** in dependence of the concentration of the catalyst (**1**). The time scale was normalized according to the VTNA method. Each data point is the average of two experiments with standard deviation of the mean included as error bars. A) $n = 0$, B) $n = 0.5$, C) $n = 1$, D) $n = 2$. Graphical analysis determined that the curves overlap best for $n = 0.5$.

6.6.2 Order in phenyl complex 4

Note. The first 15% conversion were analyzed for this experiment to have enough data points for linear regression of the 0.010 M concentration data.

Table S10: Initial rates.

Entry[a]	[**4**] (M)	rate [alcohol (**7**)] (M s^{-1})[b]	rate [ketone (**8**)] (M s^{-1})[b]
1	0.0025	1.97(3) × 10^{-6}	2.96(6) × 10^{-6}
2	0.0050	2.96(13) × 10^{-6}	3.91(12) × 10^{-6}
3	0.010	5.20(23) × 10^{-6}	6.16(55) × 10^{-6}

[a] General conditions: **5** (0.300 M), **6** (0.200 M), K_3PO_4 (0.200 M), *n*-dodecane (0.097 M).
[b] Standard errors of the mean are reported in parentheses.

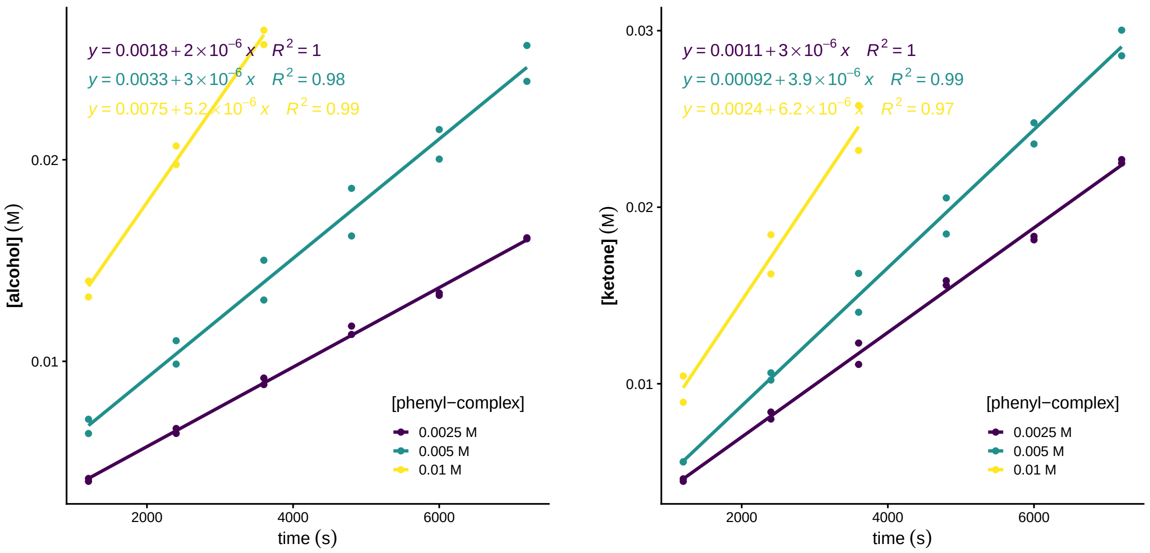

(a) Alcohol product (**7**) **(b)** Ketone product (**8**)

Figure S21: Plot of the concentration profiles of **7** and **8** in dependence of the concentration of the catalyst (**4**). Each reaction was run in duplicate in parallel.

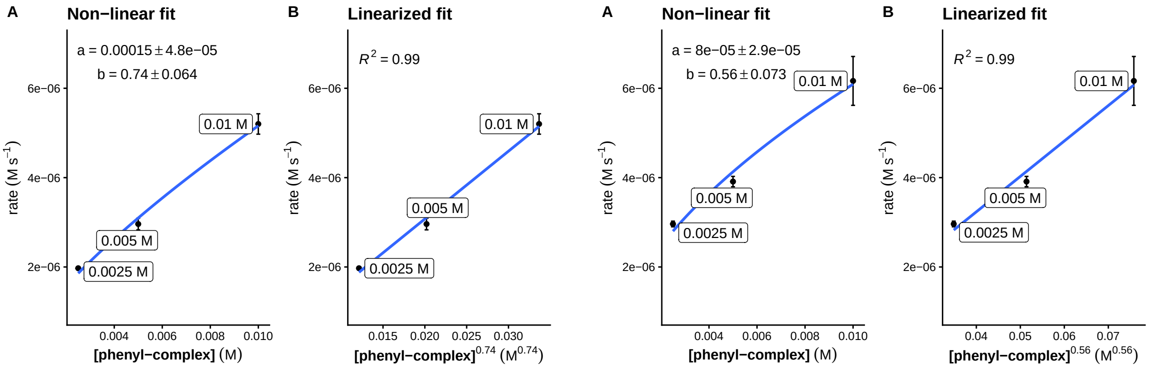

(a) Alcohol product (**7**) **(b)** Ketone product (**8**)

Figure S22: Plot of the initial reaction rate in dependence of the concentration of the catalyst (**4**). Each data point is the average of two experiments with standard deviation of the mean included as error bars. A) Non-linear exponential fit to determine order; B) Linearized fit with obtained order.

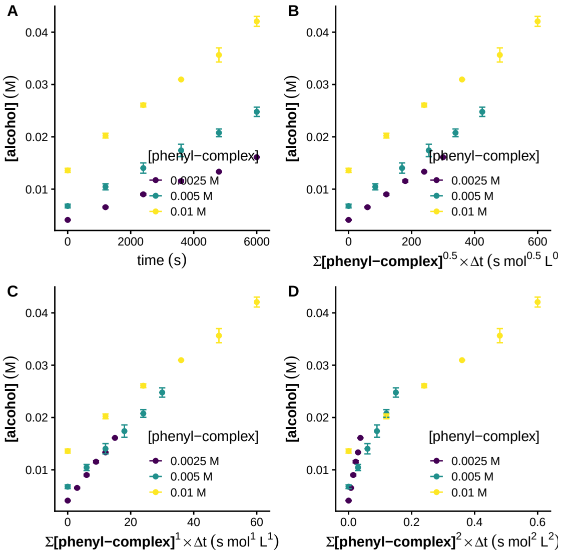

(a) Alcohol product (**7**)

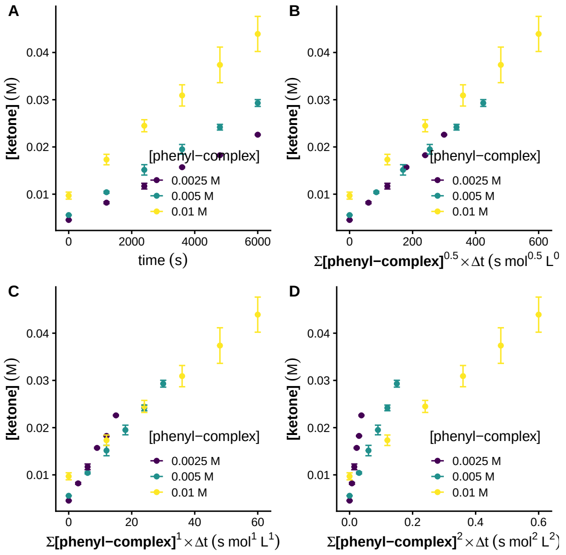

(b) Ketone product (**8**)

Figure S23: Plot of the concentration profiles of **7** and **8** in dependence of the concentration of the catalyst (**4**). Each data point is the average of three experiments with standard deviation of the mean included as error bars. The time scale was normalized according to the VTNA method. A) n = 0, B) n = 0.5, C) n = 1, D) n = 2. Graphical analysis determined that the curves overlap best for n = 0.5.

6.6.3 Order in alcohol 5

Table S11: Initial rates.

Entry[a]	[**5**] (M)	rate [alcohol (**7**)] (M s^{-1})[b]	rate [ketone (**8**)] (M s^{-1})[b]
1	0.300	2.89(12) × 10^{-6}	3.70(19) × 10^{-6}
2	0.400	3.81(9) × 10^{-6}	5.06(23) × 10^{-6}
3	0.500	4.35(15) × 10^{-6}	6.51(41) × 10^{-6}

[a] General conditions: **6** (0.200 M), **1** (0.010 M), K_3PO_4 (0.200 M), *n*-dodecane (0.097 M).
[b] Standard errors of the mean are reported in parentheses.

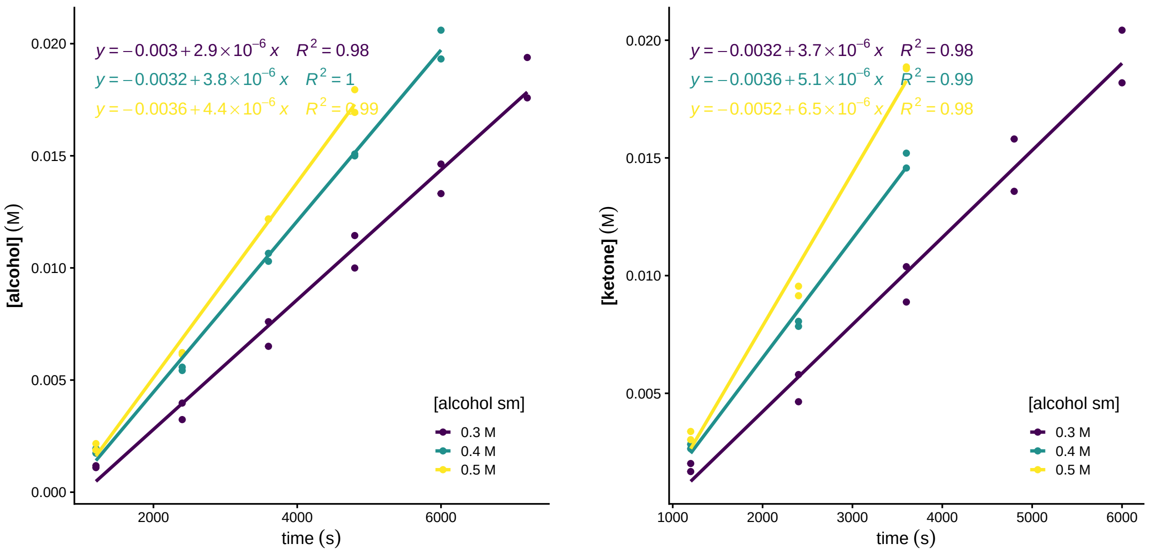

(a) Alcohol product (**7**) **(b)** Ketone product (**8**)

Figure S24: Plot of the concentration profiles of **7** and **8** in dependence of the concentration of the alcohol substrate (**5**). Each reaction was run in duplicate in parallel.

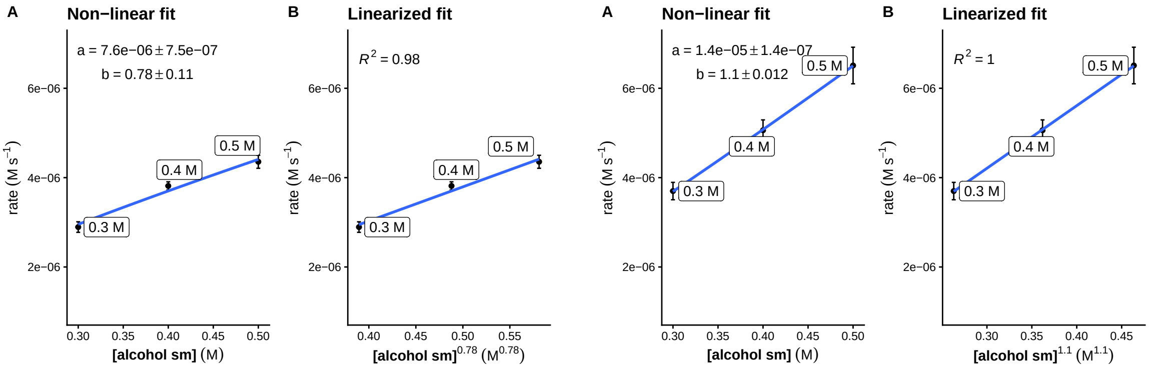

(a) Alcohol product (**7**) **(b)** Ketone product (**8**)

Figure S25: Plot of the initial reaction rate in dependence of the concentration of the alcohol substrate (**5**). Each data point is the average of two experiments with standard deviation of the mean included as error bars. A) Non-linear exponential fit to determine order; B) Linearized fit with obtained order.

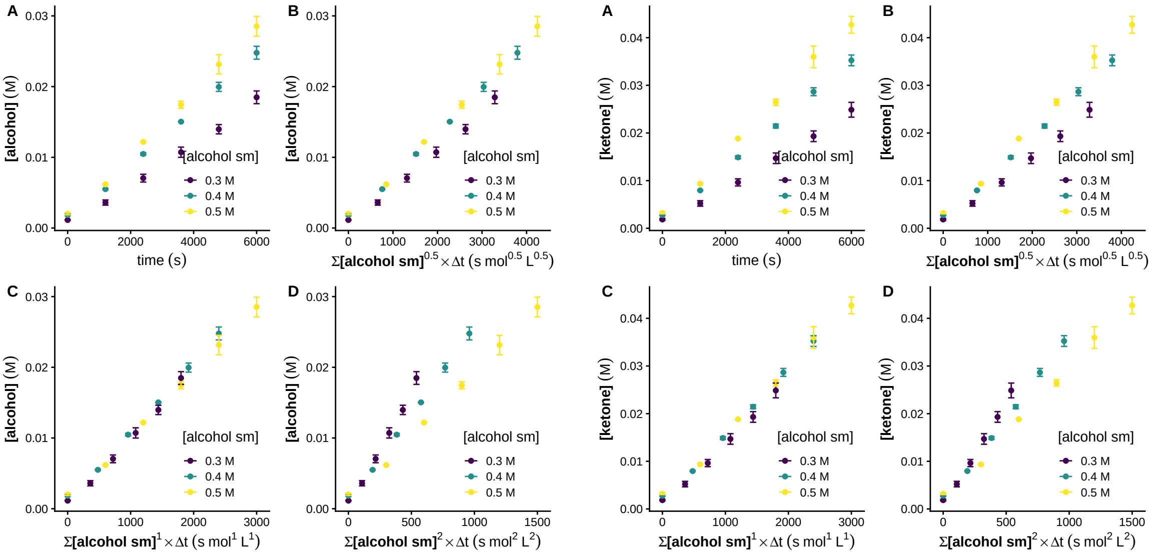

(a) Alcohol product (**7**) **(b)** Ketone product (**8**)

Figure S26: Plot of the concentration profiles of **7** and **8** in dependence of the concentration of the alcohol substrate (**5**). Each data point is the average of three experiments with standard deviation of the mean included as error bars. The time scale was normalized according to the VTNA method. A) $n = 0$, B) $n = 0.5$, C) $n = 1$, D) $n = 2$. Graphical analysis determined that the curves overlap best for $n = 1$.

6.6.4 Order in ketone 6

Table S12: Initial rates.

Entry[a]	[**6**] (M)	rate [alcohol (**7**)] (M s^{-1})[b]	rate [ketone (**8**)] (M s^{-1})[b]
1	0.200	3.47(16) × 10^{-6}	3.67(25) × 10^{-6}
2	0.320	3.62(17) × 10^{-6}	3.57(12) × 10^{-6}
3	0.440	4.02(33) × 10^{-6}	3.72(29) × 10^{-6}

[a] General conditions: **5** (0.300 M), **1** (0.010 M), K_3PO_4 (0.200 M), *n*-dodecane (0.097 M).
[b] Standard errors of the mean are reported in parentheses.

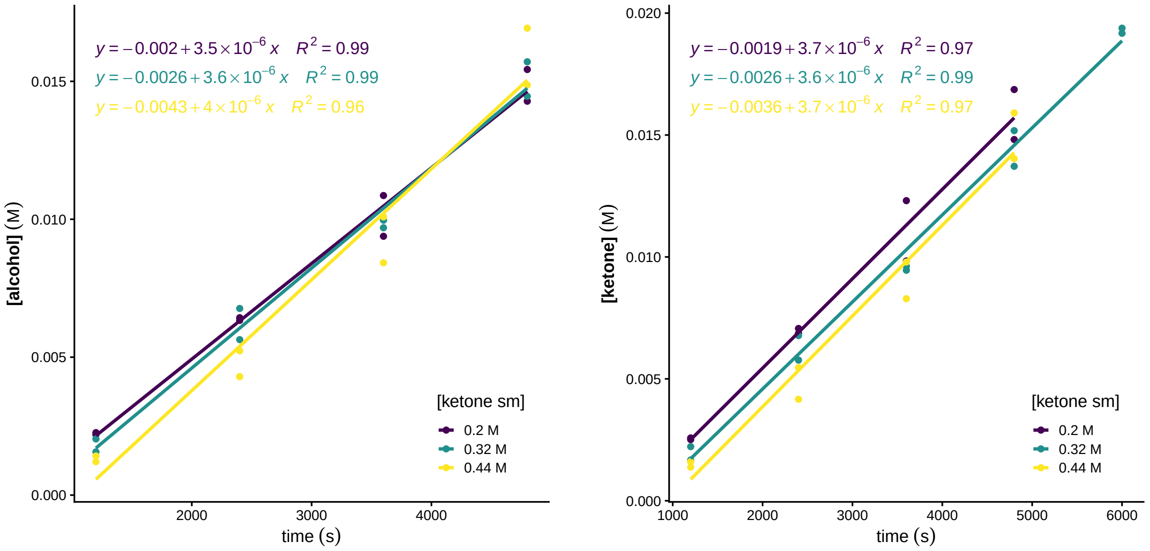

(a) Alcohol product (**7**) **(b)** Ketone product (**8**)

Figure S27: Plot of the concentration profiles of **7** and **8** in dependence of the concentration of the ketone substrate (**6**). Each reaction was run in duplicate in parallel.

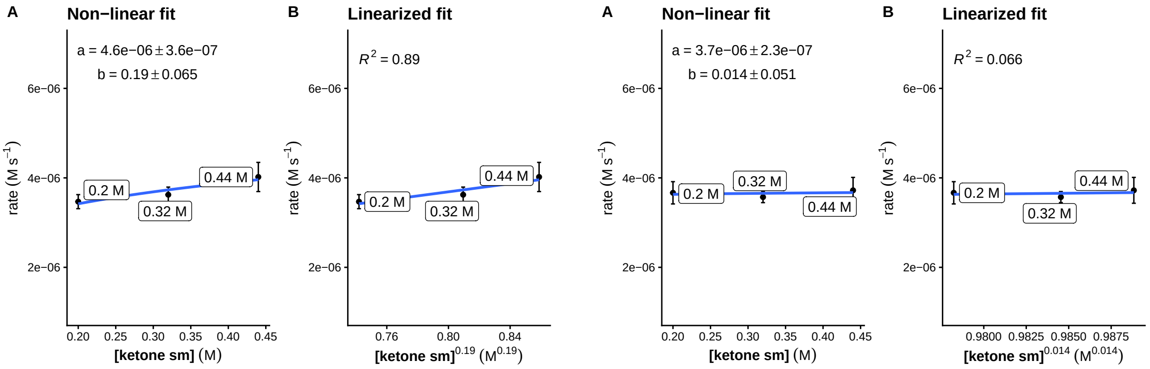

(a) Alcohol product (**7**) **(b)** Ketone product (**8**)

Figure S28: Plot of the initial reaction rate in dependence of the concentration of the ketone substrate (**6**). Each data point is the average of two experiments with standard deviation of the mean included as error bars. A) Non-linear exponential fit to determine order; B) Linearized fit with obtained order.

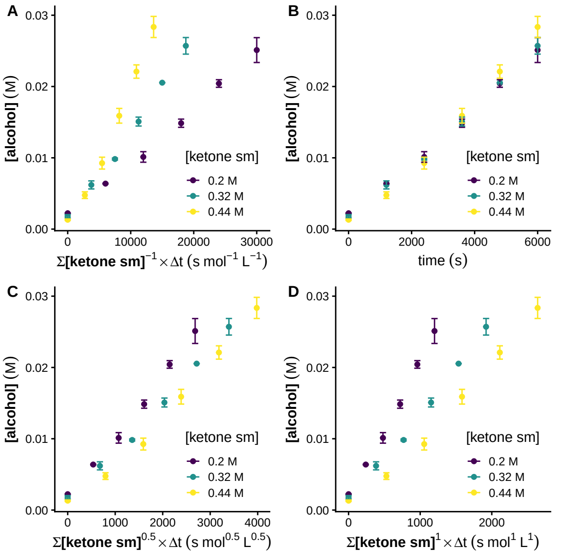

(a) Alcohol product (**7**)

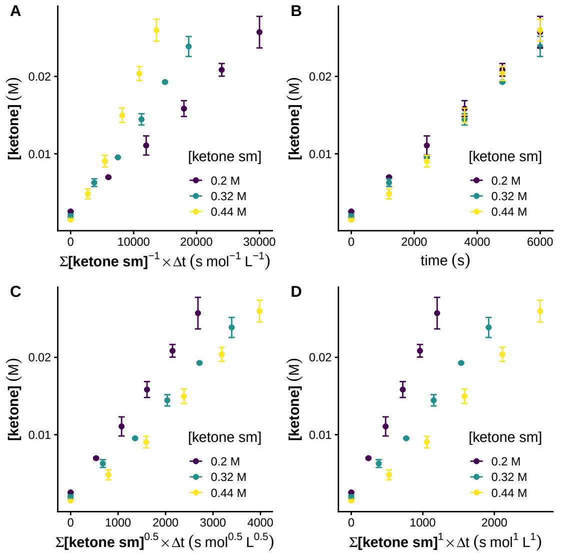

(b) Ketone product (**8**)

Figure S29: Plot of the concentration profiles of **7** and **8** in dependence of the concentration of the ketone substrate (**6**). Each data point is the average of three experiments with standard deviation of the mean included as error bars. The time scale was normalized according to the VTNA method. A) $n = -1$, B) $n = 0$, C) $n = 0.5$, D) $n = 1$. Graphical analysis determined that the curves overlap best for $n = 0$.

6.6.5 Order in base

Table S13: Initial rates.

Entry[a]	$[K_3PO_4]$ (M)	rate [alcohol (**7**)] ($M\ s^{-1}$)[b]	rate [ketone (**8**)] ($M\ s^{-1}$)[b]
1	0.100	$3.19(22) \times 10^{-6}$	$3.76(26) \times 10^{-6}$
1	0.200	$3.47(16) \times 10^{-6}$	$3.67(25) \times 10^{-6}$
3	0.400	$3.51(12) \times 10^{-6}$	$4.20(19) \times 10^{-6}$

[a] General conditions: **5** (0.300 M), **6** (0.200 M), **1** (0.010 M), *n*-dodecane (0.097 M).
[b] Standard errors of the mean are reported in parentheses.

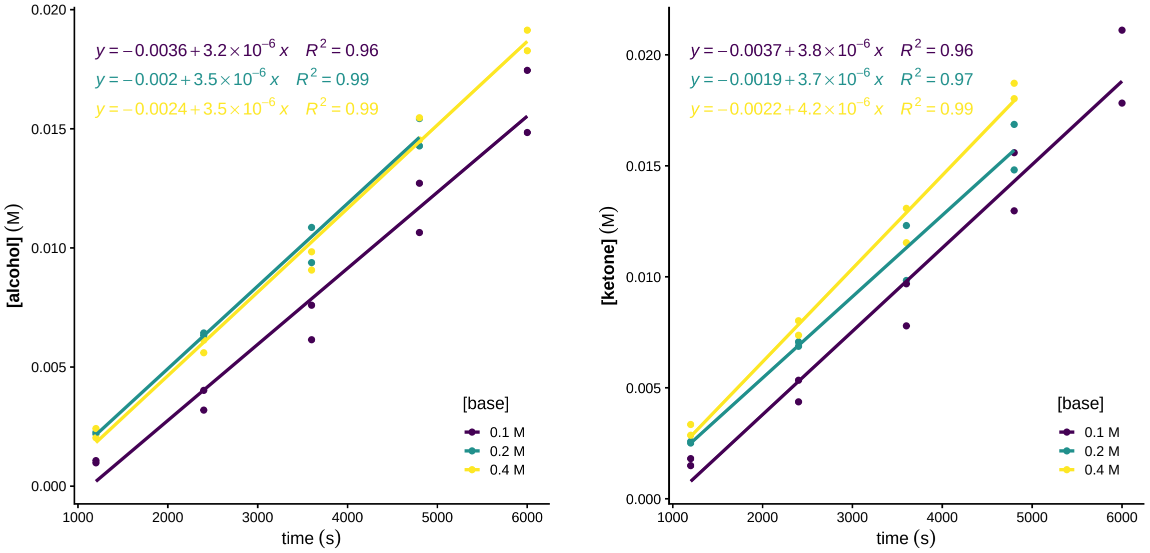

(a) Alcohol product (**7**) **(b)** Ketone product (**8**)

Figure S30: Plot of the concentration profiles of **7** and **8** in dependence of the concentration of the base (K_3PO_4). Each reaction was run in duplicate in parallel.

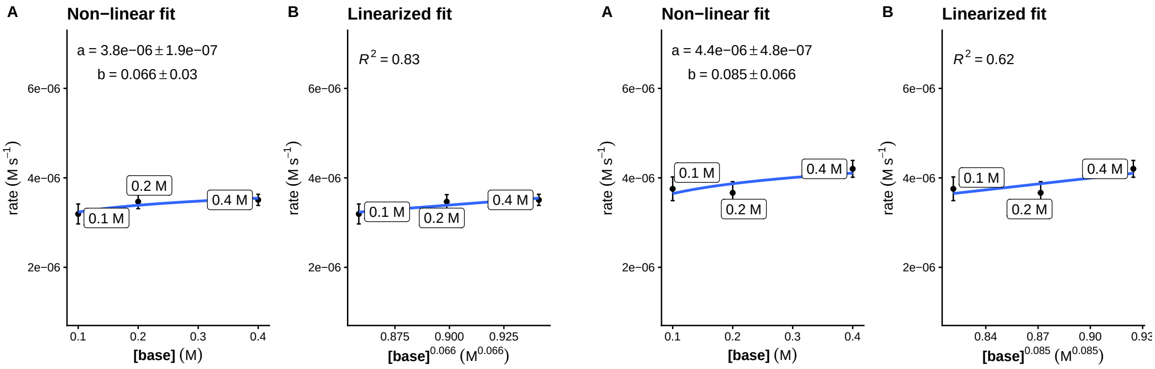

(a) Alcohol product (**7**) **(b)** Ketone product (**8**)

Figure S31: Plot of the initial reaction rate in dependence of the concentration of the base (K_3PO_4). Each data point is the average of two experiments with standard deviation of the mean included as error bars. A) Non-linear exponential fit to determine order; B) Linearized fit with obtained order.

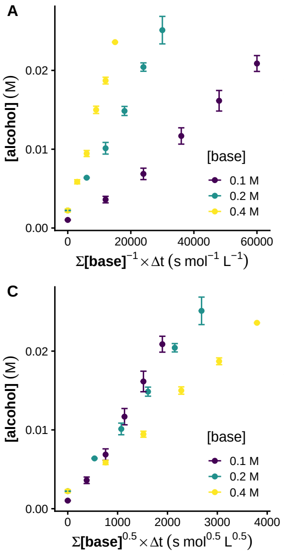

(a) Alcohol product (**7**)

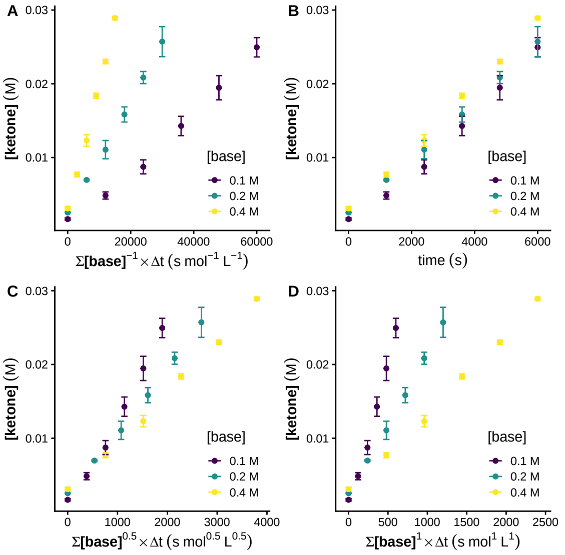

(b) Ketone product (**8**)

Figure S32: Plot of the concentration profiles of **7** and **8** in dependence of the concentration of the base (K_3PO_4). Each data point is the average of three experiments with standard deviation of the mean included as error bars. The time scale was normalized according to the VTNA method. A) $n = -1$, B) $n = 0$, C) $n = 0.5$, D) $n = 1$. Graphical analysis determined that the curves overlap best for $n = 0$.

6.6.6 Addition of COD

Table S14: Initial rates.

Entry[a]	[COD] (M)	rate [alcohol (**7**)] (M s^{-1})[b]	rate [ketone (**8**)] (M s^{-1})[b]
1	0.010	4.15(10) × 10^{-6}	5.93(20) × 10^{-6}
2	0.040	3.52(11) × 10^{-6}	5.24(30) × 10^{-6}
3	0.070	3.06(13) × 10^{-6}	5.28(29) × 10^{-6}
4	0.10	2.47(5) × 10^{-6}	5.08(19) × 10^{-6}

[a] General conditions: **5** (0.300 M), **6** (0.200 M), K_3PO_4 (0.200 M), **1** (0.010 M), *n*-dodecane (0.097 M).
[b] Estimate from linear regression with standard error of the mean.

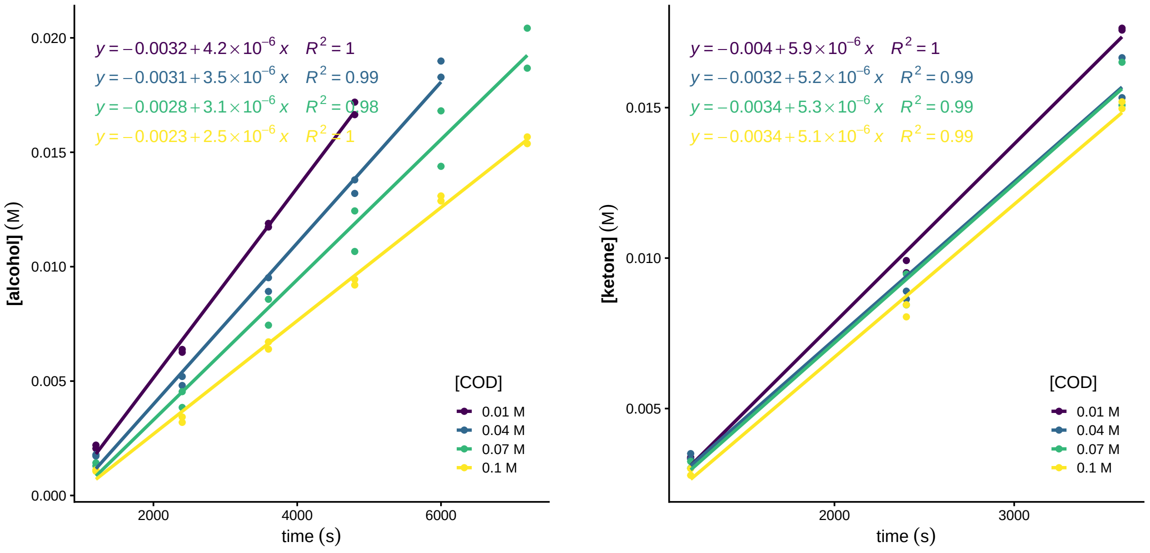

(a) Alcohol product (**7**) **(b)** Ketone product (**8**)

Figure S33: Plot of the concentration profiles of **7** and **8** in dependence of the concentration of COD. Each reaction was run in duplicate in parallel.

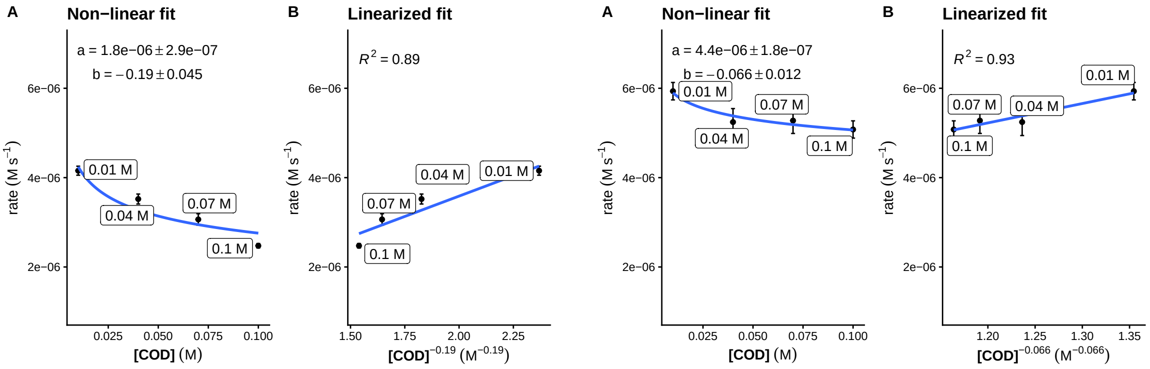

(a) Alcohol product (**7**) **(b)** Ketone product (**8**)

Figure S34: Plot of the initial reaction rate in dependence of the concentration of COD. Each data point is the average of two experiments with standard deviation of the mean included as error bars. A) Non-linear exponential fit to determine order; B) Linearized fit with obtained order.

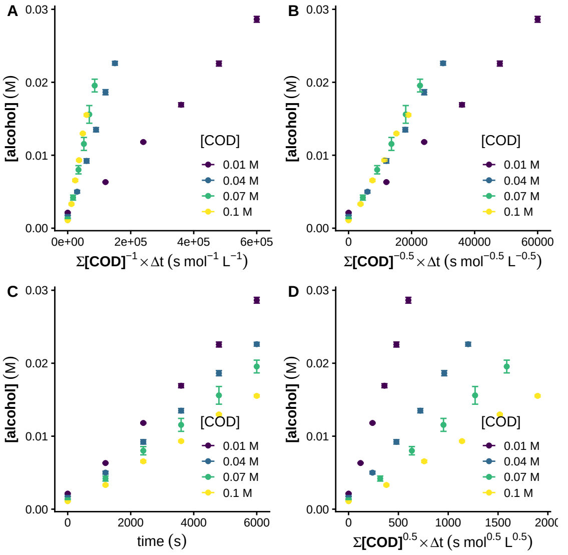

(a) Alcohol product (**7**)

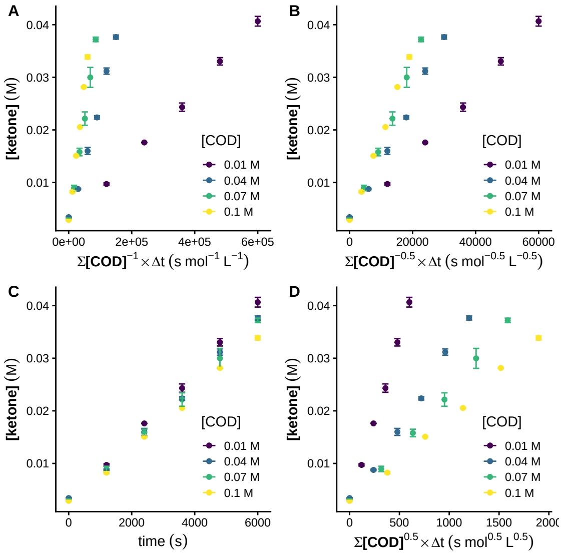

(b) Ketone product (**8**)

Figure S35: Plot of the concentration profiles of **7** and **8** in dependence of the concentration of COD. Each data point is the average of three experiments with standard deviation of the mean included as error bars. The time scale was normalized according to the VTNA method. A) $n = -1$, B) $n = -0.5$, C) $n = 0$, D) $n = 0.5$. Graphical analysis determined that the curves overlap best for $n = 0$.

A small negative order for the alcohol product was observed, while the order for the ketone product is nearly zero. Therefore, COD does not significantly inhibit the reaction.

6.6.7 Order in catalyst with added COD

Table S15: Initial rates.

Entry[a]	[**1**] (M)	[COD] (M)	rate [alcohol (**7**)] ($M\,s^{-1}$)[b]	rate [ketone (**8**)] ($M\,s^{-1}$)[b]
1	0.0025	0.050	$1.41(4) \times 10^{-6}$	$2.46(8) \times 10^{-6}$
2	0.0050	0.050	$2.16(4) \times 10^{-6}$	$3.73(11) \times 10^{-6}$
3	0.010	0.050	$2.73(6) \times 10^{-6}$	$4.46(16) \times 10^{-6}$
4	0.020	0.050	$3.21(11) \times 10^{-6}$	$5.04(18) \times 10^{-6}$

[a] General conditions: **5** (0.300 M), **6** (0.200 M), K_3PO_4 (0.200 M), *n*-dodecane (0.097 M).
[b] Estimate from linear regression with standard error of the mean.

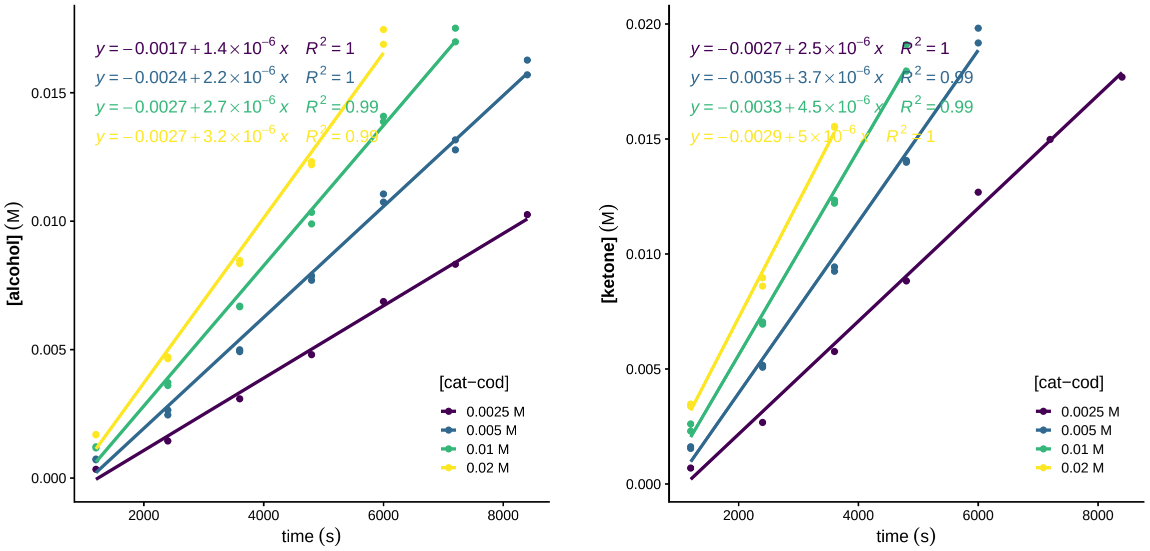

(a) Alcohol product (**7**) **(b)** Ketone product (**8**)

Figure S36: Plot of the concentration profiles of **7** and **8** in dependence of the concentration of the catalyst (**1**) in presence of an excess of COD. Each reaction was run in duplicate in parallel.

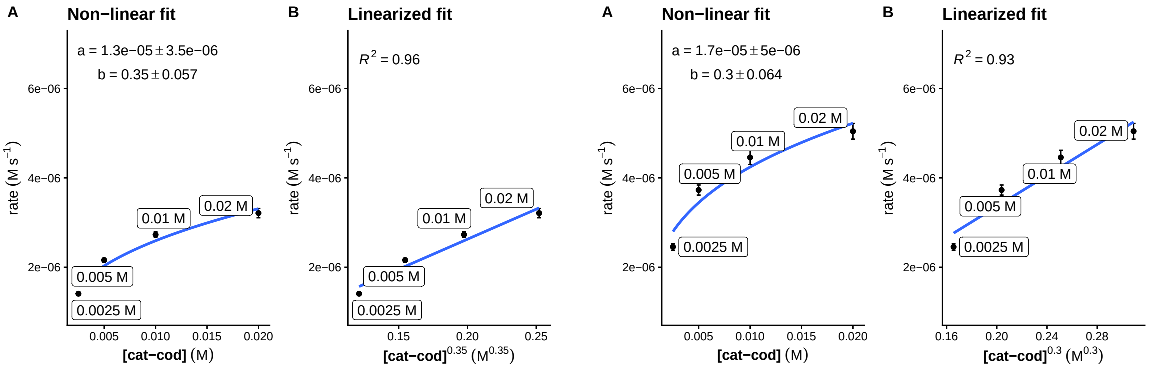

(a) Alcohol product (**7**) **(b)** Ketone product (**8**)

Figure S37: Plot of the initial reaction rate in dependence of the concentration of the catalyst (**1**) in presence of an excess of COD. Each data point is the average of two experiments with standard deviation of the mean included as error bars. A) Non-linear exponential fit to determine order; B) Linearized fit with obtained order.

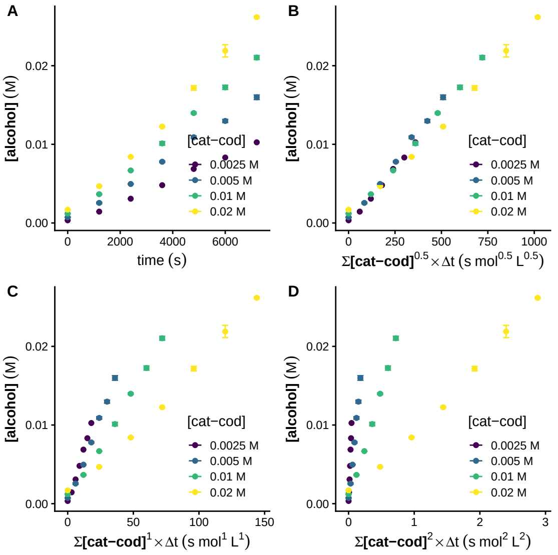

(a) Alcohol product (**7**)

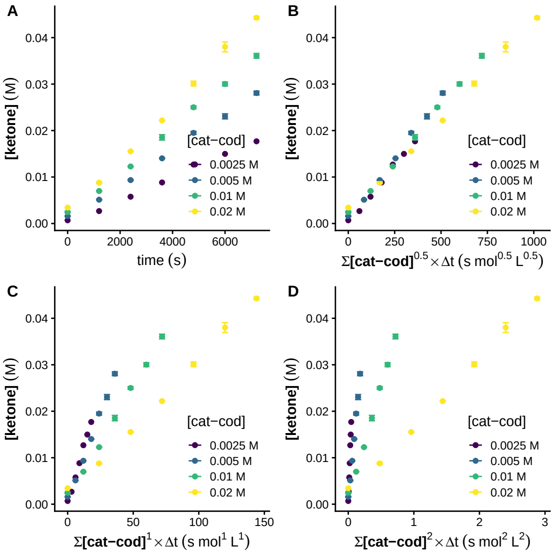

(b) Ketone product (**8**)

Figure S38: Plot of the concentration profiles of **7** and **8** in dependence of the concentration of the catalyst (**1**) in presence of an excess of COD. Each data point is the average of three experiments with standard deviation of the mean included as error bars. The time scale was normalized according to the VTNA method. A) $n = 0$, B) $n = 0.5$, C) $n = 1$, D) $n = 2$. Graphical analysis determined that the curves overlap best for $n = 0.5$.

The order in catalyst does not increase under COD saturation conditions, disproving the hypothesis that the half order in catalyst arises from COD dissociation.

6.6.8 Addition of TBACl

Due to the limited solubility of the introduced chloride source tetrabutylammonium chloride (TBACl), the obtained results are not conclusive and should rather be interpreted in the context of the whole study. Regardless, there is a qualitative trend for an increased inhibition effect with increasing chloride concentration.

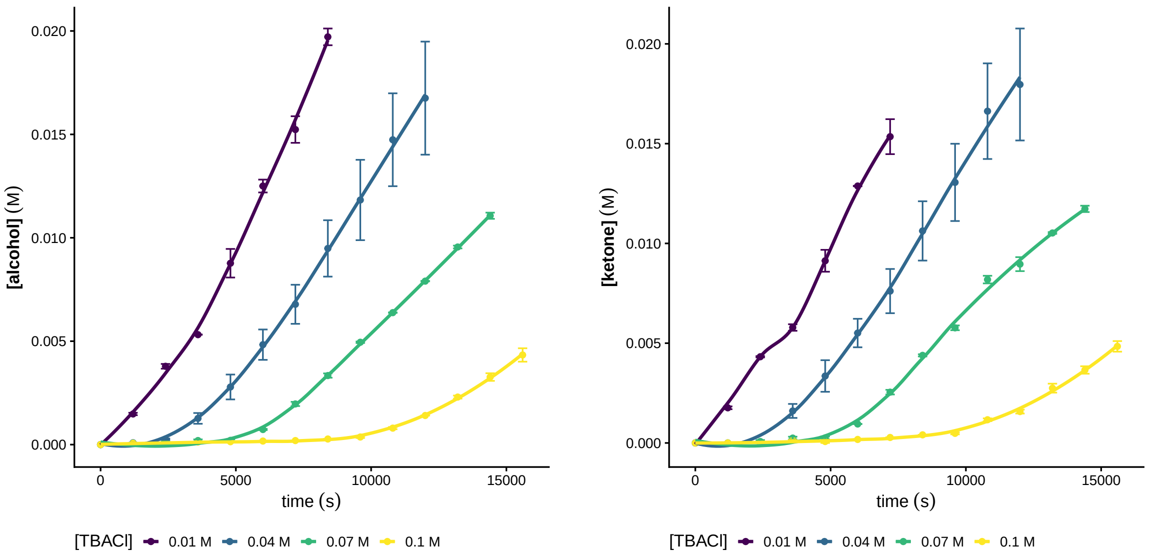

(a) Alcohol product (**7**)

(b) Ketone product (**8**)

Figure S39: Plot of the concentration profiles of **7** and **8** in dependence of the concentration of the TBACl, including early time points. Each data point is the average of two experiments with standard deviation of the mean included as error bars. The line serves as a guide to the eye, it is not a fit.

An induction period that increased with increasing chloride concentration in the system was observed.

Table S16: Initial rates.

Entry[a]	[TBACl] (M)	rate [alcohol (**7**)] ($M\,s^{-1}$)[b]	rate [ketone (**8**)] ($M\,s^{-1}$)[b]
1	0.010	$2.52(11) \times 10^{-6}$	$2.31(11) \times 10^{-6}$
2	0.040	$1.90(16) \times 10^{-6}$	$2.05(17) \times 10^{-6}$
3	0.070	$1.271(13) \times 10^{-6}$	$1.28(5) \times 10^{-6}$
4	0.10	$0.75(4) \times 10^{-6}$	$0.79(6) \times 10^{-6}$

[a] General conditions: **5** (0.300 M), **6** (0.200 M), K_3PO_4 (0.200 M), **1** (0.010 M), *n*-dodecane (0.097 M).
[b] Estimate from linear regression with standard error of the mean.

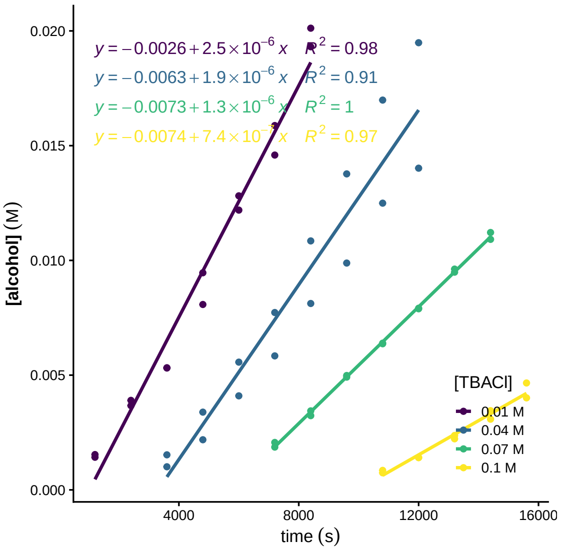

(a) Alcohol product (**7**)

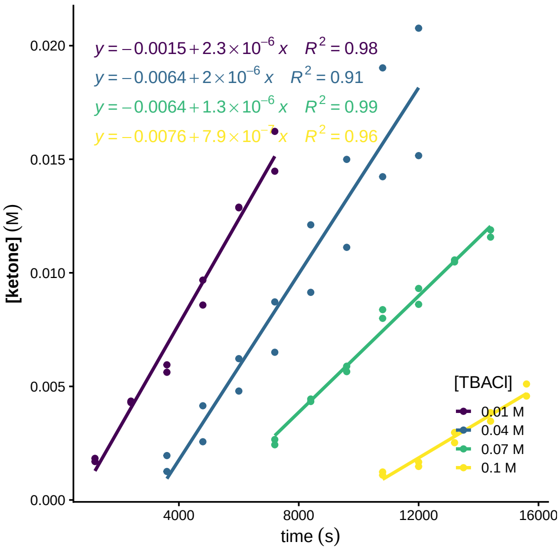

(b) Ketone product (**8**)

Figure S40: Plot of the concentration profiles of **7** and **8** in dependence of the concentration of TBACl. Each reaction was run in duplicate in parallel. The induction period was not included in the analysis.

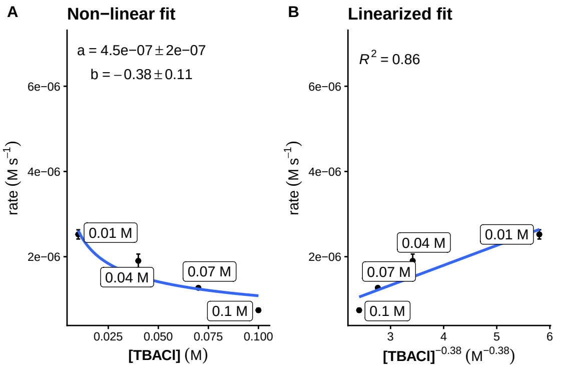

(a) Alcohol product (**7**)

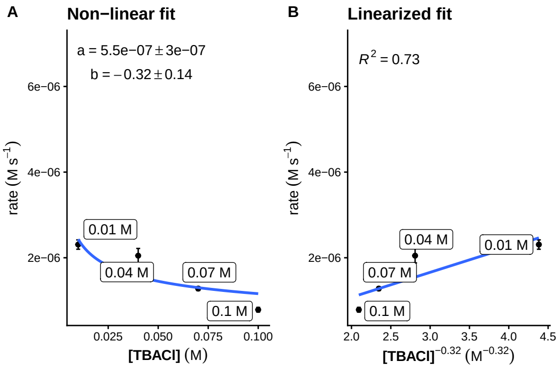

(b) Ketone product (**8**)

Figure S41: Plot of the initial reaction rate in dependence of the concentration of TBACl. Each data point is the average of two experiments with standard deviation of the mean included as error bars. A) Non-linear exponential fit to determine order; B) Linearized fit with obtained order.

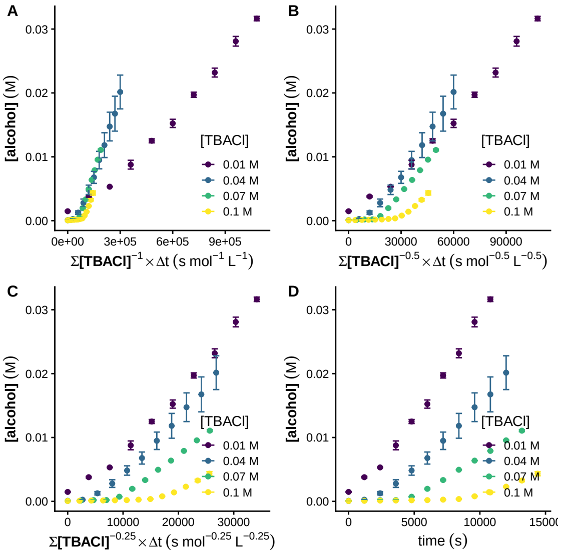

(**a**) Alcohol product (**7**)

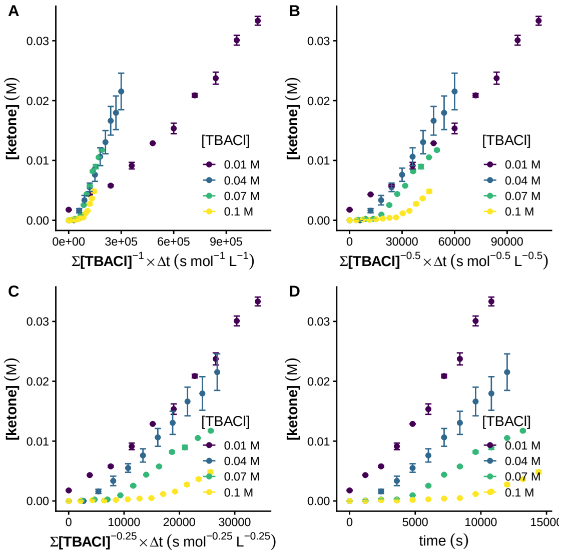

(**b**) Ketone product (**8**)

Figure S42: Plot of the concentration profiles of **7** and **8** in dependence of the concentration of TBACl. Each data point is the average of three experiments with standard deviation of the mean included as error bars. The time scale was normalized according to the VTNA method. A) $n = -1$, B) $n = -0.5$, C) $n = -0.25$, D) $n = 0$. Graphical analysis determined that the curves overlap best for $n = -0.25$.

6.6.9 Order in catalyst with added chloride

Due to the limited solubility of the introduced chloride source TBACl, the obtained results are not conclusive and should rather be interpreted in the context of the whole study.

Table S17: Initial rates.

Entry[a]	[**1**] (M)	[TBACl] (M)	rate [alcohol (**7**)] (M s^{-1})[b]	rate [ketone (**8**)] (M s^{-1})[b]
1	0.0025	0.050	1.31(6) × 10^{-6}	1.80(9) × 10^{-6}
2	0.0050	0.050	1.68(19) × 10^{-6}	2.20(15) × 10^{-6}
3	0.010	0.050	2.09(19) × 10^{-6}	2.56(25) × 10^{-6}

[a] General conditions: **5** (0.300 M), **6** (0.200 M), K_3PO_4 (0.200 M), *n*-dodecane (0.097 M).
[b] Estimate from linear regression with standard error of the mean.

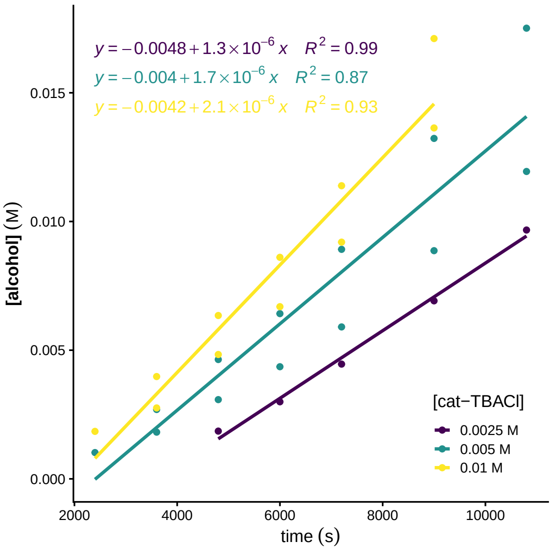

(a) Alcohol product (**7**)

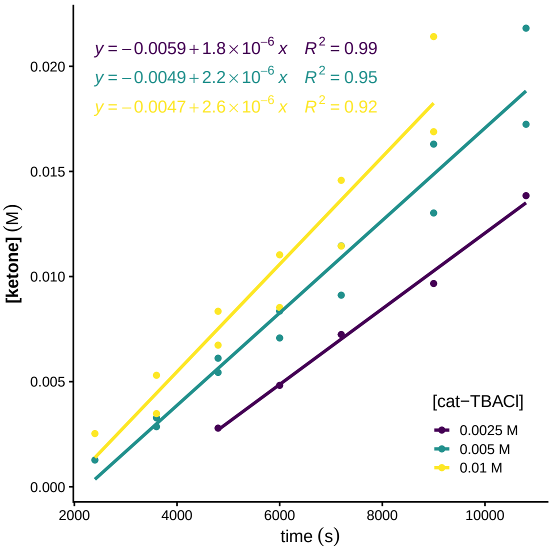

(b) Ketone product (**8**)

Figure S43: Plot of the concentration profiles of **7** and **8** in dependence of the concentration of the catalyst (**1**) in presence of an excess of TBACl. Each reaction was run in duplicate in parallel.

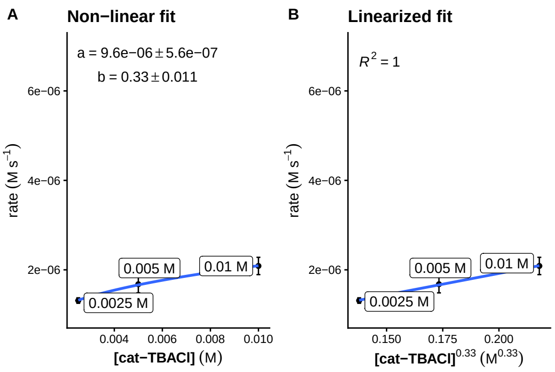

(a) Alcohol product (**7**)

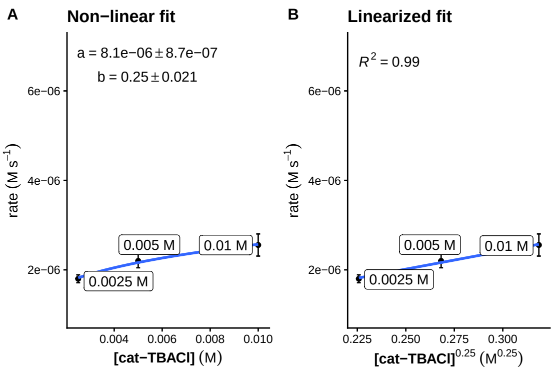

(b) Ketone product (**8**)

Figure S44: Plot of the initial reaction rate in dependence of the concentration of catalyst (**1**) in presence of an excess of TBACl. Each data point is the average of two experiments with standard deviation of the mean included as error bars. A) Non-linear exponential fit to determine order; B) Linearized fit with obtained order.

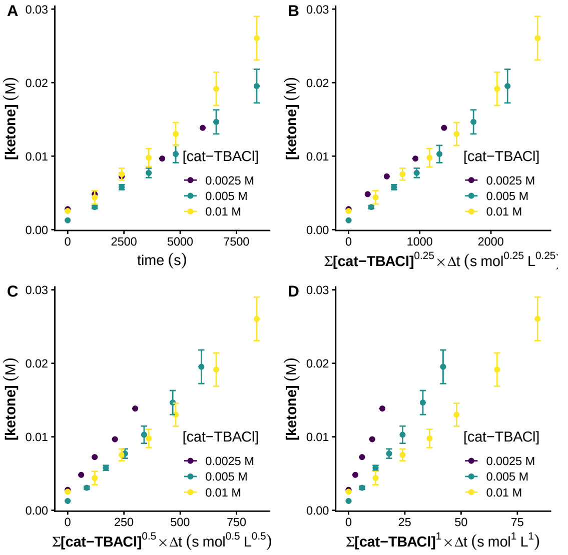

(a) Ketone product (**8**)

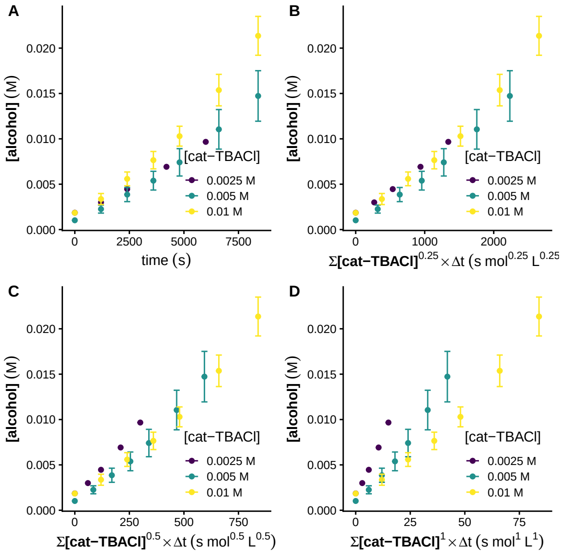

(b) Alcohol product (**7**)

Figure S45: Plot of the concentration profiles of **7** and **8** in dependence of the concentration of the catalyst (**1**) in presence of an excess of TBACl. Each data point is the average of three experiments with standard deviation of the mean included as error bars. The time scale was normalized according to the VTNA method. A) $n = 0$, B) $n = 0.25$, C) $n = 0.5$, D) $n = 1$. Graphical analysis determined that the curves overlap best for $n = 0.25$.

6.6.10 Observed rate constant

When plotting the concentration profiles against the VTNA time scale where the order of all reagents (**1**, **5** and **6** (and base)) is raised to their correct order, a straight line is obtained with its slope corresponding to the rate constant k_{obs}.[19]

Table S18: Observed rate constant (k_{obs}).

k_{obs} [alcohol (**7**)] (M s$^{-1.5}$)^a	k_{obs} [ketone (**8**)] (M s$^{-1.5}$)^a
1.08(4) × 10^{-4}	1.50(4) × 10^{-4}

^{a} Estimate from linear regression with standard error of the mean.

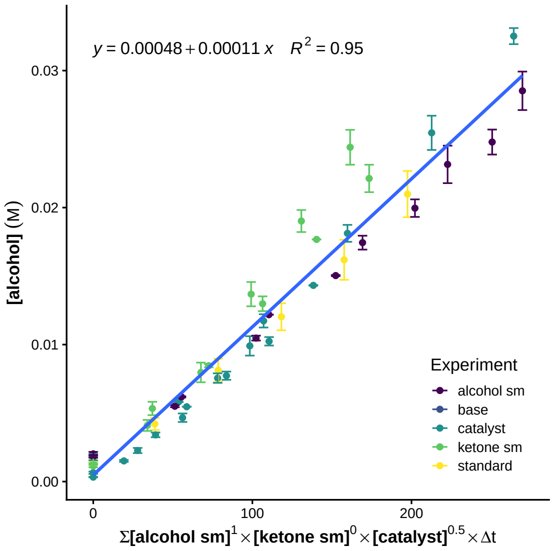

(a) Alcohol product (**7**)

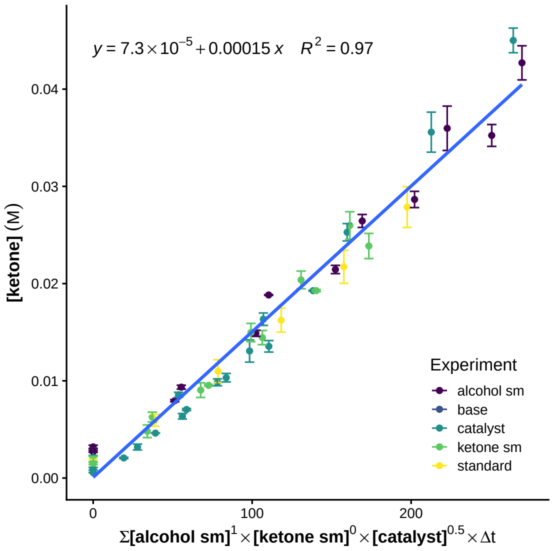

(b) Ketone product (**8**)

Figure S46: Plot of the concentration profiles of **7** and **8** in dependence of the concentrations of **1**, **5** and **6**. The time scale was normalized according to the VTNA method. The observed rate constant was extracted by linear regression of the linearized curve. Each data point is the average of two experiments with standard deviation of the mean included as error bars.

6.7 Validation of the Kinetic Analysis With a Machine Learning Model

The kinetic data for the generation of Figs. S12 and S13 was used as input for the machine learning algorithm developed by Burés and Larrosa.[20] The pre-trained model by the authors was used as provided. The full model "`M1_20_model.h5`" including substrate and product concentrations and product inhibition was used. The code was run with *Python* 3.9.

Four kinetic runs in triplicates were used as input without averaging (Table S19). Three runs describe the order in catalyst (**1**) and the fourth run is a "same-excess" product inhibition experiment with reduced amounts of starting materials and added products. The "same-excess" experiment was conducted for both products separately. Because all runs must have the same number of time points, 11 time points ranging from 0–540 min were used for the analysis.

Table S19: Kinetic data used as input for machine learning algorithm.

Entry	[Rh] (**1**) (M)	[a. sm (**5**)] (M)	[k. sm (**6**)] (M)	[a. product (**7**)] (M)	[k. product (**8**)] (M)
1	0.010	0.300	0.200	0	0
2	0.015	0.300	0.200	0	0
3	0.020	0.300	0.200	0	0
4	0.010	0.240	0.140	0.060	0
5	0.010	0.240	0.140	0	0.060

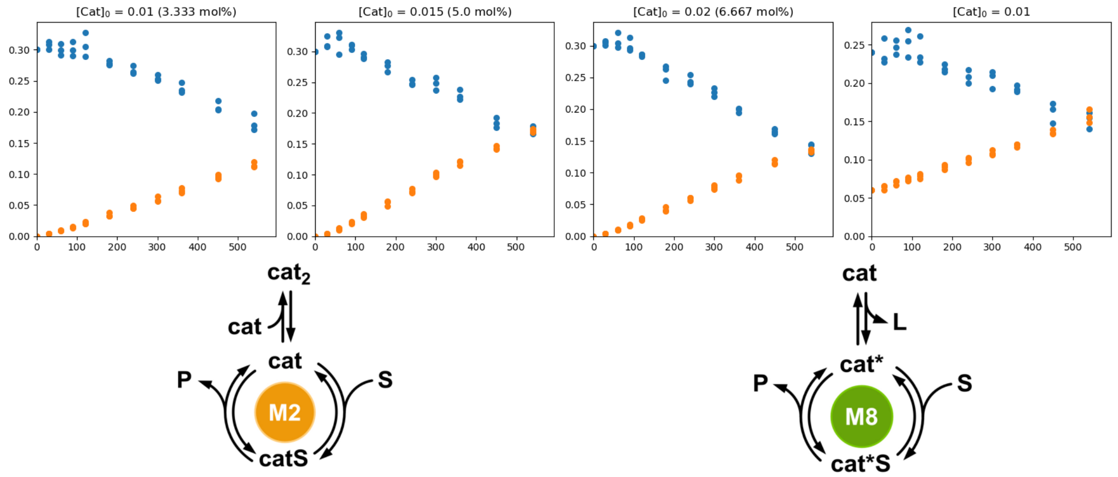

(a) Alcohol starting material (**5**) and alcohol product (**7**).

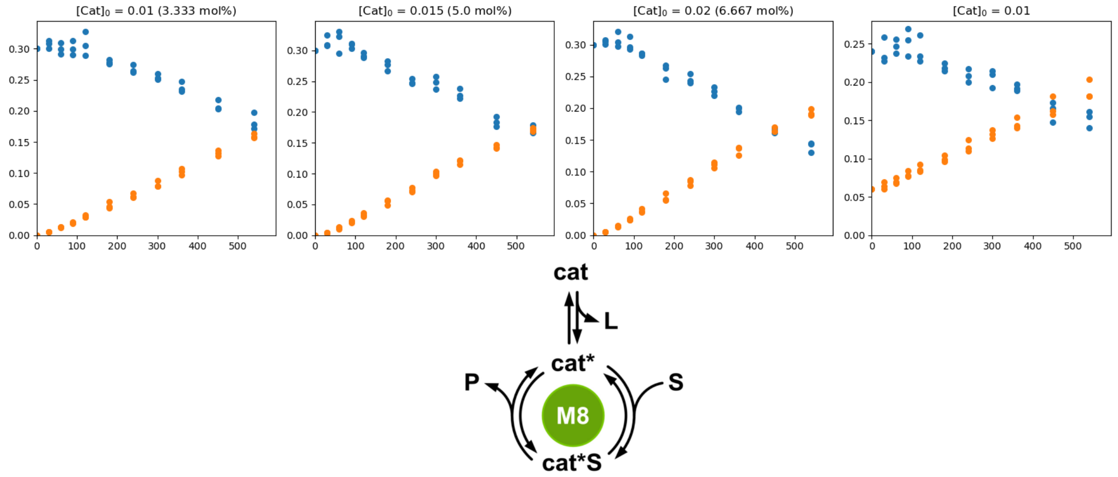

(b) Alcohol starting material (**5**) and ketone product (**8**).

Figure S47: Kinetic data used for machine learning algorithm and prediction for four substrate to product pairs.

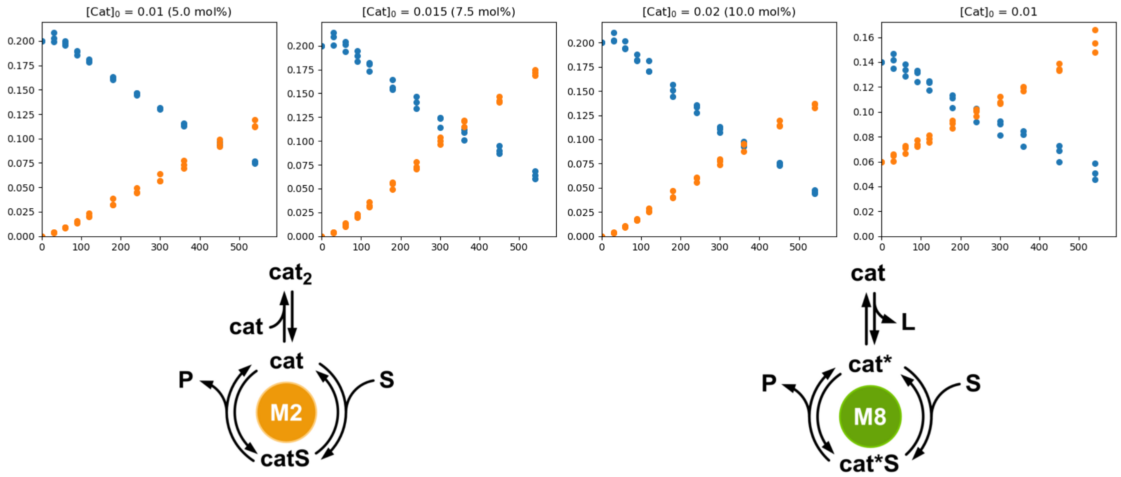

(c) Ketone starting material (**6**) and alcohol product (**7**).

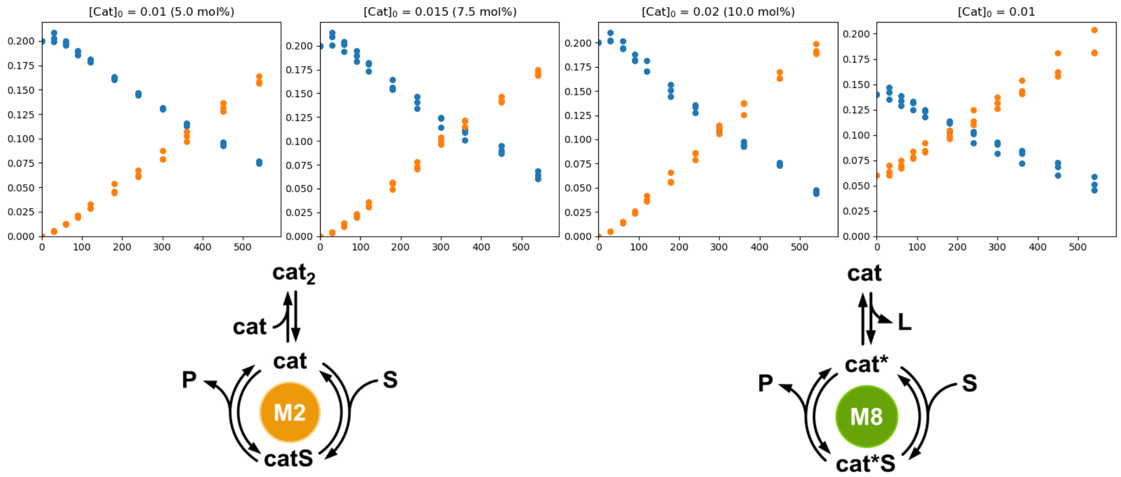

(d) Ketone starting material (**6**) and ketone product (**8**).

Figure S47: Kinetic data used for machine learning algorithm and prediction for four substrate to product pairs.

6.8 Rationalization for the Fractional Order in [Rh]

In the kinetic studies (cf. Section 6.5) a half-order dependence in [Rh] was observed. This fractional order can implicate different mechanistic scenarios, which are discussed in the following.

6.8.1 Derivation for a Monomer-dimer Equilibrium

The derivation shown here is based on the literature by Blackmond and van Gemmeren.[21,22]

Equilibrium between active monomer and inactive dimer:

$$2\,\mathrm{RhL_n} \rightleftharpoons (\mathrm{RhL_n})_2 \tag{1}$$

Steady state approximation – Rh-species accumulates before the rate determining step/sequence:

$$[\mathrm{Rh}]_{\mathrm{tot}} \simeq [\mathrm{RhL_n}] + 2\,[(\mathrm{RhL_n})_2] \tag{2}$$

$$[(\mathrm{RhL_n})_2] = \frac{1}{2}\,([\mathrm{Rh}]_{\mathrm{tot}} - [\mathrm{RhL_n}]) \tag{3}$$

Equilibrium constant of the monomer-dimer-equilibrium:

$$K = \frac{[(\mathrm{RhL_n})_2]}{[\mathrm{RhL}_n]^2} \tag{4}$$

$$K = \frac{[\mathrm{Rh}]_{\mathrm{tot}} - [\mathrm{RhL_n}]}{2\,[\mathrm{RhL_n}]^2} \tag{5}$$

$$0 = 2K\,[\mathrm{RhL_n}]^2 + [\mathrm{RhL_n}] - [\mathrm{Rh}]_{\mathrm{tot}} \tag{6}$$

$$[\mathrm{RhL_n}] = \frac{-1 \pm \sqrt{1^2 - 8K(-1)[\mathrm{Rh}]_{\mathrm{tot}}}}{4K} \tag{7}$$

$$[\mathrm{RhL_n}] = \frac{-1 + \sqrt{8K[\mathrm{Rh}]_{\mathrm{tot}} + 1}}{4K} \tag{8}$$

$$[\mathrm{RhL_n}] = \frac{\sqrt{8K[\mathrm{Rh}]_{\mathrm{tot}} + 1}}{4K} - \frac{1}{4K} \tag{9}$$

$$\blacksquare \quad [\mathrm{RhL_n}] \propto \sqrt{[\mathrm{Rh}]_{\mathrm{tot}}} \tag{10}$$

If the resting state is a dimer, the order in [Rh] stays 0.5 irregardless of the concentration of other reagents or ligands, unless other equilibria influence the system.

6.8.2 Ligand Dissociation

Reversible dissociation of a ligand can also lead to an observed half-order in catalyst. In the present case, this could either be a chloride or a COD ligand. The derivation shown here is based on the literature by Hartwig,[23] as well as Blackmond and van Gemmeren.[21,22]

The equilibrium between a resting state bearing a COD ligand and an active species without COD is described as follows:

$$\mathrm{RhL_n} + \mathrm{COD} \rightleftharpoons \mathrm{Rh(COD)L_n} \tag{11}$$

Steady state approximation – Rh-species accumulates before the rate determining step/sequence:

$$[\mathrm{Rh}]_{\mathrm{tot}} \simeq [\mathrm{RhL_n}] + [\mathrm{Rh(COD)L_n}] \tag{12}$$

$$[\mathrm{Rh(COD)L_n}] = [\mathrm{Rh}]_{\mathrm{tot}} - [\mathrm{RhL_n}] \tag{13}$$

The mass balance of COD is as follows:

$$[\mathrm{COD}]_{\mathrm{tot}} = [\mathrm{COD}] + [\mathrm{Rh(COD)L_n}] \tag{14}$$

$$[\mathrm{COD}] = [\mathrm{COD}]_{\mathrm{tot}} - [\mathrm{Rh(COD)L_n}] \tag{15}$$

Equilibrium constant of the COD ligand-dissociation equilibrium:

$$K = \frac{[\mathrm{Rh(COD)L_n}]}{[\mathrm{RhL_n}]\,[\mathrm{COD}]} \tag{16}$$

$$K = \frac{[\mathrm{Rh}]_{\mathrm{tot}} - [\mathrm{RhL_n}]}{[\mathrm{RhL_n}]\,([\mathrm{COD}]_{\mathrm{tot}} - [\mathrm{Rh(COD)L_n}])} \tag{17}$$

$$K = \frac{[\mathrm{Rh}]_{\mathrm{tot}} - [\mathrm{RhL_n}]}{[\mathrm{RhL_n}]\,([\mathrm{COD}]_{\mathrm{tot}} - ([\mathrm{Rh}]_{\mathrm{tot}} - [\mathrm{RhL_n}]))} \tag{18}$$

$$K = \frac{[\mathrm{Rh}]_{\mathrm{tot}} - [\mathrm{RhL_n}]}{[\mathrm{RhL_n}]\,([\mathrm{COD}]_{\mathrm{tot}} - [\mathrm{Rh}]_{\mathrm{tot}} + [\mathrm{RhL_n}])} \tag{19}$$

Case 1: Under the normal catalytic conditions the amount of COD and Rh are equal:

$$[\mathrm{Rh}]_{\mathrm{tot}} = [\mathrm{COD}]_{\mathrm{tot}} \tag{20}$$

Therefore, Eq. (19) simplifies to:

$$K = \frac{[\mathrm{Rh}]_{\mathrm{tot}} - [\mathrm{RhL_n}]}{[\mathrm{RhL_n}]^2} \tag{19}$$

$$0 = K\,[\mathrm{RhL_n}]^2 + [\mathrm{RhL_n}] - [\mathrm{Rh}]_{\mathrm{tot}} \tag{21}$$

$$[\mathrm{RhL_n}] = \frac{-1 \pm \sqrt{1^2 - 4K(-1)[\mathrm{Rh}]_{\mathrm{tot}}}}{2K} \tag{22}$$

$$[\mathrm{RhL_n}] = \frac{-1 + \sqrt{4K[\mathrm{Rh}]_{\mathrm{tot}} + 1}}{2K} \tag{23}$$

$$[\mathrm{RhL_n}] = \frac{\sqrt{4K[\mathrm{Rh}]_{\mathrm{tot}} + 1}}{2K} - \frac{1}{2K} \tag{24}$$

$$\blacksquare \quad [\mathrm{RhL_n}] \propto \sqrt{[\mathrm{Rh}]_{\mathrm{tot}}} \tag{25}$$

Under these conditions, a half order in [Rh] is expected.

Case 2: However, when an excess of COD is provided relative to the catalyst, the amount of free COD is similar to the initial concentration:

$$\text{When } [\mathrm{RhL_n}] \ll [\mathrm{COD}] \tag{26}$$

$$[\mathrm{COD}]_{\mathrm{tot}} \simeq [\mathrm{COD}] \tag{27}$$

Therefore, the equilibrium constant from Eq. (16) with use of Eqs. (13) and (27) simplifies to:

$$K = \frac{[\mathrm{Rh(COD)L_n}]}{[\mathrm{RhL_n}]\,[\mathrm{COD}]} \tag{16}$$

$$K = \frac{[\mathrm{Rh}]_{\mathrm{tot}} - [\mathrm{RhL_n}]}{[\mathrm{RhL_n}]\,[\mathrm{COD}]_{\mathrm{tot}}} \tag{28}$$

$$K\,[\mathrm{RhL_n}]\,[\mathrm{COD}]_{\mathrm{tot}} = [\mathrm{Rh}]_{\mathrm{tot}} - [\mathrm{RhL_n}] \tag{29}$$

$$[\mathrm{RhL_n}]\,(K\,[\mathrm{COD}]_{\mathrm{tot}} + 1) = [\mathrm{Rh}]_{\mathrm{tot}} \tag{30}$$

$$[\mathrm{RhL_n}] = \frac{[\mathrm{Rh}]_{\mathrm{tot}}}{K\,[\mathrm{COD}]_{\mathrm{tot}} + 1} \tag{31}$$

$$\blacksquare \quad [\mathrm{RhL_n}] \propto [\mathrm{Rh}]_{\mathrm{tot}} \propto [\mathrm{COD}]^{-1} \tag{32}$$

Under these conditions, the order in [Rh] changes to 1 and is inverse in [COD].

The same treatment can be done for chloride.

6.8.3 Discussion

Under the standard reaction conditions both a monomer-dimer equilibrium and a dissociation equilibrium with COD can lead to a half order in [Rh]. However, when an excess of [COD] is provided, the order in [Rh] would become 1 if a dissociation equilibrium occurs.

The kinetic experiments in Sections 6.6.6, 6.6.7 and 6.6.9 disprove the hypothesis that complete COD dissociation occurs before or during the turnover-limiting step. Partial COD dissociation from κ^4 to κ^2, however, would also fit to the kinetic data. Meanwhile, it was not possible to determine the order in chloride due to the limited solubility of the used chloride source (cf. Section 6.6.8) which can lead to misleading conclusions. However, increasing the chloride concentration led to inhibition which is in agreement with a kinetically relevant chloride dissociation step.

While these results do not exclude a dimeric resting state, NMR studies disprove the existence of dimeric species in solution (see Section 7.2).

6.9 Derivation of Theoretical Rate Law

The rate law for the transfer hydroarylation was theoretically derived by considering the following pathway (Scheme S2). We assume that all steps are reversible, however, the model reaction is far from equilibrium and we therefore assume that the turnover-limiting step (tls) is irreversible under the experimental reaction conditions used for the kinetic measurements. From the experimental data we assume further that the turnover-limiting step occurs during the first half of the catalytic cycle (before ketone association).

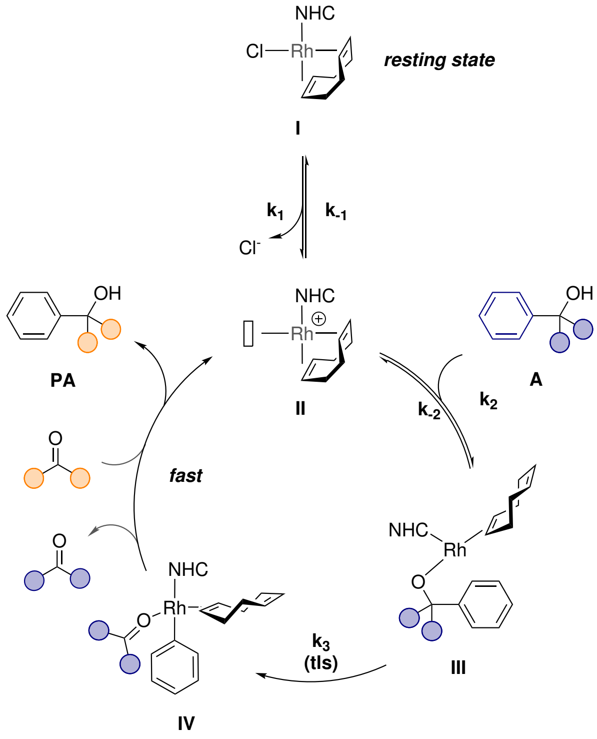

Scheme S2: Kinetic model.

If the rate-determining step is the conversion between **III** and **IV** we can assume that the rate of alcohol product formation ($\frac{d[\text{PA}]}{dt}$) is equal to the rate of the formation of **IV**:

$$rate = \frac{d[\text{PA}]}{dt} = \frac{d[\mathbf{IV}]}{dt} = k_3[\mathbf{III}] \tag{33}$$

Mass balance of the different Rh species ([**IV**] can be considered negligible):

$$[\text{Rh}]_{\text{tot}} = [\mathbf{I}] + [\mathbf{II}] + [\mathbf{III}] \tag{34}$$

The mass balance of chloride:

$$[\text{Cl}^-]_{\text{tot}} = [\mathbf{II}] + [\mathbf{III}] \tag{35}$$

Using the steady-state approximation for [**I**] and [**III**]:

$$\frac{d[\mathbf{I}]}{dt} = 0 = k_{-1}[\mathbf{II}][\text{Cl}^-] - k_1[\mathbf{I}] \tag{36}$$

$$[\mathbf{I}] = \frac{k_{-1}}{k_1}[\mathbf{II}][\text{Cl}^-] = \frac{1}{K_1}[\mathbf{II}][\text{Cl}^-] \tag{37}$$

$$\frac{d[\mathbf{III}]}{dt} = 0 = k_2[\mathbf{II}][\text{A}] - k_{-2}[\mathbf{III}] - k_3[\mathbf{III}] \tag{38}$$

$$[\mathbf{III}] = \frac{k_2}{k_{-2} + k_3}[\text{A}][\mathbf{II}] \tag{39}$$

Substituting Eq. (35) into Eq. (37):

$$[\mathbf{I}] = \frac{1}{K_1}[\mathbf{II}]([\mathbf{II}] + [\mathbf{III}]) \tag{40}$$

$$= \frac{1}{K_1}[\mathbf{II}]^2 + \frac{1}{K_1}[\mathbf{II}][\mathbf{III}] \tag{41}$$

Substituting Eq. (39) into Eq. (41):

$$[\mathbf{I}] = \frac{1}{K_1}[\mathbf{II}]^2 + \frac{1}{K_1}[\mathbf{II}]\frac{k_2}{k_{-2} + k_3}[\text{A}][\mathbf{II}] \tag{42}$$

$$= \frac{1}{K_1}[\mathbf{II}]^2 + \frac{1}{K_1}[\mathbf{II}]^2\frac{k_2}{k_{-2} + k_3}[\text{A}] \tag{43}$$

$$= \frac{1}{K_1}[\mathbf{II}]^2\left(1 + \frac{k_2}{k_{-2} + k_3}[\text{A}]\right) \tag{44}$$

Now substituting Eqs. (39) and (44) into Eq. (34):

$$[\text{Rh}]_{\text{tot}} = \frac{1}{K_1}[\mathbf{II}]^2\left(1 + \frac{k_2}{k_{-2} + k_3}[\text{A}]\right) + [\mathbf{II}] + \frac{k_2}{k_{-2} + k_3}[\text{A}][\mathbf{II}] \tag{45}$$

$$0 = \frac{1}{K_1}\left(1 + \frac{k_2}{k_{-2} + k_3}[\text{A}]\right)[\mathbf{II}]^2 + \left(\frac{k_2}{k_{-2} + k_3}[\text{A}] + 1\right)[\mathbf{II}] - [\text{Rh}]_{\text{tot}} \tag{46}$$

To simplify for the next operations, let:

$$\alpha = \frac{k_2}{k_{-2} + k_3}[\text{A}] \qquad \beta = \frac{1}{K_1} \tag{47}$$

Solving the second order equation for [**II**] we obtain (the negative solution is unphysical because it would result in a negative concentration):

$$0 = \beta(\alpha + 1)[\mathbf{II}]^2 + (\alpha + 1)[\mathbf{II}] - [\text{Rh}]_{\text{tot}} \tag{48}$$

$$[\mathbf{II}] = \frac{-b \pm \sqrt{b^2 - 4ac}}{2a} \tag{49}$$

with $a = \beta(\alpha + 1)$, $b = (\alpha + 1)$, $c = -[\text{Rh}]_{\text{tot}}$.

(50)

$$[\mathbf{II}] = \frac{-(\alpha + 1) + \sqrt{(\alpha + 1)^2 + 4\beta(\alpha + 1)[\text{Rh}]_{\text{tot}}}}{2\beta(\alpha + 1)} \tag{51}$$

$$= \frac{-(\alpha + 1) + \sqrt{(\alpha + 1)^2(1 + \frac{4\beta[\text{Rh}]_{\text{tot}}}{\alpha+1})}}{2\beta(\alpha + 1)} \tag{52}$$

$$= \frac{-(\alpha + 1) + (\alpha + 1)\sqrt{(1 + \frac{4\beta[\text{Rh}]_{\text{tot}}}{\alpha+1})}}{2\beta(\alpha + 1)} \tag{53}$$

$$= \frac{-1 + \sqrt{(1 + \frac{4\beta[\text{Rh}]_{\text{tot}}}{\alpha+1})}}{2\beta} \tag{54}$$

Substituting Eq. (54) into Eq. (33):

$$\text{rate} = \frac{d[\text{PA}]}{dt} = k_3[\mathbf{III}] = k_3\alpha[\mathbf{II}] \tag{55}$$

$$= \frac{\alpha k_3}{2\beta}\left(-1 + \sqrt{\left(1 + \frac{4\beta[\text{Rh}]_{\text{tot}}}{\alpha + 1}\right)}\right) \tag{56}$$

expanding α and β gives:

(57)

$$\text{rate} = \frac{\frac{k_2}{k_{-2}+k_3}[\text{A}]k_3}{2\frac{1}{K_1}}\left(-1 + \sqrt{\left(1 + \frac{4\frac{1}{K_1}[\text{Rh}]_{\text{tot}}}{\frac{k_2}{k_{-2}+k_3}[\text{A}] + 1}\right)}\right) \tag{58}$$

$$= \frac{K_1 k_2 k_3}{2(k_{-2} + k_3)}[\text{A}]\left(-1 + \sqrt{\left(1 + \frac{4[\text{Rh}]_{\text{tot}}}{K_1(\frac{k_2}{k_{-2}+k_3}[\text{A}] + 1)}\right)}\right) \tag{59}$$

Equation (59) can be simplified by introducing:

$$k' = \frac{k_2}{(k_{-2} + k_3)} \tag{60}$$

$$\text{rate} = \frac{K_1 k' k_3}{2}[\text{A}]\left(-1 + \sqrt{\left(1 + \frac{4[\text{Rh}]_{\text{tot}}}{K_1(k'[\text{A}] + 1)}\right)}\right) \tag{61}$$

$$\text{rate} \propto [\text{Rh}]_{\text{tot}}^{1/2}\,[\text{A}]^1 \tag{62}$$

Integrating the rate law (Equation (62)) with respect to time allows to describe the concentrations of **PA** in terms of **A**:

$$[\text{PA}] = \frac{K_1 k' k_3}{2}[\text{A}]\left(-1 + \sqrt{\left(1 + \frac{4[\text{Rh}]_{\text{tot}}}{K_1(k'[\text{A}] + 1)}\right)}\right)t \tag{63}$$

Fitting the experimental data of all reaction profiles against Eq. (63) allows the estimation of K_1, k_3, k'. Fitting was accomplished with the `nlsLM` method as implemented in the `R` package `minpack.lm`.

```
nlsFit <- nlsLM(combined$c_PA ~ K1*k2*k3/(2*(kn2+k3))*c_A*(-1+ sqrt(1 + 4*Rhtot/(
↪  K1*(1+k2/(kn2+k3)*c_A) )) )*t,
start = list(K1 = 1e-2, k2 = 1e0, kn2 = 1e-2, k3 = 1e-4),
lower = c(K1 = 0, k2 = 0, kn2 = 0, k3 = 0),
upper = c(K1 = Inf, k2 = Inf, kn2 = Inf, k3 = Inf),
data = combined,
trace = TRUE)
fitParams <- coef(nlsFit)
K1 <- fitParams[1]
k2 <- fitParams[2]
kn2 <- fitParams[3]
k3 <- fitParams[4]
kp <- k2/(kn2+k3)
```

Non-linear regression affords the following parameters:

K_1	7.194×10^{-6}
k_3	5.653×10^{-4}
k'	4.653×10^{3}

The used kinetic constants were then used to simulate concentrations of **PA**. When plotting these predicted concentrations against the measured ones, a decent correlation was found ($y = 0.87x + 0.011 \quad R^2 = 0.96$).

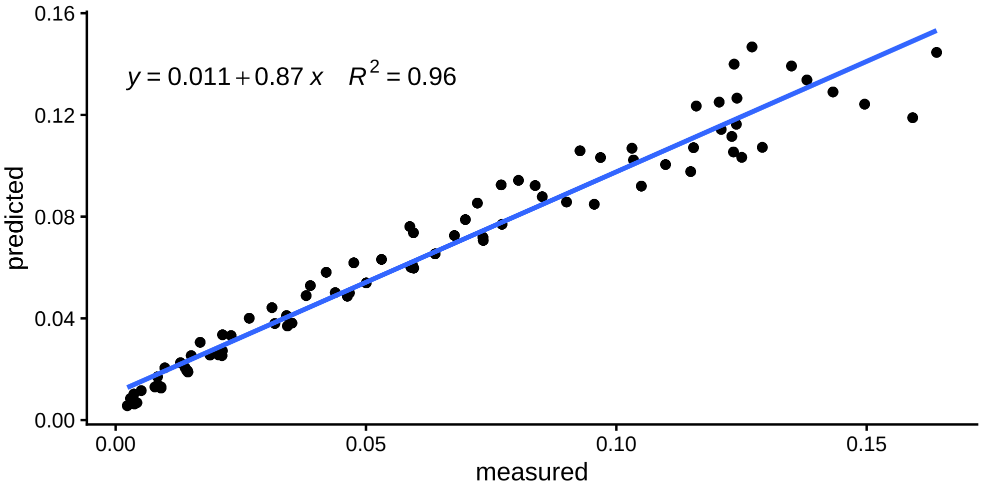

Figure S48: Correlation of predicted and experimental concentration data points of **7 / PA**.

6.10 Determination of Elasticity Coefficient – Order in Catalyst

Based on the rate equation Eq. (59), the expected order in catalyst that can be obtained depending on the value of the different constants can be calculated.[24] This is accomplished by calculating the elasticity coefficient by the following formula:

$$\alpha_\mathrm{i} = \varepsilon^\mathrm{r}_{([\mathrm{A}])} = \frac{\mathrm{dr}}{\mathrm{d[A]}}([\mathrm{A}]_\mathrm{i}) \cdot \frac{[\mathrm{A}]_\mathrm{i}}{\mathrm{r}([\mathrm{A}]_\mathrm{i})} = \frac{\mathrm{d}\ln \mathrm{r}([\mathrm{A}]_\mathrm{i})}{\mathrm{d}\ln[\mathrm{A}]_\mathrm{i}} \tag{64}$$

In the present case this translates to differentiating ln of Eq. (59):

$$\alpha_\mathrm{i} = \frac{\frac{\partial}{\partial \mathrm{x}} \ln(\mathrm{rate})}{\frac{\partial}{\partial \mathrm{x}} \ln x} \tag{65}$$

where $x = [\mathrm{Rh}]_\mathrm{tot}$.

$$\tag{66}$$

$$\alpha_\mathrm{i} = \frac{\frac{\partial}{\partial \mathrm{x}} \ln\left(\frac{K_1 k' k_3}{2}[\mathrm{A}]\left(-1 + \sqrt{\left(1 + \frac{4x}{K_1(k'[\mathrm{A}]+1)}\right)}\right)\right)}{\frac{\partial}{\partial[\mathrm{x}]} \ln x} \tag{67}$$

$$\alpha_\mathrm{i} = \frac{2x}{K_1(k'[\mathrm{A}]+1)\sqrt{\frac{4x}{K_1(k'[\mathrm{A}]+1)}+1}\left(\sqrt{\frac{4x}{K_1(k'[\mathrm{A}]+1)}+1}-1\right)} \tag{68}$$

The following figure shows the order in catalyst as a function of catalyst concentration for different values of the equilibrium constants K_1 assuming that:

$$k' = 4653.4$$
$$\mathrm{A} = 0.3$$

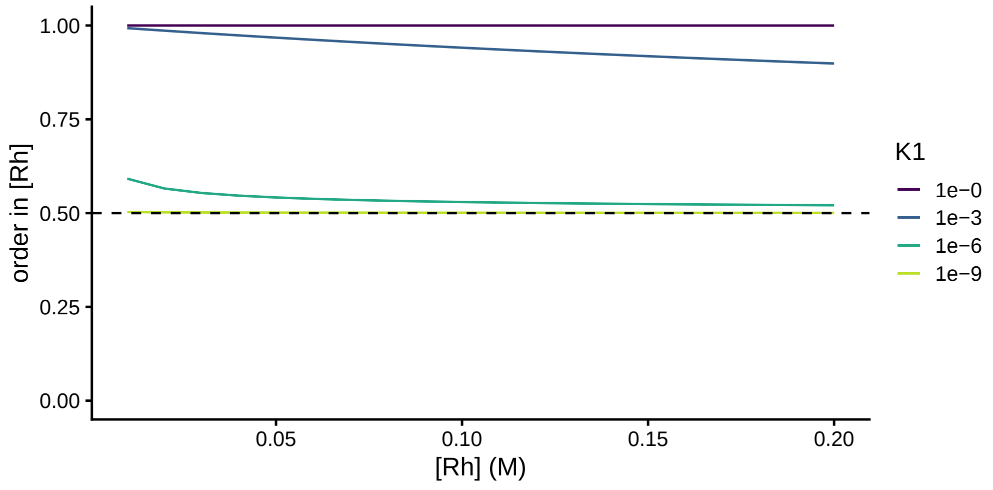

Figure S49: Order in [Rh] as a function of the chloride dissociation equilibrium constant K_1.

As can be seen, the order in catalyst approaches 1 for low catalyst concentrations and higher K_1 and tends asymptotically towards 0.5 for higher catalyst concentrations and lower K_1. This can be explained from the fact that at low catalyst concentrations or large equilibrium constants, species **II** becomes more kinetically relevant or even the resting state, which makes it transform into a catalytic cycle with first order in catalyst.

7 NMR Experiments

7.1 Reaction Monitoring

General Procedure.

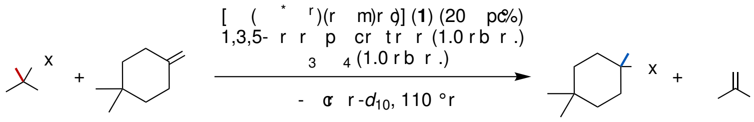

5 6 8 7

A 4 mL-vial under air was charged with **5** (19.5 mg, 0.075 mmol, 1.5 equiv.), **6** (6.3 mg, 0.050 mmol, 1.0 equiv.), 1,3,5-trimethoxybenzene (8.4 mg, 0.050 mmol, 1.0 equiv.) as internal standard, and a magnetic stirring bar and the vial was transferred into a glovebox. The vial was charged with K_3PO_4 (10.6 mg, 0.050 mmol), 1.0 equiv.), Rh complex (0.010 mmol, 0.20 equiv.), and toluene-d_8 (0.5 mL). The mixture was stirred for 5 min at room temperature, then pipetted into a fresh NMR tube. The tube was sealed with a rubber septum, taken out of the glovebox and taped with parafilm.

An initial NMR spectrum was acquired at room temperature, then the reaction mixture was heated inside the spectrometer at 110 °C (in situ) or in an oil bath (ex situ) at the indicated temperature. The upper technical limit of the heating range in the NMR instrument was 110 °C. To prevent boiling of the reaction solvent, *o*-xylene-d_{10} was employed in the in situ experiments. In the ex situ experiments, the reaction mixture was cooled to room temperature at regular time intervals and then measured at 25 °C.

7.1.1 Complex 1

The NMR sample was prepared as outlined in the general procedure (Page S65) with complex [^{12}C]-**1** or [^{13}C]-**1** (11.9 mg, 0.010 mmol, 0.20 equiv.).

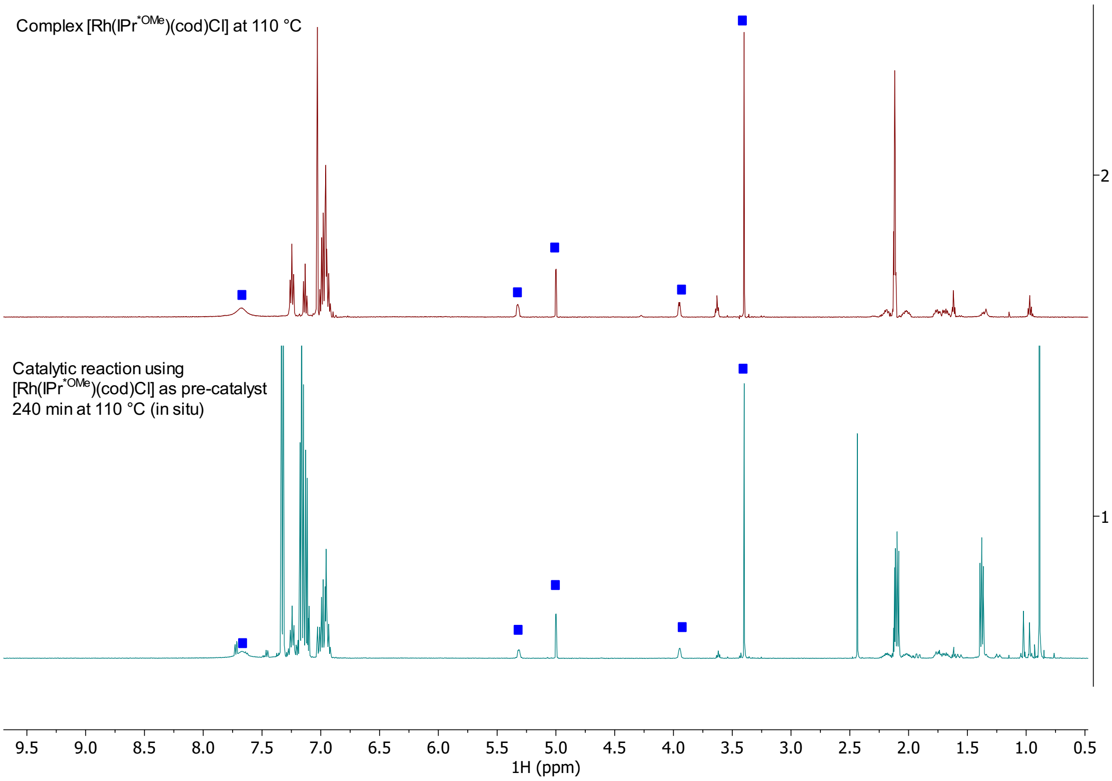

Figure S50: Stacked ^{1}H-NMR-spectra of the in situ catalytic reaction with 20 mol % [^{13}C]-**1** in *o*-xylene-d_{10} measured at 110 °C. Characteristic signals of complex **1** are indicated with blue squares. No change in speciation was observed.

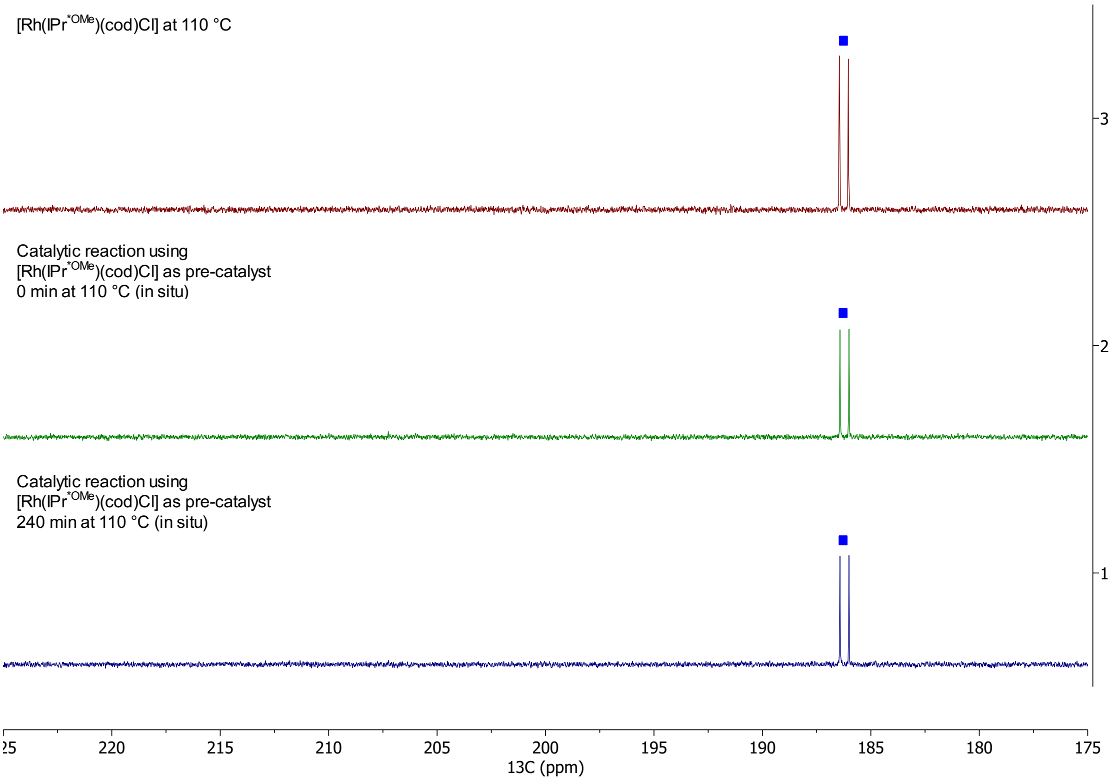

Figure S51: Stacked $^{13}C\{^{1}H\}$-NMR-spectra of the in situ catalytic reaction with 20 mol % [^{13}C]-**1** in *o*-xylene-d_{10} measured at 110 °C. The carbene signal of complex **1** is indicated with a blue square. No change in speciation was observed.

The signal of the catalyst did not change when monitoring the reaction inside the spectrometer at 110 °C for 4 h.

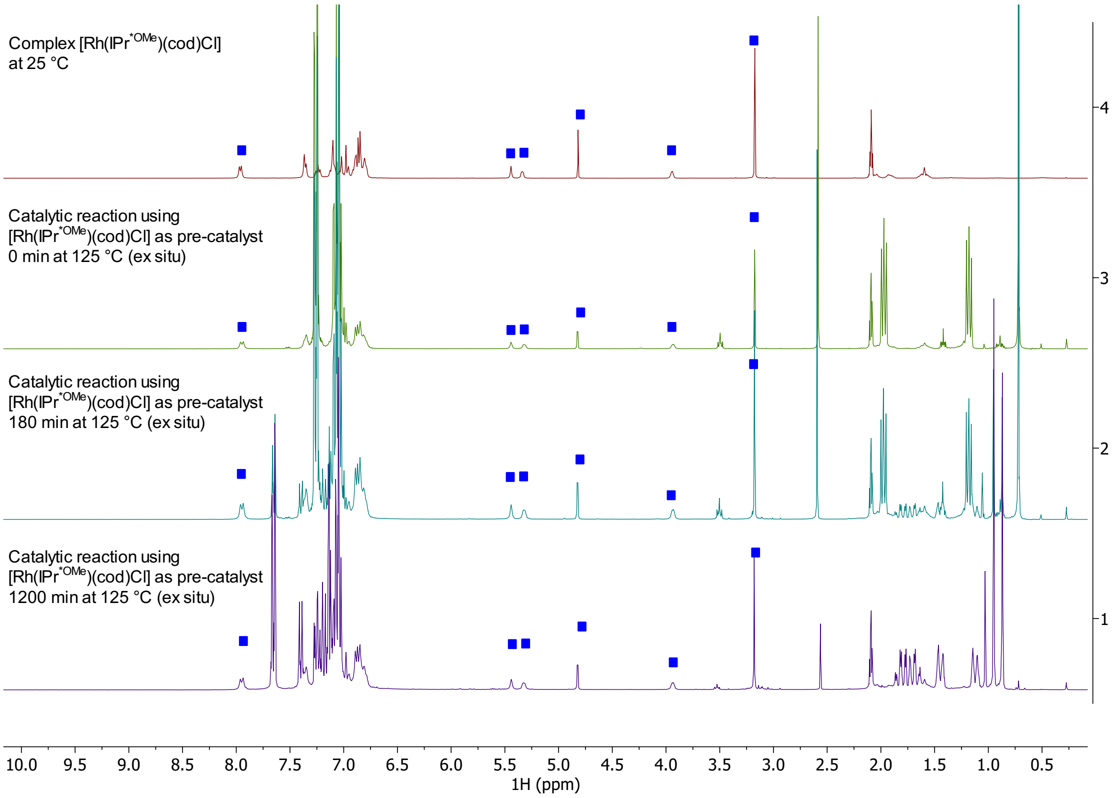

Figure S52: Stacked ^{1}H-NMR-spectra of the ex situ catalytic reaction at 125 °C with 20 mol % [^{13}C]-**1** in toluene-d_8 measured at 25 °C. No change in speciation was observed. Characteristic signals of complex **1** are indicated with blue squares. No change in speciation was observed.

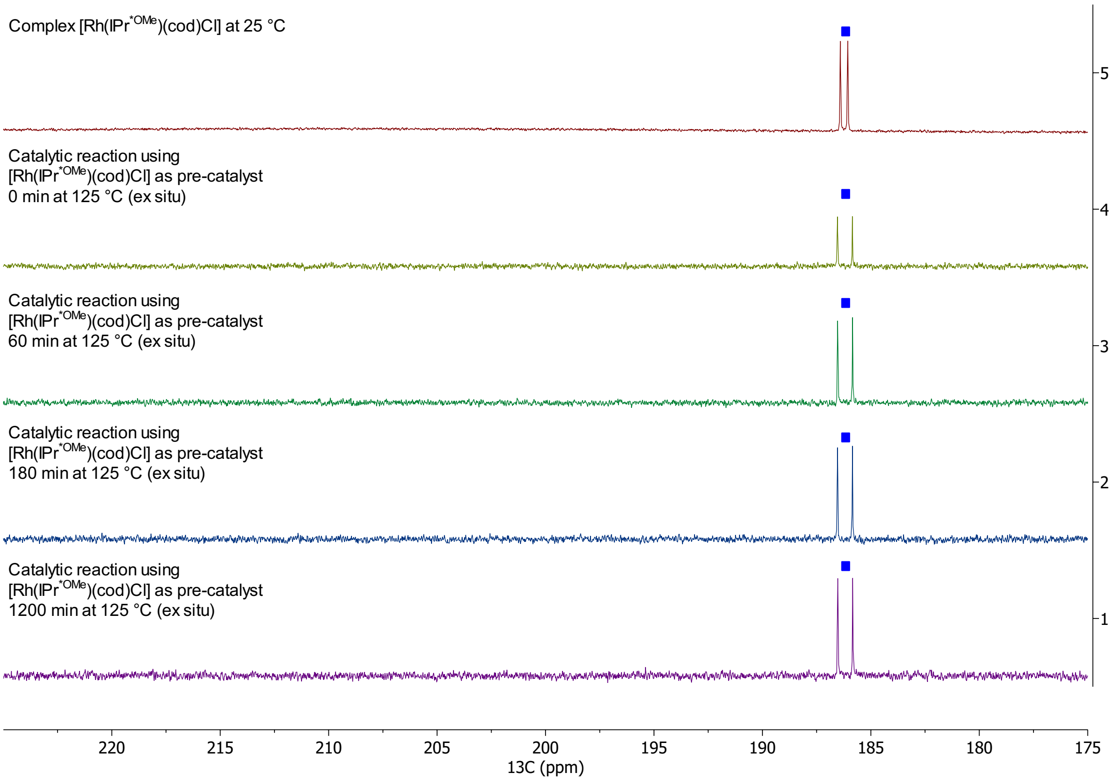

Figure S53: Stacked $^{13}C\{^{1}H\}$-NMR-spectra of the ex situ catalytic reaction with 20 mol % [^{13}C]-**1** in toluene-d_8 measured at 25 °C. The carbene signal of complex **1** is indicated with a blue square. No change in speciation was observed.

The signals of **1** did not change in response to adding substrates, nor when heating the reaction inside the spectrometer for 4 h.

7.1.2 Complex 3

In situ without pre-mixing

The NMR sample was prepared as outlined in the general procedure (Page S65) with complex [^{12}C]-**3** or [^{13}C]-**3** (12.6 mg, 0.010 mmol, 0.20 equiv.).

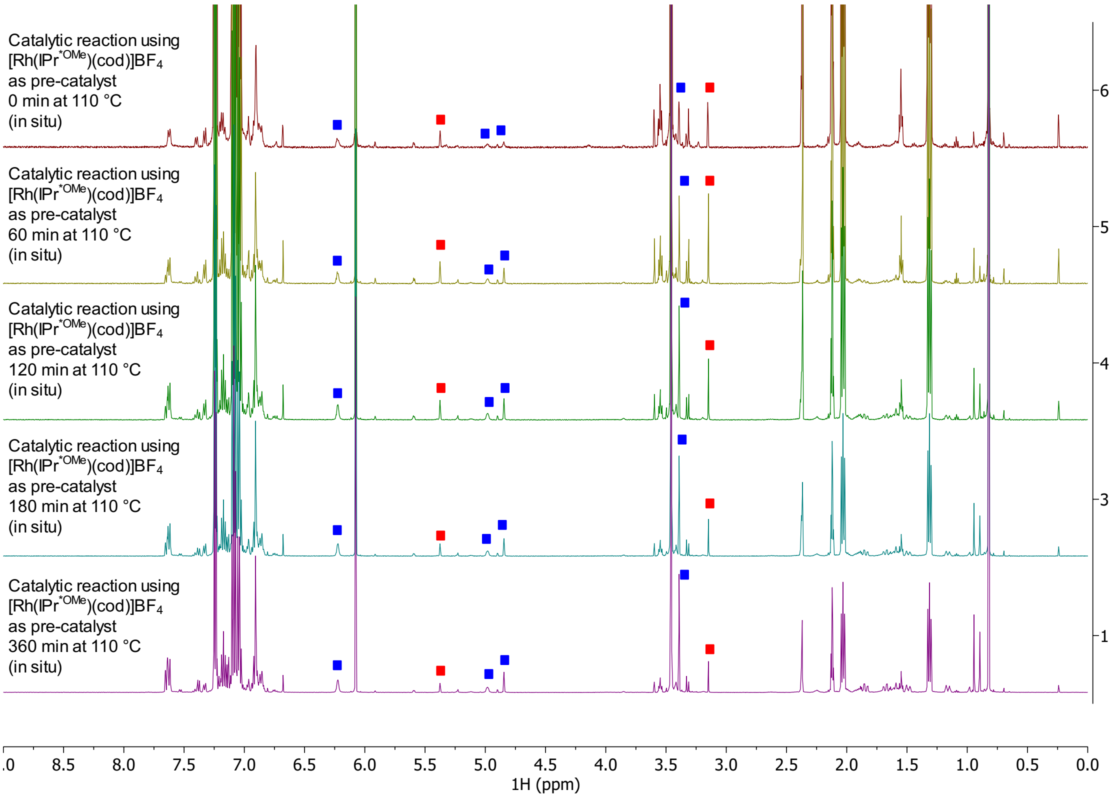

Figure S54: Stacked ^{1}H-NMR-spectra of the in situ catalytic reaction with 20 mol % [^{13}C]-**3** in *o*-xylene-d_{10} measured at 110 °C. Two species are formed upon heating (blue and red squares). The starting complex is not soluble in toluene. Slow dissolution and convection of the complex led to a signal increase over time. Over time, the blue species becomes the dominant species while the other (red squares) decreases.

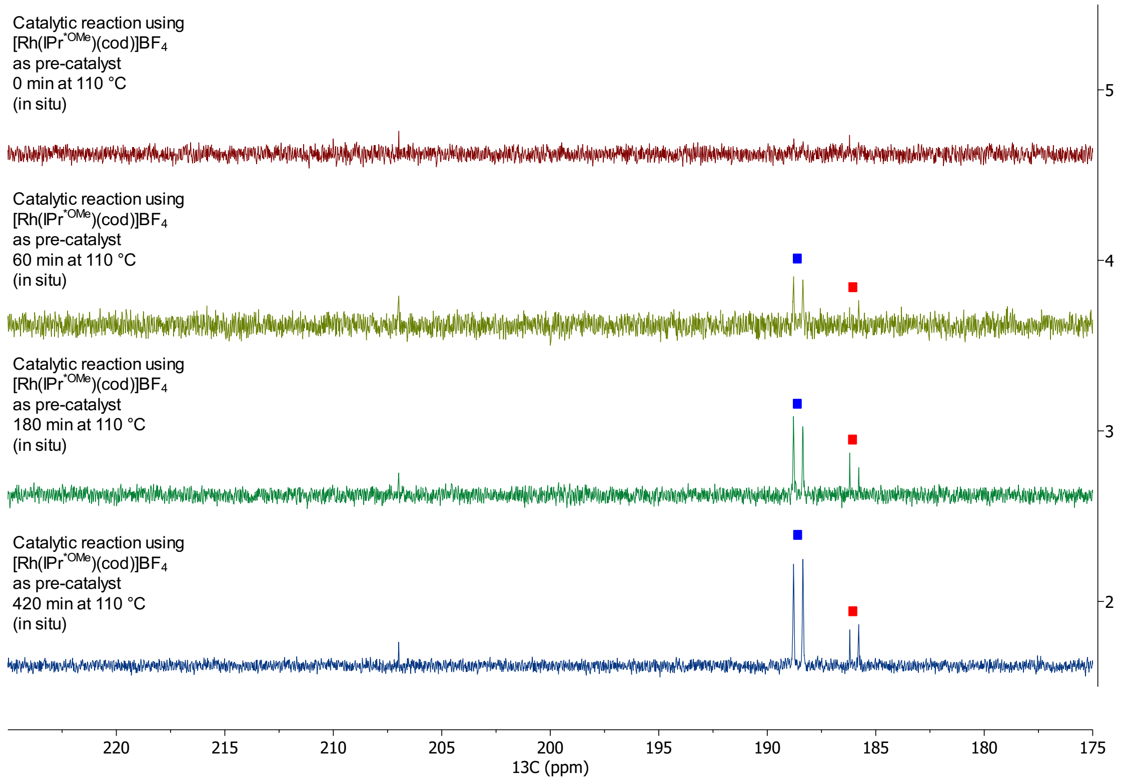

Figure S55: Stacked $^{13}C\{^{1}H\}$-NMR-spectra of the in situ catalytic reaction with 20 mol % [^{13}C]-**3** in *o*-xylene-d_{10} measured at 110 °C. Two species are formed upon heating (blue and red squares). The starting complex is not soluble in toluene. Slow dissolution and convection of the complex led to a signal increase over time. Over time, the blue species becomes the dominant species while the other (red squares) decreases.

In situ with pre-mixing

Because of the slow dissolution of the cationic complex **3** without mixing, the experiment was repeated, with the change that the reaction mixture was briefly heated and stirred at the beginning to ensure dissolution of **3**.

Modified procedure. A 4 mL-vial was charged with all reaction components and solvent as outlined in the general procedure (Page S65). The vial was sealed with a screw cap, taken out of a glovebox and placed in a preheated heating block for 5 min at 110 °C. After cooling to room temperature again, the vial was returned to a glovebox and the contents were pipetted into a fresh NMR tube. The tube was sealed with a rubber septum, taken out of a glovebox and taped with parafilm.

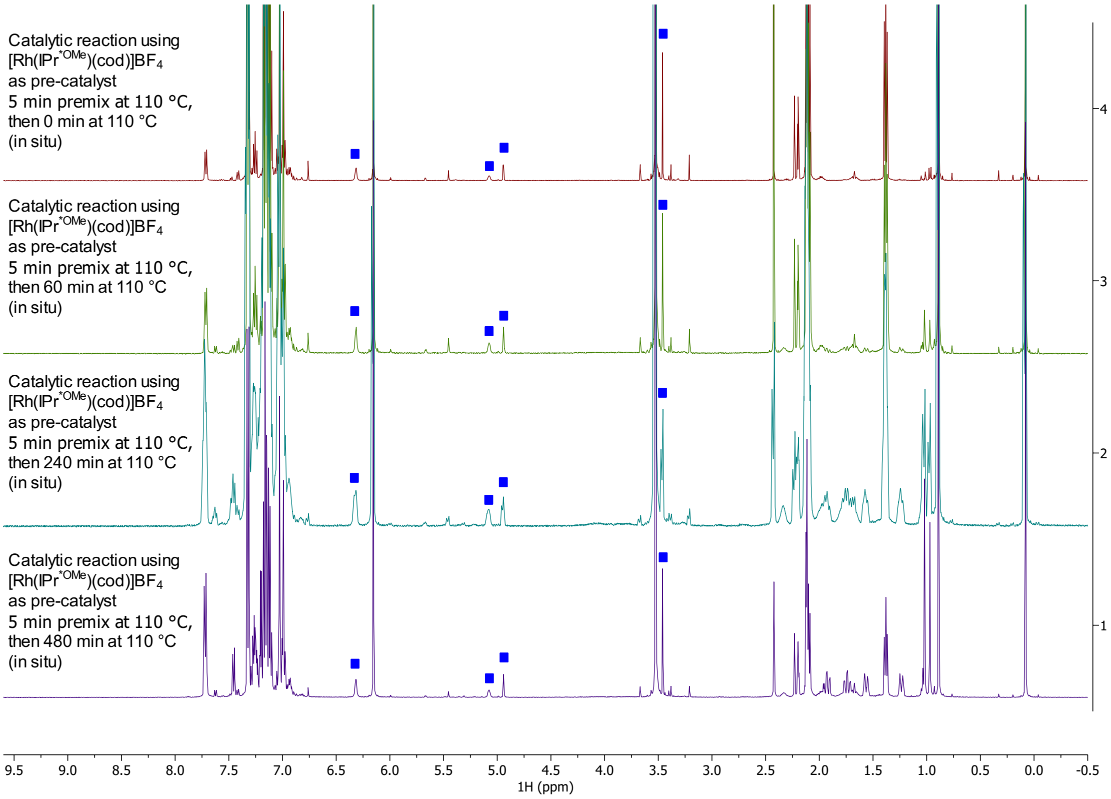

Figure S56: Stacked ^{1}H-NMR-spectra of the in situ catalytic reaction with 20 mol % [^{13}C]-**3** in *o*-xylene-d_{10} measured at 110 °C. A new species is formed upon heating (blue squares) which persists over the course of the reaction.

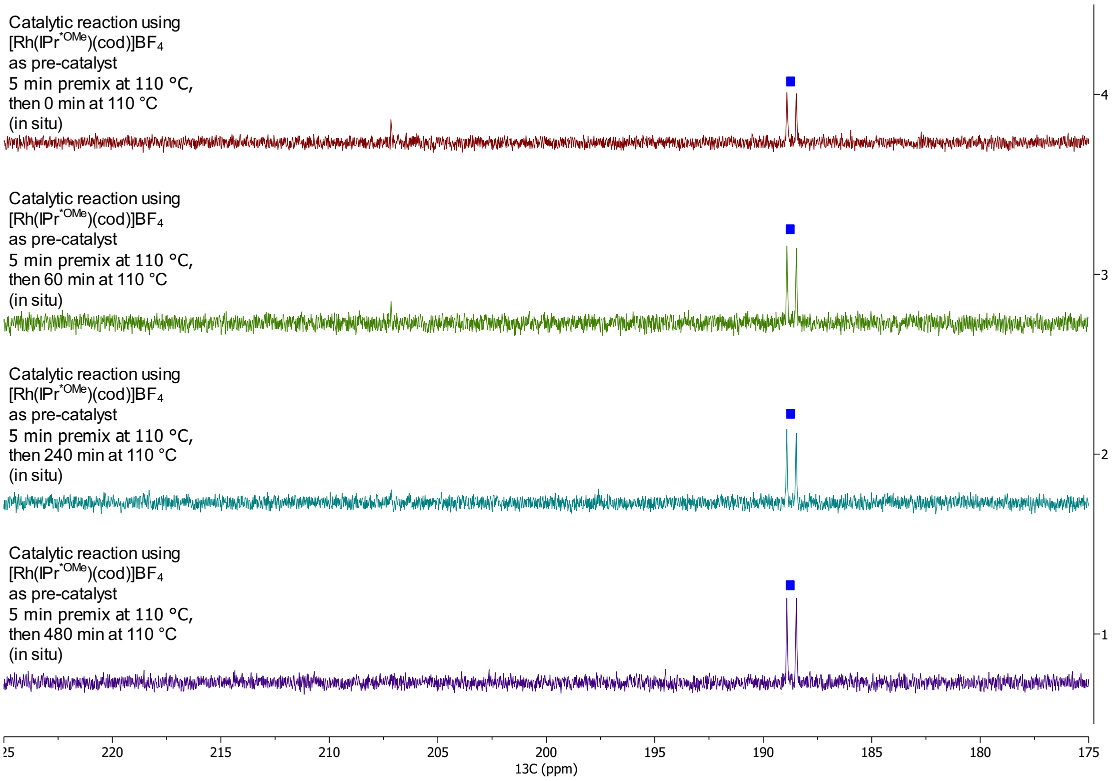

Figure S57: Stacked ^{13}C{^{1}H}-NMR-spectra of the in situ catalytic reaction with 20 mol % [^{13}C]-**3** in *o*-xylene-d_{10} measured at 110 °C. A new species is formed upon heating (blue squares) which persists over the course of the reaction.

ex situ with pre-mixing

Because of the slow dissolution of the cationic complex **3** without mixing, the experiment was repeated, with the change that the reaction mixture was briefly heated and stirred at the beginning to ensure dissolution of **3**.

Modified procedure. A 4 mL-vial was charged with all reaction components and solvent as outlined in the general procedure (Page S65). The vial was sealed with a screw cap, taken out of a glovebox and placed in a preheated heating block for 15 min at 125 °C. After cooling to room temperature again, the vial was returned to a glovebox and the contents were pipetted into a fresh NMR tube. The tube was sealed with a rubber septum, taken out of a glovebox and taped with parafilm.

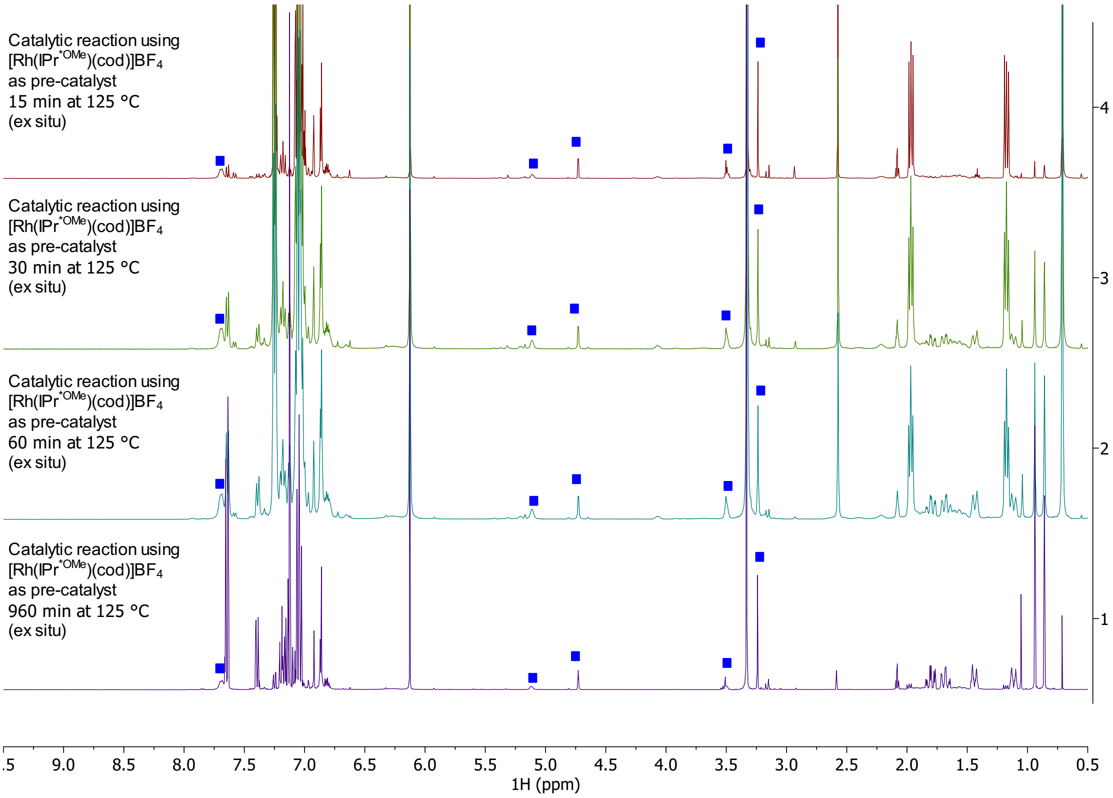

Figure S58: Stacked ^{1}H-NMR-spectra of the ex situ catalytic reaction at 125 °C with 20 mol % [^{13}C]-**3** in toluene-d_8 measured at 25 °C. A new species is formed upon heating (blue squares) which persists over the course of the reaction.

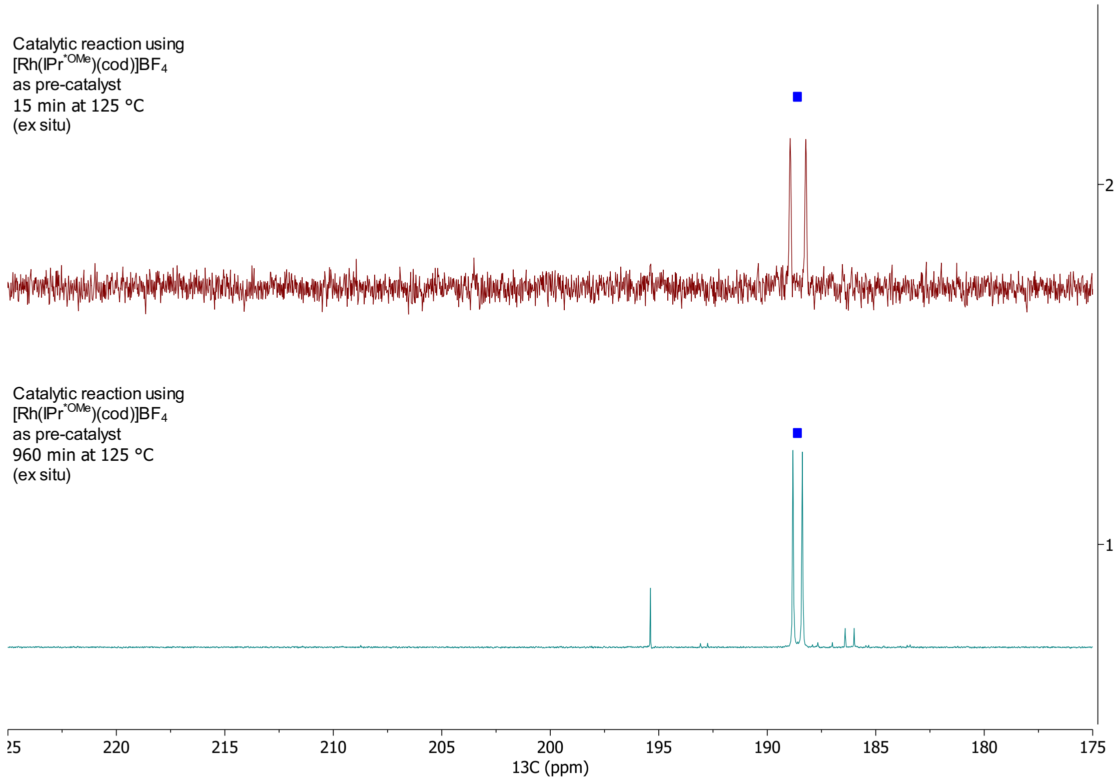

Figure S59: Stacked $^{13}C\{^{1}H\}$-NMR-spectra of the ex situ catalytic reaction at 125 °C with 20 mol % [^{13}C]-**3** in toluene-d_8 measured at 25 °C. A new species is formed upon heating (blue squares) which persists over the course of the reaction.

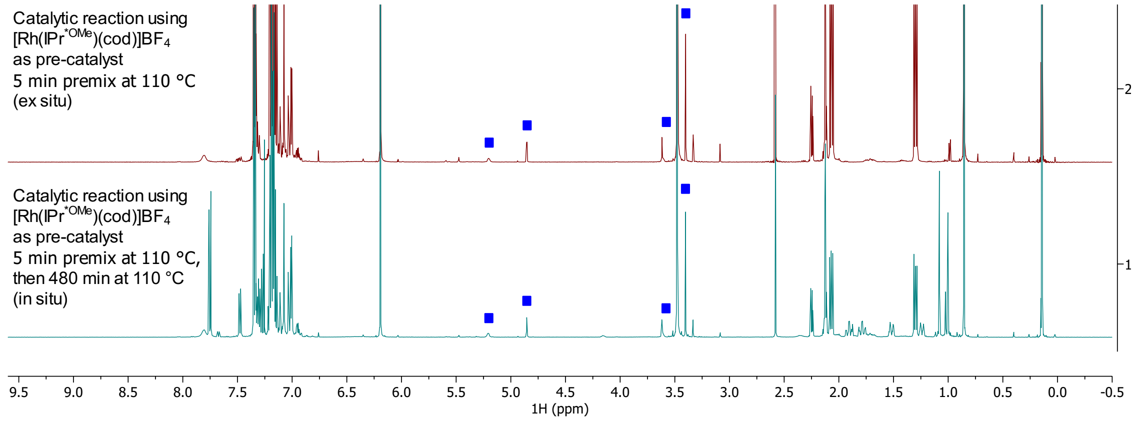

Figure S60: Stacked ^{1}H-NMR-spectra of the in situ catalytic reaction with 20 mol % [^{13}C]-**3** in o-xylene-d_{10} measured at 25 °C after premixing and after 8 h monitoring at 110 °C. The resting state remains stable at room temperature.

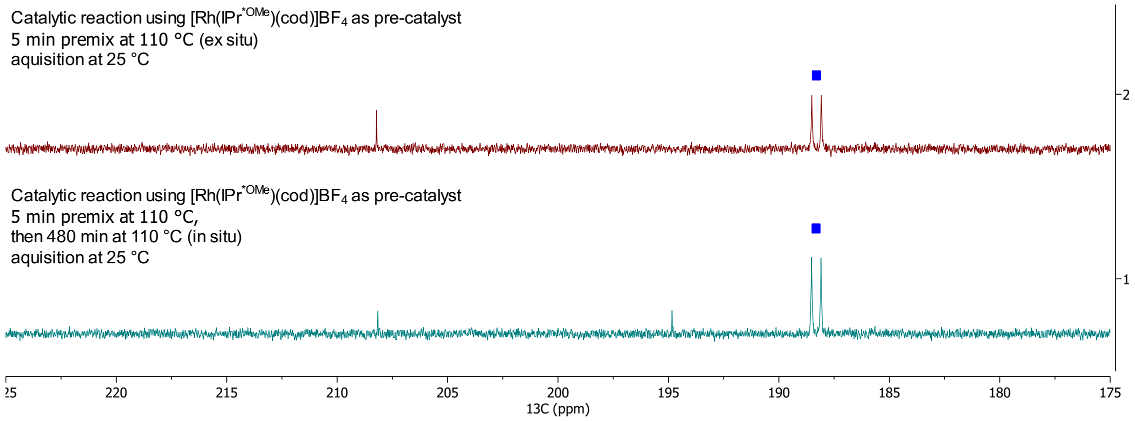

Figure S61: Stacked $^{13}C\{^{1}H\}$-NMR-spectra of the in situ catalytic reaction with 20 mol % [^{13}C]-**3** in *o*-xylene-d_{10} measured at 25 °C after premixing and after 8 h monitoring at 110 °C. The resting state remains stable at room temperature.

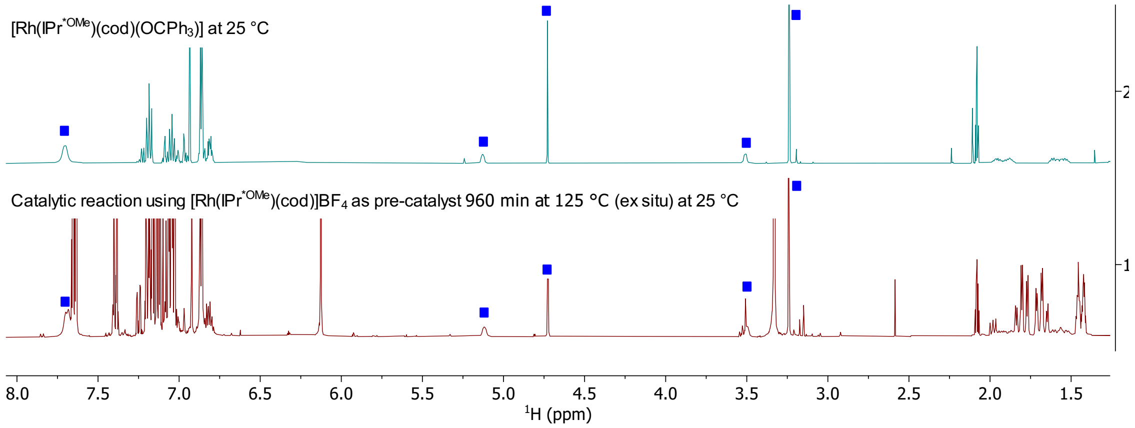

Figure S62: Stacked ^{1}H-NMR-spectra of the ex situ catalytic reaction at 125 °C with 20 mol % [^{13}C]-**3** in toluene-d_8 measured at 25 °C and isolated alkoxide complex **2** (blue squares).

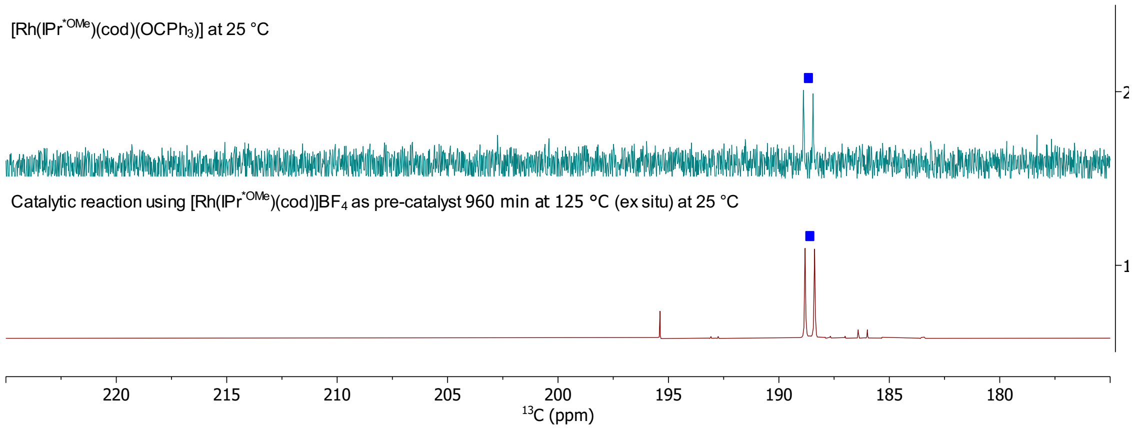

Figure S63: Stacked $^{13}C\{^{1}H\}$-NMR-spectra of the ex situ catalytic reaction at 125 °C with 20 mol % [^{13}C]-**3** in toluene-d_8 measured at 25 °C and isolated alkoxide complex **2** (blue squares).

7.1.3 Complex 3 + Chloride Source

The NMR sample was prepared as outlined in the general procedure (Page S73 with complex [^{12}C]-**3** (12.6 mg, 0.010 mmol, 0.20 equiv.). An initial spectrum was acquired, showing formation of RS-**3**, complex **2** (blue squares).

Then, the NMR sample was returned to the glovebox and added to a vial containing TBACl (3.5 mg, 0.0125 mmol, 0.25 equiv.). The mixture was stirred for 5 min at rt. A color change from red to yellow-orange was observed. The solution with small amounts of precipitate was pipetted into the same NMR tube. The tube was sealed with a rubber septum, taken out of a glovebox and taped with parafilm. A ^{1}H-NMR spectrum was acquired straight after reconstitution and after 2 h at rt. Then, the mixture was heated at for 1–17 h at 125 °C.

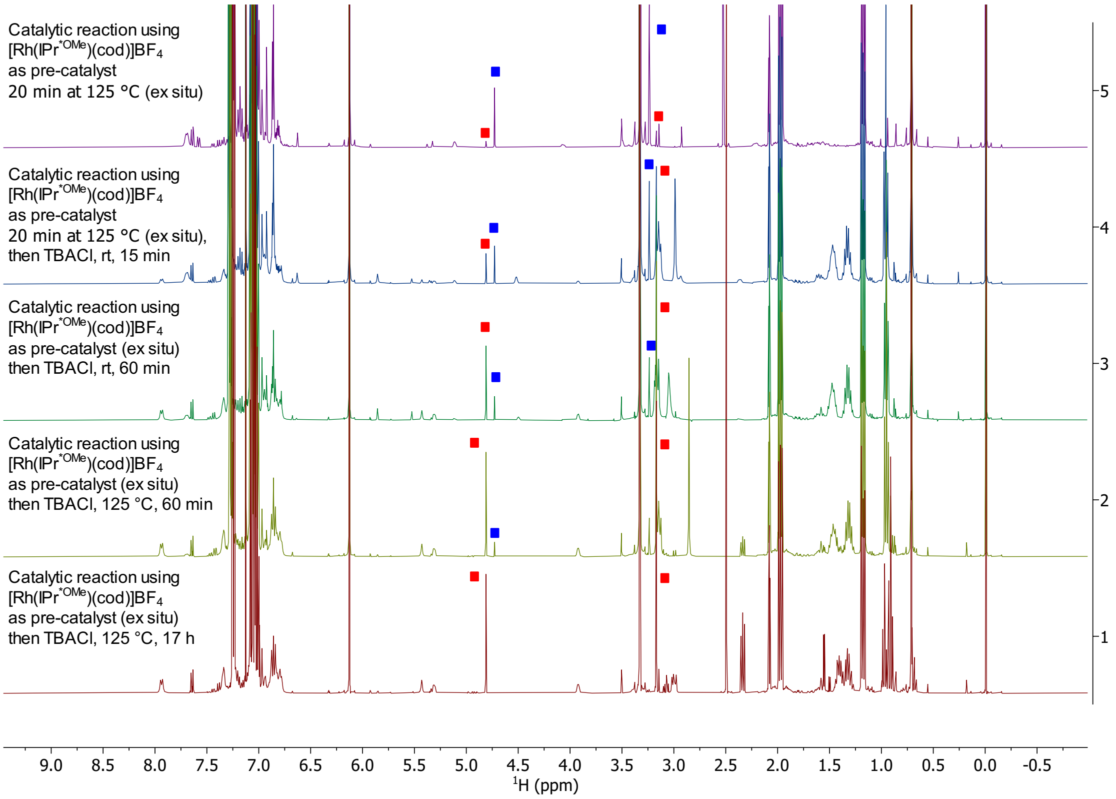

Figure S64: Stacked ^{1}H-NMR-spectra of the ex situ catalytic reaction at 125 °C with 20 mol % [^{12}C]-**3** in toluene-d_8 measured at 25 °C. The initially formed resting state RS-**3**: **2** (blue squares) is converted into RS-**1**: **1** (red squares) upon addition of TBACl.

7.2 DOSY Experiments

7.2.1 General Considerations

Estimated molecular weights (MW) were calculated from the diffusion coefficients established from the ^{1}H DOSY NMR spectrum using the Stejskal-Tanner method.[25]

NMR spectra were processed and the decay was fitted with *GNAT* version 1.3.1.[26] For the calculation of the diffusion constant, the average of multiple non-overlapping peaks was used. To visualize the processed DOSY pseudo-2D spectra *MestreNova* was used.

7.2.2 Molecular Weight Range of the Resting States

A calibration curve was established using six organometallic complexes, including structurally related complexes **1**, **4** and **S1**, at roughly 20 mм in toluene-d_8 at 25 °C (see appendix for DOSY spectra).

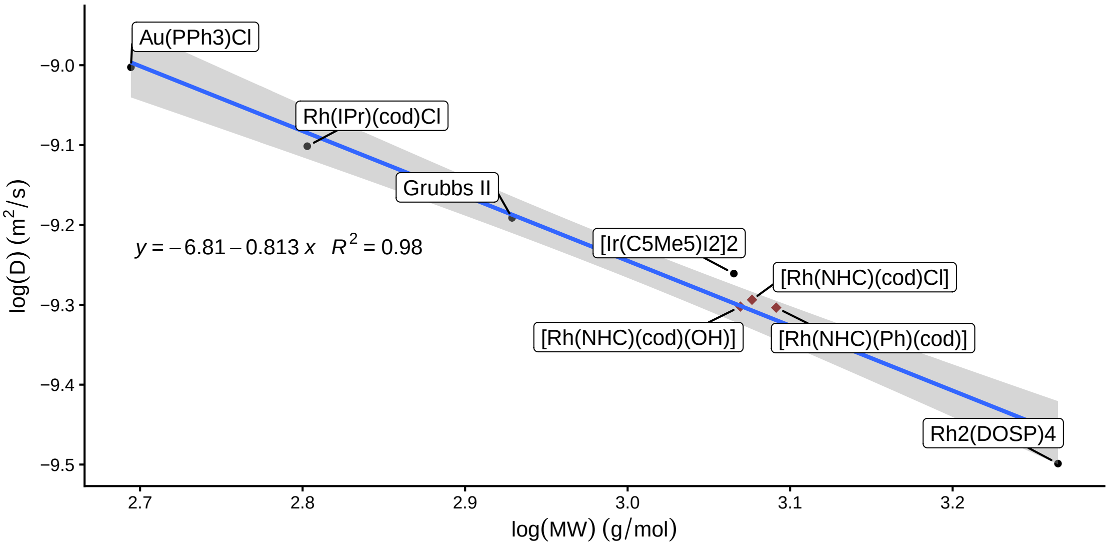

Figure S65: Calibration curve for correlating the diffusion coefficient (D) from DOSY measurements in toluene-d_8 at 25 °C with molecular weights (MW) of characterized organometallic complexes. Grey are corresponds to the standard error of the fit.

The resting states were generated by heating a NMR sample of a catalytic reaction (see Section 7.1) and then measuring a DOSY spectrum under the same conditions as above. As mentioned above, the speciation of complex **1** did not change over the course of the reaction, which was further confirmed by an identical diffusion constant (green horizontal dashed line). In the case of complex **3**, a distinct species was observed (blue horizontal dashed line).

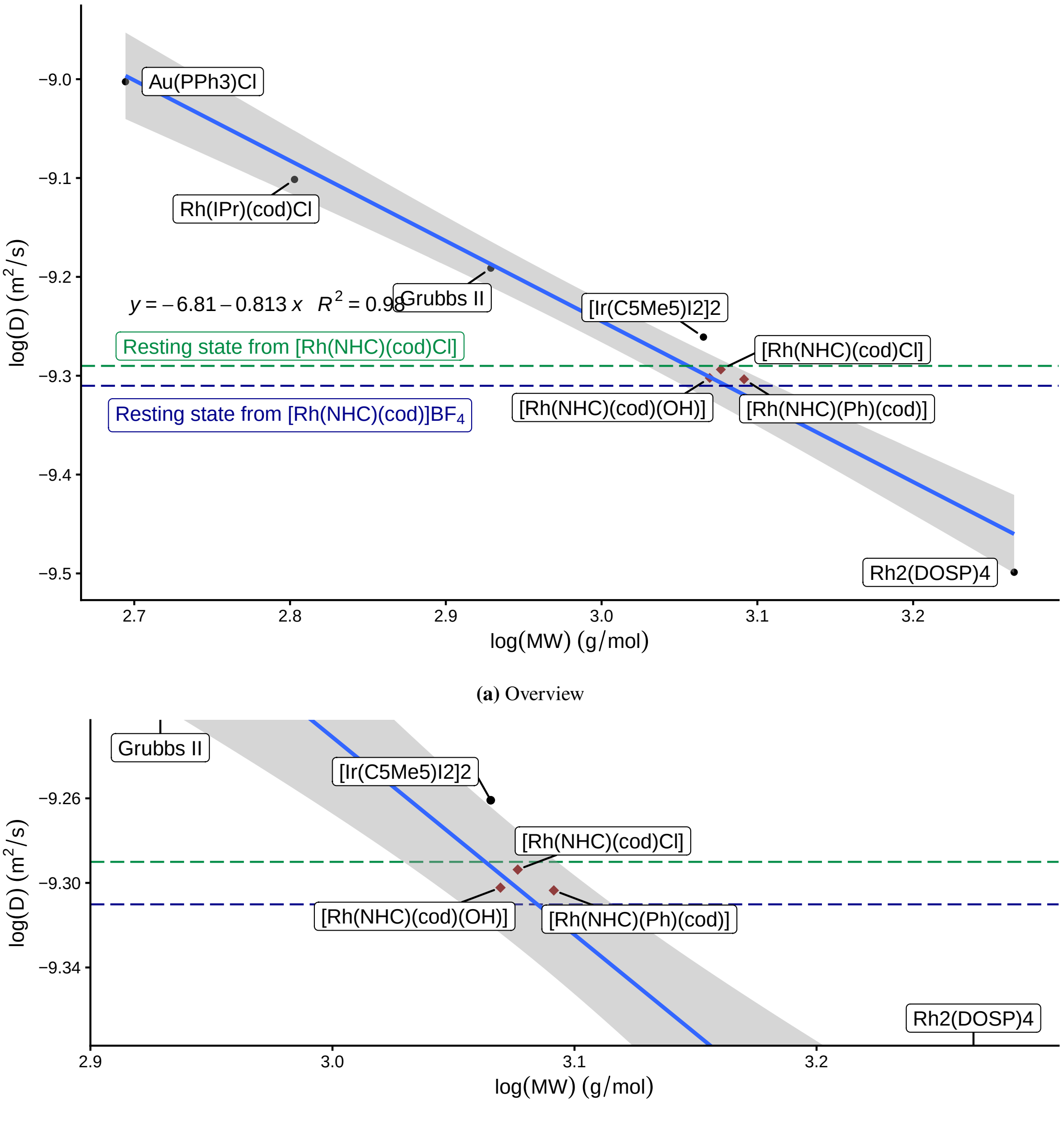

(**a**) Overview

(**b**) Zoom

Figure S66: Plot of log molecular weight (MW) versus log diffusion coefficient (D) in toluene-d_8 at 25 °C. The measured diffusion constants of the resting states are indicated with horizontal dashed lines.

The resting state arising from the cationic complex **3** possesses a comparable hydrodynamic volume (by extension molecular weight) as the known monomeric Rh-complexes, ruling out a dimeric resting state. Additionally, its apparent molecular weight is higher than for the other two resting states, indicative of the substrate-bound complex **2**. We could elucidate its structure to be **2** by comparing the NMR shifts with authentic **2**.

8 Computational Studies

8.1 Computational Details

Conformer searches were conducted with *CREST* version 2.10.2 using default settings.[27] Conformers within a window of 3 kcal mol^{-1} and selected further conformers within a 6 kcal mol^{-1} window were subjected to additional DFT calculations. Additional conformers were added manually if necessary.

All DFT calculations were conducted using the *Gaussian09* suite of programs (Revision D.01).[28] The keyword `integral(grid=ultrafine)` was used in all calculations to limit grid-based errors.[29] Optimizations were conducted at the PBE0 level of theory[30–33] including Grimme's dispersion correction (D3) with Becke-Johnson damping[34,35] and thermal correction to 398 K. Rhodium was modelled with the def2tzvp basis set and corresponding ECP.[36,37] All other atoms were modelled with the def2svp basis set. Ground states were identified by having zero imaginary frequencies in frequency calculations, whereas all reported transition states have exactly one imaginary frequency corresponding to the appropriate reactivity. Thermochemical corrections were calculated at the same level of theory as the optimizations. Quasi-harmonic vibrational corrections[38] were applied by raising all vibrations below 100 cm^{-1} to 100 cm^{-1} using the *GoodVibes* program.[39] Single point energies were calculated with the PBE0 functional including Grimme dispersion correction (D3) with Becke-Johnson damping and the SMD solvent model[40] for toluene. The PBE0 functional was chosen due to its excellent performance in benchmarking studies.[41] All atoms were modelled with the def2tzvp basis set. The corresponding ECP was used for rhodium. Structures were visualized with *CYLview*.[42,43] Buried volume calculations were carried out with *SambVca* 2.1 using standard settings and the structures optimized according to the conditions above (Bondii radii scaled by 1.17, 3.5 sphere radius, H atoms not included).[44] Energy diagrams were created using *EveRplot* (version 1.1).[45]

Input example for conformer sorting, geometry optimizations, and frequency calculations:

```
%nprocshared=8
%mem=16GB
%chk=<name of checkfile>.chk
#p opt freq=noraman pbe1pbe genecp temperature=398 integral(grid=ultrafine)
↪  empiricaldispersion=gd3bj

<title>

0 1
<xyz coordinates>

C H N O 0
def2svp
****
Rh 0
def2tzvp
****

Rh 0
def2tzvp
```

Input example for single point calculations:

```
%nprocshared=8
%mem=16GB
%oldchk=<name of checkfile>.chk
%chk=<name of checkfile>.chk
#p sp geom=allcheck scrf(solvent=toluene,smd) pbe1pbe genecp temperature=398 integral(grid=ultrafine)
↪  empiricaldispersion=gd3bj

C H N O Rh 0
def2tzvp
****
```

```
Rh 0
def2tzvp
```

8.2 Free energy profile

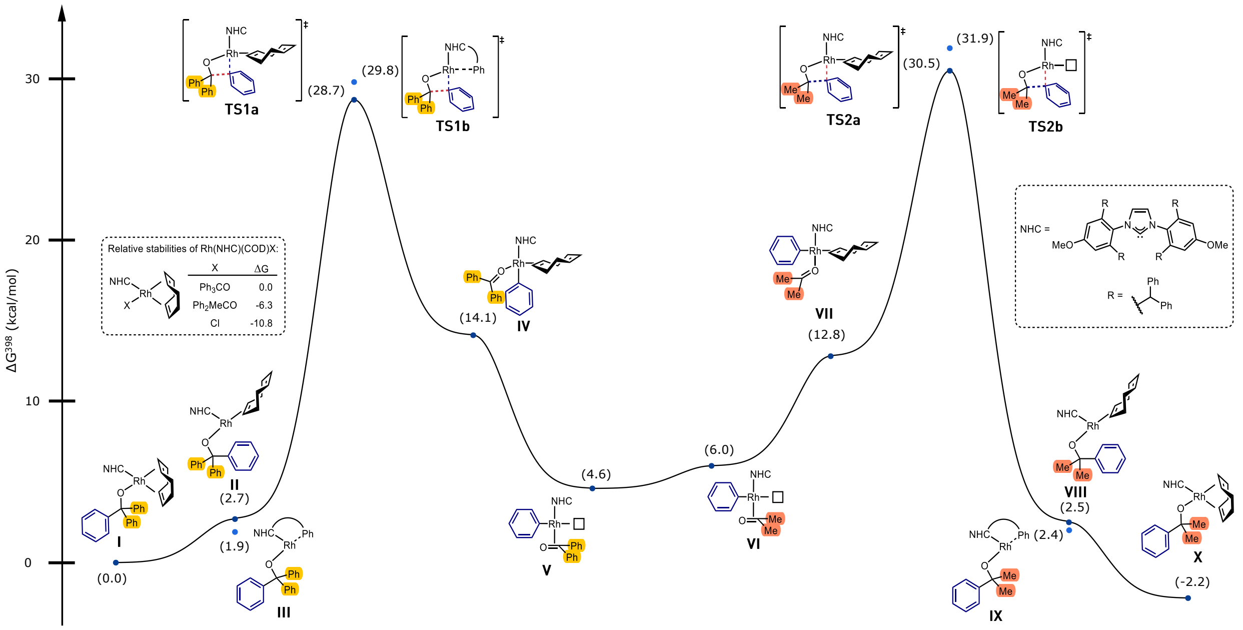

Figure S67: Free energy profile for the rhodium-catalyzed transfer hydroarylation between alcohol **5** and acetone at the PBE0-D3(BJ)/SMD(toluene)-def2tzvp//PBE0-D3(BJ)/def2svp-def2tzvp(Rh) level of theory.

Discussion of the ketone exchange step

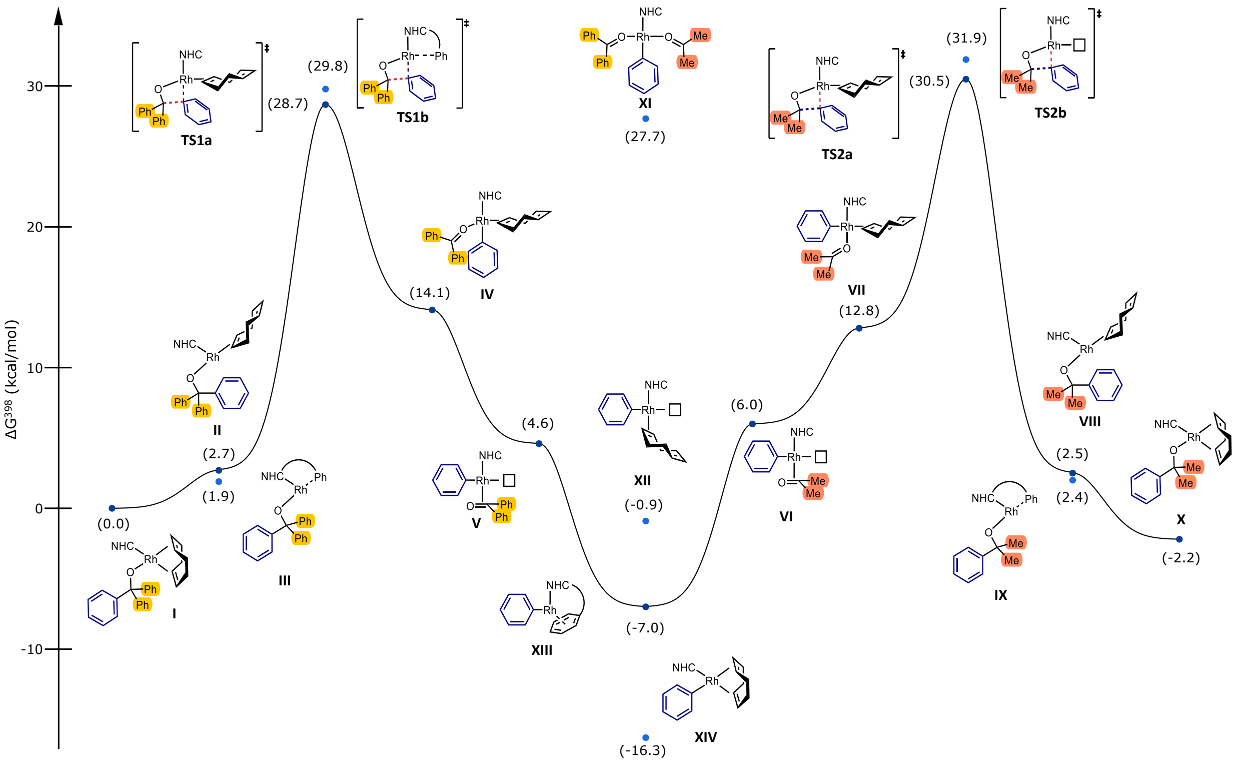

Figure S68: Free energy profile for the rhodium-catalyzed transfer hydroarylation between alcohol **5** and acetone including the ketone exchange step at the PBE0-D3(BJ)/SMD(toluene)-def2tzvp//PBE0-D3(BJ)/def2svp-def2tzvp(Rh) level of theory.

We investigated an associative (via structure **XI**) and a dissociative pathway for the ketone exchange step. The associative pathway is predicted to be high in energy and is unlikely to be catalytically relevant. In the dissociative pathway, different complexes containing the Rh(NHC)(Ph) fragment are accessible that containing different degrees of COD coordination (**XII–XIV**). Notably, **XIII** and **XIV** are calculated to have lower energy than the alkoxide complexes **I** and **X** that were found to be the resting state under chloride-free conditions. We hypothesize that **XIII** and **XIV** might be highly susceptible to proto-demetallation of the Rh–Ph bond by the alcohols present in the reaction, leading to their fast consumption and preventing buildup of these species in the reaction mixture. This is in line with the experimental observation that benzene is formed as a side-product in the reaction. Additionally, **XIV** was quickly consumed when it was used as a pre-catalyst in the reaction. In addition to proto-demetallation as a potential decomposition pathway, the formation of **XIV** might be kinetically hindered. In **XII**, the direct precursor to **XIV** containing a semi-coordinated COD ligand, the free coordination side is cis to the NHC ligand. This is in contrast to other species containing semi-coordinated COD (**II** and **VIII**) where the free coordination site is at the more accessible trans position. The re-organization of the NHC ligand required to open up the free coordination site in **XII** for the coordination of the second alkene moiety of the COD ligand might be associated with a significant penalty that is hard to track by DFT studies.

8.3 Ligand flexibility analysis

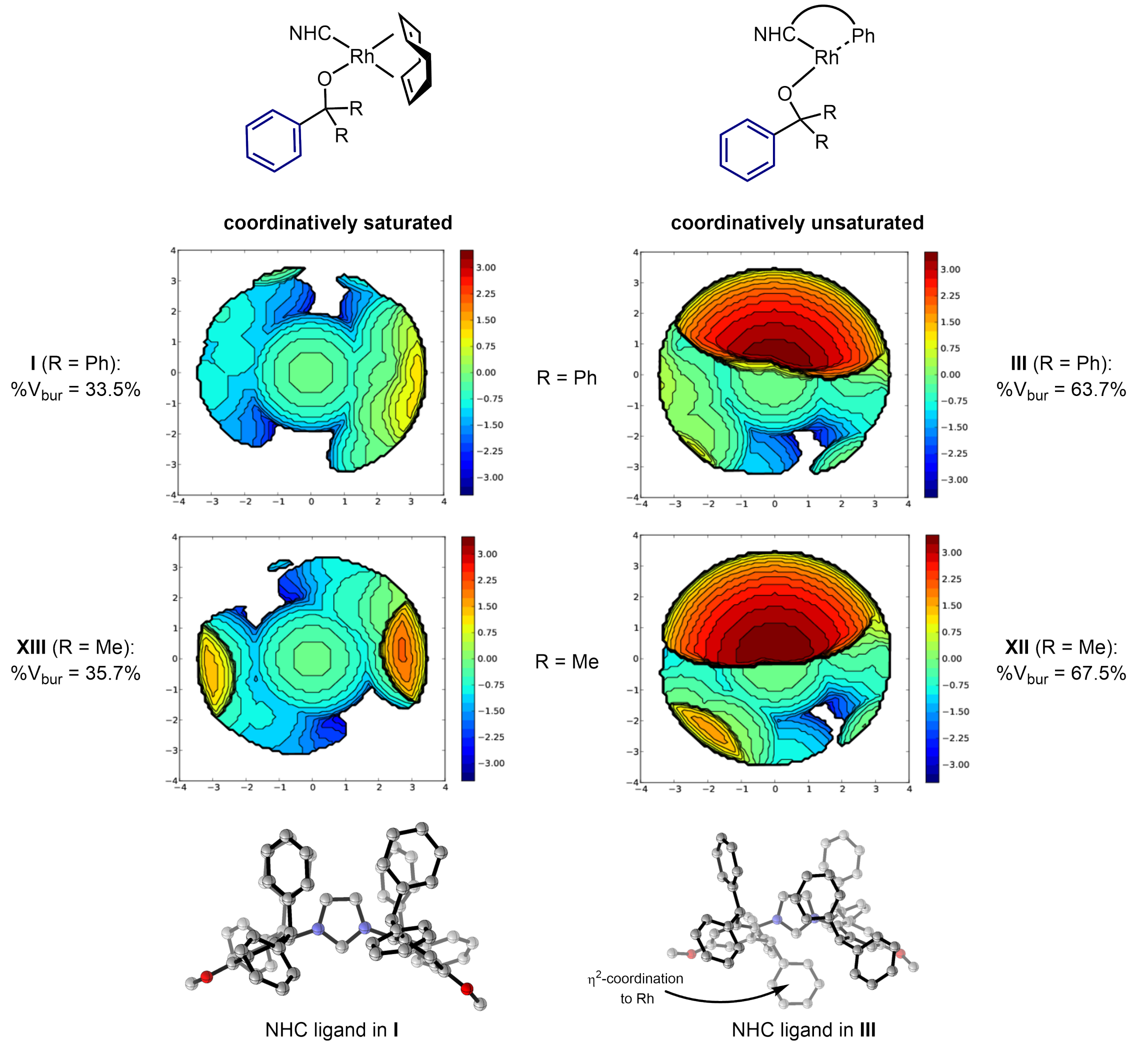

Figure S69: Buried volume analysis for different complexes.

The large NHC ligand can stabilize the complex by adapting its size buried volume to the size of bound ligands (**I** vs. **XIII** and **III** vs. **XII**). In case of low-coordinate complexes such as **III** and **XII**, the peripheral phenyl groups of the ligand can coordinate to the Rh for additional stabilization, resulting in a drastically increased buried volume compared to coordinatively saturated complexes (**I** vs. **III** and **XIII** vs. **XII**).

To gain further insights into the stabilization by the peripheral phenyl groups of the ligands, we conducted calculations using truncated complexes in which the peripheral diphenylmethyl groups of the ligand were either partially or fully replaced by methyl groups (Fig. S70). Using these species, we recalculated the energy required for the decoordination of COD from Rh(NHC)(COD)(alkoxide) complexes (**I**, **XV–XVI**) and the barrier for the β-carbon elimination from the resulting COD-free complexes. Removing all peripheral groups except for the one coordinating to the rhodium atom in the Rh(NHC)(alkoxide) complex (complex **XVIII**) led to a destabilization of ca. 10 kcal/mol compared to the complex containing all peripheral groups (**III**). This destabilization is similar in the corresponding transition state **TS4**, showing that the peripheral substituents stabilize the complexes by dispersive interactions. Removing all peripheral groups including the one coordinated to the rhodium in the Rh(NHC)(alkoxide) complex (**XVII**) leads to an additional

destabilization of 10 kcal/mol compared to complex **XVI**. Again, this effect was preserved in the corresponding transition state **TS3**, showing that the rhodium–aryl interaction also leads to strong complex stabilization.

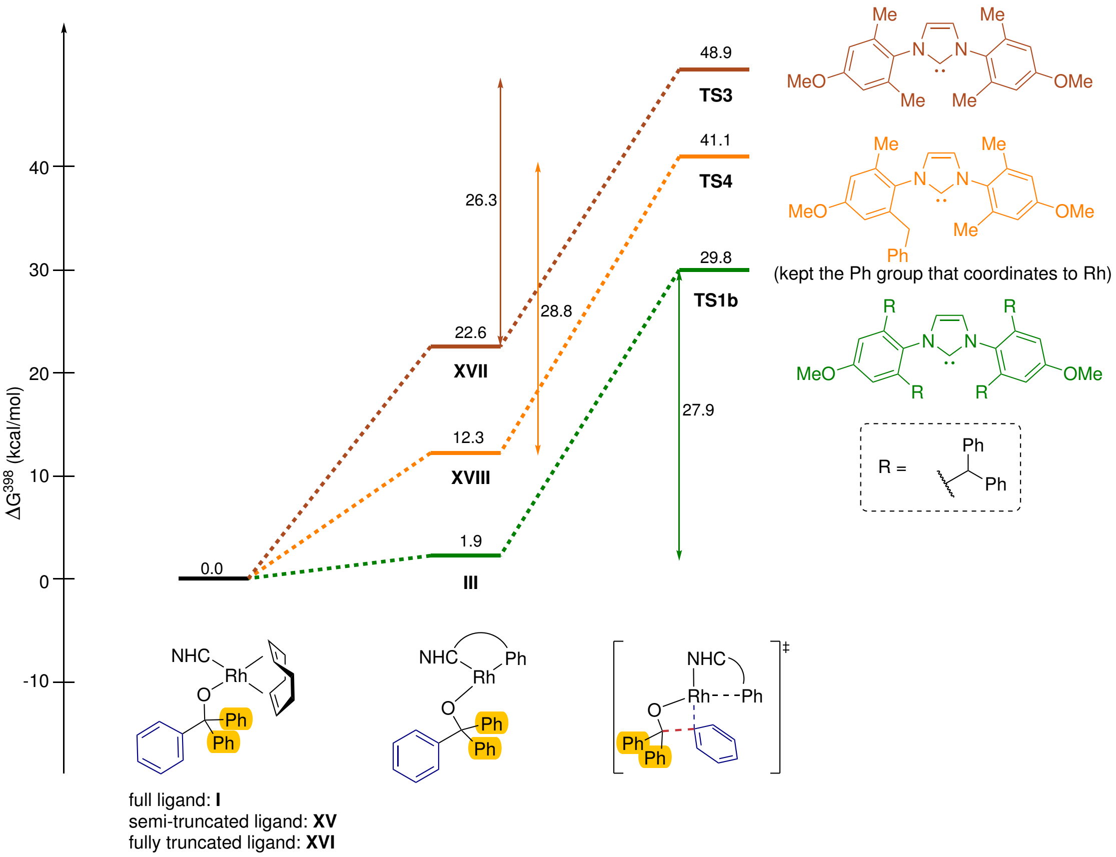

Figure S70: Investigation of the effect of the peripheral phenyl groups of the NHC ligand.

The peripheral substituents of the NHC not only stabilize the complex by intra-ligand interactions, but also by interactions with other ligands. For instance, an edge-face interaction between a peripheral ligand phenyl group and an alkoxide phenyl group is visible in the most favorable geometry of complex **III** (Fig. S71).

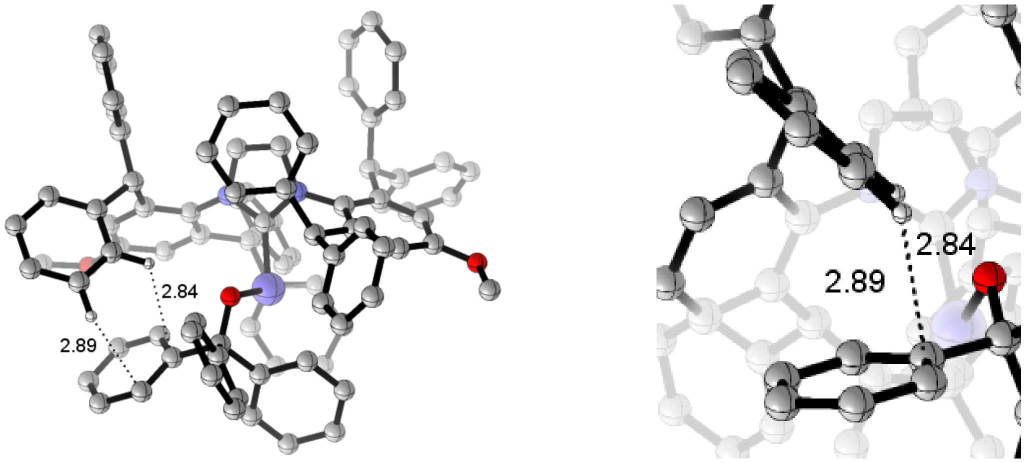

(a) Full view. **(b)** Zoom in of the structural region with the interaction.

Figure S71: Dispersive interactions between a peripheral ligand phenyl group and an alkoxide phenyl group in complex **III**.

8.4 Thermodynamics of Ion Exchange

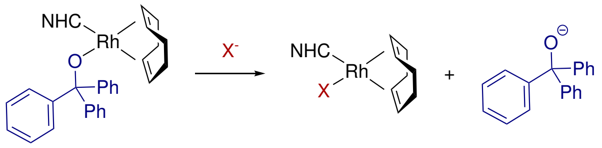

Figure S72: Thermodynamics of ion exchange processes.

X	ΔG
chloride (**XIX**)	−10.8
Ph_2MeCO^- (**X**)	−6.3
hydroxide (**XX**)	−43.6

The analysis shows that the chloride containing complex **XIX** is lower in energy than alkoxide complexes and also lower than the intermediates in the catalytic cycle. This is consistent with the experimental data indicating that chloride complex **1** (structure **XIX**) is the resting state of the reaction.

8.5 Summary of Calculated Energy Values

Table S20: Summary of calculated energy values. E_{SPC}: electronic energy of the single point calculation. E: electronic energy of the optimization. ZPE: zero-point energy correction. H_{SPC}: enthalpy of the single point calculation. T.S: entropy term. T.qh-S: entropy term with quasi-harmonic energy correction. $G(T)_{SPC}$: free energy without quasi-harmonic correction, calculated as $[G(T)_{SPC}] = [H_{SPC}] – [T.S]$. qh-$G(T)_{SPC}$: free energy with quasi-harmonic correction, calculated as [qh-$G(T)_{SPC}$] = $[H_{SPC}]$ – [T.qh-S]. All values in Hartree.

Structure	E_{SPC}	E	ZPE	H_{SPC}	T.S	T.qh-Se	$G(T)_{SPC}$	qh-$G(T)_{SPC}$
Cl^-	−460.183 07	−459.928 07	0.000 00	−460.179 92	0.024 12	0.024 12	−460.204 03	−460.204 03
COD	−311.780 78	−311.446 80	0.180 98	−311.585 55	0.059 93	0.059 71	−311.645 47	−311.645 26
Me_2CO	−193.014 50	−192.791 06	0.083 65	−192.921 10	0.051 15	0.049 24	−192.972 26	−192.970 34
OH^-	−75.790 19	−75.579 82	0.008 04	−75.777 73	0.027 42	0.027 42	−75.805 16	−75.805 16
Ph_2CO	−576.197 29	−575.572 40	0.193 21	−575.984 24	0.075 16	0.073 38	−576.059 40	−576.057 62
Ph_3CO^-	−807.719 43	−806.812 75	0.282 21	−807.408 59	0.094 80	0.090 76	−807.503 40	−807.499 35
$PhMe_2CO^-$	−424.530 10	−424.012 10	0.174 08	−424.338 55	0.067 43	0.066 90	−424.405 97	−424.405 45
**I**	−4150.937 32	−4146.691 90	1.541 23	−4149.245 37	0.355 96	0.323 63	−4149.601 33	−4149.568 99
**II**	−4150.932 15	−4146.681 96	1.541 68	−4149.238 88	0.360 52	0.325 73	−4149.599 40	−4149.564 61
**III**	−3839.117 07	−3835.189 80	1.356 81	−3837.623 49	0.331 24	0.297 24	−3837.954 72	−3837.920 73
**IV**	−4150.910 51	−4146.659 51	1.539 46	−4149.218 36	0.364 78	0.328 14	−4149.583 14	−4149.546 50
**V**	−3839.109 71	−3835.176 18	1.355 73	−3837.616 10	0.335 30	0.300 28	−3837.951 40	−3837.916 38
**VI**	−3455.919 16	−3452.382 52	1.246 06	−3454.545 76	0.314 04	0.281 15	−3454.859 80	−3454.826 90
**VII**	−3767.723 83	−3763.870 28	1.430 17	−3766.151 28	0.345 56	0.310 01	−3766.496 84	−3766.461 29
**VIII**	−3767.747 31	−3763.893 27	1.433 19	−3766.174 00	0.339 87	0.303 74	−3766.513 87	−3766.477 73
**IX**	−3455.931 36	−3452.399 19	1.249 56	−3454.556 37	0.306 42	0.276 31	−3454.862 79	−3454.832 68
**X**	−3767.758 39	−3763.907 50	1.435 07	−3766.184 06	0.335 95	0.301 10	−3766.520 01	−3766.485 16
**XI**	−4032.119 72	−4027.978 30	1.442 04	−4030.529 69	0.357 83	0.320 22	−4030.887 52	−4030.849 91
**XII**	−3574.697 62	−3571.046 53	1.342 71	−3573.222 12	0.324 76	0.290 73	−3573.546 88	−3573.512 85
**XIII**	−3262.893 03	−3259.566 71	1.159 53	−3261.616 25	0.288 79	0.261 06	−3261.905 05	−3261.877 31
**XIV**	−3574.727 11	−3571.083 43	1.345 14	−3573.250 88	0.318 20	0.286 53	−3573.569 08	−3573.537 41
**XV**	−2534.826 82	−2532.256 87	0.966 09	−2533.764 91	0.239 83	0.221 63	−2534.004 74	−2533.986 54
**XVI**	−2303.950 07	−2301.620 73	0.883 54	−2302.978 46	0.221 65	0.207 21	−2303.200 11	−2303.185 66
**XVII**	−1992.097 28	−1990.086 07	0.698 12	−1991.325 88	0.190 98	0.178 54	−1991.516 85	−1991.504 41
**XVIII**	−2222.990 18	−2220.740 97	0.781 32	−2222.127 33	0.211 17	0.194 43	−2222.338 51	−2222.321 76
**XIX**	−3803.396 25	−3799.838 23	1.255 75	−3802.015 85	0.307 26	0.275 05	−3802.323 11	−3802.290 90
**XX**	−3419.061 76	−3415.568 62	1.267 15	−3417.669 61	0.306 85	0.274 64	−3417.976 46	−3417.944 25

Table S21: Summary of calculated energy values. E_{SPC}: electronic energy of the single point calculation (continued). E: electronic energy of the optimization. ZPE: zero-point energy correction. H_{SPC}: enthalpy of the single point calculation. T.S: entropy term. T.qh-S: entropy term with quasi-harmonic energy correction. $G(T)_{SPC}$: free energy without quasi-harmonic correction, calculated as $[G(T)_{SPC}] = [H_{SPC}] - [T.S]$. qh-$G(T)_{SPC}$: free energy with quasi-harmonic correction, calculated as [qh-$G(T)_{SPC}$] = $[H_{SPC}]$ – [T.qh-S]. All values in Hartree.

Structure	E_{SPC}	E	ZPE	H_{SPC}	T.S	T.qh-Se	$G(T)_{SPC}$	qh-$G(T)_{SPC}$
**TS1a**	−4150.889 19	−4146.640 74	1.539 51	−4149.198 46	0.359 23	0.324 85	−4149.557 69	−4149.523 31
**TS1b**	−3839.069 89	−3835.142 04	1.354 07	−3837.579 09	0.330 92	0.297 12	−3837.910 01	−3837.876 21
**TS2a**	−3767.699 67	−3763.845 73	1.430 80	−3766.128 32	0.341 23	0.304 71	−3766.469 56	−3766.433 03
**TS2b**	−3455.880 38	−3452.346 66	1.245 92	−3454.508 50	0.310 84	0.277 15	−3454.819 34	−3454.785 65
**TS3**	−1992.050 67	−1990.037 41	0.694 99	−1991.281 92	0.194 35	0.180 60	−1991.476 26	−1991.462 51
**TS4**	−2222.941 30	−2220.691 88	0.778 68	−2222.081 18	0.210 21	0.194 65	−2222.291 39	−2222.275 83

9 Improved Protocol for the Shuttle Arylation

Table S22: Catalytic competence of Rh complexes at 110 °C.

Entry[a]	Complex	Time (h)	Yield **7** (%)[b]	Yield **8** (%)[b]
1	[Rh(**L1**)(COD)Cl] (**1**)	24	19	18
2	[Rh(**L1**)(COD)Cl] (**1**)	48	39	38
3	[Rh(**L1**)(COD)(OH)] (**S1**)	24	10	10
4	[Rh(**L1**)(COD)(OH)] (**S1**)	48	24	23
5	[Rh(**L1**)(COD)]BF_4 (**3**)	24	39	39
6	[Rh(**L1**)(COD)]BF_4 (**3**)	48	86	86
7	[Rh(**L1**)(Ph)(COD)] (**4**)	24	30	26
8	[Rh(**L1**)(Ph)(COD)] (**4**)	48	58	54

[a] General conditions: **5** (0.150 M), **6** (0.100 M), K_3PO_4 (0.100 M), [Rh] (0.005 M), *n*-dodecane (0.097 M), 110 °C, 24 h.
[b] Product yield was determined by GC analysis.

Table S23: Assessment of long term catalyst stability.

Entry[a]	Complex	Storage	Yield **7** (%)[b]	Yield **8** (%)[b]
1	[Rh(**L1**)(COD)]BF_4 (**3**)	Ar, rt	92	117
2	[Rh(**L1**)(COD)]BF_4 (**3**)	air, 7 months[c]	87	91
3	[Rh(**L1**)(Ph)(COD)] (**4**)	Ar, rt	80	83
4	[Rh(**L1**)(Ph)(COD)] (**4**)	air, 7 months[c]	100	113

[a] General conditions: **5** (0.150 M), **6** (0.100 M), K_3PO_4 (0.100 M), [Rh] (0.005 M), *n*-dodecane (0.097 M), 125 °C, 24 h.
[b] Product yield was determined by GC analysis.
[c] Bottled under air and kept in a closed vial under ambient atmosphere.

- Cationic complex **3** shows the highest activity, signified by full conversion of starting material at 110 °C after 48 h, in contrast to the other complexes.
- The most active complexes (**3** and **4**) are air-stable for a long time and therefore suited as precatalysts.
- While catalyst turnover is achievable with catalytic amounts of base, a stoichiometric amount is optimal for high yields when using **3**. In contrast, **4** is already efficient at catalytic amounts of base.

The new mechanism-informed optimized reaction conditions are as follows:

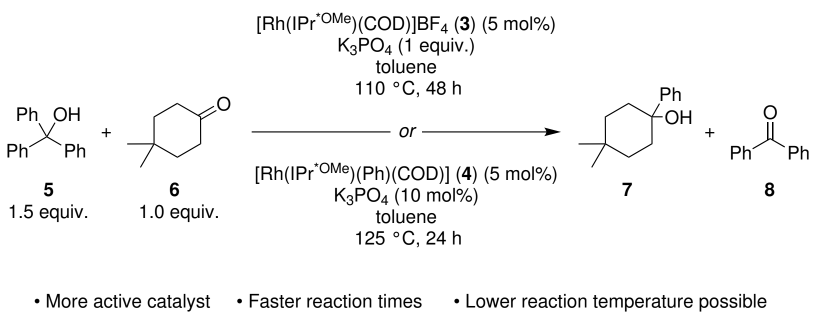

Supplemental References

(1) Pangborn, A. B., Giardello, M. A., Grubbs, R. H., Rosen, R. K., Timmers, F. J. *Organometallics* **1996**, *15*, 1518–1520.

(2) Fulmer, G. R., Miller, A. J. M., Sherden, N. H., Gottlieb, H. E., Nudelman, A., Stoltz, B. M., Bercaw, J. E., Goldberg, K. I. *Organometallics* **2010**, *29*, 2176–2179.

(3) Meiries, S., Speck, K., Cordes, D. B., Slawin, A. M. Z., Nolan, S. P. *Organometallics* **2013**, *32*, 330–339.

(4) Conway, B., Graham, D. V., Hevia, E., Kennedy, A. R., Klett, J., Mulvey, R. E. *Chem. Commun.* **2008**, 2638–2640.

(5) Clegg, W., Conway, B., Graham, D. V., Hevia, E., Kennedy, A. R., Mulvey, R. E., Russo, L., Wright, D. S. *Chem. – Eur. J.* **2009**, *15*, 7074–7082.

(6) Spee, M. P. R., Boersma, J., Meijer, M. D., Slagt, M. Q., van Koten, G., Geus, J. W. *J. Org. Chem.* **2001**, *66*, 1647–1656.

(7) DeMott, J. C., Bhuvanesh, N., Ozerov, O. V. *Chem. Sci.* **2013**, *4*, 642–649.

(8) Lutz, M. D. R., Gasser, V. C., Morandi, B. *Chem* **2021**, *7*, 1108–1119.

(9) Lutz, M. D. R., Zhong, H., Trapp, N., Morandi, B. *Helv. Chim. Acta* **2023**, *106*, e202200199.

(10) Yu, X.-Y., Patrick, B. O., James, B. R. *Organometallics* **2006**, *25*, 2359–2363.

(11) Sheldrick, G. M. *SADABS, Program for Empirical Absorption Correction of Area Detector Data*, University of Göttingen, Germany, 1996.

(12) Sheldrick, G. M. *Acta Crystallogr. A* **2015**, *71*, 3–8.

(13) Sheldrick, G. M. *Acta Crystallogr. A* **2008**, *64*, 112–122.

(14) Sheldrick, G. M. *Acta Crystallogr. C* **2015**, *71*, 3–8.

(15) Dolomanov, O. V., Bourhis, L. J., Gildea, R. J., Howard, J. A., Puschmann, H. *J. Appl. Crystallogr.* **2009**, *42*, 339–341.

(16) Sioriki, E., Lordan, R., Nahra, F., Van Hecke, K., Zabetakis, I., Nolan, S. P. *ChemMedChem* **2018**, *13*, 2484–2487.

(17) Blackmond, D. G. *Angew. Chem. Int. Ed.* **2005**, *44*, 4302–4320.

(18) Blackmond, D. G. *J. Am. Chem. Soc.* **2015**, *137*, 10852–10866.

(19) Nielsen, C. D.-T., Burés, J. *Chem. Sci.* **2019**, *10*, 348–353.

(20) Burés, J., Larrosa, I. *Nature* **2023**, *613*, 689–695.

(21) Hill, D. E., Pei, Q.-L., Zhang, E.-X., Gage, J. R., Yu, J.-Q., Blackmond, D. G. *ACS Catal.* **2018**, *8*, 1528–1531.

(22) Wedi, P., Farizyan, M., Bergander, K., Mück-Lichtenfeld, C., Gemmeren, M. van *Angew. Chem. Int. Ed.* **2021**, *60*, 15641–15649.

(23) Boller, T. M., Murphy, J. M., Hapke, M., Ishiyama, T., Miyaura, N., Hartwig, J. F. *J. Am. Chem. Soc.* **2005**, *127*, 14263–14278.

(24) Burés, J. *Top. Catal.* **2017**, *60*, 631–633.

(25) Pregosin, P. S., Anil Kumar, P. G., Fernández, I. *Chem. Rev.* **2005**, *105*, 2977–2998.

(26) Castañar, L., Poggetto, G. D., Colbourne, A. A., Morris, G. A., Nilsson, M. *Magn. Reson. Chem.* **2018**, *56*, 546–558.

(27) Pracht, P., Bohle, F., Grimme, S. *Phys. Chem. Chem. Phys.* **2020**, *22*, 7169–7192.

(28) Gaussian Inc.: Wallingford *Gaussian09*, 2009.

(29) Bootsma, A., N., Wheeler, S. *ChemRxiv* **2019**, DOI: 10.26434/CHEMRXIV.8864204.V5.

(30) Perdew, J. P., Burke, K., Ernzerhof, M. *Phys. Rev. Lett.* **1996**, *77*, 3865–3868.

(31) Perdew, J. P., Burke, K., Ernzerhof, M. *Phys. Rev. Lett.* **1997**, *78*, 1396–1396.

(32) Ernzerhof, M., Scuseria, G. E. *J. Chem. Phys.* **1999**, *110*, 5029–5036.

(33) Adamo, C., Barone, V. *J. Chem. Phys.* **1999**, *110*, 6158–6170.

(34) Grimme, S., Antony, J., Ehrlich, S., Krieg, H. *J. Chem. Phys.* **2010**, *132*, 154104.

(35) Grimme, S., Ehrlich, S., Goerigk, L. *J. Comput. Chem.* **2011**, *32*, 1456–1465.

(36) Weigend, F., Ahlrichs, R. *Phys. Chem. Chem. Phys.* **2005**, *7*, 3297.

(37) Weigend, F. *Phys. Chem. Chem. Phys.* **2006**, *8*, 1057–1065.

(38) Ribeiro, R. F., Marenich, A. V., Cramer, C. J., Truhlar, D. G. *J. Phys. Chem. B* **2011**, *115*, 14556–14562.

(39) Luchini, G., Alegre-Requena, J. V., Funes-Ardoiz, I., Paton, R. S. *F1000Research* **2020**, *9*, 291.

(40) Marenich, A. V., Cramer, C. J., Truhlar, D. G. *J. Phys. Chem. B* **2009**, *113*, 6378–6396.

(41) Weymuth, T., Couzijn, E. P. A., Chen, P., Reiher, M. *J. Chem. Theory Comput.* **2014**, *10*, 3092–3103.

(42) Legault, C. Y. *CYLview 1.0b*, Université de Sherbrooke, 2009.

(43) Legault, C. Y. *CYLview20*, Université de Sherbrooke, 2020.

(44) Falivene, L., Cao, Z., Petta, A., Serra, L., Poater, A., Oliva, R., Scarano, V., Cavallo, L. *Nat. Chem.* **2019**, *11*, 872–879.

(45) Bogdos, M. K., Morandi, B. *J. Chem. Ed.* **2023**, *100*, 3641–3644.

DFT Calculations: Cartesian Coordinates of Structures

For the energy values see Table S21.

Cl^-

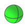

Cl	0.000 000	0.000 000	0.000 000

COD

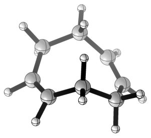

C	−1.198 528	−1.232 622	−0.498 314
C	0.028 870	−1.689 293	−0.218 909
C	1.086 308	−1.086 266	0.665 872
C	1.913 753	0.004 767	−0.020 043
C	1.198 583	1.232 617	−0.498 192
C	−0.028 848	1.689 287	−0.218 938
C	−1.086 381	1.086 269	0.665 736
C	−1.913 750	−0.004 756	−0.020 283
H	−2.730 786	−0.317 025	0.657 771
H	−2.428 954	0.438 805	−0.891 205
H	−1.778 953	1.885 744	0.972 395
H	−0.656 215	0.701 728	1.600 044
H	−0.322 252	2.616 895	−0.724 943
H	1.794 315	1.846 156	−1.185 300
H	2.730 687	0.317 052	0.658 128
H	2.429 089	−0.438 796	−0.890 885
H	1.778 854	−1.885 739	0.972 599
H	0.656 042	−0.701 738	1.600 139
H	0.322 316	−2.616 921	−0.724 852
H	−1.794 187	−1.846 181	−1.185 468

Me_2CO

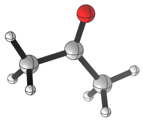

O	0.000 001	1.392 541	0.000 000
C	0.000 000	0.185 649	0.000 000
C	1.283 461	−0.610 924	0.000 888
H	2.142 294	0.070 156	0.044 234
H	1.310 493	−1.302 966	0.857 498
H	1.347 581	−1.228 766	−0.909 290
C	−1.283 462	−0.610 922	−0.000 888
H	−1.347 583	−1.228 766	0.909 289
H	−2.142 294	0.070 160	−0.044 232
H	−1.310 496	−1.302 962	−0.857 499

OH^-

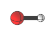

O	0.000 000	0.000 000	0.108 959
H	0.000 000	0.000 000	−0.871 673

Ph_2CO

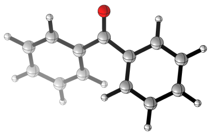

O	−0.000 016	2.308 140	0.000 127
C	−0.000 015	1.093 194	0.000 123
C	−1.294 373	0.343 516	−0.025 417
C	−2.424 786	0.979 485	0.504 432
C	−3.665 495	0.354 120	0.468 843
C	−3.795 555	−0.903 883	−0.122 690
C	−2.681 991	−1.531 384	−0.678 650
C	−1.433 794	−0.914 355	−0.625 916
H	−0.567 562	−1.401 943	−1.077 740
H	−2.786 070	−2.506 466	−1.160 169
H	−4.771 874	−1.393 499	−0.157 117
H	−4.539 468	0.850 157	0.897 468
H	−2.301 779	1.973 658	0.939 097
C	1.294 361	0.343 537	0.025 505
C	2.424 674	0.979 452	−0.504 614
C	3.665 398	0.354 104	−0.469 153
C	3.795 575	−0.903 815	0.122 529
C	2.682 113	−1.531 255	0.678 762
C	1.433 898	−0.914 253	0.626 146
H	0.567 743	−1.401 795	1.078 168
H	2.786 286	−2.506 268	1.160 400
H	4.771 907	−1.393 411	0.156 860
H	4.539 291	0.850 095	−0.897 995
H	2.301 598	1.973 571	−0.939 383

Ph_3CO^-

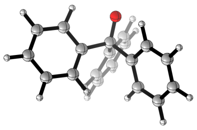

C	−0.000 307	−0.000 006	0.797 851
C	0.674 258	1.291 472	0.223 977
C	0.464 110	1.857 098	−1.038 050
C	1.132 587	3.023 096	−1.421 758
C	2.023 743	3.642 887	−0.547 625
C	2.227 198	3.093 113	0.721 052
C	1.549 480	1.938 600	1.101 167
H	1.623 444	1.478 968	2.091 935
H	2.914 066	3.580 376	1.421 189
H	2.545 851	4.556 779	−0.847 144
H	0.946 574	3.454 610	−2.410 567
H	−0.250 005	1.399 617	−1.727 830
C	0.781 135	−1.229 732	0.223 785
C	0.906 386	−2.310 508	1.101 532
C	1.568 212	−3.474 173	0.721 264
C	2.144 311	−3.573 088	−0.548 188

C	2.050 609	−2.492 141	−1.422 960
C	1.374 383	−1.330 736	−1.039 005
H	1.333 390	−0.484 007	−1.729 233
H	2.515 906	−2.546 956	−2.412 423
H	2.675 388	−4.481 772	−0.847 785
H	1.648 764	−4.312 067	1.421 872
H	0.472 530	−2.144 566	2.092 791
C	−1.456 123	−0.061 751	0.224 022
C	−1.840 620	−0.525 324	−1.038 518
C	−3.184 658	−0.529 896	−1.422 139
C	−4.167 252	−0.069 778	−0.547 374
C	−3.793 130	0.380 038	0.721 827
C	−2.454 395	0.370 910	1.101 820
H	−2.093 244	0.663 870	2.092 840
H	−4.558 770	0.729 833	1.422 434
H	−5.219 792	−0.075 015	−0.846 772
H	−3.465 165	−0.905 767	−2.411 370
H	−1.087 181	−0.913 752	−1.728 797
O	−0.000 165	−0.000 079	2.117 849

$PhMe_2CO^-$

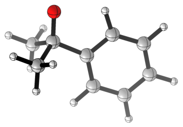

C	1.521 555	0.164 729	−0.000 001
C	2.038 363	−0.602 723	−1.263 628
H	1.759 308	−1.672 061	−1.325 931
H	3.137 912	−0.517 479	−1.274 007
H	1.656 196	−0.080 813	−2.155 612
C	2.038 358	−0.602 787	1.263 604
H	1.759 296	−1.672 126	1.325 851
H	1.656 193	−0.080 914	2.155 610
H	3.137 907	−0.517 548	1.273 980
C	−0.036 732	−0.000 397	0.000 001
C	−0.765 080	1.191 789	0.000 006
C	−2.158 617	1.187 628	0.000 006
C	−2.857 291	−0.022 375	−0.000 003
C	−2.142 497	−1.222 346	−0.000 007
C	−0.745 946	−1.207 868	−0.000 004
H	−0.200 913	−2.157 948	−0.000 004
H	−2.680 273	−2.176 977	−0.000 014
H	−3.952 098	−0.032 862	−0.000 006
H	−2.713 427	2.133 056	0.000 010
H	−0.122 856	2.082 979	0.000 011
O	1.901 259	1.422 350	0.000 034

Intermediate I

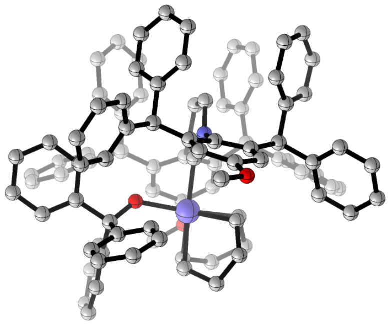

Rh	0.392 749	−0.151 900	−1.774 832
C	−1.460 745	−0.680 993	−2.605 714
C	−1.206 642	0.706 770	−2.741 044
C	−0.824 235	1.410 785	−4.016 841
C	0.293 780	0.688 815	−4.771 561
C	1.228 865	−0.023 494	−3.823 234
C	1.081 124	−1.349 873	−3.446 947
C	−0.012 641	−2.267 361	−3.928 009
C	−1.412 482	−1.670 315	−3.752 995
H	−2.136 842	−2.476 881	−3.562 313
H	−1.749 237	−1.180 058	−4.681 507
H	0.055 186	−3.203 764	−3.358 596
H	0.173 312	−2.540 909	−4.983 862
H	1.961 456	−1.847 384	−3.032 907
H	2.207 853	0.433 275	−3.681 361
H	0.866 932	1.414 832	−5.367 350
H	−0.125 952	−0.032 531	−5.489 957
H	−0.486 170	2.422 824	−3.744 875
H	−1.715 800	1.553 856	−4.657 319
H	−1.696 648	1.379 036	−2.037 200
H	−2.129 572	−0.973 695	−1.794 005
C	−0.487 142	−0.005 580	0.090 100
N	−0.619 187	−1.106 947	0.887 153
C	−1.263 743	−0.800 590	2.077 118
C	−1.555 379	0.516 844	2.029 674
N	−1.072 479	0.995 123	0.814 124
C	−1.238 853	2.347 493	0.361 706
C	−0.273 232	3.321 164	0.667 525
C	−0.383 565	4.590 488	0.099 094
C	−1.459 160	4.904 540	−0.730 888
C	−2.487 709	3.974 141	−0.909 938
C	−2.404 203	2.701 885	−0.355 508
C	−3.591 372	1.748 969	−0.434 577
C	−4.563 574	2.064 201	−1.562 770
C	−5.589 263	3.000 214	−1.380 131
C	−6.474 050	3.302 221	−2.412 532
C	−6.358 722	2.662 147	−3.645 799
C	−5.350 333	1.719 902	−3.835 239
C	−4.459 494	1.426 405	−2.803 366
H	−3.669 784	0.692 031	−2.968 035
H	−5.250 640	1.205 532	−4.794 337
H	−7.056 680	2.893 582	−4.453 877
H	−7.265 220	4.037 817	−2.248 505
H	−5.701 889	3.490 634	−0.410 259
C	−4.347 455	1.674 274	0.886 667
C	−4.199 537	2.624 752	1.898 774
C	−4.937 457	2.528 665	3.079 567
C	−5.842 404	1.486 604	3.260 481
C	−6.012 584	0.543 547	2.245 702
C	−5.272 729	0.639 451	1.071 121
H	−5.421 687	−0.095 742	0.275 405
H	−6.726 274	−0.274 748	2.370 144
H	−6.419 806	1.411 471	4.184 814
H	−4.800 408	3.277 512	3.863 086
H	−3.491 908	3.445 326	1.767 913
H	−3.186 984	0.741 249	−0.622 616
H	−3.354 188	4.268 095	−1.502 079
O	−1.598 244	6.083 740	−1.362 313
C	−0.595 447	7.052 355	−1.201 014
H	−0.508 457	7.380 754	−0.150 737
H	0.388 969	6.681 390	−1.534 968
H	−0.886 170	7.909 727	−1.820 572
H	0.392 515	5.321 210	0.319 679
C	0.802 198	3.048 319	1.709 736

C	1.864 972	4.135 937	1.798 802
C	2.975 509	4.094 086	0.950 530
C	3.947 373	5.090 869	0.990 381
C	3.825 252	6.155 277	1.881 065
C	2.725 686	6.207 231	2.735 855
C	1.758 254	5.204 177	2.698 335
H	0.914 934	5.251 075	3.389 419
H	2.621 706	7.029 900	3.447 859
H	4.587 747	6.937 390	1.915 639
H	4.804 086	5.023 246	0.316 556
H	3.083 491	3.273 333	0.243 971
C	0.151 270	2.809 892	3.061 634
C	0.656 268	1.836 367	3.925 687
C	0.052 522	1.594 901	5.157 234
C	−1.059 554	2.336 485	5.552 295
C	−1.560 451	3.325 043	4.706 487
C	−0.960 153	3.556 379	3.470 402
H	−1.370 189	4.317 243	2.801 397
H	−2.428 837	3.916 154	5.007 520
H	−1.537 189	2.141 425	6.515 269
H	0.441 279	0.804 567	5.801 426
H	1.508 657	1.229 708	3.612 585
H	1.304 469	2.116 592	1.411 875
H	−2.047 444	1.160 288	2.750 272
H	−1.443 333	−1.538 923	2.849 216
C	−0.371 581	−2.416 744	0.354 623
C	0.909 372	−2.989 186	0.389 620
C	1.160 331	−4.080 413	−0.447 770
C	0.145 347	−4.633 363	−1.227 381
C	−1.171 279	−4.188 578	−1.068 621
C	−1.447 201	−3.093 204	−0.263 377
C	−2.876 108	−2.723 207	0.112 607
C	−3.197 073	−3.299 747	1.489 984
C	−2.692 475	−4.531 804	1.917 969
C	−3.019 911	−5.037 984	3.176 268
C	−3.847 760	−4.313 803	4.031 480
C	−4.350 420	−3.080 721	3.615 431
C	−4.029 852	−2.583 558	2.355 710
H	−4.409 545	−1.608 296	2.046 878
H	−4.991 397	−2.494 505	4.278 691
H	−4.096 853	−4.705 912	5.020 425
H	−2.615 419	−6.003 097	3.491 510
H	−2.022 853	−5.097 004	1.265 048
C	−3.946 512	−3.108 286	−0.892 963
C	−4.398 754	−4.426 870	−1.026 575
C	−5.378 004	−4.754 162	−1.961 937
C	−5.939 835	−3.766 311	−2.770 284
C	−5.520 608	−2.445 724	−2.627 394
C	−4.535 273	−2.126 174	−1.695 708
H	−4.222 592	−1.086 414	−1.592 643
H	−5.961 493	−1.651 776	−3.235 344
H	−6.711 556	−4.024 623	−3.499 182
H	−5.712 615	−5.790 453	−2.052 042
H	−3.994 322	−5.206 334	−0.377 187
H	−2.926 536	−1.629 255	0.218 574
H	−1.964 630	−4.707 639	−1.606 705
O	0.339 242	−5.623 039	−2.120 050
C	1.652 656	−6.047 535	−2.371 360
H	2.116 577	−6.501 316	−1.478 277
H	1.591 295	−6.804 077	−3.163 497
H	2.287 454	−5.212 959	−2.716 665
H	2.166 036	−4.493 917	−0.474 418
C	1.967 587	−2.481 770	1.357 302
C	3.231 658	−3.330 826	1.428 007
C	3.171 040	−4.688 321	1.777 228
C	4.329 497	−5.449 811	1.895 030
C	5.579 377	−4.863 230	1.687 070
C	5.653 731	−3.509 244	1.374 814
C	4.489 488	−2.750 556	1.252 323
H	4.565 572	−1.689 500	1.015 993
H	6.621 947	−3.026 254	1.223 616
H	6.489 948	−5.459 870	1.781 973
H	4.257 521	−6.506 970	2.163 065
H	2.200 913	−5.153 455	1.968 417
C	1.457 001	−2.405 948	2.791 991
C	0.456 543	−3.252 787	3.278 901
C	0.080 476	−3.219 115	4.621 242
C	0.719 369	−2.353 519	5.506 604
C	1.738 492	−1.525 108	5.035 847
C	2.098 380	−1.548 504	3.691 382
H	2.910 435	−0.913 751	3.329 383
H	2.263 646	−0.856 242	5.722 897
H	0.431 090	−2.330 006	6.560 279
H	−0.720 856	−3.875 491	4.969 588
H	−0.056 714	−3.936 092	2.599 458
H	2.247 969	−1.467 288	1.021 843
O	2.197 919	0.053 808	−0.775 691
C	3.413 440	0.498 994	−1.231 048
C	4.374 394	0.576 261	−0.017 473
C	3.831 619	0.842 999	1.241 124
C	4.642 485	0.966 284	2.366 342
C	6.021 612	0.793 444	2.256 486
C	6.574 272	0.501 500	1.010 562
C	5.758 719	0.397 102	−0.116 715
H	6.204 674	0.143 032	−1.081 031
H	7.651 920	0.346 801	0.912 930
H	6.661 137	0.878 307	3.138 110
H	4.195 944	1.198 843	3.336 970
H	2.747 107	0.921 008	1.308 741
C	3.341 984	1.941 681	−1.800 147
C	2.107 280	2.560 588	−2.005 075
C	2.016 904	3.861 745	−2.497 644
C	3.170 211	4.587 612	−2.780 692
C	4.413 749	3.996 366	−2.556 368
C	4.496 183	2.694 071	−2.068 059
H	5.481 359	2.265 446	−1.875 751
H	5.331 214	4.556 128	−2.756 008
H	3.105 355	5.610 415	−3.160 172
H	1.030 568	4.310 237	−2.642 986
H	1.194 561	2.006 771	−1.758 909
C	4.045 784	−0.485 454	−2.242 616
C	4.680 152	−0.131 535	−3.435 303
C	5.248 398	−1.104 503	−4.261 655
C	5.207 952	−2.448 441	−3.901 518
C	4.575 341	−2.814 519	−2.712 369
C	3.991 189	−1.843 445	−1.907 024
H	3.464 986	−2.129 472	−0.998 970
H	4.535 677	−3.863 874	−2.407 449
H	5.663 372	−3.206 302	−4.543 677
H	5.728 689	−0.802 221	−5.195 788
H	4.722 946	0.914 967	−3.741 980

Intermediate II

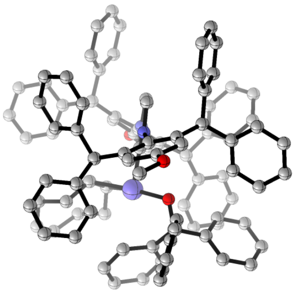

C	−4.895 168	3.672 867	−4.155 374
O	−5.266 206	2.838 204	−3.091 483
C	−4.317 415	2.433 158	−2.228 560
C	−4.729 908	1.563 438	−1.214 372
C	−3.824 383	1.072 190	−0.280 584
C	−2.472 679	1.464 002	−0.380 647
C	−2.047 574	2.354 441	−1.373 985
C	−2.979 938	2.827 404	−2.299 290
H	−2.634 096	3.500 492	−3.082 146
C	−0.600 706	2.806 509	−1.445 344
C	−0.219 088	3.204 951	−2.860 379
C	−0.066 820	4.534 497	−3.259 829
C	0.250 796	4.843 963	−4.584 394
C	0.420 125	3.828 585	−5.522 448
C	0.273 102	2.497 258	−5.128 938
C	−0.042 160	2.191 756	−3.810 025
H	−0.134 310	1.149 991	−3.489 228
H	0.416 235	1.689 389	−5.850 823
H	0.674 999	4.072 169	−6.556 611
H	0.370 984	5.888 942	−4.880 944
H	−0.189 689	5.335 448	−2.527 156
C	−0.257 169	3.881 353	−0.430 930
C	1.092 067	4.112 949	−0.129 073
C	1.469 970	5.153 604	0.713 243
C	0.498 128	5.981 037	1.279 266
C	−0.847 882	5.742 885	1.009 453
C	−1.223 558	4.699 843	0.159 837
H	−2.281 052	4.526 761	−0.054 453
H	−1.616 887	6.375 962	1.459 309
H	0.790 730	6.797 076	1.944 140
H	2.528 608	5.301 472	0.939 896
H	1.862 670	3.474 217	−0.564 803
H	0.012 050	1.919 825	−1.204 663
N	−1.539 234	0.974 731	0.580 449
C	−1.651 465	1.380 242	1.901 208
C	−0.684 393	0.743 806	2.584 495
N	0.044 417	0.004 055	1.665 541
C	−0.484 036	0.110 624	0.401 977
Rh	0.134 075	−0.822 416	−1.197 552
C	−1.730 411	−1.019 602	−2.141 657
C	−1.577 287	−2.046 087	−1.182 148
C	−1.382 886	−3.507 482	−1.501 656
H	−0.619 281	−3.634 333	−2.276 182
H	−0.990 535	−4.025 188	−0.613 934
C	−2.680 929	−4.204 026	−1.972 493
C	−2.780 740	−4.350 966	−3.466 359
C	−2.905 438	−3.379 592	−4.380 389
C	−3.003 046	−1.896 200	−4.149 912
H	−3.295 067	−1.421 220	−5.098 613
H	−3.821 484	−1.670 729	−3.443 958
C	−1.704 178	−1.229 777	−3.633 075
H	−0.841 546	−1.829 559	−3.948 139
H	−1.595 041	−0.248 486	−4.118 966
H	−2.916 775	−3.697 374	−5.429 726
H	−2.702 012	−5.375 064	−3.850 010
H	−3.550 418	−3.651 991	−1.578 303
H	−2.735 864	−5.205 992	−1.520 890
H	−2.128 382	−1.893 329	−0.244 670
H	−2.388 585	−0.194 011	−1.870 378
O	2.020 119	−0.162 771	−0.900 113

C	2.920 246	−0.558 457	−1.879 274
C	2.171 657	−1.262 618	−3.037 973
C	2.057 294	−0.706 339	−4.323 098
C	1.526 402	−1.443 515	−5.376 699
C	1.101 394	−2.759 089	−5.177 083
C	1.170 025	−3.310 800	−3.903 216
C	1.680 355	−2.563 350	−2.836 776
H	1.792 419	−3.037 639	−1.860 303
H	0.836 482	−4.336 701	−3.732 454
H	0.708 913	−3.346 295	−6.010 238
H	1.466 146	−0.994 924	−6.371 588
H	2.428 427	0.303 222	−4.503 341
C	3.655 099	0.678 787	−2.433 589
C	2.959 238	1.887 821	−2.472 954
C	3.521 073	3.034 582	−3.025 726
C	4.816 441	2.991 973	−3.540 604
C	5.531 371	1.795 963	−3.492 483
C	4.955 807	0.647 578	−2.945 707
H	5.533 232	−0.277 842	−2.913 942
H	6.551 744	1.752 467	−3.881 893
H	5.269 278	3.887 982	−3.972 045
H	2.939 519	3.959 595	−3.053 918
H	1.955 611	1.900 740	−2.056 564
C	3.965 844	−1.529 094	−1.304 269
C	4.618 682	−2.494 784	−2.081 221
C	5.596 972	−3.317 958	−1.524 251
C	5.947 926	−3.179 497	−0.181 955
C	5.309 629	−2.214 624	0.595 499
C	4.321 266	−1.404 931	0.038 768
H	3.803 114	−0.664 648	0.645 534
H	5.579 885	−2.085 123	1.645 658
H	6.719 062	−3.819 432	0.254 423
H	6.089 745	−4.069 533	−2.146 570
H	4.359 129	−2.612 643	−3.135 833
C	1.254 317	−0.606 863	2.127 688
C	2.267 608	0.234 423	2.629 031
C	3.387 884	−0.345 039	3.216 438
C	3.488 507	−1.730 798	3.357 731
C	2.470 110	−2.548 819	2.870 887
C	1.353 774	−1.995 977	2.240 624
C	0.242 069	−2.882 488	1.723 274
C	0.755 933	−4.246 480	1.295 502
C	1.839 758	−4.291 381	0.404 460
C	2.326 991	−5.502 912	−0.071 470
C	1.738 932	−6.702 023	0.338 484
C	0.674 098	−6.670 075	1.234 093
C	0.186 816	−5.450 628	1.712 411
H	−0.649 176	−5.440 026	2.414 648
H	0.213 249	−7.602 265	1.570 376
H	2.116 888	−7.657 163	−0.034 096
H	3.174 133	−5.504 245	−0.762 021
H	2.322 117	−3.357 038	0.105 363
C	−0.953 253	−2.916 856	2.657 529
C	−2.216 077	−3.268 036	2.158 238
C	−3.332 119	−3.316 754	2.989 059
C	−3.206 853	−3.013 362	4.345 647
C	−1.959 604	−2.657 925	4.854 245
C	−0.843 981	−2.610 446	4.017 368
H	0.128 722	−2.327 496	4.426 350
H	−1.849 504	−2.414 295	5.913 919
H	−4.081 041	−3.047 672	5.000 242
H	−4.305 709	−3.583 741	2.570 472
H	−2.324 265	−3.521 659	1.102 391
H	−0.099 450	−2.402 354	0.784 064
H	2.537 276	−3.632 719	2.946 003
O	4.608 559	−2.195 801	3.949 272
C	4.765 580	−3.583 441	4.083 133
H	5.735 282	−3.743 132	4.571 027
H	4.766 164	−4.089 294	3.101 875
H	3.971 829	−4.025 192	4.710 430
H	4.215 350	0.278 746	3.559 366
C	2.150 408	1.747 618	2.573 362
C	3.449 123	2.391 872	2.117 414
C	4.204 758	3.249 186	2.918 533
C	5.380 061	3.819 599	2.423 278
C	5.805 740	3.539 881	1.126 959
C	5.052 560	2.683 117	0.321 070
C	3.883 420	2.115 157	0.815 324
H	3.284 055	1.447 157	0.187 908
H	5.368 527	2.457 257	−0.699 979
H	6.724 060	3.989 509	0.741 268
H	5.963 751	4.490 128	3.059 261
H	3.870 409	3.482 014	3.931 882
C	1.606 170	2.340 521	3.863 505
C	0.767 637	3.459 115	3.787 062
C	0.250 233	4.048 044	4.936 407
C	0.559 985	3.525 420	6.192 459
C	1.390 350	2.410 813	6.281 584
C	1.908 171	1.822 461	5.126 363
H	2.545 452	0.940 430	5.213 009
H	1.637 730	1.988 408	7.258 724
H	0.151 140	3.982 073	7.097 048
H	−0.406 657	4.916 980	4.847 047
H	0.509 396	3.864 505	2.807 626
H	1.418 295	1.990 629	1.788 772
H	−0.422 973	0.761 173	3.636 031
H	−2.409 570	2.085 114	2.220 839
C	−4.301 972	0.138 789	0.821 608
C	−5.415 356	−0.792 306	0.359 269
C	−5.196 264	−1.598 317	−0.765 283
C	−6.168 419	−2.484 853	−1.215 050
C	−7.388 368	−2.583 069	−0.543 348
C	−7.618 287	−1.786 369	0.574 374
C	−6.639 267	−0.896 973	1.023 540
H	−6.832 987	−0.279 465	1.902 500
H	−8.569 983	−1.852 061	1.107 469
H	−8.155 921	−3.277 362	−0.893 363
H	−5.970 609	−3.101 861	−2.095 687
H	−4.246 040	−1.517 490	−1.295 926
C	−4.666 067	0.891 798	2.089 815
C	−5.270 587	2.151 342	2.060 906
C	−5.592 654	2.811 707	3.247 423
C	−5.315 269	2.220 586	4.478 274
C	−4.716 402	0.960 828	4.515 185
C	−4.395 993	0.304 288	3.330 822
H	−3.909 060	−0.672 821	3.365 246
H	−4.485 395	0.488 724	5.473 338
H	−5.560 452	2.741 676	5.406 759
H	−6.059 983	3.798 690	3.206 727
H	−5.480 337	2.628 492	1.101 008
H	−3.451 921	−0.512 563	1.085 883
H	−5.775 238	1.251 841	−1.190 176

H	−5.803 606	3.853 512	−4.743 364
H	−4.500 609	4.640 661	−3.799 639
H	−4.135 847	3.198 708	−4.801 216

Intermediate III

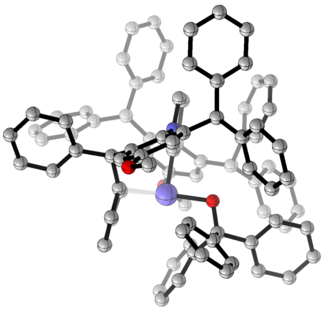

Rh	0.008 918	0.385 348	−1.355 937
C	−0.474 998	−0.373 033	0.330 064
N	−1.383 440	0.080 721	1.240 539
C	−1.425 810	−0.748 966	2.352 663
C	−0.548 623	−1.752 670	2.128 929
N	0.026 423	−1.508 842	0.886 236
C	0.841 205	−2.422 807	0.165 956
C	2.093 320	−2.792 193	0.657 519
C	2.878 906	−3.674 875	−0.089 309
C	2.413 685	−4.183 030	−1.300 218
C	1.139 081	−3.830 517	−1.763 038
C	0.343 426	−2.947 756	−1.045 663
C	−1.001 686	−2.491 202	−1.568 297
C	−0.865 220	−1.212 479	−2.391 190
C	−0.122 330	−1.270 966	−3.628 542
C	−0.385 645	−0.403 983	−4.653 653
C	−1.458 313	0.534 620	−4.566 674
C	−2.178 449	0.638 216	−3.409 889
C	−1.852 726	−0.169 643	−2.270 925
H	−2.616 421	−0.241 240	−1.497 178
H	−3.015 655	1.335 923	−3.330 201
H	−1.693 586	1.164 031	−5.426 334
H	0.212 119	−0.451 305	−5.566 822
H	0.654 729	−2.030 212	−3.739 924
C	−1.805 791	−3.577 581	−2.263 761
C	−2.498 241	−3.344 296	−3.456 385
C	−3.309 780	−4.334 582	−4.012 934
C	−3.444 372	−5.571 007	−3.387 810
C	−2.760 121	−5.813 113	−2.196 128
C	−1.951 643	−4.825 596	−1.642 203
H	−1.419 824	−5.021 489	−0.707 178
H	−2.859 157	−6.778 331	−1.693 354
H	−4.079 891	−6.344 658	−3.825 117
H	−3.839 253	−4.131 854	−4.947 320
H	−2.411 516	−2.378 169	−3.956 445
H	−1.606 145	−2.218 240	−0.687 962
H	0.785 936	−4.265 240	−2.699 548
O	3.132 463	−4.996 108	−2.100 466
C	4.428 577	−5.353 084	−1.699 685
H	5.076 837	−4.467 354	−1.581 077
H	4.421 321	−5.916 829	−0.750 474
H	4.836 211	−5.993 508	−2.491 724
H	3.871 831	−3.922 727	0.284 150
C	2.608 017	−2.258 516	1.978 793
C	4.048 165	−1.802 445	1.826 422
C	5.134 945	−2.514 402	2.338 912
C	6.439 280	−2.079 006	2.096 339
C	6.666 793	−0.932 248	1.338 961
C	5.584 335	−0.217 675	0.823 527
C	4.284 623	−0.650 133	1.066 919
H	3.433 471	−0.110 901	0.636 396
H	5.747 854	0.675 416	0.216 549
H	7.688 095	−0.594 494	1.146 950
H	7.282 139	−2.643 306	2.503 628
H	4.961 873	−3.413 728	2.935 091
C	2.368 193	−3.212 898	3.137 043
C	1.825 295	−4.489 884	2.966 286
C	1.579 024	−5.316 797	4.064 441
C	1.873 604	−4.880 869	5.352 552
C	2.420 227	−3.609 533	5.535 849
C	2.662 948	−2.787 976	4.440 481
H	3.097 551	−1.798 102	4.592 641
H	2.657 993	−3.254 872	6.541 787
H	1.680 296	−5.527 005	6.212 058
H	1.152 837	−6.310 510	3.905 773
H	1.588 288	−4.844 169	1.960 884
H	2.025 336	−1.350 940	2.203 758
H	−0.268 995	−2.607 370	2.737 414
H	−2.063 710	−0.541 698	3.203 004
C	−2.250 865	1.183 817	0.972 569
C	−3.573 636	0.913 006	0.559 571
C	−4.362 532	1.965 870	0.116 776
C	−3.864 420	3.273 330	0.107 452
C	−2.586 680	3.534 962	0.601 617
C	−1.764 380	2.496 656	1.051 038
C	−0.362 058	2.794 801	1.563 264
C	0.001 220	2.033 333	2.824 115
C	−0.860 517	1.926 925	3.920 895
C	−0.464 471	1.249 058	5.073 116
C	0.800 845	0.668 004	5.145 592
C	1.663 359	0.767 112	4.054 371
C	1.262 578	1.437 132	2.901 672
H	1.912 762	1.487 288	2.025 871
H	2.660 252	0.321 673	4.093 788
H	1.110 233	0.132 407	6.046 308
H	−1.154 060	1.166 808	5.917 166
H	−1.867 722	2.349 032	3.863 395
C	−0.104 339	4.282 509	1.748 901
C	−0.715 601	5.011 826	2.778 139
C	−0.494 861	6.379 115	2.915 693
C	0.349 587	7.047 217	2.027 462
C	0.971 319	6.331 977	1.008 232
C	0.745 357	4.961 779	0.872 866
H	1.254 884	4.412 237	0.080 882
H	1.648 152	6.831 015	0.311 318
H	0.526 587	8.119 952	2.137 667
H	−0.981 824	6.926 922	3.726 499
H	−1.375 644	4.504 548	3.484 159
H	0.341 537	2.435 479	0.788 644
H	−2.203 011	4.552 377	0.642 959
O	−4.690 963	4.222 222	−0.370 802
C	−4.237 454	5.548 029	−0.412 878
H	−4.002 605	5.935 081	0.593 943
H	−3.339 210	5.653 525	−1.046 453

H	−5.051 174	6.144 437	−0.844 081
H	−5.377 817	1.795 608	−0.241 028
C	−4.131 737	−0.497 370	0.685 971
C	−5.280 735	−0.821 879	−0.250 570
C	−6.586 127	−0.395 385	0.023 372
C	−7.629 944	−0.695 663	−0.848 404
C	−7.389 527	−1.444 052	−2.000 875
C	−6.099 776	−1.896 849	−2.268 598
C	−5.055 897	−1.587 359	−1.398 783
H	−4.054 986	−1.963 757	−1.618 857
H	−5.895 116	−2.504 065	−3.153 648
H	−8.210 422	−1.684 866	−2.680 573
H	−8.641 665	−0.351 755	−0.619 827
H	−6.789 147	0.163 061	0.940 759
C	−4.508 492	−0.816 583	2.127 154
C	−4.768 066	0.176 284	3.074 932
C	−5.093 429	−0.164 546	4.389 380
C	−5.164 065	−1.501 624	4.771 036
C	−4.916 318	−2.499 888	3.826 638
C	−4.594 752	−2.158 268	2.517 580
H	−4.396 265	−2.941 636	1.780 755
H	−4.969 498	−3.552 365	4.115 604
H	−5.411 829	−1.768 288	5.801 108
H	−5.289 185	0.624 875	5.119 304
H	−4.699 884	1.227 980	2.787 097
H	−3.317 217	−1.191 906	0.426 828
O	1.663 349	1.231 391	−0.505 131
C	2.436 541	1.806 643	−1.505 273
C	1.517 424	2.257 376	−2.664 703
C	1.866 583	2.144 018	−4.017 629
C	1.088 636	2.742 513	−5.004 401
C	−0.059 358	3.460 578	−4.661 524
C	−0.441 450	3.548 919	−3.328 143
C	0.335 649	2.946 216	−2.337 001
H	0.059 030	3.073 474	−1.289 437
H	−1.345 569	4.092 607	−3.044 033
H	−0.659 328	3.939 505	−5.439 320
H	1.383 649	2.652 475	−6.052 895
H	2.770 869	1.597 858	−4.291 662
C	3.149 265	3.044 792	−0.935 225
C	3.688 846	2.966 223	0.352 917
C	4.341 759	4.053 078	0.925 271
C	4.475 635	5.245 065	0.212 669
C	3.953 505	5.332 365	−1.075 288
C	3.293 487	4.240 896	−1.644 164
H	2.881 020	4.328 837	−2.651 455
H	4.052 612	6.259 530	−1.645 847
H	4.981 612	6.102 639	0.662 523
H	4.742 725	3.970 514	1.938 341
H	3.582 539	2.037 018	0.911 021
C	3.497 687	0.823 043	−2.039 724
C	4.770 716	1.241 602	−2.441 345
C	5.694 580	0.332 884	−2.959 660
C	5.355 734	−1.011 087	−3.093 202
C	4.088 842	−1.439 036	−2.696 805
C	3.174 904	−0.531 793	−2.167 010
H	2.189 391	−0.871 164	−1.835 133
H	3.807 831	−2.489 338	−2.798 185
H	6.076 070	−1.723 828	−3.502 638
H	6.685 687	0.682 336	−3.260 206
H	5.049 370	2.293 009	−2.345 635

Intermediate IV

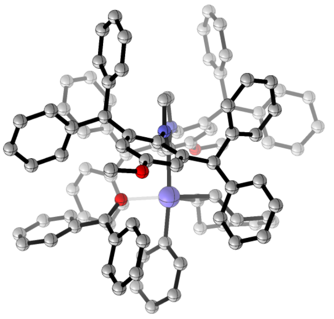

C	5.194 050	−3.890 259	3.019 572
O	3.951 850	−3.462 365	3.509 047
C	3.014 158	−3.070 560	2.624 892
C	1.833 780	−2.553 031	3.166 428
C	0.829 899	−2.044 019	2.352 092
C	0.995 838	−2.101 786	0.952 233
C	2.146 494	−2.683 685	0.394 842
C	3.166 116	−3.132 961	1.241 519
H	4.073 846	−3.530 832	0.788 904
C	2.275 934	−2.932 676	−1.098 438
C	3.701 736	−2.774 260	−1.600 816
C	4.506 479	−3.866 042	−1.938 638
C	5.808 821	−3.673 178	−2.402 466
C	6.319 547	−2.385 807	−2.546 118
C	5.523 143	−1.289 151	−2.213 140
C	4.230 230	−1.485 550	−1.737 727
H	3.607 974	−0.628 804	−1.473 989
H	5.904 764	−0.272 735	−2.328 384
H	7.334 347	−2.234 293	−2.921 815
H	6.422 459	−4.539 107	−2.663 297
H	4.106 204	−4.878 479	−1.852 137
C	1.687 984	−4.281 344	−1.492 509
C	1.174 008	−4.438 885	−2.785 217
C	0.626 646	−5.650 679	−3.193 432
C	0.583 887	−6.734 156	−2.314 540
C	1.094 036	−6.589 336	−1.027 082
C	1.641 471	−5.371 375	−0.618 687
H	2.022 723	−5.266 013	0.399 221
H	1.062 666	−7.428 227	−0.327 341
H	0.148 942	−7.685 002	−2.631 515
H	0.220 228	−5.746 083	−4.203 407
H	1.184 635	−3.587 862	−3.468 877
H	1.680 861	−2.165 487	−1.617 460
N	−0.055 849	−1.675 429	0.084 724
C	−0.727 638	−2.603 172	−0.698 755
C	−1.559 306	−1.904 618	−1.493 894
N	−1.417 442	−0.572 952	−1.137 531
C	−0.472 993	−0.388 769	−0.158 641
Rh	0.283 051	1.392 176	0.739 363
C	−1.548 353	1.693 840	1.736 162
C	−1.455 345	2.558 764	0.619 203
C	−1.451 836	4.054 029	0.784 299
H	−0.933 135	4.361 651	1.700 885
H	−0.891 333	4.526 853	−0.036 657
C	−2.864 900	4.624 693	0.759 137
C	−3.820 696	4.382 487	1.891 791
C	−3.865 437	3.606 329	2.987 584
C	−3.017 669	2.542 777	3.625 026
H	−2.957 841	2.790 562	4.700 488
H	−3.623 575	1.614 683	3.603 936
C	−1.606 908	2.228 375	3.139 795
H	−0.980 750	3.123 578	3.234 715
H	−1.184 669	1.490 957	3.839 434
H	−4.779 080	3.760 246	3.579 104
H	−4.711 420	5.016 767	1.782 057
H	−3.368 817	4.292 308	−0.170 787
H	−2.792 694	5.722 046	0.648 199
H	−1.969 749	2.253 523	−0.295 164
H	−2.138 141	0.778 392	1.595 615
O	2.263 351	1.064 348	−0.181 282

C	3.393 652	1.481 148	0.098 133
C	3.928 543	1.397 523	1.471 881
C	4.770 269	2.381 891	2.006 848
C	5.227 480	2.265 454	3.315 222
C	4.876 386	1.156 349	4.083 947
C	4.045 469	0.168 761	3.552 203
C	3.557 598	0.300 195	2.257 768
H	2.889 897	−0.447 746	1.831 618
H	3.772 092	−0.703 563	4.148 865
H	5.247 005	1.062 393	5.107 786
H	5.852 574	3.053 331	3.740 965
H	5.014 822	3.266 298	1.417 409
C	4.246 560	2.011 945	−0.993 339
C	5.647 813	2.005 755	−0.926 920
C	6.400 172	2.480 698	−1.998 365
C	5.763 859	2.964 943	−3.141 528
C	4.369 782	2.968 422	−3.218 075
C	3.618 728	2.490 402	−2.151 854
H	2.530 658	2.500 840	−2.197 617
H	3.858 469	3.348 087	−4.105 136
H	6.358 173	3.340 007	−3.978 379
H	7.491 133	2.465 873	−1.943 121
H	6.148 168	1.600 482	−0.046 126
C	1.237 567	3.002 584	1.558 848
C	1.834 017	3.957 491	0.706 077
C	2.621 011	5.011 761	1.174 048
C	2.859 390	5.155 595	2.540 022
C	2.297 926	4.225 485	3.414 912
C	1.507 905	3.181 758	2.930 353
H	1.090 985	2.485 558	3.660 110
H	2.478 810	4.310 661	4.490 922
H	3.473 374	5.977 592	2.917 561
H	3.051 158	5.723 980	0.462 605
H	1.682 122	3.883 164	−0.373 908
C	−2.258 008	0.427 650	−1.708 686
C	−1.710 655	1.386 127	−2.583 870
C	−2.542 424	2.368 710	−3.102 361
C	−3.897 852	2.420 912	−2.755 606
C	−4.436 758	1.441 101	−1.923 621
C	−3.625 769	0.427 613	−1.402 168
C	−4.251 775	−0.680 473	−0.572 841
C	−5.421 476	−0.212 869	0.282 313
C	−5.264 347	0.906 559	1.110 927
C	−6.295 130	1.322 417	1.947 890
C	−7.504 593	0.626 934	1.977 882
C	−7.674 021	−0.481 136	1.153 111
C	−6.641 306	−0.895 118	0.310 130
H	−6.787 683	−1.766 279	−0.330 413
H	−8.617 791	−1.032 186	1.159 184
H	−8.312 025	0.953 713	2.637 583
H	−6.144 989	2.203 078	2.574 889
H	−4.328 666	1.471 570	1.095 362
C	−4.616 659	−1.874 601	−1.442 248
C	−5.087 208	−1.726 239	−2.749 811
C	−5.416 497	−2.842 855	−3.519 428
C	−5.280 795	−4.125 020	−2.991 728
C	−4.815 067	−4.282 404	−1.685 817
C	−4.486 386	−3.167 150	−0.920 960
H	−4.104 758	−3.295 139	0.094 013
H	−4.696 052	−5.282 367	−1.261 191
H	−5.532 382	−4.999 320	−3.596 837

H	−5.777 282	−2.706 609	−4.541 973
H	−5.183 944	−0.726 559	−3.178 805
H	−3.485 759	−1.034 897	0.137 420
H	−5.489 808	1.454 793	−1.645 140
O	−4.603 763	3.447 293	−3.265 582
C	−5.944 426	3.592 801	−2.883 492
H	−6.310 641	4.508 112	−3.364 990
H	−6.047 367	3.694 743	−1.788 985
H	−6.562 603	2.741 408	−3.218 783
H	−2.148 637	3.142 183	−3.761 798
C	−0.230 435	1.346 295	−2.907 607
C	0.271 398	2.657 811	−3.482 412
C	0.706 257	2.803 066	−4.801 439
C	1.168 009	4.038 529	−5.262 401
C	1.205 044	5.140 266	−4.410 850
C	0.767 950	5.004 187	−3.091 572
C	0.302 669	3.774 967	−2.638 551
H	−0.038 423	3.655 670	−1.607 815
H	0.796 049	5.856 563	−2.408 233
H	1.572 913	6.103 507	−4.772 350
H	1.503 303	4.135 872	−6.298 146
H	0.691 262	1.942 838	−5.473 870
C	0.164 472	0.158 766	−3.759 374
C	1.495 809	−0.274 243	−3.732 854
C	1.934 532	−1.294 217	−4.571 647
C	1.037 767	−1.910 652	−5.446 726
C	−0.295 124	−1.504 361	−5.464 160
C	−0.727 998	−0.473 751	−4.628 536
H	−1.771 935	−0.151 269	−4.655 154
H	−1.005 916	−1.987 370	−6.139 341
H	1.377 894	−2.711 618	−6.107 754
H	2.978 877	−1.613 859	−4.530 631
H	2.198 132	0.212 525	−3.052 888
H	0.278 251	1.232 395	−1.930 450
H	−2.239 870	−2.225 911	−2.272 917
H	−0.547 400	−3.668 112	−0.618 458
C	−0.418 367	−1.427 177	2.953 971
C	−0.203 595	−1.036 655	4.405 079
C	0.699 869	−0.002 173	4.679 128
C	0.966 350	0.390 179	5.985 365
C	0.326 934	−0.249 785	7.049 350
C	−0.571 811	−1.280 650	6.788 478
C	−0.835 263	−1.673 933	5.474 053
H	−1.542 998	−2.482 815	5.280 616
H	−1.077 116	−1.787 956	7.614 087
H	0.528 741	0.056 929	8.078 501
H	1.673 474	1.202 946	6.170 227
H	1.199 329	0.494 502	3.844 116
C	−1.664 606	−2.264 974	2.756 709
C	−1.616 622	−3.643 389	2.532 393
C	−2.791 379	−4.392 232	2.445 810
C	−4.032 206	−3.772 803	2.582 917
C	−4.093 786	−2.393 943	2.792 639
C	−2.919 470	−1.651 988	2.877 402
H	−2.973 737	−0.576 728	3.061 722
H	−5.059 417	−1.890 264	2.881 253
H	−4.951 758	−4.359 202	2.517 005
H	−2.733 222	−5.470 115	2.274 548
H	−0.647 227	−4.138 462	2.434 173
H	−0.563 597	−0.477 978	2.401 406
H	1.733 705	−2.520 244	4.251 382
H	5.687 270	−3.103 704	2.422 634
H	5.814 526	−4.119 106	3.894 934
H	5.100 337	−4.799 854	2.400 810

Intermediate V

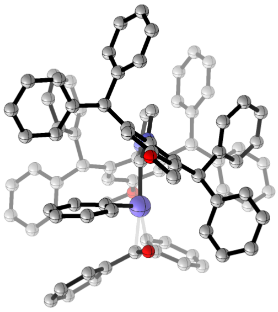

Rh	−0.349 474	0.623 502	1.588 724
C	1.145 864	1.886 877	1.641 258
C	2.183 301	1.626 279	2.552 658
C	3.150 867	2.589 497	2.839 472
C	3.103 501	3.845 695	2.233 156
C	2.090 178	4.113 115	1.315 151
C	1.134 643	3.140 354	1.011 177
H	0.336 308	3.410 317	0.321 251
H	2.027 035	5.091 310	0.830 116
H	3.852 869	4.604 191	2.472 232
H	3.943 395	2.355 871	3.555 853
H	2.222 840	0.669 202	3.075 770
C	0.164 694	−0.169 433	−0.193 280
N	−0.568 688	−0.364 078	−1.329 920
C	0.105 699	−1.157 771	−2.245 047
C	1.267 985	−1.515 564	−1.663 018
N	1.306 714	−0.890 067	−0.426 315
C	2.448 986	−0.969 920	0.430 645
C	3.662 899	−0.399 191	−0.002 551
C	4.759 116	−0.421 067	0.851 356
C	4.680 607	−1.038 288	2.101 456
C	3.497 212	−1.671 969	2.484 165
C	2.370 962	−1.644 087	1.659 467
C	1.090 452	−2.335 337	2.095 777
C	0.726 918	−3.536 772	1.246 354
C	1.656 458	−4.221 358	0.459 021
C	1.288 448	−5.381 037	−0.225 610
C	−0.013 161	−5.870 066	−0.132 854
C	−0.954 769	−5.182 640	0.635 969
C	−0.582 950	−4.028 222	1.317 066
H	−1.317 367	−3.504 018	1.933 752
H	−1.986 214	−5.538 134	0.699 187
H	−0.299 414	−6.779 167	−0.667 086
H	2.029 545	−5.906 978	−0.832 786
H	2.681 170	−3.847 989	0.386 963
C	1.086 261	−2.681 576	3.573 099
C	1.523 553	−3.928 158	4.034 070
C	1.571 300	−4.202 378	5.400 139
C	1.175 432	−3.235 363	6.323 324
C	0.726 870	−1.995 176	5.870 121
C	0.681 906	−1.718 414	4.504 670
H	0.317 434	−0.746 925	4.158 369
H	0.402 864	−1.232 635	6.582 750

H	1.208 601	−3.451 718	7.394 020
H	1.915 014	−5.181 080	5.744 493
H	1.825 709	−4.692 477	3.313 781
H	0.282 949	−1.582 857	1.962 631
H	3.421 562	−2.180 103	3.443 426
O	5.786 138	−0.990 418	2.866 947
C	5.744 142	−1.568 389	4.143 602
H	5.561 829	−2.656 293	4.096 928
H	6.725 930	−1.391 334	4.600 239
H	4.964 391	−1.108 605	4.775 436
H	5.696 208	0.055 443	0.559 236
C	3.822 096	0.150 276	−1.408 295
C	4.236 696	−0.940 566	−2.385 381
C	3.816 778	−0.852 718	−3.718 803
C	4.177 042	−1.823 641	−4.648 206
C	4.968 213	−2.906 135	−4.261 199
C	5.392 534	−3.003 311	−2.938 342
C	5.028 926	−2.028 438	−2.007 642
H	5.359 362	−2.121 579	−0.971 097
H	6.010 706	−3.847 045	−2.621 635
H	5.248 450	−3.672 389	−4.987 946
H	3.829 892	−1.738 332	−5.680 983
H	3.181 102	−0.017 804	−4.020 665
C	4.732 550	1.365 514	−1.469 504
C	5.889 047	1.412 364	−2.250 886
C	6.663 850	2.573 355	−2.300 808
C	6.290 523	3.699 253	−1.572 196
C	5.139 326	3.655 806	−0.783 384
C	4.370 509	2.498 693	−0.731 622
H	3.477 346	2.469 072	−0.103 903
H	4.833 502	4.526 492	−0.198 853
H	6.895 714	4.608 094	−1.614 527
H	7.566 066	2.593 150	−2.917 245
H	6.187 234	0.537 315	−2.831 638
H	2.833 841	0.506 754	−1.740 048
H	2.073 806	−2.149 220	−2.013 691
H	−0.310 148	−1.397 425	−3.216 285
C	−1.918 573	0.048 057	−1.539 165
C	−2.205 084	1.393 511	−1.799 869
C	−3.530 718	1.771 982	−2.018 056
C	−4.554 351	0.822 836	−1.968 373
C	−4.248 424	−0.521 502	−1.744 086
C	−2.933 420	−0.927 307	−1.545 420
C	−2.596 813	−2.402 179	−1.438 449
C	−3.623 587	−3.205 169	−0.658 000
C	−4.326 558	−4.274 085	−1.218 760
C	−5.199 264	−5.038 283	−0.442 310
C	−5.374 706	−4.746 850	0.907 491
C	−4.686 954	−3.671 402	1.471 994
C	−3.826 837	−2.903 204	0.694 285
H	−3.302 490	−2.054 533	1.142 923
H	−4.819 307	−3.427 053	2.527 719
H	−6.047 686	−5.351 839	1.519 760
H	−5.738 496	−5.872 003	−0.898 698
H	−4.179 642	−4.522 750	−2.271 470
C	−2.299 559	−2.999 127	−2.806 570
C	−2.925 406	−2.554 670	−3.974 386
C	−2.622 410	−3.132 015	−5.208 852
C	−1.687 945	−4.161 172	−5.293 350
C	−1.059 160	−4.612 149	−4.131 886
C	−1.363 201	−4.035 636	−2.902 804
H	−0.856 859	−4.380 836	−1.999 339
H	−0.317 762	−5.413 430	−4.183 399
H	−1.446 184	−4.607 976	−6.260 687
H	−3.120 096	−2.768 115	−6.111 157
H	−3.649 871	−1.739 214	−3.923 764
H	−1.663 882	−2.487 732	−0.857 970
H	−5.061 890	−1.248 356	−1.712 501
O	−5.861 902	1.123 539	−2.104 712
C	−6.224 070	2.461 858	−2.321 183
H	−7.318 543	2.482 252	−2.393 184
H	−5.905 480	3.111 602	−1.487 563
H	−5.794 824	2.855 037	−3.258 772
H	−3.745 298	2.822 912	−2.204 563
C	−1.075 829	2.404 648	−1.828 024
C	−1.573 673	3.838 014	−1.829 619
C	−1.698 948	4.577 686	−3.010 027
C	−2.204 272	5.877 125	−2.984 793
C	−2.584 531	6.459 289	−1.777 026
C	−2.457 868	5.731 949	−0.594 127
C	−1.959 888	4.431 649	−0.622 487
H	−1.869 067	3.871 919	0.312 032
H	−2.737 676	6.173 662	0.364 296
H	−2.971 234	7.481 108	−1.756 230
H	−2.292 478	6.440 734	−3.916 991
H	−1.388 911	4.134 133	−3.958 735
C	−0.078 491	2.162 261	−2.944 266
C	−0.447 069	1.584 793	−4.162 440
C	0.477 844	1.465 476	−5.200 132
C	1.782 579	1.926 095	−5.032 783
C	2.164 393	2.489 943	−3.814 097
C	1.240 237	2.602 078	−2.778 963
H	1.544 048	3.044 996	−1.826 996
H	3.189 761	2.835 626	−3.659 584
H	2.505 080	1.840 367	−5.848 185
H	0.173 325	1.013 807	−6.147 615
H	−1.470 585	1.225 559	−4.300 125
H	−0.536 601	2.248 443	−0.879 606
O	−0.823 433	1.028 441	3.546 660
C	−1.727 358	1.654 967	2.874 708
C	−3.000 460	0.910 175	2.604 328
C	−3.823 141	1.161 841	1.495 985
C	−5.045 442	0.507 822	1.363 501
C	−5.457 772	−0.410 717	2.328 841
C	−4.626 411	−0.699 990	3.410 456
C	−3.399 740	−0.055 338	3.541 195
H	−2.738 752	−0.261 927	4.385 444
H	−4.938 791	−1.427 380	4.164 179
H	−6.418 873	−0.918 727	2.222 611
H	−5.678 658	0.709 314	0.497 456
H	−3.495 583	1.865 915	0.728 939
C	−1.766 525	3.147 131	2.981 348
C	−0.632 111	3.809 562	3.476 072
C	−0.638 235	5.185 959	3.663 402
C	−1.786 375	5.930 583	3.389 094
C	−2.931 795	5.279 825	2.935 929
C	−2.922 893	3.900 807	2.732 383
H	−3.837 566	3.407 741	2.403 049
H	−3.848 557	5.844 997	2.749 460
H	−1.792 119	7.012 363	3.543 940
H	0.263 539	5.680 311	4.031 877
H	0.254 320	3.221 597	3.713 762

Intermediate VI

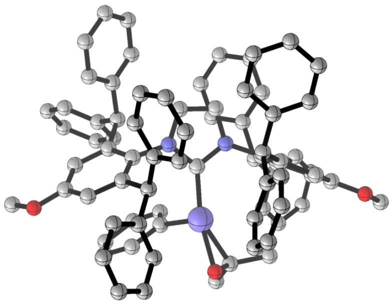

Rh	0.542 321	−0.122 802	−1.921 189
C	−1.254 386	0.377 468	−2.488 983
C	−1.957 196	−0.537 778	−3.292 181
C	−3.189 344	−0.205 449	−3.855 181
C	−3.744 381	1.057 568	−3.650 010
C	−3.053 841	1.979 079	−2.864 922
C	−1.835 717	1.637 956	−2.275 033
H	−1.317 897	2.375 249	−1.656 385
H	−3.472 258	2.976 527	−2.706 737
H	−4.702 853	1.324 951	−4.101 824
H	−3.714 662	−0.945 241	−4.466 163
H	−1.533 778	−1.524 794	−3.491 787
C	0.100 924	0.176 923	−0.012 210
N	0.943 595	0.722 218	0.912 335
C	0.518 186	0.470 836	2.205 426
C	−0.619 263	−0.253 968	2.101 900
N	−0.874 355	−0.408 497	0.746 668
C	−1.996 000	−1.088 972	0.180 991
C	−3.262 929	−0.491 616	0.259 311
C	−4.320 147	−1.055 008	−0.453 164
C	−4.126 358	−2.220 690	−1.194 189
C	−2.888 800	−2.868 631	−1.162 346
C	−1.806 097	−2.312 359	−0.493 765
C	−0.451 797	−3.005 716	−0.502 080
C	−0.075 021	−3.628 960	0.833 826
C	−0.925 158	−3.637 158	1.942 583
C	−0.541 242	−4.259 337	3.132 850
C	0.695 890	−4.891 070	3.228 012
C	1.554 563	−4.885 094	2.126 962
C	1.173 681	−4.259 578	0.945 063
H	1.848 874	−4.267 905	0.085 958
H	2.535 257	−5.362 140	2.189 536
H	0.995 744	−5.381 311	4.157 257
H	−1.223 401	−4.251 963	3.986 864
H	−1.905 381	−3.160 454	1.875 724
C	−0.305 938	−4.004 696	−1.634 688
C	−0.716 751	−5.334 504	−1.486 540
C	−0.610 250	−6.232 576	−2.546 238
C	−0.084 109	−5.815 312	−3.768 758
C	0.335 880	−4.495 326	−3.921 359
C	0.225 995	−3.596 629	−2.861 293
H	0.563 406	−2.563 029	−2.991 059
H	0.757 303	−4.154 443	−4.870 122
H	0.002 981	−6.520 887	−4.598 680
H	−0.937 179	−7.267 064	−2.414 586
H	−1.117 801	−5.669 455	−0.526 503
H	0.300 309	−2.216 273	−0.710 854
H	−2.776 879	−3.791 927	−1.730 132
O	−5.076 854	−2.786 731	−1.959 704
C	−6.296 137	−2.112 941	−2.115 970
H	−6.850 968	−2.039 282	−1.163 802
H	−6.146 726	−1.096 681	−2.519 511
H	−6.890 615	−2.700 551	−2.826 808
H	−5.285 954	−0.553 757	−0.436 182
C	−3.499 322	0.680 407	1.198 169
C	−3.620 894	0.197 555	2.639 212
C	−3.326 678	1.085 998	3.680 086
C	−3.405 199	0.680 405	5.008 382
C	−3.788 545	−0.624 676	5.322 697
C	−4.095 739	−1.513 402	4.295 423

C	−4.012 277	−1.105 065	2.962 700
H	−4.241 274	−1.813 552	2.163 053
H	−4.399 559	−2.537 136	4.528 077
H	−3.845 469	−0.946 295	6.365 267
H	−3.156 226	1.385 627	5.805 235
H	−3.003 581	2.100 433	3.437 650
C	−4.684 174	1.553 612	0.830 775
C	−4.485 744	2.713 373	0.077 866
C	−5.557 247	3.527 378	−0.282 983
C	−6.852 267	3.189 952	0.105 590
C	−7.062 865	2.040 493	0.866 242
C	−5.986 874	1.233 947	1.231 781
H	−6.157 502	0.349 544	1.850 568
H	−8.072 788	1.773 997	1.187 611
H	−7.694 949	3.826 034	−0.175 515
H	−5.378 219	4.430 457	−0.871 874
H	−3.473 296	2.970 827	−0.235 158
H	−2.607 129	1.324 502	1.152 581
H	−1.262 303	−0.675 620	2.865 179
H	1.070 454	0.813 159	3.073 555
C	2.235 217	1.218 118	0.571 558
C	2.392 293	2.544 174	0.151 205
C	3.667 997	2.992 855	−0.187 008
C	4.761 099	2.119 791	−0.144 875
C	4.579 571	0.790 840	0.249 084
C	3.323 214	0.331 366	0.632 607
C	3.119 280	−1.087 095	1.127 806
C	3.785 667	−2.126 179	0.242 714
C	4.637 015	−3.114 461	0.738 641
C	5.123 414	−4.117 185	−0.103 443
C	4.761 068	−4.147 795	−1.447 835
C	3.916 566	−3.158 423	−1.955 193
C	3.442 954	−2.157 725	−1.115 291
H	2.778 708	−1.396 106	−1.535 886
H	3.611 211	−3.163 939	−3.003 717
H	5.132 680	−4.941 063	−2.100 582
H	5.785 257	−4.886 569	0.302 099
H	4.905 542	−3.113 248	1.797 005
C	3.433 633	−1.217 352	2.607 704
C	4.609 127	−0.712 801	3.174 508
C	4.870 011	−0.874 278	4.534 205
C	3.957 178	−1.540 284	5.351 241
C	2.780 946	−2.042 208	4.797 234
C	2.522 830	−1.878 800	3.438 163
H	1.597 559	−2.269 976	3.011 748
H	2.053 495	−2.565 327	5.422 813
H	4.161 810	−1.664 123	6.417 470
H	5.793 899	−0.473 213	4.958 252
H	5.329 424	−0.180 653	2.550 250
H	2.045 733	−1.309 165	1.039 247
H	5.437 959	0.117 255	0.224 733
O	6.012 678	2.474 545	−0.488 257
C	6.240 815	3.776 295	−0.955 441
H	5.995 751	4.535 685	−0.192 401
H	7.310 230	3.841 502	−1.191 537
H	5.657 585	3.991 659	−1.867 658
H	3.786 173	4.020 156	−0.527 336
C	1.158 911	3.416 423	0.001 100
C	1.424 905	4.705 711	−0.748 719
C	1.935 607	5.839 296	−0.106 536
C	2.217 798	6.996 799	−0.829 061
C	1.985 053	7.041 594	−2.203 793
C	1.461 863	5.922 885	−2.849 463
C	1.184 437	4.765 523	−2.124 110
H	0.767 967	3.889 897	−2.627 183
H	1.263 018	5.949 756	−3.923 586
H	2.201 903	7.951 486	−2.768 450
H	2.615 476	7.874 097	−0.312 838
H	2.105 459	5.815 776	0.972 784
C	0.434 379	3.678 340	1.307 075
C	1.095 332	3.723 498	2.537 044
C	0.393 741	4.004 974	3.709 846
C	−0.977 411	4.248 858	3.667 113
C	−1.645 008	4.212 637	2.441 781
C	−0.941 239	3.928 964	1.275 392
H	−1.462 330	3.910 684	0.314 690
H	−2.720 179	4.403 994	2.390 824
H	−1.526 050	4.468 444	4.586 176
H	0.924 638	4.032 780	4.664 671
H	2.169 154	3.521 016	2.577 902
H	0.477 654	2.819 632	−0.628 596
O	1.475 290	−0.564 959	−3.688 663
C	1.560 561	0.723 591	−3.591 219
C	2.879 248	1.328 092	−3.170 553
C	0.739 151	1.561 960	−4.540 944
H	1.345 041	1.760 701	−5.443 613
H	−0.174 806	1.033 993	−4.835 589
H	0.461 233	2.531 953	−4.107 206
H	3.568 907	1.318 843	−4.034 773
H	2.761 921	2.369 886	−2.843 163
H	3.352 346	0.753 205	−2.362 424

Intermediate VII

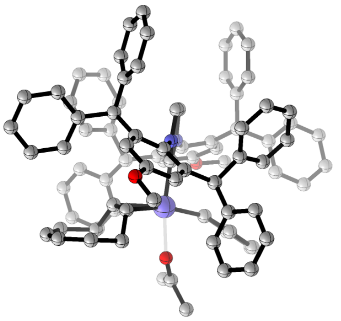

C	−6.631 753	−3.150 304	−0.987 826
O	−5.327 032	−3.607 135	−1.216 191
C	−4.301 825	−2.909 787	−0.689 758
C	−3.016 255	−3.372 563	−0.991 741
C	−1.889 743	−2.696 673	−0.539 266
C	−2.058 030	−1.546 532	0.254 573
C	−3.340 975	−1.109 201	0.618 573
C	−4.457 041	−1.785 575	0.119 112
H	−5.445 061	−1.401 394	0.370 614
C	−3.551 320	0.034 784	1.597 013
C	−4.800 982	0.848 031	1.296 203
C	−5.921 399	0.846 746	2.132 056
C	−7.042 024	1.619 176	1.820 984
C	−7.054 915	2.406 047	0.672 235
C	−5.940 613	2.410 198	−0.168 327
C	−4.826 297	1.636 783	0.140 218
H	−3.961 641	1.639 844	−0.525 794
H	−5.931 563	3.017 848	−1.075 932
H	−7.929 556	3.015 532	0.431 702
H	−7.907 561	1.607 396	2.488 286
H	−5.916 165	0.244 690	3.042 908

C	−3.530 734	−0.452 376	3.038 389
C	−3.068 692	0.410 571	4.038 975
C	−3.030 276	0.005 528	5.369 263
C	−3.457 086	−1.274 375	5.726 991
C	−3.924 689	−2.138 941	4.740 352
C	−3.961 578	−1.730 367	3.405 767
H	−4.315 040	−2.422 416	2.638 198
H	−4.260 321	−3.144 388	5.006 735
H	−3.420 771	−1.596 636	6.770 407
H	−2.653 247	0.692 057	6.131 475
H	−2.715 543	1.405 754	3.761 574
H	−2.702 197	0.727 340	1.482 403
N	−0.920 936	−0.863 850	0.782 499
C	−0.697 149	−0.890 272	2.151 220
C	0.298 772	−0.023 563	2.401 755
N	0.715 416	0.471 752	1.176 107
C	−0.037 797	−0.029 632	0.129 837
Rh	0.166 615	0.405 954	−1.779 533
C	1.915 521	−0.839 262	−2.085 571
C	2.378 684	0.452 084	−1.857 756
C	3.061 857	1.271 859	−2.913 115
H	2.647 535	1.087 668	−3.912 537
H	2.908 055	2.342 555	−2.706 082
C	4.564 774	1.014 944	−2.923 056
C	5.123 882	−0.320 960	−3.322 452
C	4.650 112	−1.547 833	−3.600 069
C	3.316 891	−2.240 718	−3.639 075
H	3.262 726	−2.778 184	−4.603 672
H	3.368 749	−3.054 249	−2.888 829
C	2.007 269	−1.485 901	−3.437 150
H	1.882 913	−0.733 783	−4.229 276
H	1.189 895	−2.210 494	−3.565 070
H	5.446 388	−2.263 104	−3.849 958
H	6.219 092	−0.264 463	−3.388 720
H	4.970 756	1.241 686	−1.917 501
H	5.043 754	1.763 098	−3.581 790
H	2.711 780	0.704 159	−0.854 453
H	1.933 151	−1.536 386	−1.242 268
O	0.307 233	1.315 111	−3.751 188
C	−0.331 240	1.281 875	−4.793 099
C	−1.809 849	0.944 756	−2.044 107
C	−2.146 972	2.295 297	−2.292 361
C	−3.349 139	2.680 174	−2.894 408
C	−4.291 141	1.720 172	−3.266 208
C	−4.009 469	0.379 302	−3.007 238
C	−2.800 755	0.010 099	−2.411 059
H	−2.627 336	−1.055 096	−2.254 227
H	−4.734 560	−0.395 925	−3.273 705
H	−5.234 271	2.013 285	−3.734 758
H	−3.547 464	3.742 259	−3.070 119
H	−1.435 309	3.089 277	−2.050 133
C	−0.378 175	2.499 350	−5.663 951
C	−1.113 622	0.084 449	−5.215 064
C	1.874 414	1.297 905	1.103 264
C	1.770 649	2.615 962	0.638 572
C	2.931 040	3.379 731	0.505 664
C	4.178 026	2.844 550	0.836 051
C	4.261 610	1.544 781	1.341 426
C	3.122 120	0.759 867	1.486 663
C	3.246 906	−0.646 439	2.050 288
C	4.519 889	−1.358 635	1.610 332
C	4.761 166	−1.543 633	0.241 708
C	5.884 288	−2.236 282	−0.198 217
C	6.793 948	−2.758 465	0.722 891
C	6.569 746	−2.572 152	2.083 599
C	5.441 168	−1.877 389	2.523 740
H	5.274 494	−1.744 497	3.594 141
H	7.276 566	−2.969 614	2.816 305
H	7.676 022	−3.303 687	0.378 370
H	6.043 635	−2.357 876	−1.271 689
H	4.066 448	−1.134 409	−0.495 084
C	3.068 112	−0.666 002	3.560 513
C	3.491 789	0.385 556	4.377 302
C	3.318 451	0.326 462	5.761 152
C	2.717 711	−0.784 873	6.347 502
C	2.292 457	−1.840 194	5.539 271
C	2.467 147	−1.779 362	4.160 293
H	2.117 347	−2.598 992	3.528 975
H	1.811 209	−2.714 428	5.984 818
H	2.575 874	−0.827 571	7.430 053
H	3.652 936	1.160 523	6.383 052
H	3.952 772	1.267 244	3.927 021
H	2.417 810	−1.241 717	1.633 365
H	5.245 941	1.140 789	1.583 429
O	5.341 247	3.508 451	0.694 092
C	5.315 177	4.794 336	0.137 005
H	4.742 895	5.502 154	0.761 828
H	4.880 864	4.791 406	−0.878 032
H	6.357 369	5.132 710	0.080 009
H	2.841 537	4.393 971	0.120 686
C	0.414 881	3.181 443	0.262 823
C	0.532 430	4.426 769	−0.593 621
C	0.391 111	5.714 511	−0.069 802
C	0.562 135	6.835 486	−0.883 522
C	0.874 294	6.683 474	−2.232 726
C	1.009 565	5.400 454	−2.765 388
C	0.839 346	4.282 830	−1.953 336
H	0.934 605	3.276 266	−2.371 108
H	1.246 501	5.269 414	−3.824 627
H	1.005 275	7.561 626	−2.869 764
H	0.445 916	7.835 397	−0.457 881
H	0.139 240	5.839 976	0.985 876
C	−0.498 014	3.383 085	1.454 638
C	−1.881 587	3.444 496	1.241 876
C	−2.755 044	3.713 932	2.291 581
C	−2.257 005	3.920 495	3.579 481
C	−0.884 530	3.838 860	3.806 952
C	−0.011 211	3.572 288	2.751 384
H	1.064 540	3.515 278	2.936 028
H	−0.486 023	3.988 071	4.813 708
H	−2.940 850	4.132 367	4.405 003
H	−3.830 648	3.747 549	2.101 801
H	−2.273 701	3.283 639	0.234 866
H	−0.049 067	2.406 690	−0.382 407
H	0.758 169	0.286 399	3.332 389
H	−1.279 295	−1.516 980	2.815 024
C	−0.491 411	−3.167 999	−0.887 669
C	−0.472 037	−4.081 081	−2.100 077
C	−0.892 113	−3.561 845	−3.333 243
C	−0.891 466	−4.346 475	−4.480 592
C	−0.468 553	−5.675 865	−4.417 532
C	−0.056 894	−6.204 734	−3.197 905

C	−0.060 086	−5.413 587	−2.046 717
H	0.266 494	−5.839 041	−1.095 731
H	0.271 461	−7.245 151	−3.135 296
H	−0.463 970	−6.295 846	−5.317 172
H	−1.221 427	−3.918 870	−5.430 777
H	−1.232 345	−2.523 822	−3.376 434
C	0.247 777	−3.745 543	0.304 794
C	−0.423 402	−4.279 782	1.408 442
C	0.284 324	−4.847 384	2.468 560
C	1.676 962	−4.890 107	2.442 396
C	2.360 362	−4.354 199	1.349 770
C	1.648 593	−3.791 713	0.293 933
H	2.194 811	−3.395 956	−0.565 088
H	3.452 780	−4.362 316	1.320 177
H	2.231 246	−5.333 480	3.273 127
H	−0.260 493	−5.260 435	3.321 198
H	−1.515 382	−4.251 568	1.436 731
H	0.043 448	−2.249 826	−1.194 702
H	−2.920 024	−4.259 767	−1.617 832
H	−6.777 847	−2.124 129	−1.368 258
H	−7.302 340	−3.831 658	−1.526 435
H	−6.892 061	−3.167 362	0.085 254
H	−0.848 673	−0.776 886	−4.592 121
H	−2.184 647	0.301 483	−5.067 926
H	−0.953 692	−0.133 797	−6.281 539
H	0.374 250	3.229 146	−5.341 655
H	−0.251 589	2.251 351	−6.727 656
H	−1.379 030	2.948 121	−5.544 746

Intermediate VIII

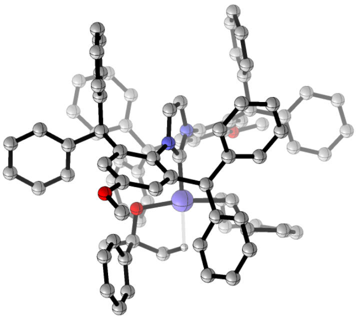

C	6.060 785	3.238 452	2.145 597
O	5.804 818	1.861 503	2.060 536
C	4.538 649	1.457 336	1.827 610
C	4.328 899	0.079 615	1.741 524
C	3.056 241	−0.436 874	1.517 743
C	1.983 230	0.456 786	1.331 871
C	2.187 979	1.839 754	1.374 415
C	3.467 719	2.329 548	1.639 767
H	3.613 056	3.408 062	1.661 107
C	1.035 971	2.794 514	1.143 738
C	1.523 728	4.122 977	0.594 798
C	2.189 262	4.118 025	−0.641 032
C	2.676 971	5.296 162	−1.193 518
C	2.503 115	6.508 442	−0.520 818
C	1.845 979	6.523 824	0.706 166
C	1.361 737	5.337 130	1.263 384
H	0.850 633	5.358 838	2.228 328
H	1.708 266	7.466 347	1.242 089
H	2.882 731	7.437 480	−0.953 129
H	3.199 754	5.260 717	−2.152 339
H	2.342 090	3.169 261	−1.162 734
C	0.111 543	2.912 378	2.338 782
C	0.495 949	2.525 663	3.625 795
C	−0.378 114	2.663 439	4.705 256
C	−1.653 998	3.189 214	4.515 353
C	−2.053 549	3.573 443	3.233 932
C	−1.176 855	3.437 492	2.161 740
H	−1.489 633	3.760 764	1.167 043
H	−3.055 874	3.975 111	3.065 976
H	−2.339 349	3.294 280	5.359 787
H	−0.055 335	2.356 473	5.703 259
H	1.494 637	2.111 998	3.784 772
H	0.445 553	2.338 113	0.323 573
N	0.662 587	−0.087 390	1.233 411
C	−0.182 289	−0.144 611	0.155 050
N	−1.201 569	−0.948 434	0.592 547
C	−0.992 024	−1.375 031	1.895 296
C	0.157 854	−0.808 217	2.305 998
H	0.682 209	−0.864 244	3.252 791
H	−1.682 371	−2.040 337	2.399 781
C	−2.370 307	−1.331 441	−0.128 210
C	−2.262 850	−2.220 044	−1.216 650
C	−3.419 784	−2.561 854	−1.904 898
C	−4.665 982	−2.046 113	−1.529 110
C	−4.757 382	−1.178 013	−0.441 090
C	−3.610 418	−0.810 437	0.268 643
C	−3.727 725	0.140 505	1.450 231
C	−4.866 361	1.139 541	1.288 729
C	−5.923 754	1.220 843	2.198 617
C	−6.947 206	2.154 481	2.022 861
C	−6.928 014	3.020 979	0.934 187
C	−5.877 040	2.946 620	0.018 484
C	−4.857 804	2.016 511	0.194 752
H	−4.049 668	1.965 999	−0.538 514
H	−5.841 609	3.612 543	−0.846 244
H	−7.728 295	3.752 205	0.796 705
H	−7.763 850	2.200 848	2.747 644
H	−5.948 467	0.549 815	3.059 138
C	−3.798 196	−0.605 248	2.771 235
C	−3.211 719	−0.036 092	3.906 522
C	−3.250 384	−0.692 451	5.132 815
C	−3.880 010	−1.932 762	5.244 954
C	−4.473 301	−2.504 499	4.121 197
C	−4.433 419	−1.844 129	2.892 353
H	−4.887 388	−2.306 243	2.012 580
H	−4.967 541	−3.476 181	4.197 381
H	−3.903 818	−2.453 976	6.204 894
H	−2.773 866	−0.236 456	6.004 220
H	−2.699 072	0.924 372	3.819 205
H	−2.798 652	0.734 201	1.480 900
H	−5.711 100	−0.743 793	−0.143 573
O	−5.717 708	−2.429 771	−2.275 311
C	−6.979 965	−1.896 332	−1.981 269
H	−6.987 366	−0.795 167	−2.061 592
H	−7.319 532	−2.178 226	−0.969 102
H	−7.676 799	−2.313 847	−2.718 674
H	−3.375 437	−3.225 983	−2.768 564
C	−0.910 192	−2.761 992	−1.642 735
C	−0.364 767	−3.844 606	−0.734 244
C	0.997 752	−4.157 302	−0.832 776
C	1.541 136	−5.219 667	−0.118 689
C	0.725 498	−5.987 546	0.715 813
C	−0.624 597	−5.667 519	0.843 098
C	−1.167 739	−4.601 167	0.122 238

H	−2.230 965	−4.364 927	0.214 020
H	−1.266 510	−6.255 691	1.503 854
H	1.148 476	−6.822 621	1.279 170
H	2.608 776	−5.435 999	−0.204 476
H	1.634 330	−3.562 154	−1.491 905
C	−0.912 011	−3.208 841	−3.093 376
C	−1.050 202	−4.545 932	−3.474 280
C	−1.084 035	−4.897 073	−4.824 318
C	−0.975 268	−3.918 355	−5.810 153
C	−0.832 654	−2.581 102	−5.438 361
C	−0.803 311	−2.235 442	−4.090 931
H	−0.693 309	−1.194 261	−3.780 026
H	−0.738 491	−1.804 624	−6.201 884
H	−0.996 348	−4.196 941	−6.866 438
H	−1.191 561	−5.947 333	−5.106 477
H	−1.124 018	−5.320 479	−2.707 366
H	−0.205 303	−1.912 316	−1.596 396
Rh	0.102 288	0.720 267	−1.568 276
C	−1.438 554	2.128 061	−1.226 696
C	−1.898 892	1.106 911	−2.084 815
C	−2.166 056	1.360 156	−3.544 964
C	−3.595 115	1.827 750	−3.798 721
C	−4.082 269	3.165 963	−3.323 439
C	−3.631 573	4.151 739	−2.530 003
C	−2.418 883	4.423 048	−1.687 220
H	−2.097 978	5.455 438	−1.915 725
C	−1.185 397	3.527 019	−1.723 308
H	−0.412 527	4.010 882	−1.108 018
H	−0.775 593	3.513 177	−2.745 194
H	−2.780 792	4.483 502	−0.641 484
H	−4.341 118	4.986 168	−2.442 357
H	−5.079 781	3.371 484	−3.735 378
H	−4.287 400	1.068 530	−3.384 760
H	−3.780 971	1.796 070	−4.887 676
H	−1.460 804	2.088 424	−3.972 564
H	−2.030 690	0.424 599	−4.110 860
H	−2.558 378	0.341 705	−1.674 539
H	−1.761 682	2.084 708	−0.179 710
O	1.899 868	−0.137 160	−1.840 508
C	2.378 051	0.393 197	−3.039 266
C	1.231 499	1.184 613	−3.699 561
H	0.457 587	0.505 279	−4.081 225
H	0.803 204	1.943 005	−2.982 259
H	1.543 134	1.822 192	−4.537 401
C	2.808 635	−0.744 962	−3.971 868
H	3.636 125	−1.302 770	−3.511 981
H	1.966 799	−1.436 024	−4.129 489
H	3.154 836	−0.367 341	−4.946 920
C	3.586 369	1.306 173	−2.800 536
C	4.299 588	1.191 397	−1.606 290
C	5.441 836	1.956 422	−1.376 349
C	5.880 220	2.871 018	−2.331 792
C	5.174 407	3.000 216	−3.527 420
C	4.045 043	2.216 954	−3.761 278
H	3.523 999	2.321 370	−4.716 554
H	5.509 058	3.709 482	−4.289 031
H	6.771 423	3.477 027	−2.150 126
H	5.991 392	1.830 390	−0.440 996
H	3.933 512	0.492 891	−0.855 317
C	2.849 834	−1.940 605	1.510 663
C	2.656 956	−2.505 020	2.909 642
C	1.782 262	−3.583 853	3.085 460
C	1.576 111	−4.142 534	4.342 862
C	2.241 795	−3.629 032	5.456 417
C	3.112 355	−2.553 897	5.294 857
C	3.317 113	−1.995 612	4.031 845
H	3.991 436	−1.143 884	3.924 233
H	3.637 288	−2.139 041	6.159 019
H	2.078 243	−4.062 064	6.446 227
H	0.883 095	−4.980 553	4.452 584
H	1.247 697	−3.981 710	2.221 676
C	3.935 759	−2.653 139	0.720 724
C	4.014 483	−2.402 638	−0.656 571
C	4.974 406	−3.044 717	−1.431 809
C	5.869 092	−3.944 148	−0.847 384
C	5.795 650	−4.193 699	0.520 016
C	4.832 120	−3.551 534	1.301 335
H	4.772 474	−3.761 009	2.371 481
H	6.490 703	−4.895 484	0.987 882
H	6.621 373	−4.447 839	−1.459 305
H	5.025 641	−2.844 061	−2.505 048
H	3.305 642	−1.698 495	−1.111 521
H	1.916 549	−2.141 672	0.963 445
H	5.186 905	−0.588 477	1.834 966
H	5.809 055	3.758 930	1.205 160
H	7.135 455	3.345 466	2.339 222
H	5.498 487	3.706 905	2.972 013

Intermediate IX

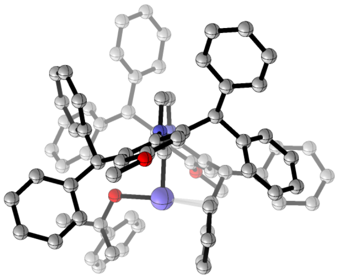

Rh	−0.191 647	0.024 516	−1.636 668
C	−3.402 691	−1.361 503	−2.595 967
C	−3.683 093	−0.168 111	−1.925 922
C	−4.885 610	0.508 455	−2.133 583
C	−5.831 592	0.006 281	−3.023 709
C	−5.562 132	−1.178 551	−3.707 823
C	−4.360 751	−1.850 948	−3.494 654
H	−4.170 699	−2.778 113	−4.041 606
H	−6.294 377	−1.586 069	−4.409 947
H	−6.777 438	0.531 207	−3.179 386
H	−5.094 150	1.424 081	−1.576 279
H	−2.944 046	0.216 749	−1.221 300
C	0.337 225	0.090 103	0.184 237
N	1.424 996	−0.400 946	0.848 657
C	1.309 919	−0.164 755	2.214 104
C	0.167 875	0.530 182	2.407 464
N	−0.417 808	0.677 110	1.159 297
C	−1.612 378	1.405 737	0.903 277
C	−2.819 453	0.995 618	1.495 514
C	−3.977 373	1.707 604	1.202 738
C	−3.943 850	2.818 434	0.355 619
C	−2.734 643	3.223 879	−0.212 729
C	−1.560 071	2.515 645	0.047 185
C	−0.268 509	2.892 244	−0.650 598

C	−0.091 491	2.077 181	−1.925 326
C	1.198 861	1.527 584	−2.257 447
C	1.515 868	1.213 717	−3.620 028
C	0.552 651	1.282 740	−4.591 204
C	−0.761 446	1.743 270	−4.254 171
C	−1.061 501	2.180 906	−2.988 820
H	−2.056 683	2.570 333	−2.765 663
H	−1.534 442	1.768 039	−5.026 081
H	0.775 578	0.998 881	−5.621 799
H	2.535 024	0.902 664	−3.861 764
H	2.028 107	1.663 369	−1.563 508
C	−0.102 446	4.388 273	−0.842 999
C	−0.174 117	5.223 040	0.281 176
C	0.050 389	6.591 778	0.173 904
C	0.347 603	7.156 387	−1.068 504
C	0.415 486	6.337 464	−2.192 242
C	0.195 379	4.963 265	−2.080 371
H	0.273 963	4.330 897	−2.966 044
H	0.649 296	6.766 946	−3.169 562
H	0.524 676	8.230 920	−1.156 704
H	−0.004 467	7.223 154	1.064 228
H	−0.406 222	4.784 822	1.256 007
H	0.561 545	2.592 846	0.009 211
H	−2.681 871	4.092 896	−0.867 923
O	−5.125 477	3.423 135	0.118 589
C	−5.155 419	4.521 795	−0.751 515
H	−4.530 328	5.353 721	−0.383 075
H	−4.819 559	4.246 248	−1.766 689
H	−6.199 696	4.854 533	−0.800 648
H	−4.939 723	1.386 786	1.605 002
C	−2.866 348	−0.198 452	2.430 480
C	−4.084 193	−1.062 579	2.150 313
C	−4.114 812	−1.771 597	0.942 691
C	−5.226 475	−2.533 766	0.599 653
C	−6.317 156	−2.611 345	1.467 480
C	−6.286 584	−1.920 588	2.676 517
C	−5.175 368	−1.146 560	3.017 126
H	−5.157 044	−0.605 745	3.966 268
H	−7.135 230	−1.978 959	3.362 730
H	−7.191 371	−3.209 387	1.198 871
H	−5.242 819	−3.058 427	−0.358 875
H	−3.262 705	−1.701 239	0.259 146
C	−2.725 294	0.195 320	3.891 352
C	−3.142 179	1.434 533	4.386 452
C	−3.008 225	1.743 585	5.741 090
C	−2.450 637	0.820 044	6.621 507
C	−2.024 926	−0.417 247	6.136 617
C	−2.161 668	−0.723 999	4.786 229
H	−1.812 475	−1.687 073	4.408 140
H	−1.577 250	−1.148 081	6.814 731
H	−2.342 411	1.064 017	7.681 014
H	−3.340 869	2.718 015	6.107 308
H	−3.570 896	2.171 870	3.704 771
H	−1.996 406	−0.828 203	2.190 191
H	−0.295 066	0.911 635	3.312 192
H	2.044 806	−0.537 702	2.919 768
C	2.599 201	−1.004 597	0.299 234
C	3.831 791	−0.330 296	0.417 557
C	4.990 981	−0.971 771	−0.010 568
C	4.943 334	−2.257 244	−0.552 045
C	3.717 897	−2.919 642	−0.644 517
C	2.537 981	−2.308 317	−0.217 599
C	1.218 914	−3.052 652	−0.247 845
C	0.704 285	−3.344 415	1.146 530
C	1.551 798	−3.735 611	2.187 759
C	1.031 249	−4.050 330	3.443 168
C	−0.343 834	−3.986 499	3.667 478
C	−1.194 282	−3.590 475	2.633 943
C	−0.671 874	−3.260 128	1.385 455
H	−1.314 841	−2.918 670	0.568 740
H	−2.274 376	−3.531 733	2.795 780
H	−0.752 143	−4.245 139	4.647 985
H	1.703 311	−4.356 705	4.249 016
H	2.629 430	−3.797 377	2.011 302
C	1.243 016	−4.276 482	−1.143 933
C	1.522 279	−4.105 676	−2.508 516
C	1.509 326	−5.185 021	−3.385 076
C	1.212 462	−6.465 520	−2.913 928
C	0.935 638	−6.648 138	−1.562 402
C	0.951 489	−5.562 380	−0.684 155
H	0.721 120	−5.716 115	0.371 733
H	0.698 593	−7.644 755	−1.181 834
H	1.194 518	−7.315 011	−3.600 835
H	1.722 141	−5.025 619	−4.445 188
H	1.755 611	−3.103 658	−2.878 738
H	0.463 507	−2.387 859	−0.710 313
H	3.660 454	−3.932 936	−1.037 607
O	6.116 413	−2.789 018	−0.941 979
C	6.120 974	−4.080 372	−1.488 067
H	5.767 811	−4.833 906	−0.762 661
H	7.161 397	−4.304 033	−1.755 063
H	5.494 627	−4.144 750	−2.394 788
H	5.963 579	−0.488 988	0.098 200
C	3.945 836	1.070 298	0.997 979
C	5.131 729	1.183 427	1.949 288
C	5.231 776	0.281 235	3.017 654
C	6.280 392	0.360 661	3.927 032
C	7.258 936	1.345 733	3.782 377
C	7.172 628	2.243 688	2.723 138
C	6.115 523	2.164 829	1.814 158
H	6.056 187	2.877 860	0.989 667
H	7.933 902	3.017 454	2.597 219
H	8.086 160	1.408 513	4.493 248
H	6.338 438	−0.353 292	4.752 264
H	4.483 984	−0.509 410	3.125 075
C	3.927 235	2.159 571	−0.057 425
C	3.280 807	3.366 897	0.233 722
C	3.248 211	4.406 631	−0.692 275
C	3.867 861	4.252 064	−1.931 237
C	4.524 083	3.059 407	−2.229 019
C	4.554 695	2.020 576	−1.299 496
H	5.052 033	1.082 516	−1.552 778
H	5.008 305	2.929 098	−3.199 902
H	3.828 655	5.059 252	−2.666 018
H	2.720 881	5.332 622	−0.452 987
H	2.788 644	3.490 160	1.202 980
H	3.054 216	1.249 158	1.616 453
O	−1.371 639	−1.586 338	−1.278 396
C	−2.071 078	−2.099 432	−2.370 487
C	−1.244 917	−1.963 126	−3.660 409
H	−0.299 605	−2.512 503	−3.558 757
H	−1.783 146	−2.347 063	−4.540 334

H	−1.023 680	−0.902 049	−3.872 582
C	−2.332 612	−3.584 861	−2.089 166
H	−2.935 074	−3.696 556	−1.175 912
H	−1.374 021	−4.099 886	−1.934 337
H	−2.861 324	−4.085 427	−2.913 593

Intermediate X

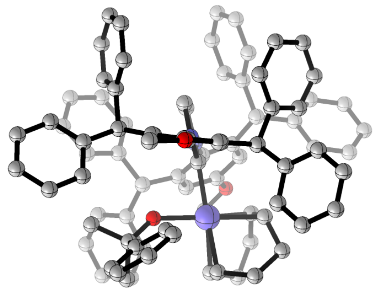

Rh	0.007 132	0.177 878	2.113 650
C	−1.003 750	−1.569 325	2.814 535
C	−1.914 674	−0.528 665	2.545 294
C	−2.605 134	0.337 234	3.564 426
C	−1.646 197	0.821 267	4.653 505
C	−0.253 516	1.032 292	4.104 294
C	0.769 003	0.093 279	4.150 568
C	0.633 316	−1.288 823	4.726 488
C	−0.609 099	−2.017 838	4.211 076
H	−0.419 653	−3.101 054	4.199 461
H	−1.458 287	−1.870 223	4.897 189
H	0.633 206	−1.237 827	5.831 685
H	1.537 329	−1.854 281	4.457 101
H	1.796 821	0.455 107	4.058 995
H	0.047 141	2.069 373	3.958 854
H	−2.012 078	1.768 833	5.078 481
H	−1.612 416	0.108 936	5.491 404
H	−3.020 675	1.204 140	3.027 301
H	−3.472 764	−0.193 132	4.001 115
H	−2.479 203	−0.612 856	1.617 831
H	−0.971 728	−2.372 205	2.074 163
C	−0.239 500	−0.117 288	0.050 175
N	−1.289 418	0.370 519	−0.679 532
C	−1.144 417	0.119 009	−2.034 281
C	0.001 085	−0.573 332	−2.171 394
N	0.567 942	−0.676 629	−0.908 462
C	1.851 395	−1.312 923	−0.796 199
C	2.991 235	−0.704 604	−1.355 666
C	4.199 469	−1.407 780	−1.351 695
C	4.292 117	−2.684 438	−0.804 574
C	3.144 713	−3.293 837	−0.295 312
C	1.926 833	−2.625 845	−0.292 725
C	0.688 045	−3.320 712	0.226 978
C	1.035 822	−4.325 401	1.312 600
C	0.822 746	−5.699 157	1.186 201
C	1.197 212	−6.571 960	2.210 321
C	1.790 454	−6.083 270	3.370 778
C	2.012 903	−4.711 100	3.502 688
C	1.639 408	−3.845 348	2.481 375
H	1.821 217	−2.771 981	2.575 907
H	2.480 085	−4.313 189	4.407 251
H	2.082 612	−6.767 866	4.170 575
H	1.023 520	−7.644 574	2.094 202
H	0.360 958	−6.094 747	0.279 444
C	−0.188 330	−3.907 647	−0.860 703
C	−1.512 244	−4.241 732	−0.543 039
C	−2.339 906	−4.860 320	−1.474 291
C	−1.856 991	−5.145 745	−2.753 353
C	−0.551 210	−4.794 764	−3.088 499
C	0.278 874	−4.181 801	−2.147 700
H	1.305 976	−3.920 287	−2.413 346
H	−0.168 288	−5.004 480	−4.090 306
H	−2.503 697	−5.629 649	−3.489 101
H	−3.369 159	−5.106 619	−1.202 040
H	−1.892 383	−4.034 095	0.460 571
H	0.096 321	−2.533 222	0.713 208
H	3.227 078	−4.299 335	0.118 354
O	5.436 265	−3.386 945	−0.721 036
C	6.633 485	−2.758 977	−1.095 628
H	6.800 853	−1.831 919	−0.520 295
H	7.441 020	−3.469 549	−0.879 174
H	6.652 656	−2.516 836	−2.172 711
H	5.079 319	−0.919 217	−1.765 456
C	2.941 636	0.651 130	−2.045 349
C	2.450 637	0.502 222	−3.478 396
C	1.534 200	1.420 071	−3.998 192
C	1.063 724	1.302 141	−5.303 245
C	1.505 224	0.257 152	−6.114 320
C	2.427 997	−0.658 367	−5.609 580
C	2.899 309	−0.534 231	−4.302 843
H	3.611 754	−1.264 755	−3.913 302
H	2.783 027	−1.480 151	−6.236 527
H	1.130 551	0.154 695	−7.135 620
H	0.336 378	2.026 735	−5.677 349
H	1.162 602	2.223 839	−3.361 507
C	4.260 835	1.424 630	−1.998 645
C	4.699 119	2.180 234	−3.092 005
C	5.872 969	2.931 499	−3.023 127
C	6.637 622	2.937 725	−1.860 085
C	6.213 611	2.186 842	−0.764 247
C	5.040 504	1.442 078	−0.834 045
H	4.725 475	0.867 662	0.036 800
H	6.790 815	2.178 865	0.163 360
H	7.558 625	3.523 449	−1.806 993
H	6.190 247	3.512 591	−3.892 641
H	4.117 803	2.184 529	−4.015 301
H	2.194 367	1.257 592	−1.503 051
H	0.471 320	−1.004 941	−3.046 157
H	−1.868 100	0.456 767	−2.765 937
C	−2.478 587	0.985 473	−0.178 010
C	−2.457 358	2.314 582	0.257 497
C	−3.639 826	2.877 513	0.750 275
C	−4.816 174	2.131 290	0.816 711
C	−4.832 190	0.820 460	0.328 197
C	−3.681 847	0.246 575	−0.197 222
C	−3.750 516	−1.117 556	−0.864 907
C	−4.761 146	−2.053 833	−0.221 197
C	−5.992 076	−2.344 169	−0.815 598
C	−6.898 276	−3.205 976	−0.195 952
C	−6.584 852	−3.795 833	1.025 599
C	−5.357 299	−3.515 063	1.626 735
C	−4.456 999	−2.652 527	1.008 738
H	−3.502 945	−2.435 232	1.494 000

H	−5.096 189	−3.972 761	2.584 053
H	−7.292 373	−4.474 172	1.508 274
H	−7.855 320	−3.419 551	−0.678 247
H	−6.241 972	−1.898 296	−1.780 557
C	−3.972 948	−0.965 411	−2.364 411
C	−4.685 142	0.105 878	−2.911 867
C	−4.849 842	0.219 768	−4.293 545
C	−4.304 126	−0.735 694	−5.147 443
C	−3.596 762	−1.811 141	−4.608 335
C	−3.435 267	−1.923 053	−3.231 221
H	−2.862 424	−2.754 631	−2.818 447
H	−3.155 079	−2.564 723	−5.265 130
H	−4.425 240	−0.641 411	−6.229 215
H	−5.405 739	1.067 217	−4.702 433
H	−5.104 090	0.870 018	−2.253 583
H	−2.766 630	−1.599 284	−0.745 975
H	−5.766 533	0.258 142	0.364 948
O	−5.975 634	2.599 215	1.313 332
C	−6.004 319	3.898 843	1.839 194
H	−5.284 625	4.020 753	2.667 279
H	−5.785 258	4.658 533	1.068 693
H	−7.020 525	4.060 572	2.219 834
H	−3.616 760	3.912 159	1.087 058
C	−1.213 767	3.171 785	0.098 656
C	−1.256 679	4.371 066	1.031 627
C	−1.210 761	4.129 852	2.410 139
C	−1.288 512	5.172 824	3.326 258
C	−1.414 529	6.488 865	2.876 777
C	−1.466 087	6.741 138	1.508 689
C	−1.389 797	5.689 933	0.591 784
H	−1.434 123	5.902 136	−0.478 255
H	−1.566 847	7.766 751	1.144 801
H	−1.471 133	7.312 965	3.592 009
H	−1.243 521	4.958 837	4.397 332
H	−1.122 336	3.096 778	2.748 741
C	−0.992 523	3.576 693	−1.348 828
C	−1.990 322	3.489 045	−2.324 185
C	−1.760 151	3.935 397	−3.626 708
C	−0.532 206	4.494 212	−3.972 046
C	0.472 668	4.584 908	−3.007 201
C	0.246 769	4.119 619	−1.715 019
H	1.039 505	4.182 763	−0.967 890
H	1.447 007	5.006 586	−3.265 972
H	−0.354 658	4.854 341	−4.988 291
H	−2.554 131	3.850 293	−4.372 985
H	−2.964 558	3.068 598	−2.064 708
H	−0.334 788	2.575 963	0.420 349
O	1.305 257	1.609 285	1.463 079
C	2.420 865	2.252 542	1.969 286
C	3.581 285	1.273 800	2.235 066
C	3.426 377	−0.100 996	2.039 976
C	4.480 388	−0.990 685	2.253 520
C	5.724 853	−0.517 437	2.663 016
C	5.895 223	0.851 949	2.873 941
C	4.835 022	1.731 483	2.668 027
H	4.995 742	2.799 431	2.834 887
H	6.863 028	1.238 844	3.204 348
H	6.554 941	−1.209 771	2.824 532
H	4.326 667	−2.059 446	2.085 554
H	2.454 761	−0.469 915	1.703 250
C	2.847 541	3.277 092	0.902 246
H	3.813 625	3.753 555	1.124 663
H	2.079 892	4.064 515	0.854 295
H	2.921 414	2.795 452	−0.079 106
C	2.176 754	3.066 482	3.259 410
H	3.031 783	3.725 818	3.470 845
H	2.032 958	2.434 128	4.143 872
H	1.293 757	3.706 107	3.120 495

Intermediate XI

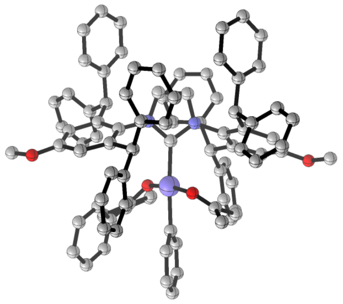

C	−4.644 308	−4.759 592	−2.173 468
O	−3.371 785	−4.424 508	−2.655 798
C	−2.521 762	−3.782 574	−1.831 668
C	−1.312 959	−3.363 029	−2.397 674
C	−0.397 089	−2.621 511	−1.663 361
C	−0.689 233	−2.324 883	−0.316 384
C	−1.855 013	−2.813 918	0.286 944
C	−2.783 342	−3.517 787	−0.487 704
H	−3.706 867	−3.848 698	−0.013 242
C	−2.065 099	−2.686 317	1.783 484
C	−3.525 354	−2.526 708	2.166 348
C	−4.262 218	−3.545 872	2.775 397
C	−5.601 505	−3.347 466	3.115 706
C	−6.216 834	−2.124 576	2.861 105
C	−5.489 133	−1.100 738	2.252 452
C	−4.158 527	−1.306 412	1.902 137
H	−3.592 905	−0.507 916	1.417 510
H	−5.954 526	−0.134 079	2.050 725
H	−7.261 466	−1.965 203	3.139 180
H	−6.162 471	−4.154 964	3.592 952
H	−3.780 368	−4.500 495	2.997 768
C	−1.391 897	−3.829 629	2.530 110
C	−0.962 667	−3.618 261	3.846 177
C	−0.337 578	−4.631 865	4.565 461
C	−0.129 674	−5.882 361	3.981 536
C	−0.553 704	−6.104 532	2.673 907
C	−1.179 420	−5.085 338	1.953 414
H	−1.495 107	−5.267 423	0.923 839
H	−0.393 723	−7.077 915	2.203 427
H	0.366 218	−6.677 685	4.543 087
H	−0.000 263	−4.440 276	5.587 128
H	−1.100 003	−2.633 897	4.297 973
H	−1.560 583	−1.762 838	2.107 672
N	0.236 935	−1.563 101	0.452 475
C	0.999 506	−2.099 614	1.481 582
C	1.741 536	−1.086 908	1.975 169
N	1.444 893	0.031 304	1.208 015
C	0.488 260	−0.230 101	0.261 604
Rh	−0.362 357	1.117 647	−1.071 544
O	−2.183 911	1.129 322	−0.157 597

C	−3.325 193	1.254 392	−0.641 057
C	−3.638 706	0.815 072	−2.015 626
C	−4.444 264	1.576 019	−2.873 273
C	−4.677 516	1.142 799	−4.173 832
C	−4.144 516	−0.069 552	−4.613 521
C	−3.360 616	−0.841 022	−3.755 876
C	−3.088 619	−0.388 820	−2.469 459
H	−2.444 597	−0.960 570	−1.801 695
H	−2.944 485	−1.791 819	−4.093 138
H	−4.335 809	−0.412 799	−5.633 333
H	−5.270 634	1.760 759	−4.851 545
H	−4.835 835	2.537 444	−2.536 951
C	−4.381 194	1.834 627	0.214 271
C	−5.746 366	1.619 163	−0.031 562
C	−6.706 830	2.184 233	0.803 203
C	−6.318 444	2.966 557	1.891 380
C	−4.962 595	3.183 790	2.144 831
C	−4.001 809	2.623 212	1.312 759
H	−2.943 142	2.812 448	1.491 172
H	−4.645 623	3.802 579	2.987 175
H	−7.075 338	3.410 569	2.542 599
H	−7.766 734	2.005 887	0.607 167
H	−6.051 326	0.987 529	−0.867 491
C	−1.140 914	2.534 809	−2.338 292
C	−1.962 768	3.588 657	−1.877 381
C	−2.496 084	4.565 760	−2.720 441
C	−2.241 442	4.528 028	−4.090 947
C	−1.433 104	3.506 178	−4.589 299
C	−0.892 273	2.548 146	−3.728 820
H	−0.258 351	1.773 728	−4.170 352
H	−1.223 619	3.451 788	−5.662 694
H	−2.663 365	5.282 991	−4.759 651
H	−3.121 739	5.360 487	−2.300 769
H	−2.208 746	3.657 153	−0.814 902
C	2.147 892	1.264 478	1.345 589
C	1.491 801	2.395 228	1.873 396
C	2.210 147	3.572 438	2.035 496
C	3.556 288	3.652 327	1.658 912
C	4.195 941	2.529 070	1.136 473
C	3.503 477	1.320 657	0.995 511
C	4.246 794	0.073 750	0.546 060
C	5.342 210	0.371 716	−0.462 079
C	4.994 391	0.553 261	−1.804 587
C	5.963 479	0.831 922	−2.764 515
C	7.303 696	0.944 252	−2.392 854
C	7.661 106	0.769 096	−1.057 922
C	6.688 363	0.478 917	−0.100 558
H	6.980 019	0.318 403	0.939 912
H	8.708 780	0.847 469	−0.757 048
H	8.067 078	1.159 566	−3.144 240
H	5.672 917	0.954 199	−3.811 002
H	3.944 470	0.462 383	−2.089 944
C	4.773 140	−0.727 903	1.726 990
C	5.096 209	−0.140 566	2.953 158
C	5.575 024	−0.918 289	4.009 399
C	5.735 363	−2.292 807	3.853 006
C	5.415 967	−2.885 805	2.630 320
C	4.940 969	−2.109 440	1.578 682
H	4.674 052	−2.581 079	0.630 724
H	5.526 738	−3.964 797	2.496 526
H	6.102 338	−2.902 244	4.682 314
H	5.817 291	−0.443 248	4.963 348
H	4.955 210	0.933 811	3.091 847
H	3.519 975	−0.571 832	0.028 661
H	5.240 944	2.566 083	0.830 501
O	4.152 800	4.848 315	1.830 029
C	5.493 866	4.988 092	1.453 263
H	5.773 949	6.027 723	1.664 645
H	5.641 441	4.788 580	0.377 116
H	6.155 159	4.315 584	2.028 025
H	1.728 990	4.466 458	2.433 167
C	0.022 442	2.297 365	2.223 554
C	−0.650 938	3.645 839	2.384 541
C	−1.195 443	4.090 042	3.592 200
C	−1.851 738	5.320 873	3.664 608
C	−1.974 262	6.121 846	2.531 677
C	−1.427 806	5.688 599	1.322 226
C	−0.770 729	4.465 083	1.253 725
H	−0.362 931	4.113 200	0.303 476
H	−1.524 937	6.298 210	0.420 417
H	−2.495 794	7.080 345	2.588 544
H	−2.272 831	5.651 843	4.617 463
H	−1.117 433	3.462 630	4.482 501
C	−0.253 509	1.365 788	3.381 885
C	−1.488 341	0.709 428	3.440 412
C	−1.824 841	−0.086 265	4.531 742
C	−0.916 601	−0.254 366	5.578 656
C	0.325 146	0.375 940	5.519 658
C	0.651 893	1.184 797	4.430 879
H	1.622 727	1.686 006	4.394 360
H	1.044 674	0.244 252	6.331 728
H	−1.176 182	−0.878 882	6.437 209
H	−2.798 689	−0.582 618	4.555 657
H	−2.194 200	0.839 292	2.617 436
H	−0.439 508	1.846 880	1.320 791
H	2.450 041	−1.058 668	2.794 645
H	0.942 655	−3.143 636	1.766 962
C	0.905 441	−2.160 338	−2.287 745
C	0.774 823	−2.008 339	−3.791 102
C	0.043 417	−0.917 105	−4.273 256
C	−0.164 386	−0.747 991	−5.638 428
C	0.361 031	−1.670 301	−6.545 342
C	1.089 639	−2.760 293	−6.073 434
C	1.294 499	−2.930 394	−4.702 828
H	1.865 813	−3.788 022	−4.339 633
H	1.501 858	−3.489 039	−6.776 022
H	0.200 699	−1.540 287	−7.618 579
H	−0.747 074	0.107 320	−5.990 414
H	−0.375 254	−0.208 130	−3.550 324
C	2.096 246	−3.001 026	−1.867 391
C	1.979 127	−4.185 856	−1.136 417
C	3.111 304	−4.924 721	−0.787 412
C	4.379 090	−4.493 995	−1.169 777
C	4.509 708	−3.309 062	−1.897 783
C	3.379 096	−2.574 486	−2.238 329
H	3.484 719	−1.657 925	−2.822 229
H	5.497 548	−2.948 156	−2.194 720
H	5.264 450	−5.074 226	−0.898 830
H	2.995 539	−5.847 421	−0.213 308
H	0.989 916	−4.538 630	−0.836 031
H	1.077 676	−1.140 605	−1.903 157
H	−1.119 629	−3.598 734	−3.444 831

H	−5.193 965	−3.867 269	−1.827 174
H	−5.188 280	−5.213 868	−3.011 042
H	−4.592 143	−5.488 131	−1.345 268
C	1.826 287	3.606 525	−1.668 735
H	0.962 007	4.167 981	−2.060 973
H	1.601 152	3.348 611	−0.625 436
H	2.725 017	4.234 971	−1.739 892
C	2.803 509	2.370 814	−3.694 477
H	3.818 905	2.737 573	−3.483 610
H	2.834 405	1.376 071	−4.156 736
H	2.339 607	3.078 661	−4.402 190
C	1.962 476	2.354 090	−2.459 511
O	1.366 164	1.320 046	−2.154 651

Intermediate XII

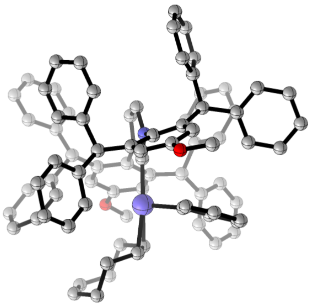

Rh	−0.667 029	0.300 575	−1.705 838
C	0.122 592	0.040 992	0.178 678
N	−0.516 654	0.479 289	1.301 059
C	0.087 888	0.008 581	2.457 878
C	1.135 639	−0.749 436	2.062 397
N	1.156 336	−0.700 002	0.677 113
C	2.119 154	−1.355 194	−0.147 300
C	1.753 677	−2.534 491	−0.819 430
C	2.652 811	−3.107 364	−1.707 276
C	3.909 285	−2.522 887	−1.920 458
C	4.298 139	−1.416 044	−1.164 198
C	3.410 308	−0.835 110	−0.255 337
C	3.874 580	0.246 530	0.697 776
C	4.917 727	1.171 338	0.099 500
C	4.574 115	1.902 191	−1.044 961
C	5.475 659	2.795 929	−1.612 512
C	6.740 003	2.973 012	−1.046 934
C	7.092 606	2.243 765	0.085 572
C	6.186 259	1.347 455	0.656 465
H	6.467 583	0.785 118	1.549 220
H	8.080 827	2.371 383	0.534 588
H	7.448 314	3.676 785	−1.490 939
H	5.182 433	3.353 853	−2.504 643
H	3.590 550	1.762 396	−1.500 576
C	4.302 792	−0.375 088	2.019 991
C	4.074 223	0.321 639	3.212 286
C	4.444 146	−0.220 081	4.440 288
C	5.051 784	−1.474 321	4.499 932
C	5.286 729	−2.176 019	3.319 494
C	4.914 548	−1.630 767	2.090 432
H	5.090 478	−2.196 892	1.173 349
H	5.759 842	−3.160 721	3.352 064
H	5.336 540	−1.904 427	5.463 118
H	4.246 739	0.338 965	5.358 426
H	3.580 484	1.294 242	3.172 837
H	3.003 511	0.883 352	0.920 803
H	5.286 768	−0.971 890	−1.275 663
O	4.686 155	−3.104 832	−2.852 369
C	5.933 102	−2.531 251	−3.137 789
H	5.832 766	−1.481 408	−3.463 780
H	6.611 109	−2.567 481	−2.266 947
H	6.370 742	−3.121 073	−3.952 867
H	2.392 103	−4.010 078	−2.261 474
C	0.445 547	−3.200 668	−0.449 123
C	−0.065 842	−4.153 282	−1.506 271
C	−0.582 689	−3.606 891	−2.687 142
C	−1.066 133	−4.431 157	−3.698 072
C	−1.034 341	−5.818 530	−3.543 501
C	−0.516 725	−6.369 031	−2.373 564
C	−0.035 779	−5.540 691	−1.358 064
H	0.359 470	−5.975 487	−0.437 109
H	−0.489 185	−7.453 871	−2.244 950
H	−1.414 135	−6.468 498	−4.335 457
H	−1.471 819	−3.989 132	−4.611 620
H	−0.600 446	−2.516 730	−2.798 873
C	0.530 156	−3.797 450	0.946 849
C	1.751 972	−4.138 778	1.535 237
C	1.803 844	−4.629 813	2.839 663
C	0.631 890	−4.792 566	3.574 002
C	−0.594 103	−4.473 855	2.989 890
C	−0.643 235	−3.985 073	1.687 341
H	−1.607 726	−3.738 671	1.234 820
H	−1.517 609	−4.590 759	3.560 109
H	0.670 908	−5.164 174	4.600 635
H	2.771 264	−4.873 517	3.285 598
H	2.678 507	−3.997 565	0.975 002
H	−0.297 890	−2.389 462	−0.394 969
H	1.858 578	−1.324 119	2.631 110
H	−0.290 539	0.245 323	3.446 799
C	−1.730 330	1.230 843	1.311 764
C	−2.930 566	0.573 332	1.631 271
C	−4.104 198	1.316 888	1.714 856
C	−4.093 884	2.694 483	1.500 143
C	−2.895 662	3.333 326	1.165 444
C	−1.708 670	2.611 381	1.047 758
C	−0.437 246	3.279 042	0.552 534
C	0.690 493	3.327 840	1.559 108
C	2.003 973	3.431 338	1.084 889
C	3.065 266	3.628 507	1.963 316
C	2.827 451	3.711 594	3.336 850
C	1.527 262	3.578 758	3.821 039
C	0.464 656	3.390 894	2.936 056
H	−0.557 467	3.308 972	3.315 953
H	1.335 223	3.633 453	4.895 607
H	3.658 322	3.870 412	4.028 583
H	4.082 445	3.705 638	1.571 085
H	2.189 814	3.374 672	0.008 704
C	−0.704 975	4.657 591	−0.031 988
C	−0.379 928	5.836 329	0.645 267
C	−0.680 892	7.082 054	0.092 673
C	−1.308 416	7.169 849	−1.147 498
C	−1.635 688	5.999 759	−1.833 585
C	−1.336 942	4.759 513	−1.277 831
H	−1.607 821	3.845 693	−1.809 030
H	−2.123 537	6.051 722	−2.810 049
H	−1.537 801	8.145 932	−1.581 625

H	−0.415 708	7.991 454	0.637 625
H	0.120 588	5.781 281	1.614 138
H	−0.100 473	2.635 740	−0.283 077
H	−2.874 477	4.401 325	0.957 452
O	−5.274 949	3.330 153	1.611 338
C	−5.327 451	4.705 227	1.341 831
H	−6.374 364	5.008 883	1.466 136
H	−4.701 123	5.285 627	2.041 503
H	−5.006 662	4.932 843	0.310 290
H	−5.055 922	0.838 572	1.946 396
C	−2.968 615	−0.924 762	1.876 544
C	−3.890 186	−1.616 724	0.887 209
C	−5.264 867	−1.747 924	1.115 114
C	−6.083 828	−2.377 113	0.180 110
C	−5.539 756	−2.904 894	−0.990 306
C	−4.172 249	−2.788 348	−1.224 954
C	−3.360 707	−2.136 701	−0.298 367
H	−2.290 192	−2.034 885	−0.501 729
H	−3.726 534	−3.211 560	−2.127 550
H	−6.180 429	−3.410 926	−1.716 055
H	−7.155 244	−2.467 619	0.374 997
H	−5.696 594	−1.376 652	2.047 773
C	−3.241 880	−1.285 756	3.329 588
C	−3.092 697	−0.355 119	4.362 754
C	−3.263 699	−0.729 633	5.695 711
C	−3.592 562	−2.042 937	6.019 799
C	−3.750 194	−2.979 154	4.997 958
C	−3.575 468	−2.603 581	3.669 123
H	−3.708 990	−3.342 851	2.875 912
H	−4.014 238	−4.012 400	5.237 340
H	−3.731 477	−2.336 192	7.062 950
H	−3.143 480	0.016 381	6.485 120
H	−2.848 912	0.682 146	4.122 627
H	−1.959 391	−1.311 380	1.666 506
C	1.803 438	0.063 018	−3.217 748
C	2.864 344	0.502 318	−4.008 478
C	3.097 347	1.866 398	−4.193 812
C	2.259 572	2.782 137	−3.560 233
C	1.199 408	2.343 235	−2.760 459
C	0.937 127	0.972 357	−2.581 685
H	0.557 915	3.094 983	−2.295 658
H	2.423 718	3.856 540	−3.686 161
H	3.923 264	2.208 852	−4.822 012
H	3.510 327	−0.236 564	−4.492 386
H	1.638 576	−1.011 174	−3.108 290
C	−4.964 193	−0.540 523	−4.118 040
C	−5.362 873	0.412 320	−3.267 543
C	−1.630 682	0.332 396	−3.638 055
C	−4.605 482	1.660 345	−2.913 002
C	−2.061 793	1.382 471	−2.811 645
C	−3.355 219	1.402 224	−2.035 515
H	−4.289 380	2.190 782	−3.828 662
H	−0.886 827	0.604 233	−4.396 801
H	−1.648 792	2.372 706	−3.042 050
H	−5.281 713	2.344 312	−2.378 111
H	−3.280 953	2.180 144	−1.261 433
H	−3.501 396	0.452 157	−1.490 959
C	−3.674 039	−0.565 885	−4.887 209
H	−3.503 550	0.402 731	−5.388 363
H	−3.754 963	−1.315 958	−5.689 005
C	−2.442 120	−0.887 037	−4.000 777
H	−2.789 107	−1.419 378	−3.103 191
H	−1.781 303	−1.585 693	−4.536 183
H	−5.613 515	−1.417 063	−4.227 319
H	−6.307 400	0.249 586	−2.736 136

Intermediate XIII

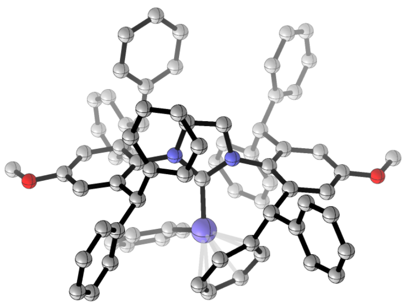

Rh	0.516 317	−1.131 345	−1.689 160
C	−1.161 911	−0.361 980	−2.510 941
C	−2.403 904	−1.023 624	−2.540 971
C	−3.438 470	−0.624 113	−3.390 132
C	−3.268 177	0.460 065	−4.250 975
C	−2.059 163	1.155 154	−4.221 862
C	−1.036 441	0.759 360	−3.355 842
H	−0.106 426	1.332 364	−3.348 319
H	−1.907 524	2.022 279	−4.871 932
H	−4.071 523	0.765 175	−4.926 895
H	−4.384 441	−1.174 466	−3.381 348
H	−2.575 368	−1.883 663	−1.890 591
C	0.271 517	−0.317 884	0.077 857
N	1.306 211	0.226 020	0.803 402
C	0.954 261	0.429 920	2.133 293
C	−0.333 519	0.050 525	2.254 316
N	−0.743 727	−0.380 452	1.000 463
C	−2.079 468	−0.815 155	0.753 289
C	−2.371 551	−2.190 664	0.719 589
C	−3.686 941	−2.590 725	0.525 915
C	−4.702 716	−1.643 437	0.345 841
C	−4.401 617	−0.282 173	0.395 655
C	−3.088 702	0.142 537	0.611 373
C	−2.767 239	1.622 664	0.729 528
C	−3.736 998	2.490 220	−0.053 553
C	−4.704 984	3.289 250	0.561 222
C	−5.593 542	4.042 842	−0.207 864
C	−5.522 913	4.006 260	−1.598 595
C	−4.557 348	3.210 356	−2.217 186
C	−3.671 666	2.459 710	−1.450 724
H	−2.920 901	1.836 687	−1.941 400
H	−4.487 726	3.164 504	−3.306 667
H	−6.218 034	4.597 547	−2.199 948
H	−6.343 530	4.664 758	0.287 357
H	−4.762 922	3.328 786	1.651 206
C	−2.643 167	2.061 334	2.178 269
C	−1.818 572	3.143 888	2.503 492
C	−1.678 645	3.570 019	3.820 903
C	−2.370 132	2.919 341	4.843 282
C	−3.206 207	1.849 226	4.530 918
C	−3.343 432	1.426 124	3.208 330
H	−3.989 956	0.577 421	2.972 594

H	−3.756 200	1.334 665	5.322 875
H	−2.258 830	3.247 358	5.879 531
H	−1.019 376	4.411 855	4.046 230
H	−1.266 413	3.658 889	1.713 866
H	−1.778 884	1.761 638	0.260 925
H	−5.172 056	0.473 156	0.247 042
O	−5.938 507	−2.130 950	0.125 587
C	−6.974 386	−1.227 273	−0.146 800
H	−7.872 459	−1.827 581	−0.339 483
H	−6.753 855	−0.610 602	−1.035 543
H	−7.170 394	−0.555 794	0.707 767
H	−3.948 319	−3.648 295	0.477 231
C	−1.236 489	−3.182 579	0.885 478
C	−1.583 113	−4.581 665	0.417 937
C	−1.640 355	−5.677 774	1.281 065
C	−1.957 193	−6.948 909	0.797 759
C	−2.220 977	−7.141 347	−0.555 231
C	−2.169 004	−6.051 480	−1.426 571
C	−1.852 577	−4.786 136	−0.943 418
H	−1.819 823	−3.930 750	−1.623 479
H	−2.378 300	−6.188 848	−2.490 462
H	−2.468 164	−8.136 498	−0.932 574
H	−1.996 205	−7.794 456	1.489 005
H	−1.430 319	−5.537 759	2.343 534
C	−0.651 456	−3.133 529	2.281 413
C	−1.445 680	−2.881 425	3.404 339
C	−0.880 114	−2.814 416	4.677 168
C	0.490 858	−3.000 333	4.847 517
C	1.289 873	−3.269 057	3.736 478
C	0.719 333	−3.338 088	2.468 230
H	1.349 506	−3.552 956	1.602 485
H	2.365 091	−3.425 674	3.855 375
H	0.935 181	−2.940 069	5.843 852
H	−1.516 022	−2.610 335	5.542 324
H	−2.518 674	−2.718 923	3.273 623
H	−0.452 153	−2.818 296	0.194 016
H	−0.993 264	0.028 687	3.113 961
H	1.662 857	0.812 997	2.860 360
C	2.548 348	0.727 821	0.317 812
C	3.627 629	−0.144 329	0.084 410
C	4.879 103	0.385 868	−0.193 322
C	5.069 824	1.773 457	−0.246 303
C	3.979 593	2.628 051	−0.080 307
C	2.705 360	2.111 327	0.180 637
C	1.505 236	3.039 544	0.213 735
C	1.780 566	4.284 901	1.040 864
C	1.701 152	4.208 087	2.436 410
C	1.983 917	5.316 145	3.230 483
C	2.354 215	6.524 394	2.639 313
C	2.431 227	6.613 147	1.251 204
C	2.142 124	5.502 874	0.457 542
H	2.181 370	5.584 975	−0.631 431
H	2.710 007	7.557 912	0.777 958
H	2.574 360	7.396 124	3.260 015
H	1.911 834	5.236 914	4.318 250
H	1.401 643	3.265 904	2.902 509
C	0.991 866	3.376 017	−1.180 767
C	−0.293 542	3.919 558	−1.302 010
C	−0.795 440	4.314 707	−2.537 220
C	−0.013 431	4.178 511	−3.683 887
C	1.258 273	3.621 953	−3.580 981
C	1.754 654	3.217 295	−2.340 429
H	2.747 774	2.769 983	−2.279 046
H	1.872 533	3.490 230	−4.475 167
H	−0.402 298	4.490 002	−4.656 120
H	−1.807 593	4.719 749	−2.600 891
H	−0.914 678	4.045 912	−0.411 948
H	0.691 586	2.503 904	0.723 414
H	4.095 916	3.709 243	−0.150 809
O	6.326 589	2.192 109	−0.484 879
C	6.572 847	3.569 411	−0.560 492
H	6.309 171	4.085 366	0.379 215
H	7.648 330	3.686 840	−0.742 920
H	6.016 064	4.040 466	−1.389 766
H	5.734 729	−0.267 359	−0.371 500
C	3.348 979	−1.628 923	−0.009 667
C	4.571 342	−2.492 532	0.210 139
C	5.279 085	−3.062 451	−0.850 803
C	6.419 872	−3.829 251	−0.611 961
C	6.864 821	−4.038 255	0.691 285
C	6.162 268	−3.476 005	1.757 772
C	5.025 653	−2.709 993	1.516 364
H	4.477 734	−2.264 979	2.352 388
H	6.501 586	−3.638 357	2.783 748
H	7.755 995	−4.642 054	0.877 976
H	6.961 798	−4.269 138	−1.452 677
H	4.928 443	−2.908 348	−1.874 218
C	2.615 845	−1.912 471	−1.312 275
C	1.771 592	−3.054 794	−1.386 277
C	0.985 543	−3.300 924	−2.526 410
C	0.999 014	−2.348 589	−3.570 395
C	1.905 514	−1.258 739	−3.556 879
C	2.725 096	−1.046 311	−2.436 537
H	3.414 970	−0.202 216	−2.412 064
H	1.934 313	−0.565 682	−4.398 972
H	0.317 076	−2.464 014	−4.414 408
H	0.353 701	−4.187 386	−2.581 421
H	1.746 275	−3.757 590	−0.550 377
H	2.635 165	−1.865 124	0.796 190

Intermediate XIV

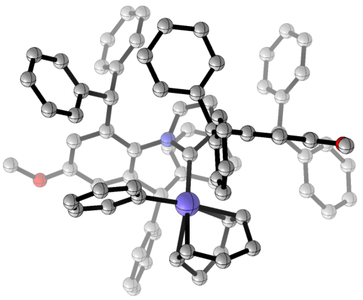

Rh	0.118 650	0.055 947	−2.194 776
C	−1.813 900	−0.642 828	−2.352 788
C	−2.916 460	0.217 584	−2.522 594
C	−4.169 718	−0.235 695	−2.940 259
C	−4.376 645	−1.585 874	−3.219 212
C	−3.315 824	−2.472 458	−3.040 072
C	−2.071 826	−2.007 768	−2.603 260
H	−1.270 290	−2.744 604	−2.507 975
H	−3.448 863	−3.539 342	−3.243 974
H	−5.350 887	−1.941 428	−3.564 413
H	−4.987 659	0.481 779	−3.058 053
H	−2.804 539	1.287 895	−2.335 608
C	0.092 050	0.025 423	−0.097 785
N	1.019 317	−0.508 837	0.761 071
C	0.728 790	−0.225 749	2.085 550

C	−0.373 472	0.546 522	2.077 710
N	−0.772 698	0.660 483	0.755 701
C	−2.032 116	1.268 145	0.452 438
C	−3.205 952	0.629 197	0.877 285
C	−4.440 784	1.212 572	0.583 071
C	−4.505 229	2.441 092	−0.071 282
C	−3.322 379	3.110 408	−0.404 804
C	−2.082 556	2.533 728	−0.161 173
C	−0.793 960	3.241 176	−0.535 398
C	−1.021 611	4.392 761	−1.499 107
C	−0.844 356	5.729 739	−1.136 225
C	−1.081 347	6.754 129	−2.054 819
C	−1.500 159	6.456 852	−3.348 518
C	−1.685 662	5.124 026	−3.719 923
C	−1.448 340	4.106 157	−2.802 798
H	−1.601 846	3.064 008	−3.090 502
H	−2.017 290	4.875 341	−4.731 121
H	−1.683 356	7.259 366	−4.066 925
H	−0.935 205	7.793 820	−1.751 775
H	−0.513 300	5.975 408	−0.125 264
C	0.019 291	3.660 428	0.674 810
C	−0.573 854	3.924 580	1.912 480
C	0.190 424	4.380 806	2.986 976
C	1.561 227	4.582 781	2.838 937
C	2.166 918	4.315 344	1.610 007
C	1.399 698	3.859 930	0.541 347
H	1.879 348	3.677 491	−0.422 651
H	3.243 607	4.453 045	1.482 142
H	2.159 583	4.942 248	3.679 620
H	−0.292 965	4.583 026	3.946 051
H	−1.649 366	3.773 566	2.034 480
H	−0.201 641	2.484 733	−1.084 972
H	−3.399 399	4.082 648	−0.891 293
O	−5.654 189	3.056 150	−0.407 746
C	−6.863 780	2.392 444	−0.160 772
H	−7.661 040	3.035 527	−0.554 092
H	−7.033 340	2.233 964	0.918 881
H	−6.902 759	1.414 368	−0.671 213
H	−5.344 189	0.676 786	0.872 287
C	−3.169 674	−0.627 146	1.730 277
C	−4.335 909	−1.564 381	1.462 047
C	−4.491 754	−2.074 835	0.168 244
C	−5.507 841	−2.978 905	−0.120 979
C	−6.392 761	−3.386 049	0.879 448
C	−6.253 212	−2.873 229	2.166 333
C	−5.230 510	−1.967 470	2.456 504
H	−5.123 897	−1.580 087	3.471 473
H	−6.942 259	−3.179 950	2.957 319
H	−7.189 945	−4.098 671	0.653 843
H	−5.602 908	−3.364 439	−1.138 475
H	−3.806 491	−1.758 378	−0.620 580
C	−3.034 966	−0.288 568	3.207 599
C	−2.332 116	−1.163 805	4.044 401
C	−2.181 836	−0.889 905	5.400 212
C	−2.734 377	0.268 731	5.948 153
C	−3.440 885	1.143 368	5.126 023
C	−3.590 548	0.866 006	3.765 990
H	−4.134 100	1.566 198	3.128 001
H	−3.877 973	2.054 440	5.542 398
H	−2.611 358	0.489 400	7.011 196
H	−1.619 671	−1.582 575	6.031 587
H	−1.883 309	−2.062 710	3.617 051
H	−2.265 095	−1.192 387	1.452 924
H	−0.920 688	1.016 313	2.885 285
H	1.335 004	−0.599 965	2.901 503
C	2.204 116	−1.226 007	0.421 990
C	2.101 877	−2.498 072	−0.157 495
C	3.270 140	−3.180 758	−0.499 516
C	4.523 290	−2.608 906	−0.269 046
C	4.608 797	−1.352 475	0.336 279
C	3.463 045	−0.652 532	0.701 512
C	3.597 236	0.671 906	1.435 374
C	3.627 649	0.463 489	2.943 483
C	2.994 847	1.395 196	3.774 902
C	3.010 269	1.246 317	5.158 649
C	3.661 921	0.157 831	5.739 004
C	4.296 207	−0.774 640	4.921 116
C	4.278 556	−0.623 209	3.534 052
H	4.769 273	−1.368 155	2.904 478
H	4.807 624	−1.633 143	5.363 391
H	3.670 723	0.034 770	6.824 621
H	2.500 658	1.981 603	5.786 341
H	2.468 331	2.238 782	3.324 335
C	4.761 974	1.527 446	0.946 782
C	4.915 693	1.769 159	−0.425 752
C	5.923 220	2.605 096	−0.896 570
C	6.805 641	3.215 933	−0.004 738
C	6.669 883	2.974 392	1.359 038
C	5.657 084	2.137 071	1.830 528
H	5.563 251	1.959 801	2.903 182
H	7.357 914	3.438 817	2.069 786
H	7.597 410	3.872 305	−0.373 541
H	6.019 556	2.779 069	−1.971 182
H	4.238 909	1.291 626	−1.136 127
H	2.688 495	1.261 170	1.226 492
H	5.597 140	−0.920 331	0.500 677
O	5.688 031	−3.199 349	−0.592 085
C	5.654 685	−4.447 306	−1.230 907
H	5.187 404	−5.220 781	−0.596 808
H	5.109 482	−4.401 767	−2.189 683
H	6.697 453	−4.727 940	−1.424 588
H	3.179 631	−4.161 299	−0.963 204
C	0.739 385	−3.115 553	−0.416 859
C	0.012 343	−3.534 957	0.844 958
C	0.677 779	−3.823 906	2.039 091
C	−0.025 808	−4.309 143	3.142 972
C	−1.402 201	−4.513 165	3.064 373
C	−2.078 882	−4.208 124	1.881 693
C	−1.375 468	−3.718 942	0.784 978
H	−1.907 001	−3.483 038	−0.140 766
H	−3.161 834	−4.336 627	1.813 666
H	−1.952 137	−4.896 716	3.927 093
H	0.509 583	−4.534 303	4.068 818
H	1.758 838	−3.675 838	2.103 694
C	0.819 371	−4.244 072	−1.427 067
C	0.774 701	−5.592 640	−1.066 624
C	0.906 432	−6.588 039	−2.036 581
C	1.080 176	−6.247 743	−3.376 299
C	1.120 917	−4.901 849	−3.744 809
C	0.992 671	−3.912 980	−2.775 326
H	1.022 867	−2.855 222	−3.048 730
H	1.245 756	−4.621 699	−4.794 032

H	1.176 326	−7.029 022	−4.134 098
H	0.866 934	−7.639 212	−1.740 251
H	0.630 241	−5.866 677	−0.019 130
H	0.132 427	−2.322 713	−0.889 919
C	−0.095 535	0.997 692	−4.129 061
C	0.209 466	−0.346 654	−4.337 812
C	1.561 895	−0.838 773	−4.779 962
C	2.711 959	−0.276 944	−3.933 839
C	2.297 423	0.087 469	−2.523 883
C	1.874 285	1.359 896	−2.150 965
C	1.698 451	2.503 292	−3.115 890
C	0.895 949	2.119 869	−4.368 164
H	0.356 783	3.007 497	−4.730 757
H	1.575 991	1.829 337	−5.184 569
H	1.179 425	3.324 285	−2.600 224
H	2.684 857	2.914 305	−3.404 700
H	2.008 690	1.648 506	−1.103 807
H	2.703 092	−0.559 533	−1.747 250
H	3.523 753	−1.018 252	−3.880 188
H	3.148 777	0.608 024	−4.423 220
H	1.567 633	−1.937 241	−4.729 994
H	1.717 985	−0.596 562	−5.848 540
H	−0.625 458	−1.024 874	−4.539 998
H	−1.156 140	1.267 051	−4.168 330

Intermediate XV

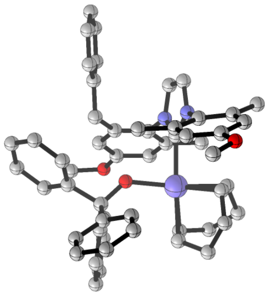

Rh	−0.764 110	0.730 931	−1.196 349
C	−1.712 149	0.540 464	−3.089 972
C	−2.652 700	0.740 868	−2.051 595
C	−3.395 912	2.020 542	−1.764 183
C	−2.493 504	3.257 197	−1.775 924
C	−1.095 009	2.929 864	−1.309 154
C	−0.054 713	2.553 128	−2.144 439
C	−0.119 227	2.408 187	−3.641 241
C	−1.334 421	1.604 870	−4.104 724
H	−1.116 795	1.130 898	−5.074 683
H	−2.195 090	2.267 153	−4.289 213
H	0.802 277	1.897 026	−3.961 184
H	−0.090 991	3.407 382	−4.116 005
H	0.954 724	2.656 030	−1.741 831
H	−0.820 014	3.295 320	−0.318 686
H	−2.920 075	4.029 425	−1.118 335
H	−2.456 771	3.702 839	−2.782 153
H	−3.852 739	1.913 697	−0.767 310
H	−4.240 501	2.135 996	−2.470 455
H	−3.199 032	−0.138 648	−1.706 689
H	−1.623 170	−0.489 040	−3.455 045
C	−0.630 464	−1.310 859	−1.062 205
N	0.515 642	−2.009 912	−1.301 138
C	0.303 994	−3.377 234	−1.225 323
C	−1.000 283	−3.549 516	−0.913 708
N	−1.554 630	−2.281 700	−0.818 064
C	−2.889 400	−2.054 097	−0.367 096
C	−3.077 353	−1.569 614	0.931 616
C	−4.377 762	−1.267 530	1.349 377
C	−5.464 163	−1.482 922	0.497 929
C	−5.253 453	−2.025 202	−0.776 296
C	−3.970 488	−2.314 420	−1.227 255
C	−3.744 967	−2.838 662	−2.614 482
H	−3.047 947	−2.192 086	−3.169 521
H	−6.121 522	−2.192 649	−1.416 386
O	−6.742 389	−1.208 242	0.820 393
C	−7.014 473	−0.631 297	2.067 225
H	−6.722 041	−1.294 437	2.900 246
H	−6.500 899	0.338 603	2.190 594
H	−8.098 683	−0.467 646	2.108 311
H	−4.519 126	−0.871 122	2.354 881
C	−1.914 799	−1.399 269	1.857 171
H	−1.174 923	−0.684 774	1.456 974
H	−1.579 723	−4.446 973	−0.720 213
H	1.108 618	−4.092 004	−1.364 368
C	1.814 347	−1.441 655	−1.501 620
C	2.684 470	−1.352 608	−0.409 251
C	3.953 447	−0.798 286	−0.601 225
C	4.335 991	−0.322 683	−1.857 168
C	3.463 354	−0.455 218	−2.942 516
C	2.203 419	−1.024 338	−2.785 467
C	1.284 332	−1.184 030	−3.956 332
H	0.912 305	−2.217 373	−4.034 487
H	3.794 632	−0.098 118	−3.919 389
O	5.512 813	0.285 948	−2.107 700
C	6.405 256	0.482 947	−1.044 426
H	6.734 268	−0.473 950	−0.602 913
H	7.278 661	1.001 080	−1.459 937
H	5.960 525	1.108 592	−0.250 804
H	4.609 290	−0.703 612	0.264 043
C	2.269 260	−1.811 688	0.957 289
C	2.303 524	−3.303 867	1.175 503
C	3.196 903	−4.133 602	0.489 290
C	3.205 186	−5.511 162	0.707 707
C	2.322 325	−6.082 541	1.622 628
C	1.437 780	−5.262 642	2.323 742
C	1.431 651	−3.888 161	2.101 443
H	0.736 321	−3.247 109	2.649 467
H	0.743 483	−5.698 774	3.046 586
H	2.325 305	−7.162 048	1.791 472
H	3.907 622	−6.142 856	0.157 734
H	3.885 761	−3.693 197	−0.236 821
H	1.264 934	−1.414 170	1.158 636
O	0.321 640	0.717 397	0.568 926
C	0.684 489	1.722 455	1.446 000
C	1.540 290	1.048 464	2.553 851
C	0.909 859	0.114 944	3.388 209
C	1.623 056	−0.609 273	4.335 903
C	2.996 742	−0.405 714	4.486 923
C	3.629 621	0.545 759	3.693 789
C	2.906 606	1.269 578	2.739 942
H	3.425 091	2.013 437	2.134 191
H	4.699 392	0.735 972	3.815 196
H	3.562 528	−0.975 495	5.227 865
H	1.105 416	−1.337 369	4.965 990
H	−0.163 668	−0.041 278	3.282 947
C	−0.531 096	2.342 085	2.166 884

C	−1.832 434	2.055 397	1.753 388
C	−2.935 659	2.584 290	2.423 788
C	−2.755 158	3.408 340	3.532 026
C	−1.459 513	3.688 104	3.968 851
C	−0.362 754	3.153 431	3.297 575
H	0.645 998	3.353 090	3.668 682
H	−1.301 846	4.319 704	4.846 883
H	−3.616 681	3.823 852	4.060 773
H	−3.943 533	2.342 901	2.073 806
H	−1.972 521	1.403 330	0.886 397
C	1.539 431	2.790 360	0.736 488
C	1.406 509	4.171 227	0.889 881
C	2.238 571	5.052 002	0.193 053
C	3.229 114	4.568 400	−0.657 128
C	3.373 645	3.189 002	−0.819 303
C	2.528 871	2.319 149	−0.137 788
H	2.609 117	1.243 898	−0.288 313
H	4.130 936	2.783 925	−1.496 332
H	3.878 387	5.261 111	−1.198 255
H	2.104 027	6.129 427	0.318 332
H	0.633 659	4.576 961	1.544 723
H	−1.391 783	−2.359 466	1.996 121
H	−2.248 962	−1.041 579	2.839 871
H	−3.304 674	−3.847 585	−2.605 313
H	−4.688 601	−2.885 288	−3.173 216
H	1.791 056	−0.924 801	−4.894 921
H	0.405 732	−0.535 545	−3.839 775
H	2.922 456	−1.331 867	1.703 109

Intermediate XVI

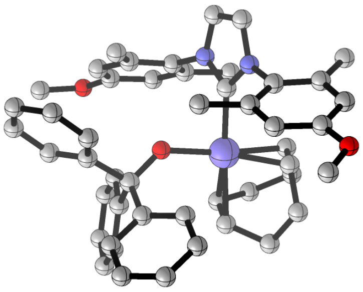

Rh	0.471 261	−0.715 241	0.924 643
C	2.301 423	−1.225 123	1.752 916
C	1.417 717	−2.310 095	1.966 466
C	0.808 708	−2.647 339	3.315 979
C	−0.546 116	−1.964 137	3.504 095
C	−0.631 775	−0.636 377	2.800 454
C	0.306 359	0.382 103	2.855 498
C	1.594 989	0.316 230	3.640 264
C	2.747 117	−0.225 736	2.790 008
H	3.224 115	0.608 559	2.251 014
H	3.539 830	−0.664 718	3.425 949
H	1.847 678	1.324 418	4.001 459
H	1.451 833	−0.301 421	4.540 575
H	−0.034 146	1.378 561	2.570 065
H	−1.636 492	−0.357 613	2.477 468
H	−1.341 406	−2.605 562	3.093 818
H	−0.786 496	−1.834 449	4.576 428
H	0.688 343	−3.738 343	3.404 975
H	1.505 638	−2.362 933	4.120 335
H	1.551 145	−3.170 019	1.299 743
H	3.032 456	−1.336 819	0.950 402
C	0.804 999	−1.677 096	−0.854 331
N	−0.153 907	−2.373 686	−1.527 018
C	0.360 706	−2.985 810	−2.656 933
C	1.672 942	−2.661 177	−2.711 553
N	1.927 118	−1.865 368	−1.605 526
C	3.174 053	−1.200 071	−1.404 220
C	3.241 384	0.172 446	−1.666 792
C	4.438 365	0.843 623	−1.394 692
C	5.547 785	0.147 820	−0.908 095
C	5.470 042	−1.236 781	−0.709 972
C	4.287 944	−1.927 719	−0.950 624
C	4.190 759	−3.400 333	−0.681 536
H	3.363 143	−3.619 224	0.010 719
H	3.997 387	−3.973 852	−1.601 236
H	5.119 805	−3.778 718	−0.235 996
H	6.355 327	−1.756 153	−0.338 758
O	6.728 998	0.722 356	−0.612 069
C	6.859 187	2.109 586	−0.754 704
H	7.877 749	2.364 317	−0.435 946
H	6.138 307	2.655 876	−0.121 040
H	6.722 623	2.430 172	−1.802 427
H	4.483 120	1.916 706	−1.580 803
C	2.066 578	0.899 938	−2.239 790
H	1.191 333	0.856 664	−1.568 071
H	2.314 325	1.953 725	−2.422 465
H	1.756 815	0.447 338	−3.195 871
H	2.446 524	−2.903 337	−3.434 104
H	−0.261 475	−3.585 573	−3.314 645
C	−1.559 013	−2.350 161	−1.253 261
C	−2.355 622	−1.484 048	−2.011 860
C	−3.729 696	−1.446 221	−1.756 803
C	−4.285 825	−2.246 059	−0.755 961
C	−3.473 668	−3.132 650	−0.040 475
C	−2.105 917	−3.206 147	−0.285 005
C	−1.239 027	−4.168 407	0.465 900
H	−0.663 533	−4.807 799	−0.221 803
H	−1.838 944	−4.814 760	1.119 624
H	−0.511 031	−3.621 645	1.080 483
H	−3.938 181	−3.762 497	0.720 435
O	−5.587 083	−2.213 424	−0.400 363
C	−6.427 199	−1.277 880	−1.019 737
H	−6.075 595	−0.244 437	−0.853 682
H	−6.510 974	−1.456 743	−2.105 964
H	−7.418 315	−1.395 799	−0.563 784
H	−4.339 081	−0.741 357	−2.321 770
C	−1.743 151	−0.579 006	−3.033 199
H	−2.484 417	0.120 766	−3.439 213
H	−1.300 379	−1.142 083	−3.869 800
H	−0.951 354	0.012 210	−2.551 905
O	−0.563 414	0.643 443	−0.244 880
C	−1.161 663	1.855 117	0.046 192
C	−2.249 460	1.683 240	1.123 766
C	−2.460 293	2.532 219	2.211 197
C	−3.481 503	2.276 267	3.130 715
C	−4.321 631	1.178 057	2.969 404
C	−4.121 486	0.319 925	1.885 980
C	−3.088 014	0.567 858	0.988 747
H	−2.896 120	−0.117 233	0.164 936
H	−4.757 662	−0.558 024	1.744 136
H	−5.121 673	0.985 283	3.688 478
H	−3.616 123	2.946 995	3.983 173
H	−1.812 820	3.397 035	2.364 220
C	−1.828 053	2.343 167	−1.271 116

C	−0.989763	2.709134	−2.333511
C	−1.504155	3.074605	−3.571819
C	−2.885027	3.096525	−3.779490
C	−3.731522	2.764711	−2.726575
C	−3.207565	2.396480	−1.483755
H	−3.893074	2.152843	−0.671625
H	−4.815821	2.798965	−2.863553
H	−3.294653	3.385710	−4.750482
H	−0.823510	3.350097	−4.381648
H	0.088968	2.708917	−2.175965
C	−0.134719	2.941159	0.429322
C	1.201902	2.613424	0.659572
C	2.142829	3.595759	0.969509
C	1.762128	4.933247	1.046609
C	0.432757	5.278331	0.798489
C	−0.500477	4.293248	0.485373
H	−1.531067	4.579511	0.259375
H	0.122134	6.325545	0.838312
H	2.496155	5.706087	1.288207
H	3.182678	3.306815	1.147767
H	1.499683	1.563136	0.593909

Intermediate XVII

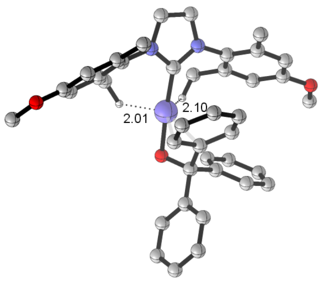

Rh	0.458522	0.111423	−0.747983
C	1.047125	−1.713491	−0.307361
N	2.334305	−2.173567	−0.310887
C	2.377195	−3.558907	−0.282951
C	1.092745	−3.984599	−0.233515
N	0.293127	−2.851707	−0.233252
C	−1.127239	−2.857244	−0.119408
C	−1.717648	−3.424684	1.026085
C	−3.099569	−3.362404	1.156645
C	−3.892010	−2.728747	0.192483
C	−3.289194	−2.171274	−0.936822
C	−1.903347	−2.241417	−1.109272
C	−1.283462	−1.627727	−2.327168
H	−0.975258	−0.573025	−2.120001
H	−2.009715	−1.558215	−3.148490
H	−0.405501	−2.187259	−2.675902
H	−3.881187	−1.655223	−1.692404
O	−5.214292	−2.707410	0.435520
C	−6.056497	−2.058901	−0.480177
H	−5.788565	−0.995584	−0.595402
H	−6.030143	−2.544305	−1.471967
H	−7.074064	−2.134146	−0.076361
H	−3.595504	−3.782879	2.033625
C	−0.894927	−4.059471	2.110142
H	0.030517	−3.495106	2.293262
H	−1.465054	−4.102208	3.048019
H	−0.606458	−5.092408	1.857285
H	0.675655	−4.986394	−0.217129
H	3.310568	−4.111898	−0.319814
C	3.471176	−1.317435	−0.307666
C	4.355477	−1.354800	0.786557
C	5.445061	−0.490726	0.786201
C	5.652356	0.413754	−0.261306
C	4.748818	0.455228	−1.325364
C	3.648358	−0.406538	−1.356036
C	2.655085	−0.297505	−2.473221
H	2.225207	−1.266953	−2.754964
H	1.823866	0.410192	−2.197045
H	3.105631	0.158845	−3.365602
H	4.877226	1.164534	−2.143033
O	6.734369	1.207004	−0.154804
C	6.969458	2.167944	−1.146689
H	7.880196	2.705261	−0.853911
H	6.137527	2.889774	−1.223007
H	7.131370	1.705485	−2.136337
H	6.151226	−0.484306	1.618661
C	4.118158	−2.267196	1.954945
H	3.051698	−2.293237	2.223261
H	4.427165	−3.302988	1.742822
H	4.688730	−1.927389	2.829643
O	−0.300674	1.928378	−1.231549
C	−1.130376	2.061284	−0.128323
C	−0.583969	0.979754	0.837607
C	−1.351158	0.054564	1.607712
C	−0.761613	−0.666517	2.620119
C	0.619097	−0.523891	2.911710
C	1.399593	0.335706	2.175982
C	0.829085	1.100728	1.114662
H	1.358836	1.997185	0.778579
H	2.457992	0.470283	2.413773
H	1.056551	−1.092742	3.736966
H	−1.368755	−1.359104	3.206953
H	−2.412961	−0.072054	1.388455
C	−2.602016	1.833163	−0.505093
C	−3.648573	1.955841	0.417034
C	−4.971940	1.777564	0.020552
C	−5.274393	1.489558	−1.311698
C	−4.239408	1.375788	−2.238481
C	−2.915419	1.546681	−1.834948
H	−2.084999	1.494748	−2.541232
H	−4.465466	1.169690	−3.288559
H	−6.314697	1.374619	−1.627680
H	−5.775461	1.882191	0.754299
H	−3.430410	2.217356	1.454949
C	−1.014530	3.458758	0.485023
C	−1.131470	3.687765	1.858187
C	−1.047711	4.982635	2.370310
C	−0.840026	6.061856	1.513559
C	−0.709113	5.836357	0.142407
C	−0.792888	4.543281	−0.367148
H	−0.675084	4.344306	−1.434246
H	−0.536176	6.677268	−0.534631
H	−0.771200	7.076474	1.913971
H	−1.136249	5.147000	3.447414
H	−1.269510	2.842909	2.539008

Intermediate XVIII

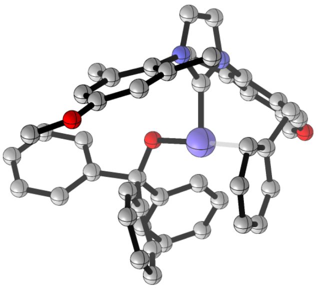

Rh	0.067 635	−0.757 408	0.624 174
C	0.139 270	−1.887 420	−0.884 158
N	−0.936 325	−2.381 967	−1.549 493
C	−0.537 817	−3.154 490	−2.629 722
C	0.818 688	−3.130 383	−2.644 934
N	1.219 489	−2.354 410	−1.568 382
C	2.554 202	−1.952 398	−1.257 557
C	3.004 911	−0.727 383	−1.772 180
C	4.252 909	−0.253 524	−1.359 428
C	5.027 167	−0.986 061	−0.454 734
C	4.582 907	−2.234 794	−0.009 586
C	3.347 317	−2.738 101	−0.406 793
C	2.913 653	−4.098 641	0.055 251
H	3.079 317	−4.221 694	1.135 350
H	1.854 320	−4.290 556	−0.156 550
H	3.500 566	−4.880 587	−0.453 379
H	5.224 802	−2.799 727	0.669 355
O	6.211 043	−0.567 089	0.034 627
C	6.680 953	0.701 129	−0.332 116
H	7.628 707	0.850 743	0.200 344
H	6.867 546	0.773 044	−1.417 932
H	5.975 156	1.498 443	−0.039 654
H	4.592 218	0.709 783	−1.740 331
C	2.142 336	0.085 272	−2.685 055
H	2.699 151	0.931 608	−3.108 264
H	1.745 032	−0.521 543	−3.513 364
H	1.277 326	0.484 260	−2.118 310
H	1.536 134	−3.595 298	−3.314 515
H	−1.251 657	−3.658 769	−3.274 045
C	−2.273 830	−2.070 676	−1.173 815
C	−3.045 568	−1.268 708	−2.020 935
C	−4.352 392	−0.955 596	−1.635 018
C	−4.858 006	−1.404 055	−0.413 519
C	−4.047 865	−2.162 431	0.439 990
C	−2.744 376	−2.487 703	0.083 327
C	−1.816 342	−3.142 952	1.069 543
H	−2.404 640	−3.728 054	1.794 574
H	−1.147 734	−3.849 444	0.555 677
C	−0.965 255	−2.124 774	1.822 475
C	0.447 757	−2.332 917	2.026 506
C	1.119 166	−1.700 378	3.124 647
C	0.467 425	−0.781 531	3.900 808
C	−0.915 024	−0.506 585	3.659 333
C	−1.622 064	−1.191 816	2.705 138
H	−2.690 201	−1.003 482	2.574 786
H	−1.424 059	0.245 028	4.267 186
H	0.989 937	−0.256 563	4.701 137
H	2.170 080	−1.938 095	3.306 892
H	0.925 321	−3.202 768	1.571 820
H	−4.456 208	−2.467 507	1.405 322
O	−6.092 247	−1.112 794	0.045 838
C	−6.930 760	−0.308 073	−0.737 964
H	−7.144 217	−0.771 696	−1.717 013
H	−6.496 149	0.693 409	−0.903 084
H	−7.871 194	−0.201 296	−0.182 884
H	−4.945 532	−0.311 697	−2.283 929
C	−2.443 302	−0.651 795	−3.248 044
H	−2.156 332	−1.395 623	−4.006 379
H	−1.532 696	−0.100 478	−2.961 334
H	−3.144 058	0.053 395	−3.713 769
O	−0.109 764	0.804 823	−0.670 969
C	−0.133 667	2.042 361	−0.051 294
C	0.428 327	3.069 171	−1.048 809
C	1.395 734	4.021 805	−0.725 866
C	1.869 317	4.912 446	−1.693 149
C	1.380 739	4.860 016	−2.995 005
C	0.407 145	3.913 632	−3.324 369
C	−0.063 895	3.030 662	−2.359 379
H	−0.821 661	2.285 092	−2.607 489
H	0.013 027	3.867 143	−4.343 070
H	1.752 741	5.554 782	−3.752 195
H	2.628 474	5.650 805	−1.421 886
H	1.791 332	4.069 242	0.291 053
C	−1.571 163	2.456 585	0.313 705
C	−2.558 845	1.477 498	0.439 181
C	−3.857 024	1.817 271	0.819 919
C	−4.191 983	3.148 211	1.064 211
C	−3.219 194	4.137 047	0.916 556
C	−1.921 339	3.792 602	0.542 335
H	−1.168 299	4.575 264	0.419 260
H	−3.472 782	5.186 122	1.089 998
H	−5.209 057	3.416 560	1.361 679
H	−4.610 061	1.031 844	0.925 072
H	−2.290 978	0.439 557	0.230 346
C	0.752 568	1.995 653	1.213 202
C	0.411 719	2.559 867	2.446 358
C	1.304 838	2.520 829	3.517 825
C	2.553 336	1.916 050	3.377 362
C	2.899 832	1.333 775	2.159 939
C	2.004 747	1.368 765	1.092 646
H	2.290 158	0.934 441	0.134 027
H	3.868 553	0.843 124	2.032 273
H	3.250 184	1.892 037	4.219 074
H	1.018 423	2.967 406	4.473 548
H	−0.562 480	3.035 463	2.570 961

Intermediate XIX

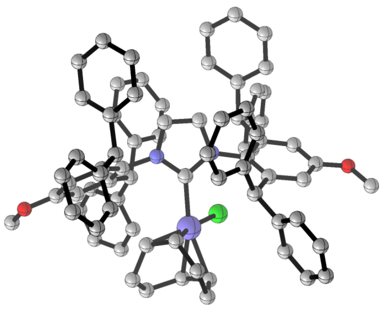

Rh	0.195 124	0.477 561	−2.166 914
C	0.017 724	0.097 176	−0.142 014
N	0.921 353	−0.479 303	0.702 967
C	0.452 578	−0.524 868	2.006 051
C	−0.761 769	0.059 538	1.992 451
N	−1.039 015	0.391 312	0.675 944
C	−2.360 890	0.812 761	0.309 924
C	−3.433 840	−0.090 014	0.477 366
C	−4.716 046	0.328 303	0.144 565
C	−4.957 200	1.627 075	−0.308 626
C	−3.894 867	2.523 121	−0.423 485
C	−2.590 956	2.121 964	−0.124 955
C	−1.435 376	3.097 739	−0.245 962
C	−1.792 195	4.312 668	−1.082 999
C	−2.073 337	4.126 823	−2.444 506

C	−2.417 652	5.201 151	−3.257 103
C	−2.489 986	6.488 983	−2.721 019
C	−2.220 083	6.683 633	−1.369 657
C	−1.874 734	5.602 493	−0.555 352
H	−1.663 324	5.766 350	0.503 335
H	−2.276 702	7.686 354	−0.938 867
H	−2.757 031	7.336 030	−3.357 390
H	−2.630 691	5.033 816	−4.315 980
H	−2.029 570	3.115 456	−2.856 433
C	−0.825 208	3.460 843	1.094 756
C	−1.538 245	3.355 459	2.292 240
C	−0.957 206	3.727 849	3.504 727
C	0.347 660	4.215 012	3.539 249
C	1.070 545	4.326 361	2.350 365
C	0.485 179	3.955 103	1.142 976
H	1.049 435	4.071 695	0.214 904
H	2.097 557	4.700 039	2.360 912
H	0.802 350	4.506 956	4.489 064
H	−1.532 152	3.635 292	4.429 468
H	−2.561 292	2.971 781	2.274 367
H	−0.658 914	2.554 240	−0.816 404
H	−4.061 002	3.544 923	−0.758 283
O	−6.235 464	1.935 092	−0.597 957
C	−6.522 018	3.214 958	−1.089 910
H	−5.976 023	3.425 884	−2.025 999
H	−7.600 371	3.240 052	−1.291 381
H	−6.277 538	4.002 632	−0.355 374
H	−5.558 356	−0.360 225	0.227 440
C	−3.242 262	−1.470 295	1.083 991
C	−3.290 863	−1.415 851	2.604 192
C	−2.491 219	−2.294 254	3.344 909
C	−2.504 654	−2.273 369	4.736 026
C	−3.322 711	−1.370 671	5.417 049
C	−4.128 083	−0.496 875	4.690 232
C	−4.112 532	−0.520 124	3.294 424
H	−4.739 301	0.177 630	2.734 964
H	−4.774 617	0.214 049	5.210 910
H	−3.330 179	−1.347 862	6.509 481
H	−1.863 263	−2.962 271	5.291 549
H	−1.836 909	−2.991 049	2.818 349
C	−4.199 905	−2.515 473	0.524 647
C	−4.163 605	−2.802 353	−0.846 750
C	−4.981 318	−3.789 020	−1.387 735
C	−5.857 058	−4.506 284	−0.570 405
C	−5.908 051	−4.220 376	0.790 687
C	−5.084 543	−3.232 297	1.334 598
H	−5.128 527	−3.025 846	2.405 417
H	−6.592 299	−4.769 927	1.442 087
H	−6.498 413	−5.282 240	−0.995 558
H	−4.931 732	−3.999 718	−2.458 943
H	−3.480 357	−2.239 503	−1.488 539
H	−2.232 514	−1.815 810	0.809 258
H	−1.462 955	0.259 463	2.792 811
H	1.022 802	−0.971 360	2.811 649
C	2.201 433	−0.986 356	0.333 429
C	2.285 568	−2.113 580	−0.498 178
C	3.548 847	−2.532 152	−0.921 061
C	4.703 585	−1.866 099	−0.504 201
C	4.601 579	−0.774 916	0.363 643
C	3.358 218	−0.322 577	0.790 563
C	3.261 977	0.856 953	1.745 077
C	4.388 606	1.862 364	1.553 254
C	5.387 446	2.059 529	2.509 871
C	6.407 261	2.988 237	2.294 458
C	6.440 763	3.736 471	1.121 329
C	5.447 107	3.547 205	0.159 724
C	4.433 710	2.618 660	0.374 346
H	3.669 342	2.466 312	−0.390 216
H	5.461 507	4.126 987	−0.766 532
H	7.237 403	4.465 388	0.954 622
H	7.179 303	3.126 827	3.055 314
H	5.366 634	1.485 028	3.438 047
C	3.136 641	0.400 044	3.189 196
C	2.412 070	1.188 056	4.090 499
C	2.266 722	0.800 619	5.418 778
C	2.848 691	−0.384 362	5.871 571
C	3.577 243	−1.172 625	4.983 746
C	3.720 146	−0.782 935	3.650 962
H	4.281 394	−1.414 444	2.958 422
H	4.035 861	−2.103 353	5.326 683
H	2.729 607	−0.694 013	6.912 545
H	1.684 023	1.422 746	6.102 647
H	1.932 913	2.102 192	3.733 323
H	2.327 289	1.391 797	1.508 471
H	5.514 737	−0.264 014	0.672 288
O	5.945 719	−2.211 784	−0.887 232
C	6.101 522	−3.280 818	−1.781 359
H	5.724 900	−4.228 408	−1.358 432
H	7.178 038	−3.377 255	−1.969 447
H	5.584 033	−3.093 008	−2.738 165
H	3.612 238	−3.386 519	−1.591 718
C	1.031 826	−2.863 772	−0.918 393
C	0.379 086	−3.635 457	0.215 863
C	1.044 249	−3.982 286	1.394 002
C	0.419 346	−4.788 804	2.348 192
C	−0.875 191	−5.257 100	2.134 214
C	−1.555 806	−4.896 290	0.968 786
C	−0.932 474	−4.090 295	0.022 448
H	−1.462 722	−3.809 905	−0.891 220
H	−2.582 890	−5.229 046	0.799 845
H	−1.361 765	−5.890 093	2.880 078
H	0.954 232	−5.056 044	3.263 158
H	2.063 625	−3.626 569	1.565 273
C	1.252 417	−3.782 277	−2.105 552
C	0.872 305	−3.361 349	−3.382 218
C	1.096 157	−4.172 300	−4.494 312
C	1.700 540	−5.418 727	−4.342 447
C	2.061 263	−5.857 984	−3.068 094
C	1.831 535	−5.048 675	−1.957 616
H	2.093 160	−5.406 589	−0.958 104
H	2.514 570	−6.843 814	−2.936 774
H	1.874 641	−6.056 196	−5.212 762
H	0.785 398	−3.829 323	−5.484 429
H	0.366 584	−2.398 917	−3.486 019
H	0.299 212	−2.112 099	−1.254 557
C	2.267 880	0.911 080	−2.231 403
C	1.546 780	2.057 064	−1.840 297
C	1.286 895	3.235 575	−2.744 907
H	2.210 609	3.833 088	−2.866 272
H	0.565 100	3.903 070	−2.252 077
C	0.730 621	2.822 412	−4.115 655
C	−0.028 635	1.511 865	−4.077 234

C	0.534 774	0.264 834	−4.315 964
C	1.991 868	−0.015 492	−4.561 586
H	2.254 544	0.203 017	−5.613 972
H	2.149 656	−1.096 215	−4.426 275
C	2.902 817	0.746 822	−3.598 619
H	3.854 138	0.205 498	−3.485 460
H	3.165 941	1.735 174	−4.006 974
H	−0.160 105	−0.532 084	−4.598 920
H	−1.117 605	1.569 919	−4.178 326
H	0.059 383	3.612 545	−4.483 952
H	1.541 673	2.747 709	−4.856 095
H	1.553 110	2.297 036	−0.771 455
H	2.756 393	0.339 734	−1.444 746
Cl	−1.760 537	−0.820 497	−2.479 598

Intermediate XX

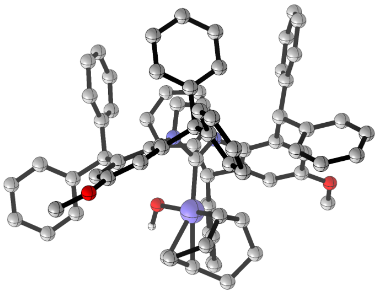

Rh	0.194 100	−1.027 231	−1.901 636
C	−0.042 000	−0.168 421	−0.064 380
N	0.998 390	0.144 226	0.755 755
C	0.555 026	0.432 577	2.040 467
C	−0.792 747	0.320 439	2.017 009
N	−1.142 910	−0.029 522	0.717 464
C	−2.479 960	−0.033 811	0.210 253
C	−3.125 417	−1.243 559	−0.080 245
C	−4.407 179	−1.206 054	−0.634 840
C	−5.037 650	0.010 218	−0.899 950
C	−4.394 377	1.205 629	−0.571 810
C	−3.124 692	1.200 812	−0.006 558
C	−2.497 670	2.512 643	0.437 159
C	−2.622 563	3.597 630	−0.615 654
C	−1.611 146	3.746 931	−1.568 247
C	−1.700 307	4.712 794	−2.567 727
C	−2.814 151	5.547 329	−2.631 086
C	−3.829 084	5.411 774	−1.684 337
C	−3.731 777	4.448 867	−0.682 051
H	−4.519 445	4.368 800	0.071 292
H	−4.702 670	6.067 180	−1.721 104
H	−2.888 265	6.308 454	−3.411 308
H	−0.889 467	4.816 410	−3.292 421
H	−0.739 025	3.091 774	−1.528 863
C	−3.002 553	2.950 584	1.808 367
C	−2.648 990	4.213 709	2.302 583
C	−3.041 908	4.623 225	3.573 211
C	−3.798 929	3.775 426	4.382 400
C	−4.164 663	2.522 141	3.900 284
C	−3.773 787	2.116 649	2.623 469
H	−4.078 170	1.134 894	2.255 556
H	−4.765 297	1.850 058	4.518 076
H	−4.107 799	4.095 464	5.380 207
H	−2.757 677	5.615 350	3.933 283
H	−2.069 330	4.892 981	1.672 918
H	−1.418 347	2.328 422	0.561 558
H	−4.911 689	2.142 749	−0.777 677
O	−6.259 207	0.119 499	−1.454 044
C	−6.931 979	−1.051 270	−1.831 222
H	−6.354 924	−1.635 291	−2.569 118
H	−7.149 128	−1.698 079	−0.963 238
H	−7.879 603	−0.735 838	−2.285 483
H	−4.895 993	−2.151 773	−0.863 304
C	−2.480 934	−2.572 416	0.252 244
C	−2.531 251	−2.890 310	1.731 766
C	−3.385 826	−2.241 157	2.626 960
C	−3.392 429	−2.577 532	3.981 742
C	−2.551 005	−3.578 672	4.460 134
C	−1.701 041	−4.241 965	3.572 851
C	−1.688 983	−3.896 001	2.225 149
H	−1.017 370	−4.406 197	1.529 259
H	−1.034 096	−5.027 813	3.936 724
H	−2.557 913	−3.845 254	5.519 829
H	−4.065 314	−2.054 269	4.665 970
H	−4.058 589	−1.462 926	2.258 550
C	−3.028 824	−3.685 645	−0.620 284
C	−2.708 498	−3.665 725	−1.984 919
C	−3.202 973	−4.636 453	−2.849 325
C	−4.031 372	−5.650 668	−2.363 178
C	−4.357 152	−5.678 271	−1.009 749
C	−3.859 265	−4.701 976	−0.143 464
H	−4.117 039	−4.733 370	0.917 659
H	−5.004 599	−6.467 420	−0.618 937
H	−4.419 247	−6.416 898	−3.038 842
H	−2.939 639	−4.604 092	−3.910 013
H	−2.066 516	−2.861 796	−2.355 614
H	−1.406 240	−2.512 780	−0.020 047
H	−1.532 939	0.458 736	2.798 061
H	1.234 066	0.680 231	2.849 674
C	2.347 097	0.218 580	0.270 771
C	3.156 932	−0.925 428	0.278 289
C	4.414 947	−0.865 565	−0.326 997
C	4.868 350	0.318 206	−0.909 309
C	4.089 348	1.477 227	−0.822 738
C	2.836 597	1.446 080	−0.223 656
C	2.053 656	2.730 540	0.001 807
C	2.550 304	3.902 379	−0.829 817
C	2.116 809	4.063 955	−2.150 942
C	2.584 035	5.114 433	−2.936 815
C	3.499 194	6.026 899	−2.414 184
C	3.936 826	5.878 502	−1.100 037
C	3.463 703	4.827 963	−0.314 908
H	3.804 464	4.728 996	0.717 774
H	4.650 491	6.589 400	−0.676 549
H	3.866 504	6.852 641	−3.028 072
H	2.228 628	5.219 665	−3.964 898
H	1.405 188	3.353 820	−2.575 963
C	2.005 193	3.072 361	1.482 487
C	3.029 434	2.705 085	2.360 075
C	2.938 584	3.002 043	3.719 781
C	1.822 039	3.669 405	4.218 981
C	0.804 392	4.056 239	3.346 595
C	0.903 424	3.766 972	1.989 203
H	0.103 576	4.066 270	1.307 237
H	−0.081 734	4.568 407	3.727 182

H	1.741 914	3.885 836	5.286 794
H	3.741 733	2.696 365	4.394 559
H	3.893 785	2.156 140	1.979 518
H	1.012 326	2.535 962	−0.301 724
H	4.489 486	2.405 233	−1.231 767
O	6.049 541	0.438 742	−1.543 430
C	6.862 084	−0.696 897	−1.667 028
H	6.349 319	−1.509 445	−2.210 633
H	7.183 337	−1.081 664	−0.683 338
H	7.748 285	−0.388 297	−2.235 620
H	5.027 777	−1.765 268	−0.332 670
C	2.693 343	−2.178 353	0.999 531
C	2.542 719	−1.903 578	2.482 322
C	1.465 989	−2.458 877	3.177 936
C	1.265 266	−2.182 067	4.527 372
C	2.151 555	−1.348 592	5.209 451
C	3.240 181	−0.801 470	4.530 823
C	3.432 866	−1.076 480	3.177 309
H	4.271 529	−0.620 919	2.643 995
H	3.939 895	−0.146 741	5.056 483
H	1.991 668	−1.120 493	6.266 060
H	0.396 193	−2.604 377	5.037 810
H	0.750 847	−3.081 856	2.638 767
C	3.567 354	−3.380 725	0.699 969
C	4.611 603	−3.791 252	1.533 647
C	5.422 854	−4.870 126	1.179 048
C	5.199 321	−5.556 478	−0.012 607
C	4.150 836	−5.162 386	−0.845 205
C	3.342 721	−4.086 559	−0.489 074
H	2.505 515	−3.770 468	−1.115 359
H	3.957 389	−5.702 621	−1.775 638
H	5.833 738	−6.402 611	−0.287 967
H	6.233 470	−5.178 463	1.844 319
H	4.789 090	−3.265 897	2.474 704
H	1.697 002	−2.422 447	0.583 510
C	0.518 594	0.805 648	−2.887 854
C	−0.841 651	0.434 263	−2.961 540
C	−1.522 740	−0.178 985	−4.159 379
H	−1.810 888	0.600 896	−4.890 022
H	−2.467 482	−0.619 403	−3.801 137
C	−0.681 202	−1.272 735	−4.830 703
C	0.141 329	−2.052 682	−3.826 030
C	1.454 982	−1.757 708	−3.460 329
C	2.296 423	−0.611 013	−3.957 453
H	2.748 728	−0.859 283	−4.936 609
H	3.133 633	−0.494 647	−3.250 829
C	1.520 075	0.706 578	−4.022 446
H	2.230 492	1.545 575	−3.958 043
H	1.011 643	0.819 291	−4.992 294
H	2.028 752	−2.564 350	−2.989 542
H	−0.204 333	−3.071 344	−3.615 304
H	−1.345 913	−1.966 139	−5.368 754
H	−0.021 745	−0.840 202	−5.597 982
H	−1.537 124	0.942 904	−2.284 097
H	0.774 833	1.542 084	−2.123 570
O	0.347 412	−2.746 507	−0.850 710
H	0.311 559	−3.510 905	−1.436 654
C	5.597 564	2.843 278	−3.701 978
O	4.438 861	2.165 965	−4.107 158
C	3.421 684	2.050 552	−3.227 082
C	2.309 515	1.321 065	−3.658 369
C	1.233 160	1.084 361	−2.811 848
C	1.254 467	1.637 691	−1.517 520
C	2.348 472	2.398 502	−1.081 827
C	3.443 582	2.572 818	−1.935 781
H	4.310 790	3.113 041	−1.556 249
C	2.357 798	3.090 739	0.269 341
C	3.692 346	2.940 489	0.983 554
C	4.547 057	4.013 808	1.242 321
C	5.749 467	3.809 573	1.924 196
C	6.104 933	2.535 158	2.359 184
C	5.254 626	1.456 934	2.103 322
C	4.062 058	1.660 945	1.417 735
H	3.396 035	0.821 833	1.203 943
H	5.513 295	0.449 884	2.440 954
H	7.042 216	2.380 631	2.899 395
H	6.408 258	4.659 108	2.121 719
H	4.267 186	5.019 682	0.921 043
C	1.893 925	4.537 704	0.174 202
C	1.224 723	5.099 502	1.268 460
C	0.782 816	6.418 202	1.240 076
C	0.999 885	7.207 076	0.109 754
C	1.659 735	6.658 847	−0.986 964
C	2.101 234	5.334 655	−0.955 645
H	2.599 528	4.914 793	−1.831 282
H	1.829 885	7.262 444	−1.882 047
H	0.649 419	8.241 625	0.082 169
H	0.255 912	6.828 573	2.105 243
H	1.036 736	4.483 437	2.149 103
H	1.626 980	2.573 829	0.909 038
N	0.106 612	1.548 000	−0.668 187
C	−0.573 303	2.716 040	−0.353 886
C	−1.517 675	2.387 001	0.546 145
N	−1.433 298	1.015 085	0.723 249
C	−0.412 094	0.458 549	−0.009 504
Rh	0.343 562	−1.440 666	−0.031 985
C	−1.432 571	−2.092 175	−0.985 276
C	−1.454 767	−2.519 209	0.357 293
C	−1.470 667	−3.982 497	0.703 182
H	−0.863 624	−4.578 499	0.009 904
H	−1.025 730	−4.143 664	1.696 550
C	−2.890 271	−4.536 685	0.755 804
C	−3.708 784	−4.719 661	−0.490 181
C	−3.625 185	−4.358 748	−1.781 560
C	−2.698 748	−3.546 614	−2.639 497
H	−2.521 436	−4.133 115	−3.559 106
H	−3.288 677	−2.677 483	−2.992 311
C	−1.345 545	−3.065 549	−2.128 620
H	−0.741 495	−3.934 914	−1.846 557
H	−0.822 563	−2.601 115	−2.979 169
H	−4.468 381	−4.726 782	−2.382 682
H	−4.611 711	−5.305 612	−0.269 030
H	−3.489 786	−3.914 942	1.450 515
H	−2.853 330	−5.527 280	1.244 679
H	−2.034 661	−1.923 405	1.065 724

TS1a

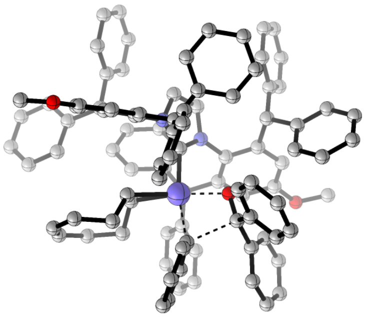

H	−1.999 716	−1.185 581	−1.230 034
O	2.227 514	−0.664 786	0.156 769
C	3.003 648	−1.682 298	0.241 624
C	3.855 295	−2.004 988	−0.947 138
C	4.442 295	−3.258 213	−1.172 831
C	5.241 271	−3.462 981	−2.291 723
C	5.470 208	−2.422 070	−3.197 028
C	4.898 964	−1.172 373	−2.972 397
C	4.097 631	−0.968 550	−1.849 677
H	3.639 665	−0.001 194	−1.652 189
H	5.058 017	−0.351 360	−3.675 640
H	6.091 882	−2.591 651	−4.079 655
H	5.679 734	−4.448 132	−2.468 405
H	4.235 906	−4.084 491	−0.489 345
C	3.582 050	−1.949 986	1.600 421
C	4.883 259	−2.426 326	1.802 957
C	5.397 222	−2.545 770	3.094 226
C	4.624 311	−2.193 111	4.198 693
C	3.329 260	−1.707 270	4.007 419
C	2.825 885	−1.583 434	2.719 882
H	1.816 919	−1.210 050	2.553 179
H	2.703 211	−1.432 176	4.859 501
H	5.029 530	−2.293 920	5.208 379
H	6.417 083	−2.912 332	3.234 035
H	5.513 314	−2.678 028	0.949 830
C	1.509 040	−3.323 085	0.036 260
C	1.619 210	−4.138 672	1.174 170
C	1.712 459	−5.527 399	1.082 944
C	1.698 666	−6.143 615	−0.168 201
C	1.621 238	−5.358 832	−1.320 877
C	1.564 562	−3.971 509	−1.210 156
H	1.556 845	−3.376 509	−2.124 876
H	1.623 226	−5.828 229	−2.308 434
H	1.768 053	−7.231 750	−0.247 185
H	1.797 551	−6.129 944	1.991 428
H	1.644 946	−3.684 194	2.167 369
C	−2.397 357	0.341 921	1.532 132
C	−2.023 098	−0.176 136	2.787 057
C	−2.980 090	−0.823 473	3.556 029
C	−4.294 620	−0.968 034	3.096 456
C	−4.661 309	−0.420 367	1.867 967
C	−3.720 715	0.250 499	1.079 221
C	−4.164 083	0.920 600	−0.210 111
C	−5.273 561	0.169 184	−0.933 421
C	−5.137 205	−1.205 726	−1.168 992
C	−6.109 414	−1.906 924	−1.875 759
C	−7.237 480	−1.248 690	−2.366 999
C	−7.385 690	0.115 383	−2.133 794
C	−6.412 890	0.817 035	−1.419 944
H	−6.541 998	1.886 310	−1.244 971
H	−8.266 458	0.644 437	−2.506 219
H	−7.998 916	−1.800 290	−2.923 657
H	−5.977 104	−2.978 538	−2.036 172
H	−4.266 206	−1.743 148	−0.785 233
C	−4.517 724	2.381 344	0.025 921
C	−5.157 615	2.810 939	1.191 770
C	−5.471 311	4.157 970	1.376 830
C	−5.151 029	5.095 082	0.396 777
C	−4.516 436	4.674 122	−0.772 302
C	−4.202 863	3.330 261	−0.953 299
H	−3.690 532	3.008 764	−1.862 127
H	−4.252 435	5.398 679	−1.546 672
H	−5.390 695	6.150 757	0.544 829
H	−5.965 902	4.475 900	2.298 018
H	−5.400 840	2.087 315	1.972 804
H	−3.301 238	0.918 565	−0.897 566
H	−5.677 984	−0.517 587	1.489 133
O	−5.134 508	−1.647 201	3.898 897
C	−6.444 088	−1.878 951	3.456 481
H	−6.936 310	−2.478 161	4.232 607
H	−6.460 379	−2.438 034	2.504 619
H	−7.005 582	−0.937 555	3.322 690
H	−2.717 757	−1.260 133	4.519 882
C	−0.585 508	−0.057 570	3.250 215
C	−0.244 071	−1.081 059	4.317 685
C	−0.009 627	−0.743 956	5.652 371
C	0.294 253	−1.734 396	6.589 599
C	0.369 561	−3.070 360	6.203 335
C	0.133 868	−3.415 096	4.871 042
C	−0.171 844	−2.427 656	3.941 914
H	−0.347 393	−2.686 155	2.895 126
H	0.196 616	−4.458 367	4.551 378
H	0.613 702	−3.842 325	6.936 941
H	0.476 316	−1.454 284	7.630 099
H	−0.057 331	0.302 073	5.962 402
C	−0.188 018	1.346 193	3.655 822
C	1.169 694	1.692 232	3.644 959
C	1.596 287	2.927 939	4.121 144
C	0.664 713	3.847 890	4.607 029
C	−0.690 833	3.525 510	4.594 599
C	−1.114 275	2.281 309	4.124 002
H	−2.177 480	2.027 891	4.130 184
H	−1.427 959	4.244 012	4.961 743
H	0.996 229	4.819 342	4.981 716
H	2.660 812	3.174 249	4.097 034
H	1.906 889	0.977 379	3.273 072
H	0.018 138	−0.325 289	2.360 853
H	−2.244 545	2.991 689	1.074 846
H	−0.307 543	3.670 649	−0.793 150
C	0.052 879	0.247 971	−3.265 486
C	0.433 280	−0.716 845	−4.376 354
C	1.439 566	−1.656 421	−4.113 750
C	1.829 147	−2.581 289	−5.074 633
C	1.212 569	−2.585 078	−6.327 814
C	0.217 699	−1.651 480	−6.603 446
C	−0.168 719	−0.720 726	−5.635 446
H	−0.950 587	0.005 759	−5.865 174
H	−0.266 892	−1.641 056	−7.582 942
H	1.511 585	−3.311 545	−7.087 280
H	2.620 379	−3.298 037	−4.840 749
H	1.934 302	−1.639 896	−3.140 620
C	−1.177 249	1.069 920	−3.591 467
C	−1.102 051	2.409 893	−3.980 825
C	−2.250 943	3.107 181	−4.358 304
C	−3.492 807	2.474 682	−4.351 817
C	−3.583 582	1.141 298	−3.948 870
C	−2.435 212	0.451 552	−3.571 101
H	−2.510 010	−0.596 948	−3.275 489
H	−4.552 722	0.637 606	−3.917 154
H	−4.391 615	3.020 849	−4.647 656
H	−2.171 189	4.153 193	−4.664 937
H	−0.130 351	2.909 468	−3.998 687

H	−0.200 929	−0.388 939	−2.396 124
H	2.324 596	0.900 573	−4.664 122
H	6.074 510	2.353 238	−2.835 273
H	6.289 649	2.818 598	−4.552 993
H	5.387 048	3.895 707	−3.442 827

TS1b

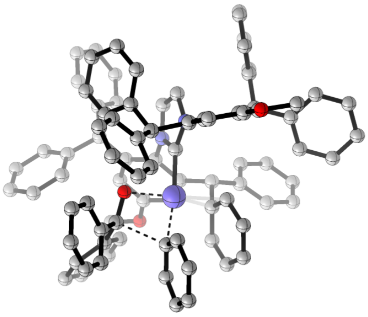

Rh	0.301 749	0.719 957	−1.178 531
C	−0.467 662	−0.356 811	0.321 859
N	−1.430 129	−0.089 269	1.252 088
C	−1.490 714	−1.087 509	2.217 677
C	−0.581 928	−2.024 797	1.872 085
N	0.032 060	−1.563 177	0.714 242
C	0.864 574	−2.336 094	−0.135 820
C	2.080 206	−2.841 461	0.327 720
C	2.873 748	−3.599 258	−0.538 595
C	2.450 972	−3.853 196	−1.841 326
C	1.218 301	−3.358 753	−2.285 566
C	0.418 040	−2.587 930	−1.452 173
C	−0.873 843	−1.975 404	−1.950 837
C	−0.630 717	−0.602 067	−2.586 830
C	0.032 647	−0.594 425	−3.867 253
C	−0.228 050	0.368 544	−4.802 665
C	−1.164 331	1.407 408	−4.540 953
C	−1.787 810	1.468 200	−3.324 819
C	−1.520 667	0.496 008	−2.306 301
H	−2.283 223	0.391 461	−1.532 620
H	−2.520 657	2.251 758	−3.116 389
H	−1.367 141	2.159 634	−5.305 751
H	0.284 909	0.338 953	−5.766 916
H	0.719 613	−1.409 713	−4.105 449
C	−1.722 910	−2.914 567	−2.793 510
C	−2.425 908	−2.478 797	−3.921 272
C	−3.289 462	−3.340 331	−4.599 169
C	−3.467 622	−4.649 980	−4.162 844
C	−2.774 372	−5.094 973	−3.036 955
C	−1.913 576	−4.234 901	−2.361 434
H	−1.375 218	−4.590 496	−1.478 670
H	−2.906 231	−6.119 912	−2.681 178
H	−4.144 471	−5.322 480	−4.695 055
H	−3.827 827	−2.977 922	−5.478 511
H	−2.301 310	−1.454 589	−4.276 620
H	−1.494 374	−1.780 738	−1.063 552
H	0.898 815	−3.596 553	−3.301 367
O	3.178 854	−4.538 620	−2.748 579
C	4.425 139	−5.047 887	−2.356 773
H	4.844 904	−5.558 624	−3.232 418
H	5.114 486	−4.244 711	−2.042 158
H	4.327 841	−5.773 561	−1.530 343
H	3.838 775	−3.953 632	−0.178 250
C	2.544 524	−2.583 171	1.747 207
C	4.008 739	−2.181 882	1.764 067
C	5.029 511	−3.061 758	2.133 083
C	6.364 920	−2.661 322	2.067 646
C	6.691 696	−1.377 538	1.636 402
C	5.676 510	−0.493 403	1.269 497
C	4.345 300	−0.893 545	1.331 528
H	3.549 891	−0.210 920	1.018 484
H	5.919 564	0.513 218	0.923 246
H	7.737 335	−1.064 222	1.586 747
H	7.154 018	−3.358 971	2.359 630
H	4.777 442	−4.066 509	2.481 520
C	2.209 398	−3.719 629	2.699 660
C	1.677 690	−4.938 596	2.269 477
C	1.351 413	−5.940 440	3.186 202
C	1.552 449	−5.739 328	4.548 285
C	2.085 727	−4.526 990	4.989 701
C	2.409 268	−3.531 297	4.074 617
H	2.831 558	−2.587 942	4.427 196
H	2.248 708	−4.356 013	6.056 659
H	1.295 576	−6.522 131	5.265 819
H	0.935 290	−6.884 885	2.826 880
H	1.511 203	−5.106 854	1.203 413
H	1.984 629	−1.705 578	2.106 674
H	−0.314 765	−2.967 308	2.339 983
H	−2.169 462	−1.034 377	3.060 710
C	−2.350 352	1.002 642	1.206 965
C	−3.662 221	0.761 850	0.769 644
C	−4.577 608	1.818 065	0.755 307
C	−4.201 599	3.081 961	1.207 808
C	−2.894 466	3.301 506	1.656 193
C	−1.950 428	2.282 869	1.644 079
C	−0.508 554	2.515 219	2.073 996
C	−0.105 289	1.603 962	3.215 125
C	−0.937 506	1.406 737	4.321 637
C	−0.539 288	0.578 996	5.370 600
C	0.701 543	−0.056 467	5.330 024
C	1.538 836	0.142 166	4.233 109
C	1.134 486	0.959 419	3.180 087
H	1.768 565	1.082 391	2.298 605
H	2.518 330	−0.340 340	4.189 228
H	1.013 179	−0.707 532	6.150 373
H	−1.203 151	0.428 706	6.225 747
H	−1.912 427	1.901 347	4.354 357
C	−0.174 409	3.975 357	2.342 202
C	0.253 839	4.434 538	3.590 505
C	0.573 919	5.779 662	3.787 662
C	0.473 358	6.689 273	2.739 141
C	0.054 915	6.241 992	1.485 585
C	−0.261 169	4.902 433	1.293 963
H	−0.587 864	4.565 057	0.308 911
H	−0.013 291	6.938 334	0.646 663
H	0.725 932	7.741 011	2.893 678
H	0.905 520	6.113 944	4.773 950
H	0.345 269	3.732 794	4.421 212
H	0.112 310	2.215 035	1.210 139
H	−2.631 917	4.302 595	1.997 173
O	−5.028 162	4.143 741	1.244 130
C	−6.343 316	3.986 563	0.787 474
H	−6.832 418	4.962 678	0.895 941

H	−6.376 018	3.684 239	−0.273 816
H	−6.898 187	3.239 996	1.382 801
H	−5.583 043	1.630 807	0.382 438
C	−4.107 569	−0.637 072	0.363 123
C	−5.244 146	−0.643 430	−0.646 360
C	−6.583 486	−0.603 409	−0.240 421
C	−7.612 192	−0.578 426	−1.180 301
C	−7.318 679	−0.609 391	−2.543 049
C	−5.989 444	−0.671 800	−2.956 468
C	−4.962 852	−0.687 009	−2.015 136
H	−3.927 748	−0.746 823	−2.354 432
H	−5.742 617	−0.717 803	−4.019 824
H	−8.125 386	−0.598 856	−3.279 942
H	−8.651 624	−0.547 881	−0.844 109
H	−6.823 423	−0.609 089	0.825 744
C	−4.442 509	−1.507 626	1.563 046
C	−4.880 007	−0.973 242	2.777 371
C	−5.174 520	−1.809 296	3.855 655
C	−5.036 834	−3.189 568	3.730 662
C	−4.610 279	−3.731 164	2.516 750
C	−4.319 442	−2.896 392	1.442 909
H	−3.978 779	−3.322 381	0.494 631
H	−4.497 733	−4.812 739	2.409 228
H	−5.259 752	−3.843 912	4.576 670
H	−5.508 708	−1.375 359	4.801 345
H	−4.969 996	0.110 347	2.887 691
H	−3.249 277	−1.116 264	−0.131 506
O	1.909 605	1.233 949	0.017 159
C	2.673 275	1.838 020	−0.818 935
C	1.212 256	2.224 821	−2.466 587
C	1.887 529	2.144 848	−3.692 417
C	1.815 696	3.173 896	−4.632 085
C	1.062 167	4.316 736	−4.360 063
C	0.394 162	4.431 571	−3.139 272
C	0.501 165	3.407 524	−2.202 704
H	0.014 830	3.523 202	−1.229 673
H	−0.190 740	5.327 336	−2.913 351
H	1.009 705	5.126 473	−5.092 856
H	2.354 653	3.086 943	−5.579 803
H	2.491 564	1.262 834	−3.920 229
C	3.023 422	3.260 027	−0.502 904
C	2.914 620	3.679 693	0.826 555
C	3.263 601	4.977 609	1.193 019
C	3.715 957	5.874 547	0.229 991
C	3.823 751	5.465 104	−1.101 796
C	3.485 001	4.168 229	−1.466 547
H	3.537 186	3.861 179	−2.513 142
H	4.163 394	6.168 456	−1.865 893
H	3.977 645	6.897 312	0.512 361
H	3.159 907	5.288 141	2.234 750
H	2.554 683	2.976 492	1.576 976
C	3.690 807	1.014 178	−1.554 209
C	5.013 949	1.450 495	−1.697 856
C	5.969 885	0.633 806	−2.301 324
C	5.616 329	−0.625 328	−2.777 152
C	4.300 517	−1.071 131	−2.634 831
C	3.351 698	−0.262 738	−2.022 194
H	2.312 738	−0.595 318	−1.920 235
H	4.007 433	−2.051 661	−3.014 778
H	6.362 291	−1.259 466	−3.262 639
H	6.998 849	0.988 782	−2.396 957
H	5.307 312	2.429 453	−1.316 385

TS2a

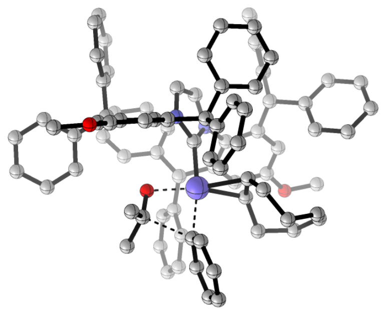

C	−6.685 011	2.703 830	−1.564 102
O	−5.392 863	3.161 707	−1.850 714
C	−4.358 485	2.314 901	−1.684 633
C	−3.087 369	2.829 340	−1.960 145
C	−1.945 117	2.054 902	−1.790 401
C	−2.092 300	0.720 593	−1.362 411
C	−3.364 202	0.178 335	−1.128 906
C	−4.491 782	0.991 430	−1.268 243
H	−5.468 727	0.562 568	−1.047 198
C	−3.544 698	−1.297 837	−0.818 866
C	−4.733 873	−1.571 187	0.088 343
C	−5.943 053	−2.082 588	−0.390 646
C	−7.015 372	−2.302 809	0.475 997
C	−6.889 957	−2.023 148	1.834 322
C	−5.682 863	−1.519 085	2.321 768
C	−4.616 371	−1.293 242	1.456 338
H	−3.670 933	−0.887 067	1.824 527
H	−5.569 589	−1.305 923	3.388 145
H	−7.726 825	−2.203 080	2.513 511
H	−7.952 918	−2.704 769	0.083 626
H	−6.045 019	−2.322 756	−1.451 309
C	−3.598 941	−2.122 750	−2.097 002
C	−3.120 343	−3.437 228	−2.074 896
C	−3.141 032	−4.224 860	−3.221 200
C	−3.644 531	−3.711 319	−4.417 295
C	−4.128 139	−2.405 451	−4.449 969
C	−4.105 242	−1.616 835	−3.298 066
H	−4.469 644	−0.587 987	−3.340 458
H	−4.522 843	−1.990 276	−5.380 757
H	−3.655 383	−4.325 685	−5.320 831
H	−2.749 620	−5.244 482	−3.180 240
H	−2.707 662	−3.838 707	−1.148 325
H	−2.653 401	−1.627 585	−0.263 793
N	−0.952 588	−0.135 904	−1.235 890
C	−0.728 456	−1.161 454	−2.142 928
C	0.293 420	−1.894 477	−1.658 043
N	0.699 028	−1.276 527	−0.484 074
C	−0.061 308	−0.174 818	−0.193 236
Rh	−0.108 547	1.111 443	1.370 133
C	1.683 771	2.020 591	0.638 982
C	1.969 547	1.147 931	1.714 236
C	2.561 882	1.501 777	3.052 614
H	2.180 902	2.444 424	3.451 736
H	2.268 249	0.727 765	3.779 940

C	4.089 161	1.519 015	2.992 671
C	4.735 828	2.540 701	2.106 365
C	4.222 141	3.638 922	1.534 200
C	2.816 928	4.168 529	1.589 106
H	2.391 519	4.086 875	2.597 040
H	2.843 261	5.248 426	1.371 569
C	1.868 956	3.523 189	0.571 259
H	0.902 716	4.041 677	0.637 648
H	2.244 484	3.762 619	−0.439 292
H	4.913 709	4.225 191	0.915 246
H	5.799 772	2.350 273	1.914 909
H	4.440 657	0.520 965	2.670 743
H	4.492 776	1.639 657	4.016 330
H	2.311 378	0.155 227	1.416 019
H	1.821 713	1.555 803	−0.343 421
O	−2.042 574	0.535 128	1.718 500
C	−2.385 434	1.490 988	2.500 715
C	−3.183 588	2.610 018	1.880 528
C	−2.793 432	1.102 949	3.899 889
C	−0.531 454	2.567 894	2.997 086
C	−0.148 842	2.320 875	4.328 072
C	0.196 327	3.341 649	5.211 061
C	0.149 479	4.671 541	4.791 647
C	−0.265 768	4.959 926	3.492 225
C	−0.617 678	3.920 960	2.631 466
H	−0.968 120	4.180 949	1.631 503
H	−0.320 979	5.996 681	3.149 065
H	0.419 770	5.478 136	5.477 888
H	0.507 435	3.099 219	6.231 012
H	−0.103 765	1.294 159	4.694 605
C	1.805 704	−1.737 298	0.285 048
C	1.585 057	−2.271 641	1.570 080
C	2.687 413	−2.596 417	2.349 006
C	3.990 214	−2.402 247	1.873 597
C	4.191 709	−1.939 799	0.573 594
C	3.099 883	−1.616 342	−0.237 295
C	3.330 030	−1.201 859	−1.678 169
C	4.582 610	−0.358 986	−1.868 936
C	5.583 785	−0.700 371	−2.781 853
C	6.694 346	0.126 361	−2.965 077
C	6.818 180	1.307 156	−2.238 917
C	5.826 878	1.652 271	−1.318 588
C	4.722 940	0.826 527	−1.132 892
H	3.965 450	1.106 864	−0.396 308
H	5.905 721	2.571 644	−0.734 748
H	7.684 374	1.956 876	−2.385 547
H	7.465 509	−0.159 165	−3.684 929
H	5.494 044	−1.620 368	−3.362 566
C	3.283 532	−2.412 186	−2.600 335
C	3.755 544	−3.668 697	−2.208 589
C	3.705 809	−4.754 225	−3.083 874
C	3.180 387	−4.601 221	−4.364 921
C	2.704 242	−3.352 310	−4.764 375
C	2.755 890	−2.270 940	−3.889 357
H	2.363 057	−1.300 582	−4.198 679
H	2.279 597	−3.219 443	−5.762 577
H	3.135 894	−5.452 946	−5.047 838
H	4.077 040	−5.728 608	−2.756 671
H	4.157 135	−3.805 533	−1.202 438
H	2.488 642	−0.554 703	−1.974 985
H	5.194 474	−1.794 643	0.172 619
O	4.985 621	−2.687 627	2.733 526
C	6.306 785	−2.446 235	2.333 175
H	6.945 524	−2.699 981	3.188 337
H	6.464 939	−1.386 342	2.067 352
H	6.596 881	−3.073 747	1.472 088
H	2.560 054	−2.980 523	3.361 350
C	0.170 880	−2.470 869	2.083 576
C	0.128 632	−2.543 214	3.596 700
C	0.218 940	−1.346 503	4.313 275
C	0.235 953	−1.346 672	5.704 636
C	0.161 614	−2.553 268	6.402 281
C	0.071 487	−3.751 013	5.696 177
C	0.055 026	−3.747 266	4.300 628
H	−0.020 524	−4.689 538	3.752 835
H	0.012 425	−4.699 977	6.234 935
H	0.172 810	−2.558 714	7.494 848
H	0.307 388	−0.401 829	6.250 202
H	0.284 148	−0.414 892	3.741 408
C	−0.551 860	−3.637 081	1.437 272
C	−1.927 550	−3.773 287	1.672 026
C	−2.635 568	−4.860 297	1.171 398
C	−1.979 379	−5.832 060	0.411 879
C	−0.617 732	−5.695 628	0.154 009
C	0.092 319	−4.607 343	0.666 450
H	1.163 282	−4.516 946	0.469 634
H	−0.095 595	−6.445 129	−0.445 998
H	−2.533 026	−6.686 343	0.015 047
H	−3.708 163	−4.940 101	1.364 052
H	−2.443 887	−3.019 238	2.270 790
H	−0.384 814	−1.554 859	1.815 086
H	0.770 378	−2.792 576	−2.033 002
H	−1.323 143	−1.275 666	−3.041 137
C	−0.559 158	2.621 671	−2.048 610
C	−0.551 366	4.133 158	−2.220 884
C	−0.929 878	4.941 965	−1.141 546
C	−0.929 088	6.328 297	−1.239 796
C	−0.548 034	6.941 806	−2.434 222
C	−0.177 486	6.151 661	−3.518 089
C	−0.179 257	4.759 262	−3.412 826
H	0.119 528	4.154 604	−4.270 885
H	0.119 863	6.618 934	−4.460 103
H	−0.542 521	8.031 182	−2.517 351
H	−1.226 519	6.933 165	−0.379 608
H	−1.245 907	4.462 847	−0.213 661
C	0.166 954	1.931 585	−3.185 051
C	1.566 633	1.896 807	−3.184 357
C	2.271 057	1.367 899	−4.262 592
C	1.580 629	0.857 940	−5.363 336
C	0.186 856	0.873 083	−5.368 371
C	−0.513 867	1.408 747	−4.287 802
H	−1.606 804	1.428 313	−4.300 996
H	−0.362 020	0.471 223	−6.223 734
H	2.129 793	0.445 973	−6.213 599
H	3.363 681	1.349 419	−4.235 856
H	2.115 233	2.312 475	−2.335 552
H	0.006 295	2.409 817	−1.122 961
H	−3.014 652	3.864 089	−2.293 875
H	−6.976 466	1.860 108	−2.214 394
H	−6.783 426	2.385 299	−0.511 412
H	−7.365 083	3.545 142	−1.747 531
H	−3.807 197	0.671 662	3.813 477

H	−2.131 550	0.327 498	4.303 276
H	−2.837 832	1.960 272	4.582 045
H	−4.182 801	2.188 163	1.672 358
H	−3.294 313	3.477 506	2.541 972
H	−2.754 709	2.909 742	0.916 802

TS2b

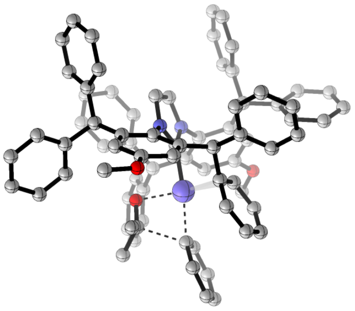

Rh	0.099 706	0.455 469	−1.651 597
C	−0.031 192	−0.125 130	0.247 843
N	−1.107 451	−0.776 159	0.767 828
C	−0.985 258	−0.955 234	2.140 133
C	0.192 757	−0.391 993	2.493 674
N	0.769 220	0.098 400	1.328 503
C	2.070 500	0.674 743	1.219 352
C	2.209 174	2.055 038	1.012 159
C	3.488 913	2.572 653	0.796 041
C	4.605 635	1.735 405	0.789 325
C	4.453 245	0.367 613	1.036 747
C	3.194 897	−0.175 375	1.263 491
C	3.021 822	−1.645 372	1.614 524
C	4.103 943	−2.553 092	1.059 443
C	5.328 223	−2.718 428	1.717 704
C	6.316 499	−3.542 267	1.183 533
C	6.090 079	−4.227 279	−0.010 032
C	4.866 023	−4.087 047	−0.660 501
C	3.882 781	−3.255 760	−0.128 583
H	2.924 811	−3.165 130	−0.643 964
H	4.663 965	−4.632 496	−1.585 414
H	6.863 019	−4.878 723	−0.424 805
H	7.267 315	−3.657 866	1.709 573
H	5.502 381	−2.207 677	2.668 172
C	2.853 767	−1.849 058	3.113 157
C	2.206 383	−3.004 083	3.567 366
C	2.017 351	−3.226 343	4.927 106
C	2.478 927	−2.296 164	5.860 520
C	3.134 366	−1.149 656	5.419 223
C	3.321 124	−0.927 620	4.053 465
H	3.822 807	−0.018 439	3.713 540
H	3.502 674	−0.416 638	6.141 298
H	2.326 756	−2.467 091	6.928 808
H	1.501 123	−4.129 035	5.262 810
H	1.838 267	−3.730 736	2.837 703
H	2.076 776	−1.975 222	1.155 289
H	5.342 953	−0.262 255	1.032 683
O	5.861 537	2.164 241	0.565 131
C	6.067 305	3.520 109	0.275 514
H	5.524 023	3.831 104	−0.633 832
H	5.755 820	4.171 402	1.110 887
H	7.144 510	3.645 152	0.108 747
H	3.593 504	3.641 446	0.620 787
C	0.987 909	2.962 318	0.973 076
C	1.335 550	4.424 449	0.750 894
C	1.808 326	5.236 730	1.788 876
C	2.158 860	6.563 895	1.553 170
C	2.035 913	7.107 759	0.274 169
C	1.554 342	6.313 316	−0.763 677
C	1.206 719	4.984 658	−0.522 853
H	0.828 150	4.365 731	−1.337 362
H	1.443 378	6.726 931	−1.769 122
H	2.307 338	8.150 186	0.090 836
H	2.524 913	7.181 261	2.377 369
H	1.897 007	4.826 523	2.797 197
C	0.074 229	2.813 492	2.173 863
C	0.559 505	2.616 515	3.470 105
C	−0.317 212	2.533 551	4.551 340
C	−1.691 493	2.649 829	4.349 992
C	−2.182 663	2.849 682	3.059 485
C	−1.305 874	2.926 142	1.980 724
H	−1.683 565	3.066 149	0.965 187
H	−3.257 311	2.949 862	2.884 037
H	−2.378 227	2.583 073	5.197 389
H	0.076 890	2.371 175	5.557 767
H	1.635 199	2.501 430	3.632 035
H	0.394 746	2.645 144	0.099 151
H	0.666 372	−0.289 389	3.462 735
H	−1.744 420	−1.454 142	2.734 675
C	−2.095 840	−1.343 651	−0.077 743
C	−1.672 615	−2.235 494	−1.086 595
C	−2.630 885	−2.773 227	−1.936 413
C	−3.980 328	−2.415 813	−1.818 672
C	−4.379 878	−1.530 410	−0.816 821
C	−3.439 699	−0.994 282	0.069 020
C	−3.879 493	−0.071 492	1.188 212
C	−4.305 229	−0.827 108	2.437 372
C	−4.566 184	−2.200 114	2.443 209
C	−4.939 241	−2.851 172	3.621 046
C	−5.055 266	−2.139 364	4.811 323
C	−4.795 425	−0.767 765	4.817 213
C	−4.424 985	−0.121 805	3.642 773
H	−4.216 647	0.949 731	3.653 925
H	−4.877 672	−0.198 206	5.746 214
H	−5.343 720	−2.649 470	5.733 411
H	−5.136 802	−3.925 829	3.603 505
H	−4.469 818	−2.770 720	1.517 193
C	−4.915 551	0.928 752	0.709 005
C	−6.279 210	0.793 369	0.980 461
C	−7.195 814	1.719 501	0.479 879
C	−6.758 191	2.794 129	−0.290 885
C	−5.396 223	2.938 327	−0.560 699
C	−4.482 486	2.011 649	−0.066 616
H	−3.415 886	2.107 989	−0.292 304
H	−5.042 304	3.782 223	−1.158 761
H	−7.475 685	3.522 338	−0.676 598
H	−8.259 648	1.601 644	0.700 776
H	−6.625 901	−0.039 182	1.597 767
H	−2.998 024	0.524 387	1.472 414
H	−5.421 657	−1.228 816	−0.711 549
O	−4.821 850	−2.973 430	−2.711 275
C	−6.176 679	−2.622 048	−2.664 963
H	−6.324 442	−1.537 877	−2.813 090

H	−6.670 673	−3.164 604	−3.480 794
H	−6.644 354	−2.913 406	−1.707 743
H	−2.352 384	−3.490 497	−2.709 807
C	−0.199 223	−2.538 892	−1.264 323
C	0.442 951	−1.574 195	−2.267 527
C	1.705 222	−0.949 136	−1.970 448
C	2.619 671	−0.649 488	−3.031 434
C	2.297 625	−0.914 129	−4.334 645
C	1.033 217	−1.487 137	−4.645 147
C	0.136 671	−1.794 849	−3.658 402
H	−0.810 678	−2.273 448	−3.916 531
H	0.777 154	−1.685 700	−5.688 833
H	2.997 848	−0.677 419	−5.138 196
H	3.594 753	−0.229 007	−2.773 062
H	2.146 259	−1.067 082	−0.979 691
C	0.114 904	−4.004 113	−1.527 990
C	1.174 746	−4.391 483	−2.356 495
C	1.519 266	−5.736 436	−2.492 006
C	0.813 229	−6.719 451	−1.803 449
C	−0.241 708	−6.345 285	−0.971 795
C	−0.583 704	−5.002 482	−0.835 657
H	−1.409 911	−4.719 639	−0.178 454
H	−0.803 160	−7.105 201	−0.422 456
H	1.082 959	−7.772 549	−1.912 690
H	2.348 534	−6.014 606	−3.147 422
H	1.739 962	−3.635 884	−2.904 887
H	0.287 275	−2.327 340	−0.299 776
O	−1.279 481	1.978 163	−1.272 094
C	−1.570 329	2.086 783	−2.517 477
C	−2.609 839	1.147 757	−3.086 859
C	−1.598 639	3.483 957	−3.091 387
C	0.310 771	1.536 650	−3.525 532
C	1.372 662	2.419 209	−3.260 337
C	2.149 926	2.968 459	−4.278 640
C	1.840 651	2.689 512	−5.610 814
C	0.767 496	1.848 253	−5.903 660
C	0.023 250	1.277 107	−4.870 955
H	−0.796 511	0.602 631	−5.130 111
H	0.513 206	1.629 493	−6.944 835
H	2.429 489	3.132 810	−6.418 134
H	2.993 264	3.620 139	−4.032 954
H	1.630 238	2.653 648	−2.221 111
H	−0.773 999	4.090 534	−2.703 471
H	−1.571 418	3.484 710	−4.187 898
H	−2.548 974	3.941 029	−2.759 862
H	−2.502 003	0.140 297	−2.667 026
H	−3.600 627	1.542 054	−2.799 047
H	−2.575 808	1.101 974	−4.181 812

TS 3

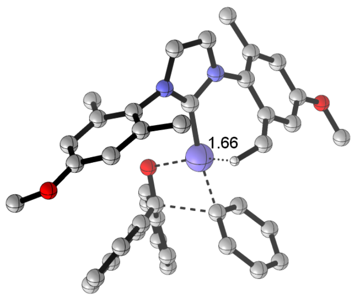

Rh	0.365 064	−0.298 603	−0.332 398
C	0.130 053	−2.206 897	0.165 213
N	1.195 302	−3.059 851	0.214 340
C	0.779 167	−4.372 932	0.362 515
C	−0.577 067	−4.342 743	0.421 526
N	−0.953 266	−3.014 111	0.296 814
C	−2.290 040	−2.523 569	0.215 936
C	−2.966 836	−2.618 987	−1.012 442
C	−4.240 522	−2.073 298	−1.107 151
C	−4.817 965	−1.409 174	−0.017 879
C	−4.132 389	−1.334 799	1.195 058
C	−2.857 901	−1.895 735	1.327 831
C	−2.097 515	−1.766 367	2.610 963
H	−1.547 539	−2.687 501	2.852 873
H	−4.564 181	−0.817 478	2.051 587
O	−6.024 832	−0.852 891	−0.240 064
C	−6.579 214	−0.034 216	0.753 359
H	−7.518 088	0.361 470	0.345 339
H	−6.804 380	−0.599 699	1.674 733
H	−5.910 583	0.808 677	1.000 107
H	−4.799 522	−2.114 526	−2.044 118
C	−2.300 011	−3.237 481	−2.204 827
H	−2.021 314	−4.287 343	−2.025 844
H	−1.304 496	−5.140 040	0.543 722
H	1.469 613	−5.208 668	0.403 500
C	2.512 292	−2.536 079	0.104 191
C	3.503 021	−2.857 960	1.044 874
C	4.733 033	−2.208 540	0.964 110
C	4.967 727	−1.226 611	−0.003 477
C	3.966 313	−0.899 663	−0.917 930
C	2.722 263	−1.542 931	−0.875 268
C	1.716 809	−1.236 144	−1.947 940
H	1.470 214	−2.165 195	−2.492 181
H	4.122 606	−0.131 549	−1.674 277
O	6.185 276	−0.647 059	0.020 121
C	6.463 812	0.368 422	−0.902 221
H	7.483 487	0.714 914	−0.691 115
H	6.419 658	0.002 534	−1.943 371
H	5.767 146	1.218 739	−0.799 844
H	5.526 577	−2.428 932	1.680 242
C	3.257 931	−3.840 803	2.154 085
H	2.251 291	−3.723 563	2.582 014
O	−0.502 356	0.611 538	1.237 381
C	−0.557 929	1.762 048	0.646 044
C	−1.871 999	2.084 009	−0.026 630
C	−2.596 900	3.234 193	0.307 863
C	−3.867 805	3.455 200	−0.223 777
C	−4.432 609	2.532 230	−1.100 207
C	−3.724 251	1.374 592	−1.427 439
C	−2.462 578	1.151 243	−0.889 096
H	−1.897 399	0.238 583	−1.125 867
H	−4.162 559	0.635 825	−2.100 943
H	−5.422 967	2.709 690	−1.527 115
H	−4.417 525	4.358 186	0.053 516
H	−2.176 796	3.957 682	1.008 002
C	0.173 317	2.887 093	1.325 193
C	0.728 750	2.644 687	2.583 504
C	1.423 263	3.650 014	3.255 240
C	1.569 787	4.907 016	2.673 940
C	1.020 522	5.154 060	1.413 034
C	0.330 179	4.152 501	0.742 474
H	−0.057 262	4.334 239	−0.262 485
H	1.145 584	6.132 714	0.943 464
H	2.116 866	5.695 004	3.197 460
H	1.853 307	3.447 865	4.239 401
H	0.607 920	1.650 057	3.015 778
C	0.843 505	1.669 735	−1.000 756

C	2.160 022	1.986 491	−0.620 714
C	2.978 769	2.759 989	−1.437 871
C	2.481 686	3.272 165	−2.640 236
C	1.167 619	2.996 921	−3.016 440
C	0.353 961	2.209 489	−2.197 198
H	−0.678 578	2.013 672	−2.498 450
H	0.769 756	3.402 158	−3.950 950
H	3.114 541	3.898 785	−3.274 067
H	4.001 896	2.990 128	−1.126 451
H	2.542 663	1.624 380	0.338 632
H	0.554 023	−0.871 137	−1.874 244
H	−1.363 760	−0.950 118	2.512 322
H	−2.771 283	−1.530 582	3.445 816
H	3.991 409	−3.698 084	2.958 751
H	3.349 365	−4.884 579	1.812 149
H	2.136 599	−0.517 875	−2.663 726
H	−1.369 695	−2.696 929	−2.443 607
H	−2.955 051	−3.202 353	−3.085 126

TS 4

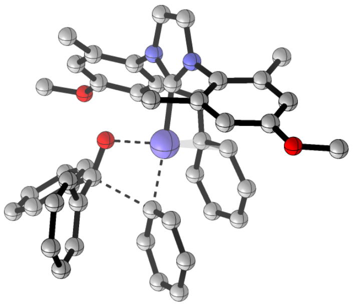

Rh	0.169 070	−0.237 106	0.316 477
C	0.467 323	−1.878 834	−0.753 765
N	1.606 788	−2.386 803	−1.305 449
C	1.330 767	−3.479 269	−2.113 506
C	−0.010 761	−3.672 993	−2.062 701
N	−0.516 561	−2.684 480	−1.233 604
C	−1.855 731	−2.572 454	−0.772 701
C	−2.885 676	−2.309 848	−1.679 690
C	−4.194 440	−2.215 664	−1.195 016
C	−4.457 299	−2.362 069	0.167 322
C	−3.404 729	−2.591 414	1.060 604
C	−2.092 029	−2.677 399	0.611 268
C	−0.951 820	−2.787 428	1.587 639
C	−0.370 463	−1.438 554	2.027 957
C	−1.152 327	−0.713 868	2.999 720
C	−0.571 727	0.127 553	3.908 000
C	0.835 572	0.333 675	3.919 069
C	1.620 337	−0.318 247	3.008 431
C	1.050 783	−1.204 643	2.037 736
H	1.721 971	−1.957 214	1.620 271
H	2.705 622	−0.187 874	3.018 069
H	1.278 911	1.016 232	4.646 647
H	−1.196 381	0.651 165	4.636 140
H	−2.232 192	−0.883 534	3.031 473
H	−0.143 365	−3.395 968	1.158 096
H	−3.638 784	−2.679 473	2.123 068
O	−5.685 394	−2.249 286	0.717 756
C	−6.768 192	−1.936 823	−0.114 338
H	−7.654 460	−1.881 938	0.530 446
H	−6.627 820	−0.963 256	−0.616 461
H	−6.933 448	−2.713 593	−0.881 593
H	−4.994 298	−1.985 187	−1.898 443
C	−2.587 823	−2.045 023	−3.125 943
H	−1.760 504	−1.323 721	−3.212 960
H	−0.641 005	−4.426 785	−2.524 781
H	2.111 777	−4.014 536	−2.645 574
C	2.910 362	−1.880 911	−1.021 224
C	3.713 976	−2.546 436	−0.090 189
C	4.958 966	−1.996 396	0.238 429
C	5.388 806	−0.811 644	−0.361 926
C	4.582 325	−0.185 076	−1.320 279
C	3.342 799	−0.707 360	−1.666 769
C	2.487 002	−0.047 673	−2.701 997
H	1.535 541	0.297 663	−2.267 884
H	4.951 918	0.729 100	−1.788 447
O	6.562 576	−0.208 562	−0.091 233
C	7.403 586	−0.769 444	0.876 541
H	8.281 259	−0.114 746	0.948 923
H	6.914 199	−0.820 871	1.865 217
H	7.738 408	−1.783 336	0.593 773
H	5.577 775	−2.509 098	0.975 200
C	3.283 234	−3.839 792	0.539 772
H	3.735 712	−3.963 725	1.533 174
O	−0.447 840	0.751 854	−1.396 303
C	−0.847 518	1.868 491	−0.913 447
C	0.168 969	1.796 216	1.074 717
C	−0.607 470	2.470 933	2.025 363
C	−0.060 979	3.471 664	2.829 464
C	1.284 422	3.817 592	2.693 348
C	2.075 400	3.173 453	1.739 261
C	1.507 114	2.201 012	0.920 263
H	2.121 955	1.739 863	0.137 039
H	3.126 989	3.447 973	1.620 197
H	1.713 896	4.603 804	3.320 291
H	−0.686 465	3.986 613	3.564 416
H	−1.663 687	2.212 887	2.140 442
C	−0.169 697	3.104 989	−1.427 658
C	0.498 885	3.023 201	−2.653 412
C	1.138 932	4.139 181	−3.189 447
C	1.120 404	5.351 219	−2.503 646
C	0.451 734	5.441 792	−1.280 696
C	−0.190 661	4.330 989	−0.748 057
H	−0.677 503	4.395 886	0.226 935
H	0.443 447	6.385 047	−0.729 347
H	1.627 813	6.225 928	−2.917 975
H	1.653 224	4.060 147	−4.150 590
H	0.499 706	2.069 814	−3.182 621
C	−2.285 336	1.985 120	−0.491 116
C	−3.060 221	3.097 025	−0.846 364
C	−4.411 453	3.160 525	−0.506 802
C	−5.008 381	2.115 986	0.194 000
C	−4.248 663	0.995 228	0.537 383
C	−2.904 515	0.930 093	0.191 402
H	−2.292 400	0.065 544	0.471 010
H	−4.704 844	0.169 644	1.087 929
H	−6.062 642	2.174 186	0.476 922
H	−4.998 296	4.036 484	−0.794 020
H	−2.609 705	3.916 486	−1.407 987
H	−3.466 782	−1.624 974	−3.632 560
H	3.595 588	−4.699 664	−0.075 958
H	2.192 164	−3.904 006	0.648 337
H	2.231 917	−0.752 732	−3.509 206

H	3.002 583	0.814 807	−3.144 058
H	−1.303 153	−3.321 330	2.485 606
H	−2.291 820	−2.955 059	−3.671 020

NMR Spectra

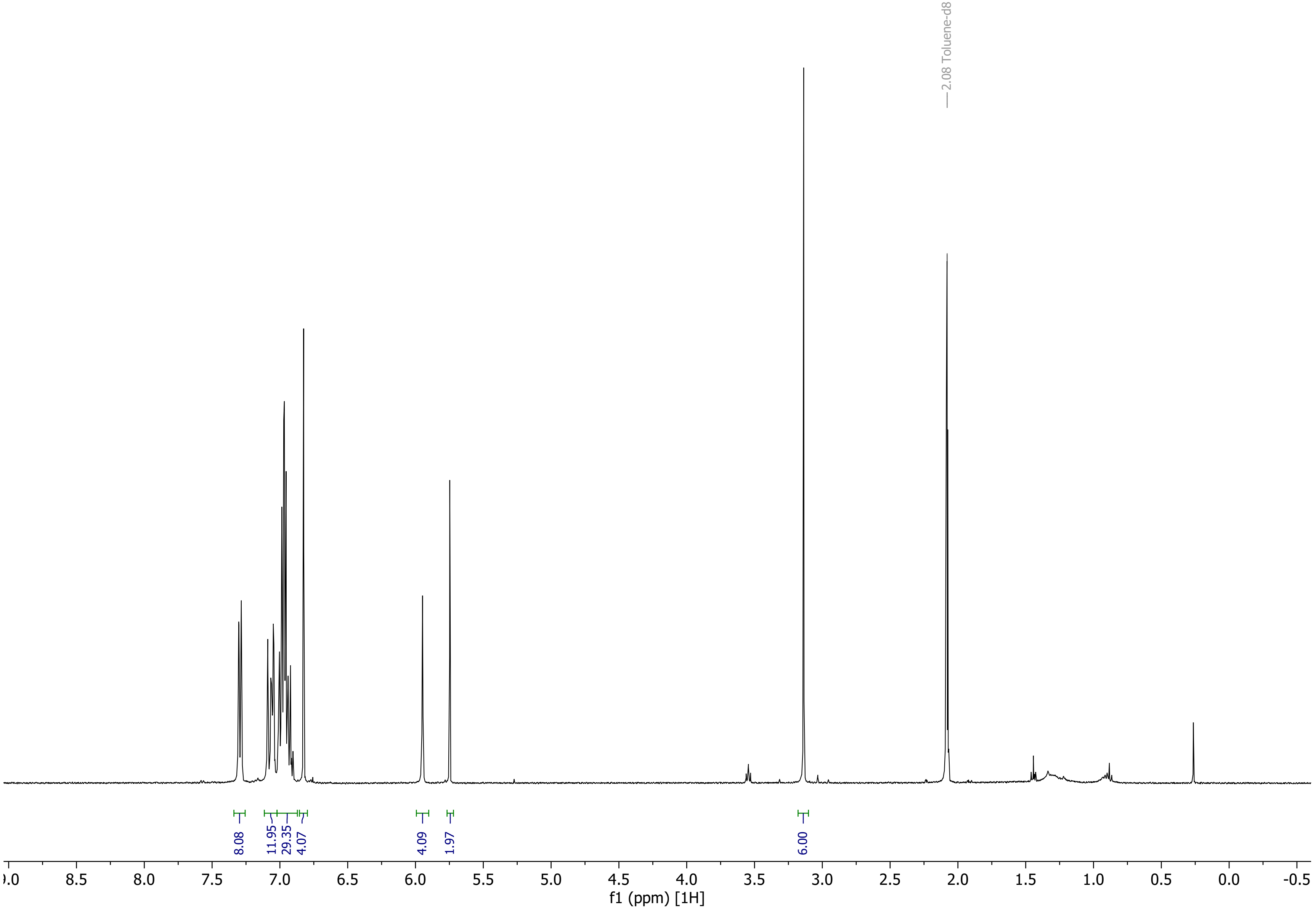

Figure S73: ^{1}H-NMR spectrum of **L1** (500 MHz, toluene-d_8).

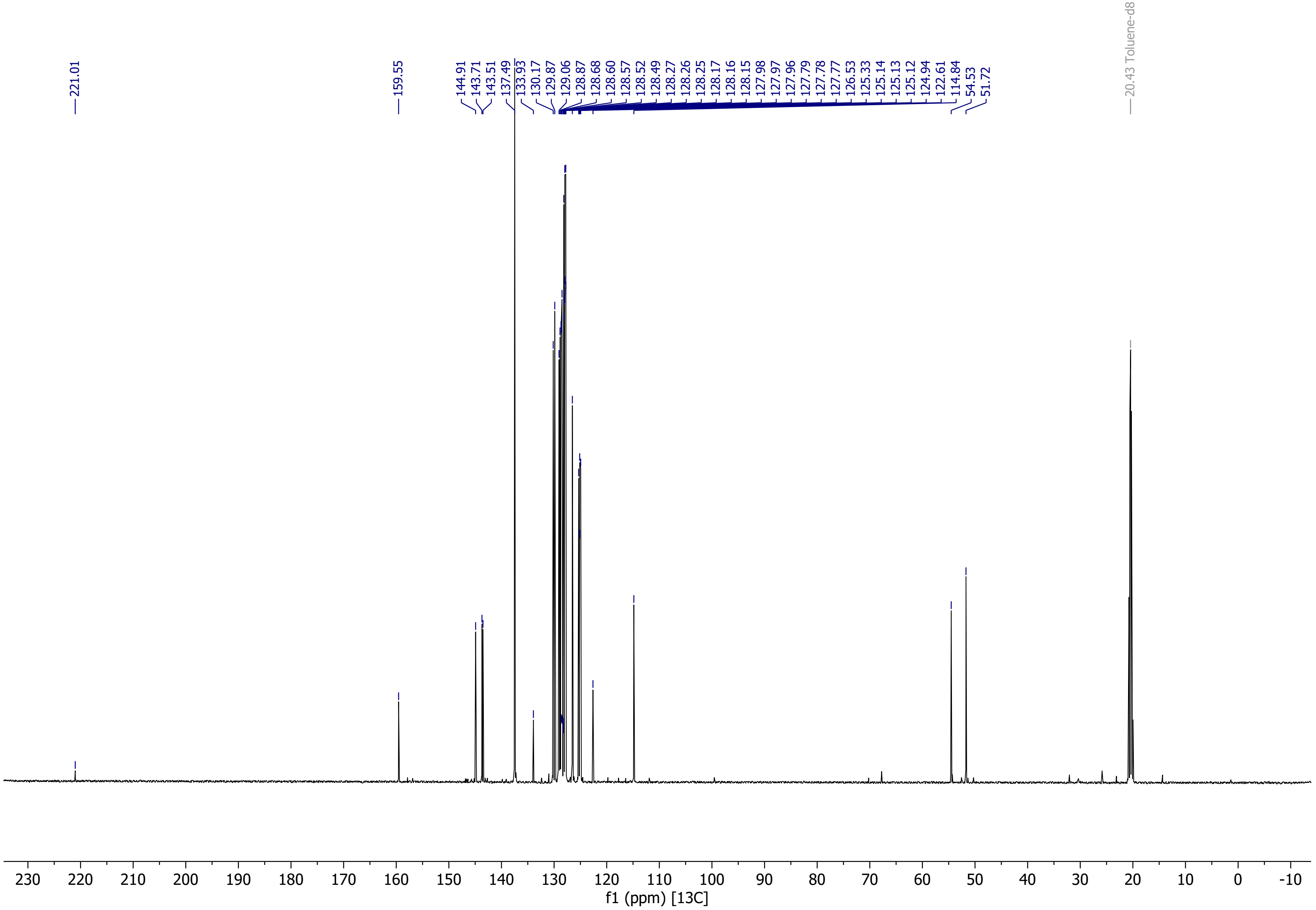

Figure S74: $^{13}C\{^{1}H\}$-NMR spectrum of **L1** (126 MHz, toluene-d_8).

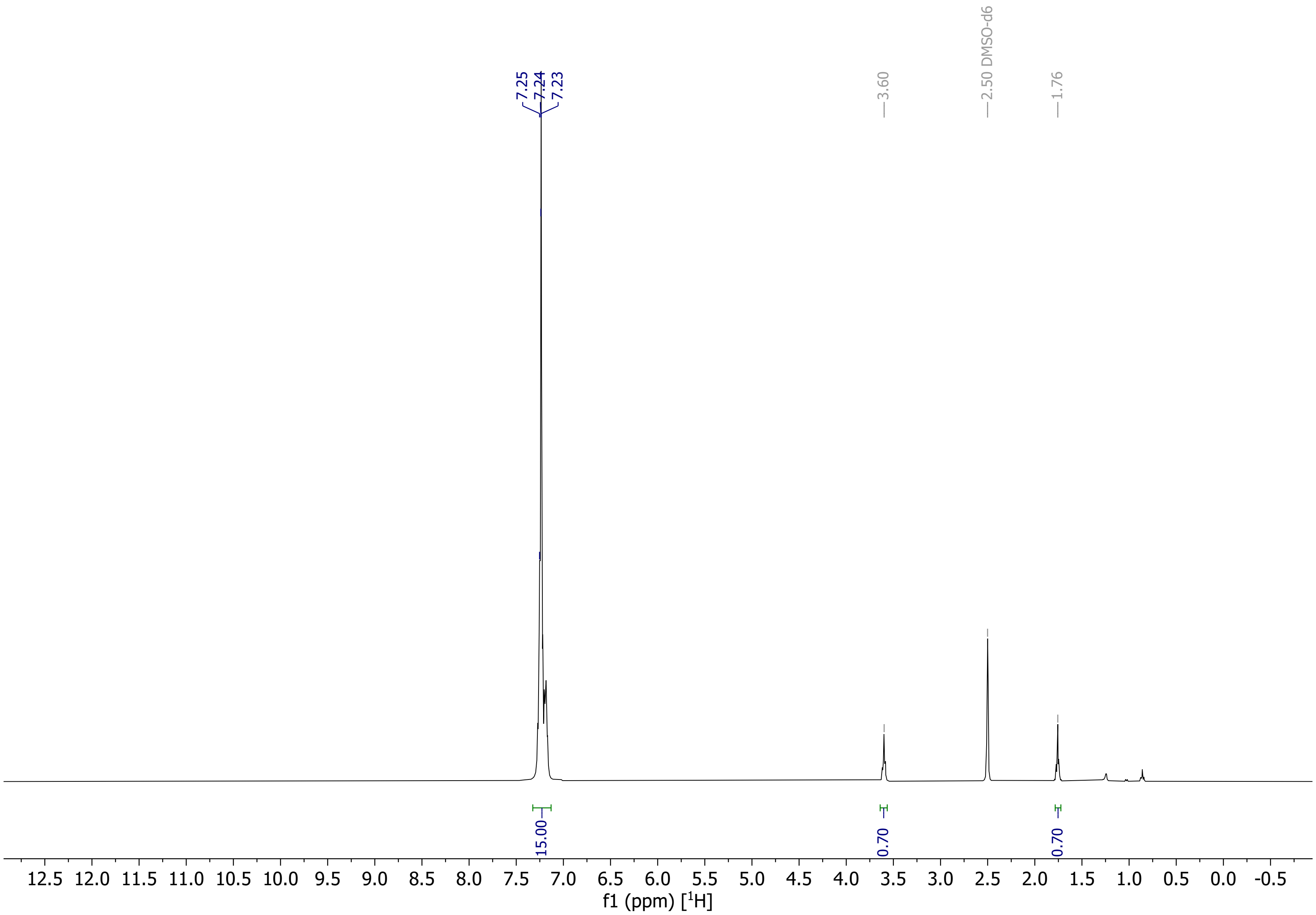

Figure S75: ^{1}H-NMR spectrum of Li-**5** (400 MHz, DMSO-d_6).

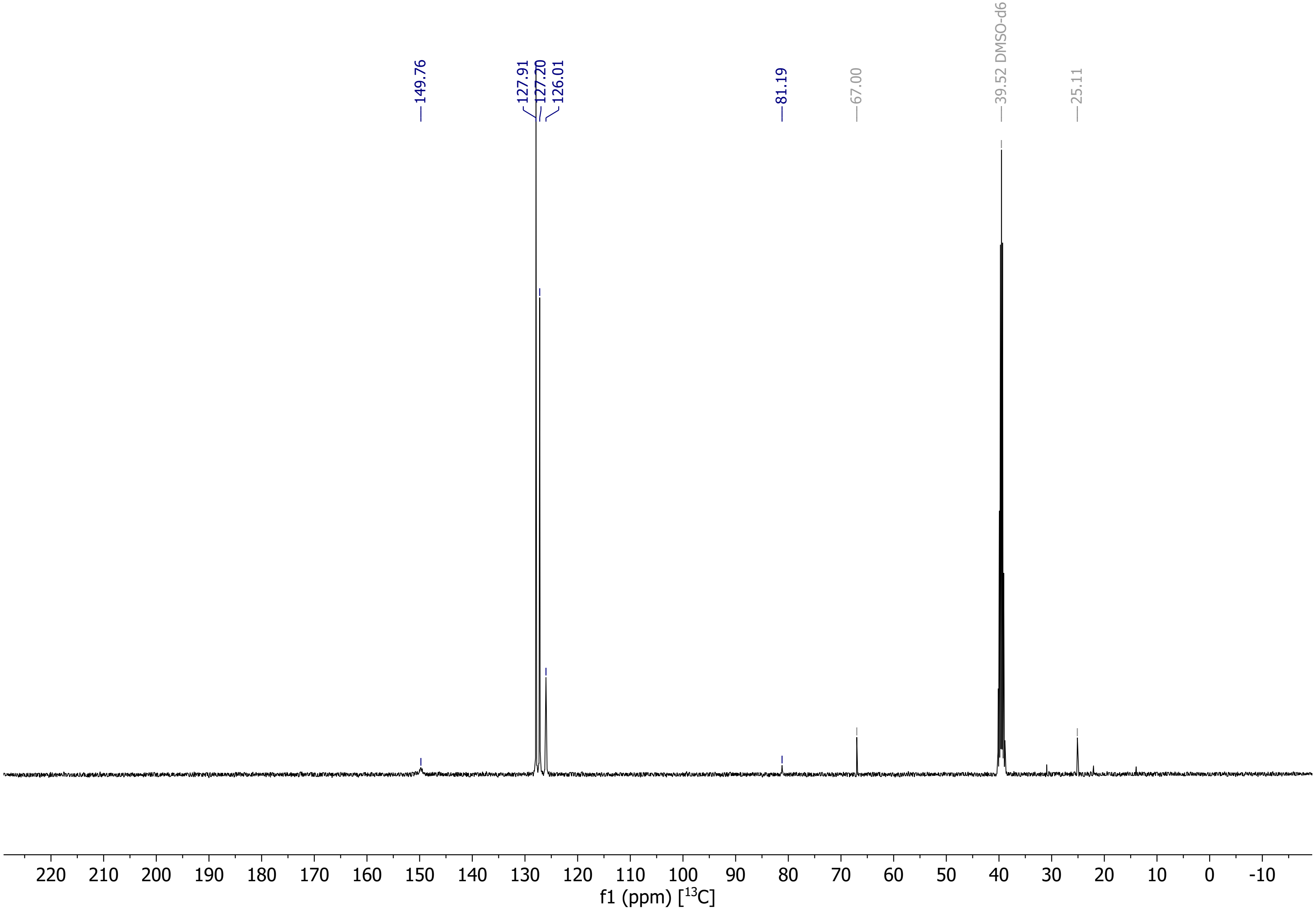

Figure S76: $^{13}C\{^1H\}$-NMR spectrum of Li-**5** (101 MHz, DMSO-d_6).

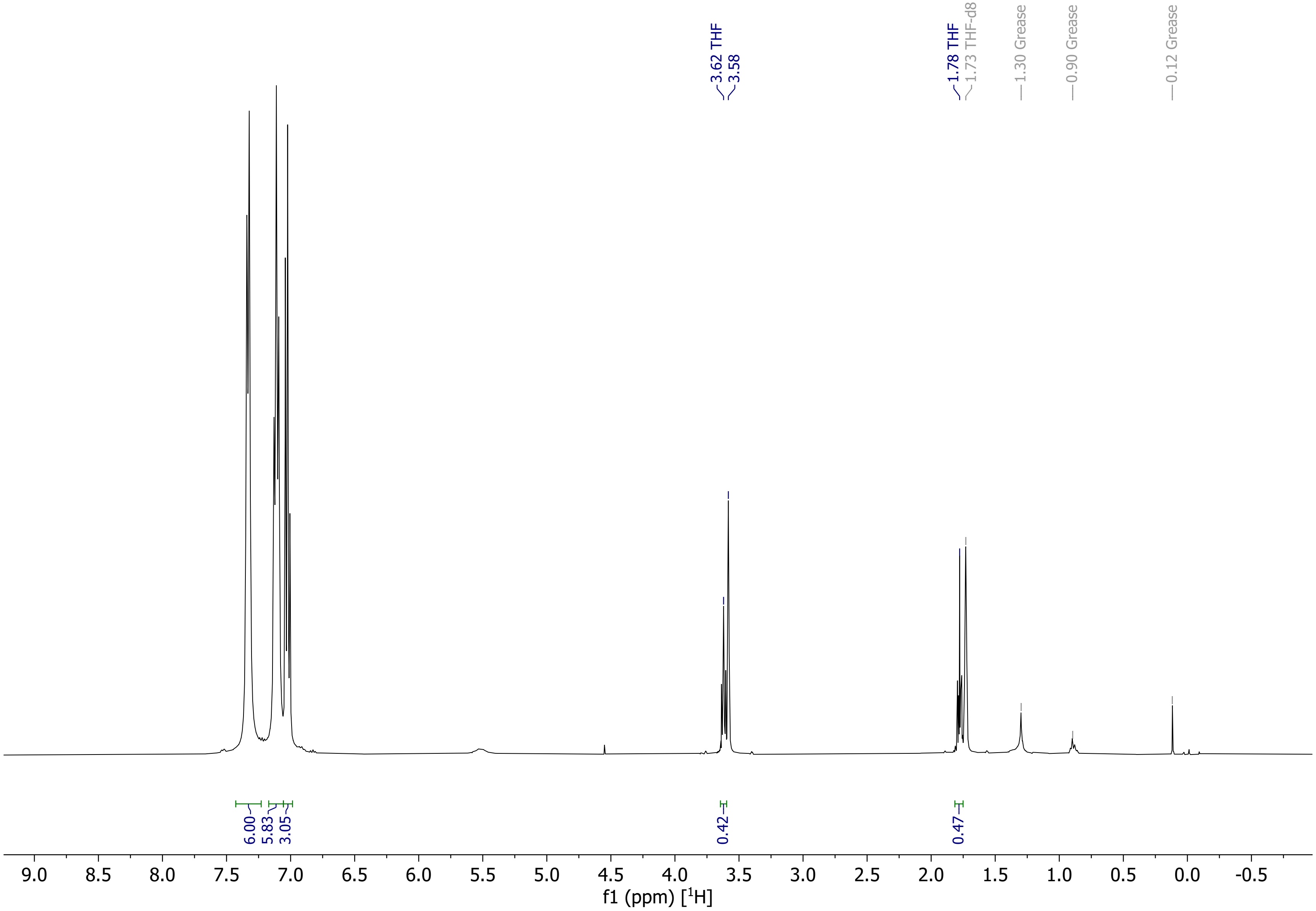

Figure S77: ^{1}H-NMR spectrum of Na-**5** (400 MHz, THF-d_8).

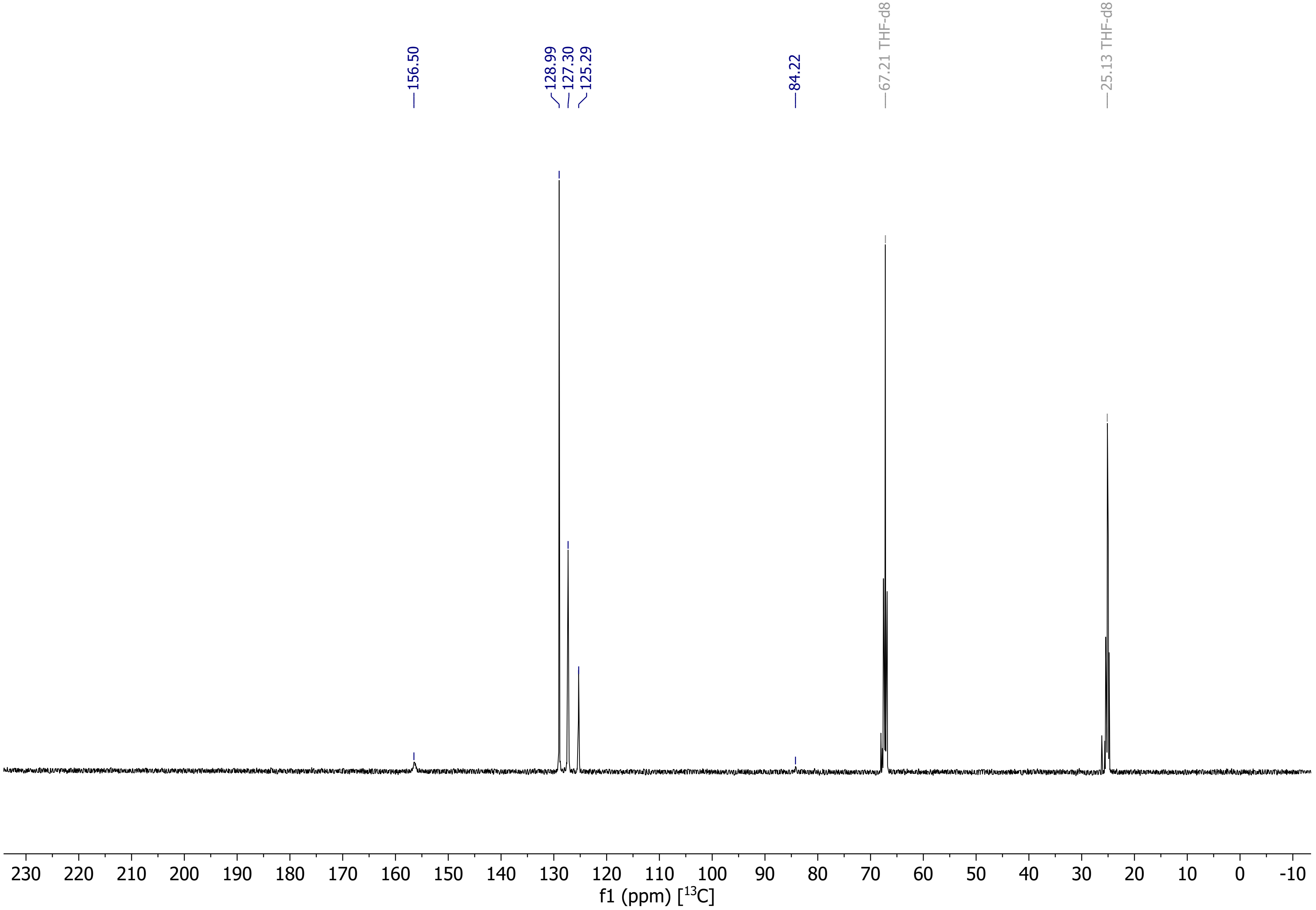

Figure S78: $^{13}C\{^{1}H\}$-NMR spectrum of Na-**5** (126 MHz, THF-d_8).

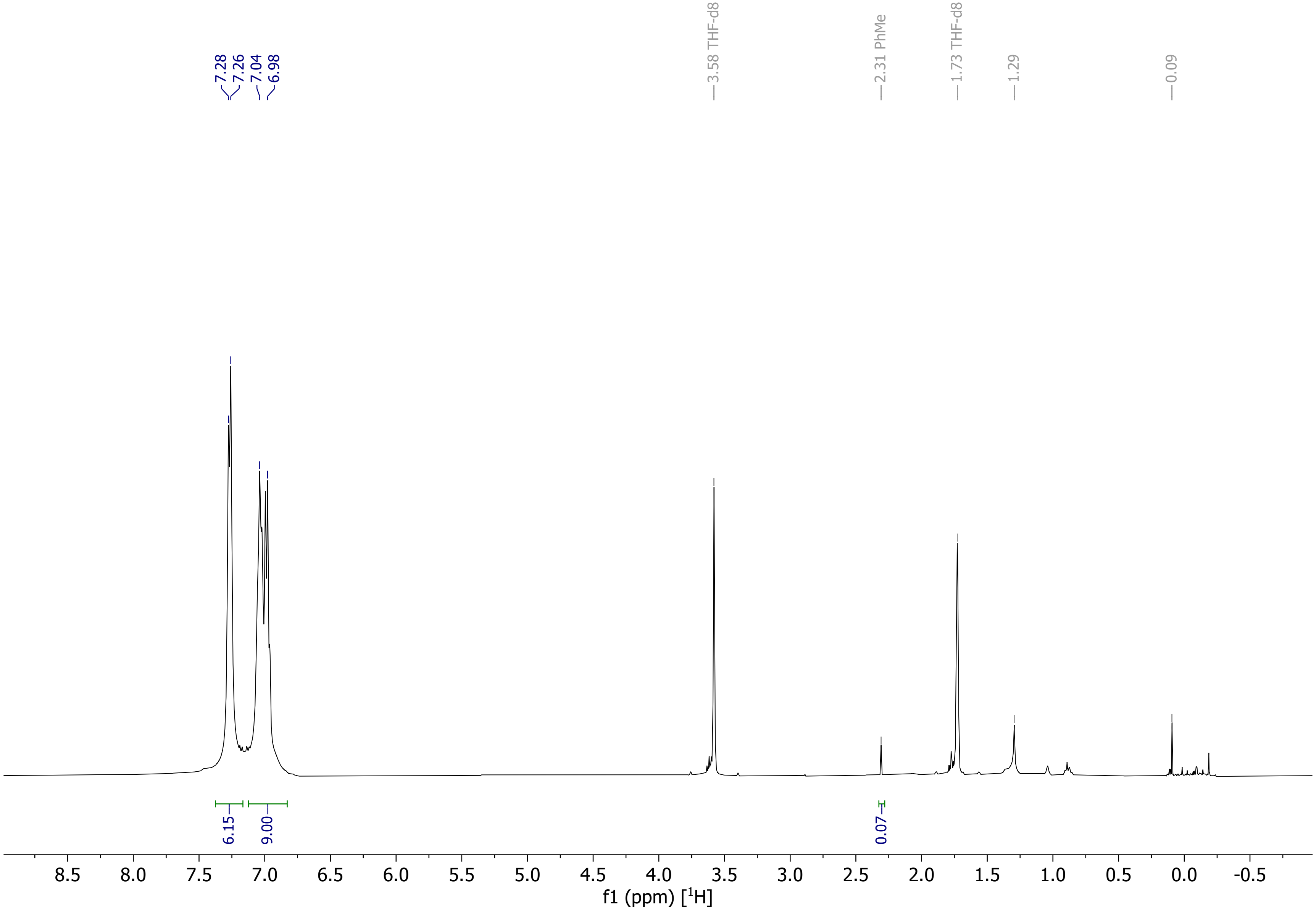

Figure S79: ^{1}H-NMR spectrum of K-**5** (400 MHz, THF-d_8).

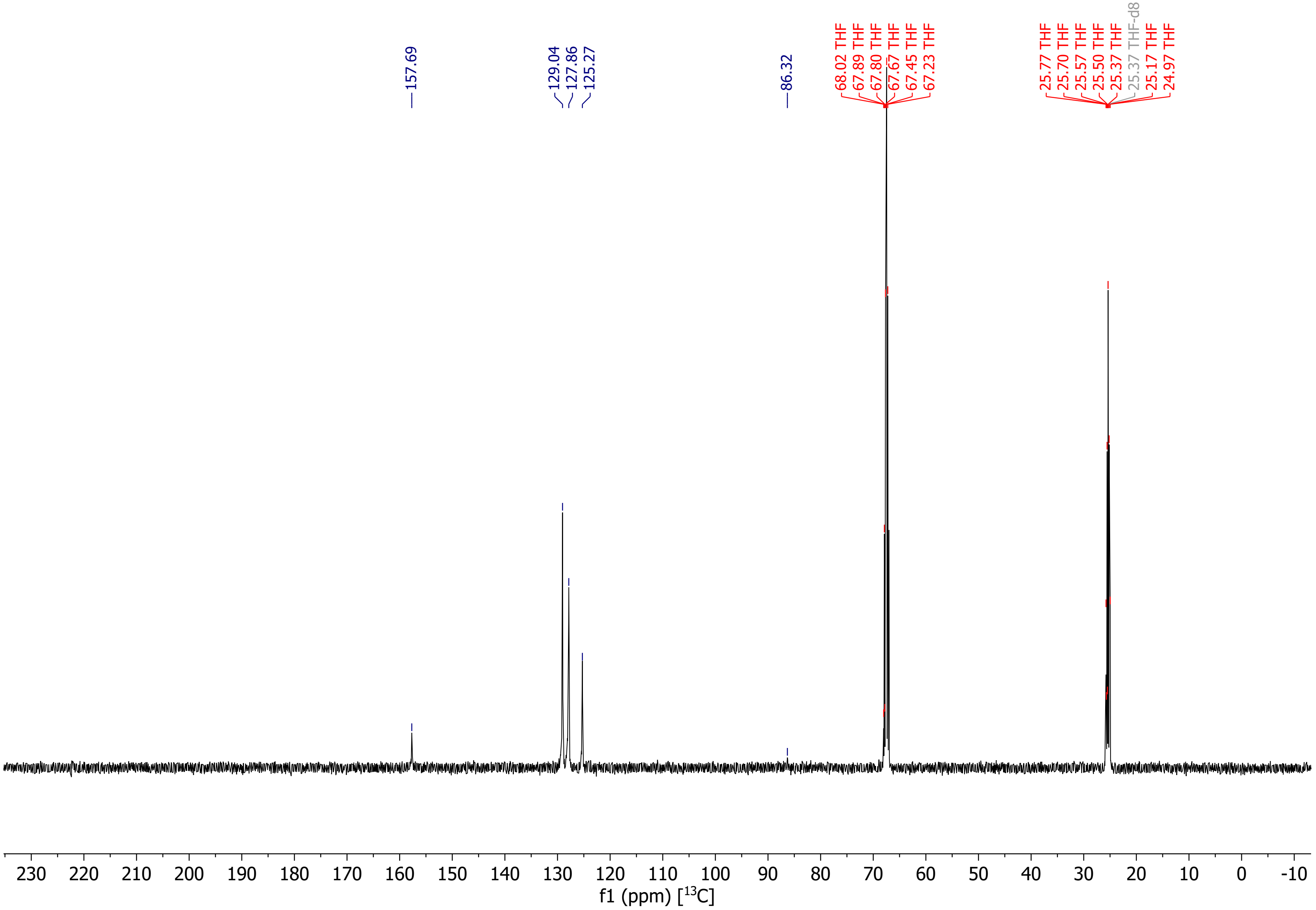

Figure S80: $^{13}C\{^{1}H\}$-NMR spectrum of K-**5** (101 MHz, THF-d_8).

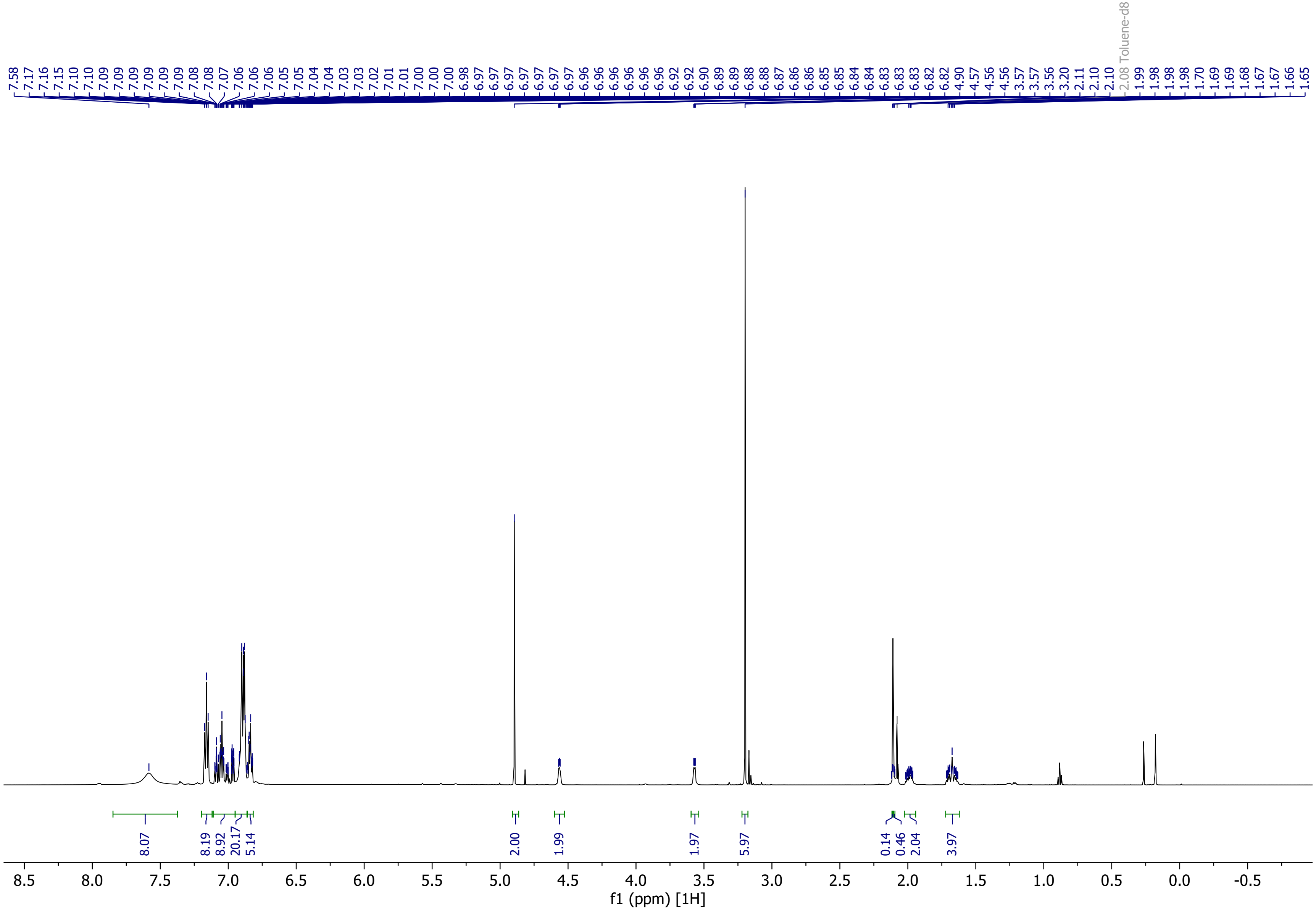

Figure S81: ^{1}H-NMR spectrum of **S1** (600 MHz, toluene-d_8).

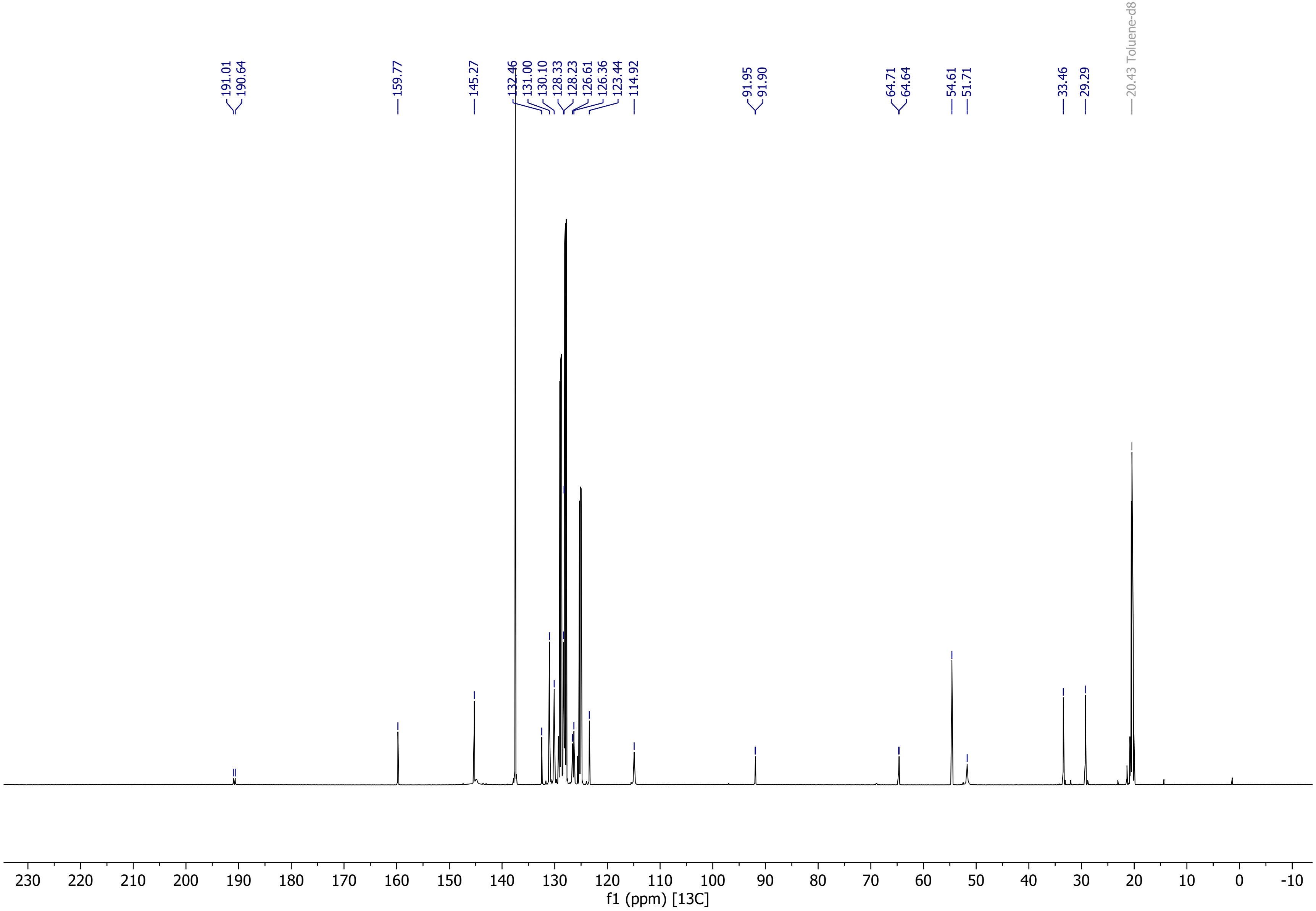

Figure S82: ${}^{13}C\{{}^{1}H\}$-NMR spectrum of **S1** (150 MHz, toluene-d_8).

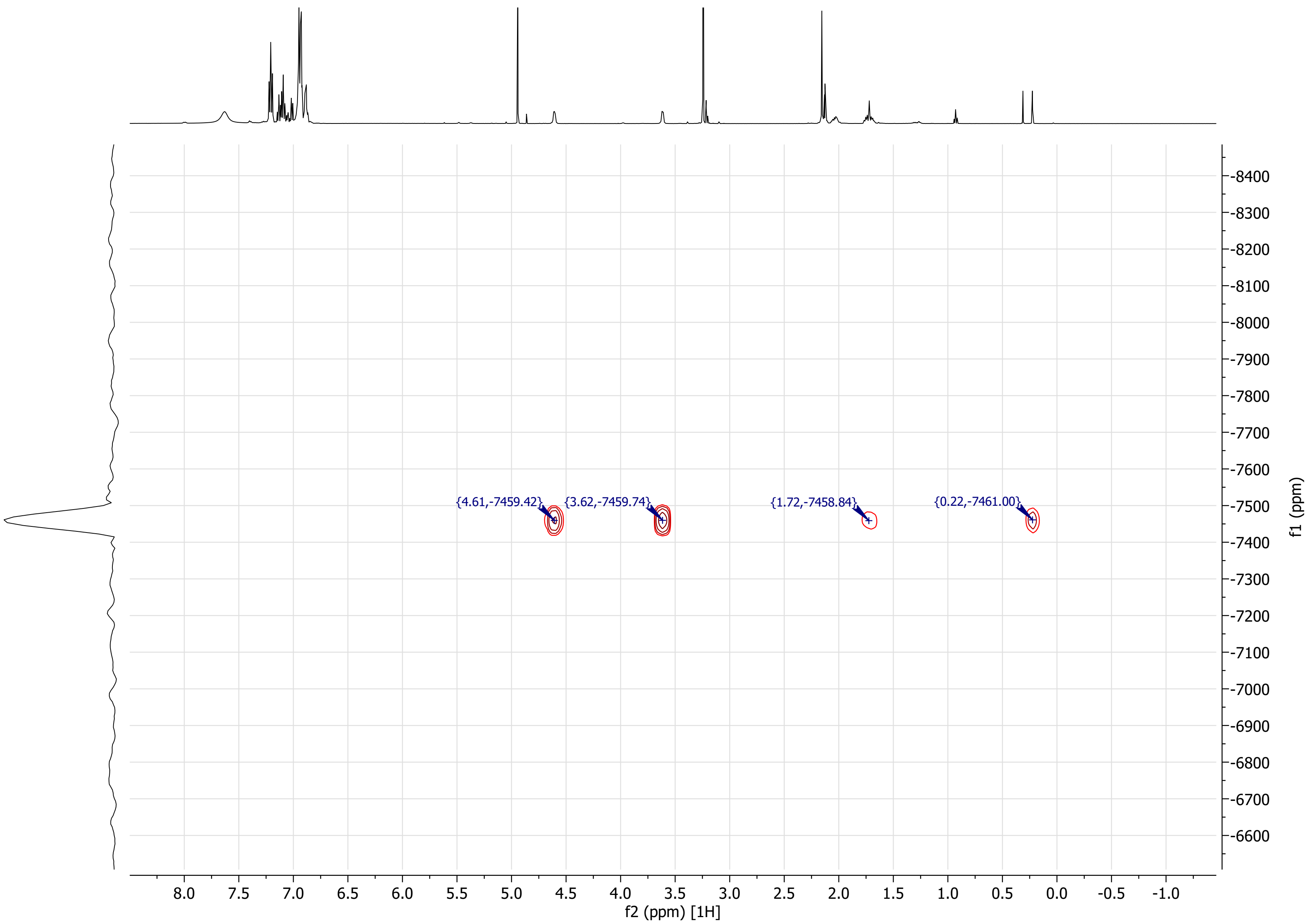

Figure S83: ${}^{1}H$-${}^{103}Rh$ HMBC spectrum of **S1** (16 MHz, toluene-d_8).

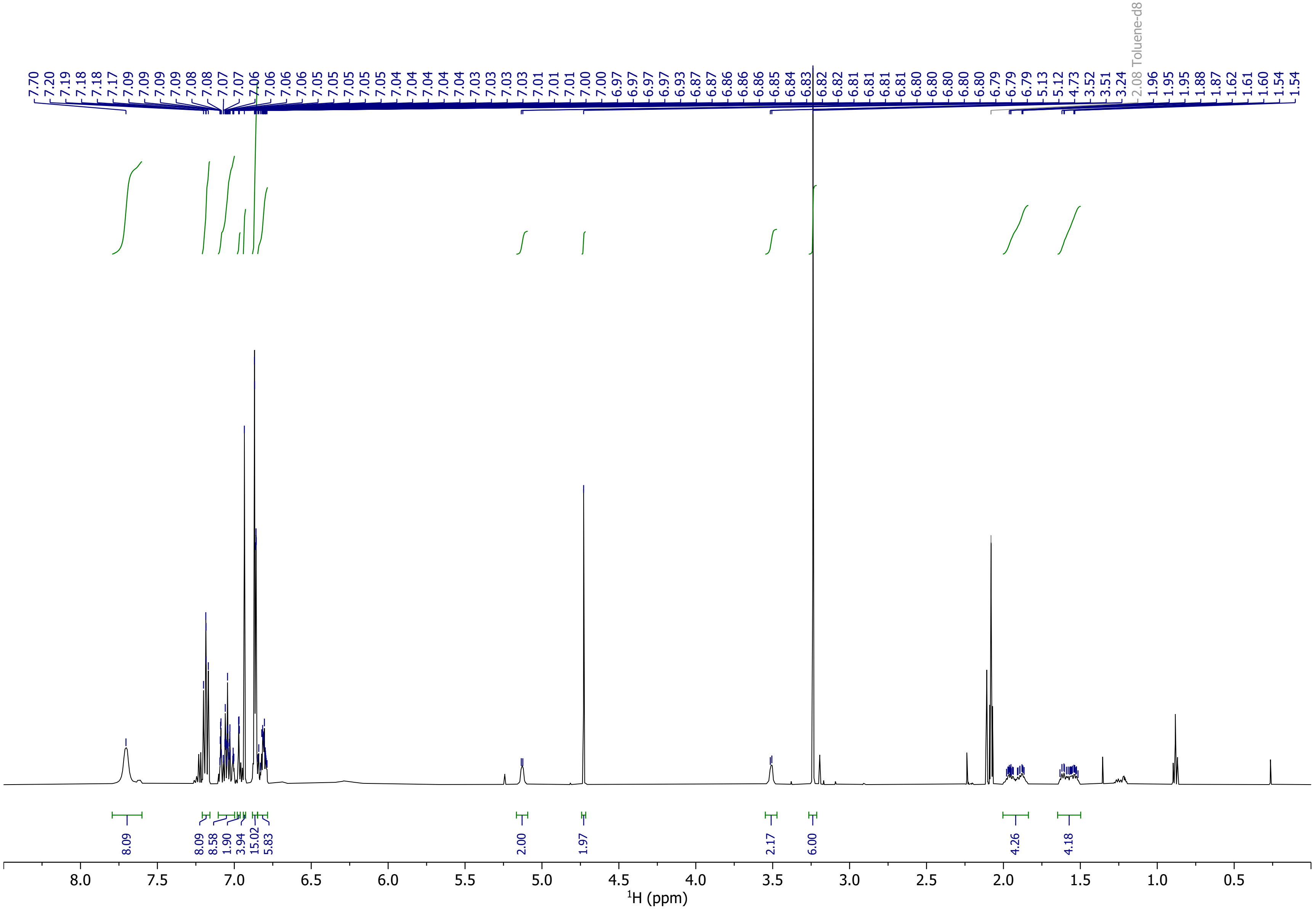

Figure S84: ^{1}H-NMR spectrum of **2** (500 MHz, toluene-d_8).

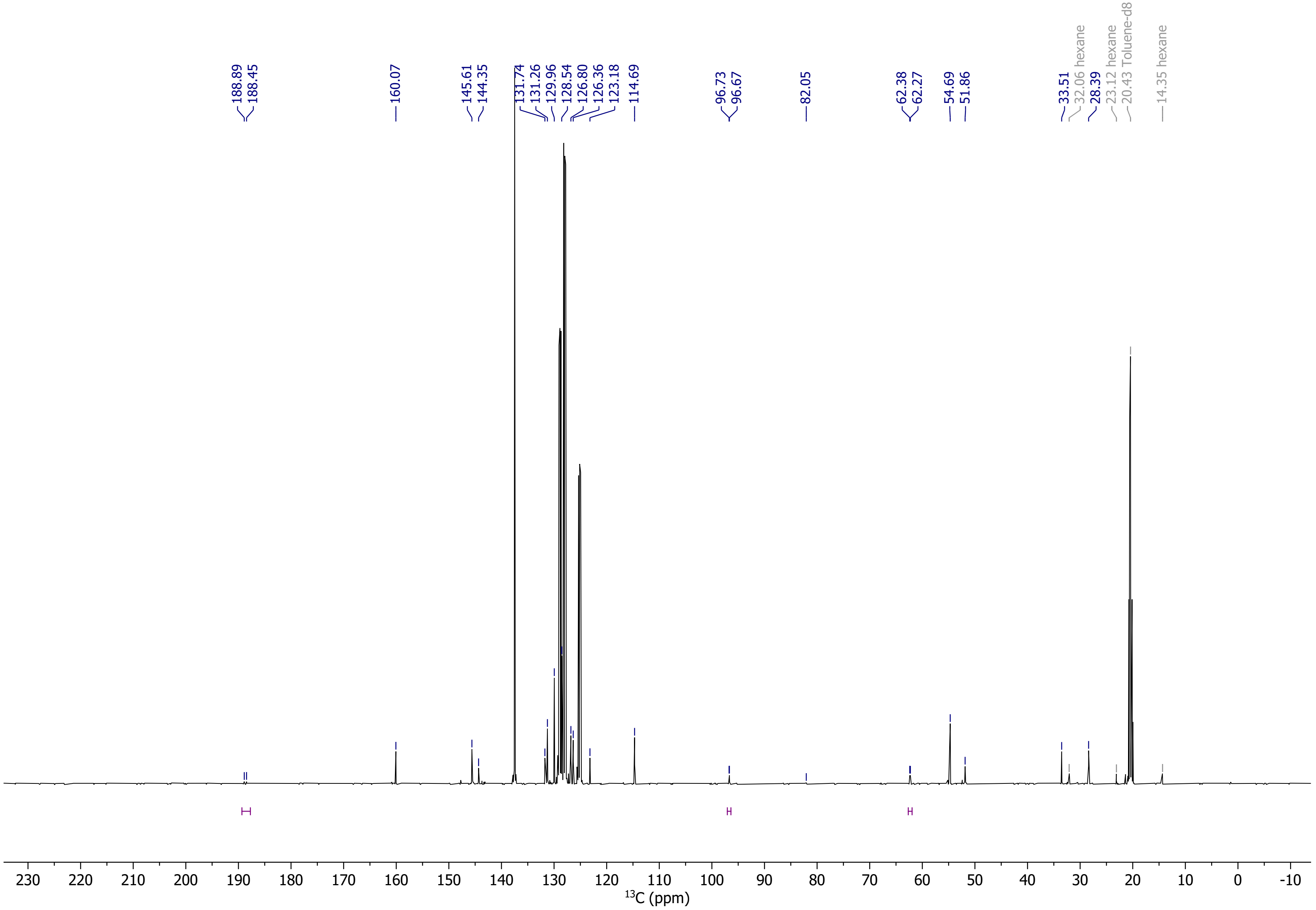

Figure S85: $^{13}C\{^{1}H\}$-NMR spectrum of **2** (126 MHz, toluene-d_8).

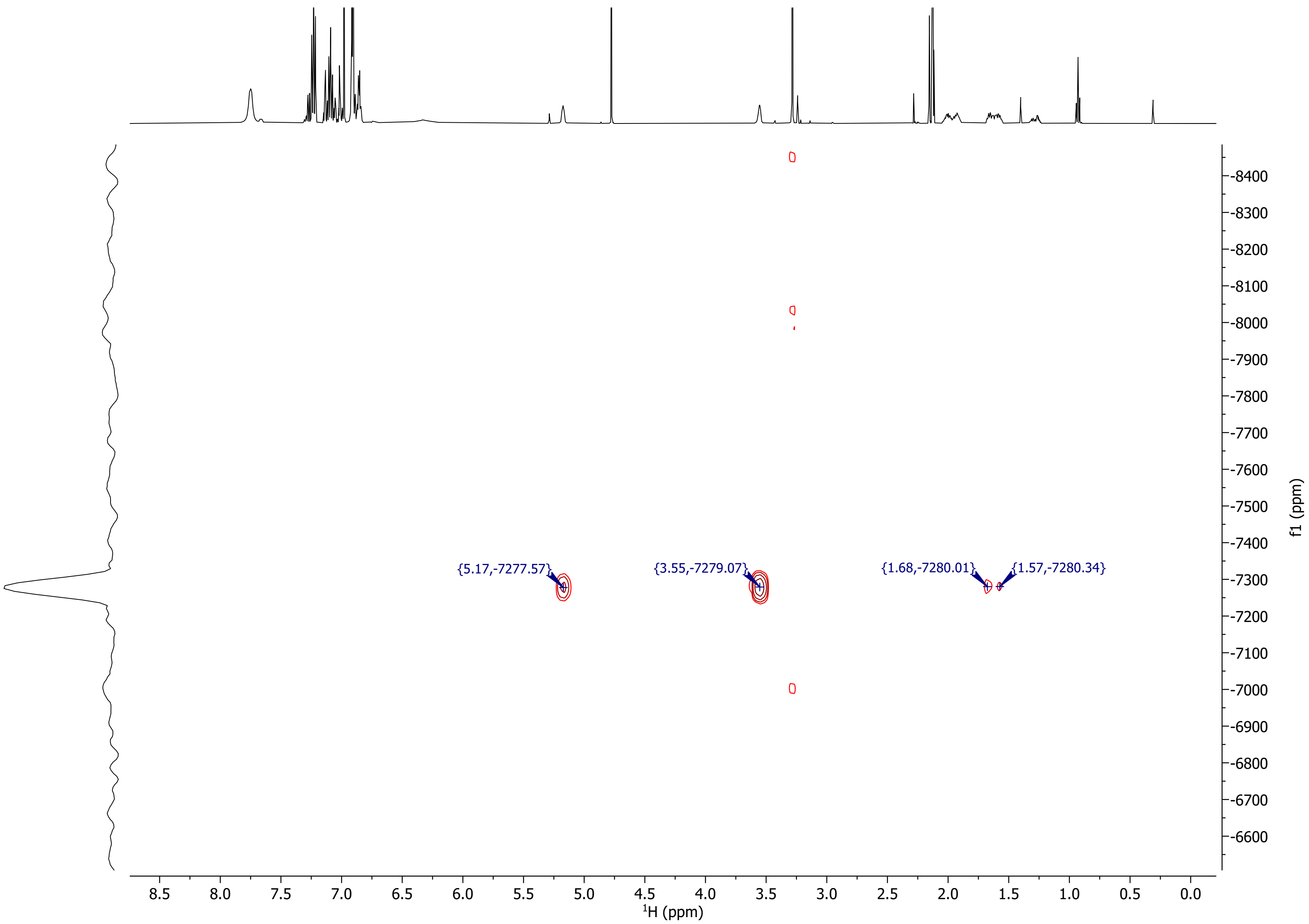

Figure S86: ^{1}H-^{103}Rh HMBC spectrum of **2** (16 MHz, toluene-d_8).

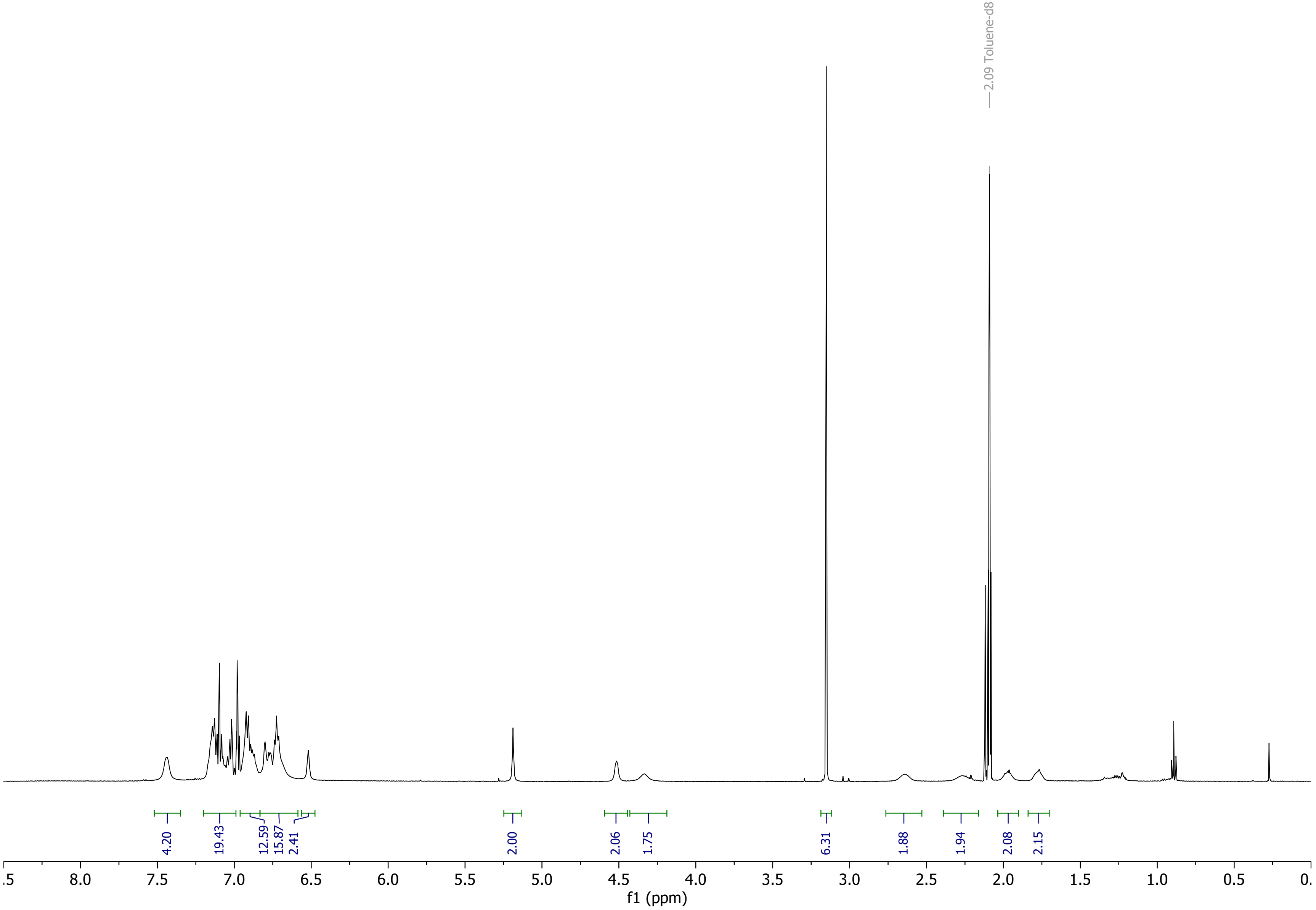

Figure S87: ^{1}H-NMR spectrum of **4** (600 MHz, toluene-d_8).

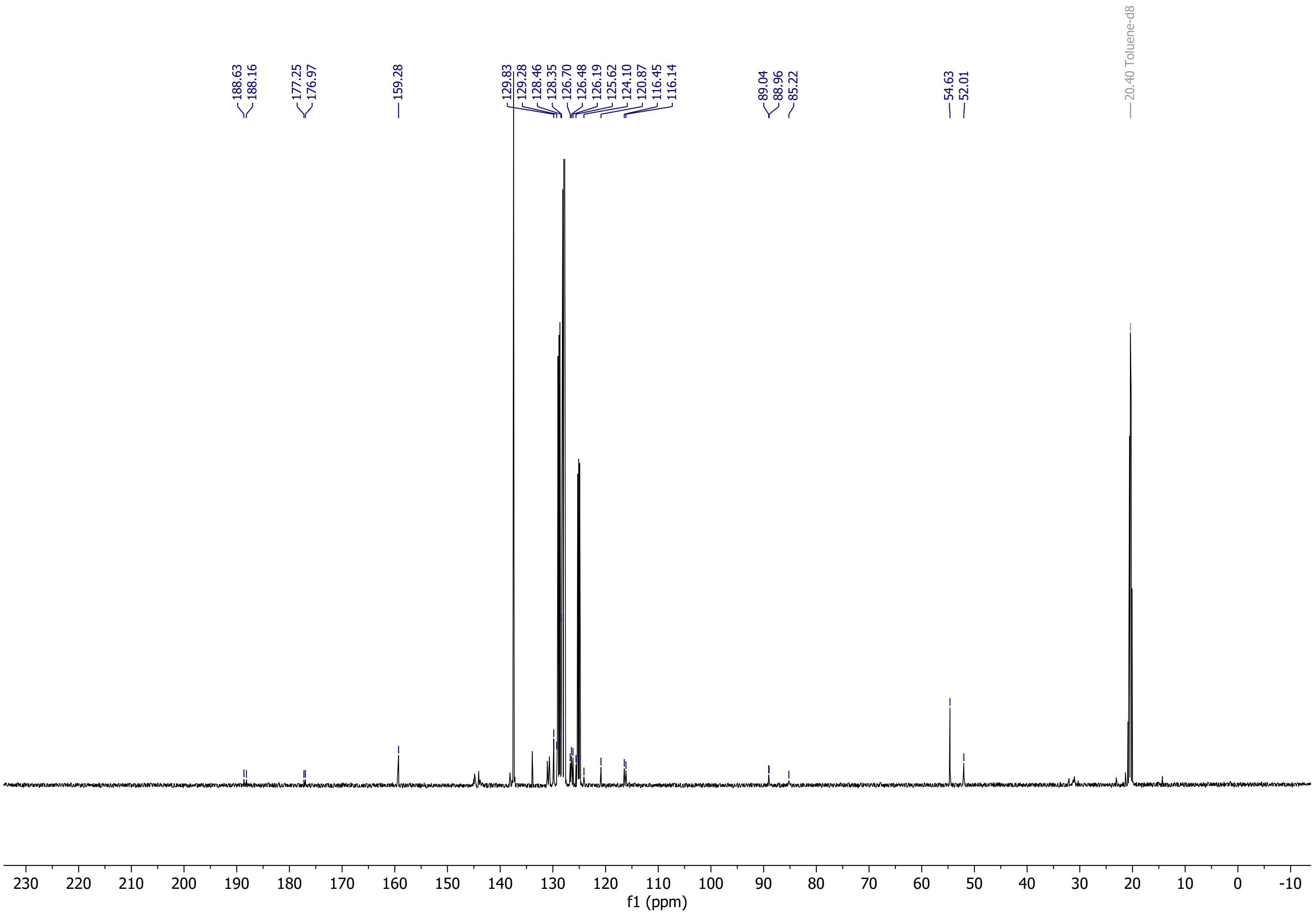

Figure S88: $^{13}C\{^1H\}$-NMR spectrum of **4** (151 MHz, toluene-d_8).

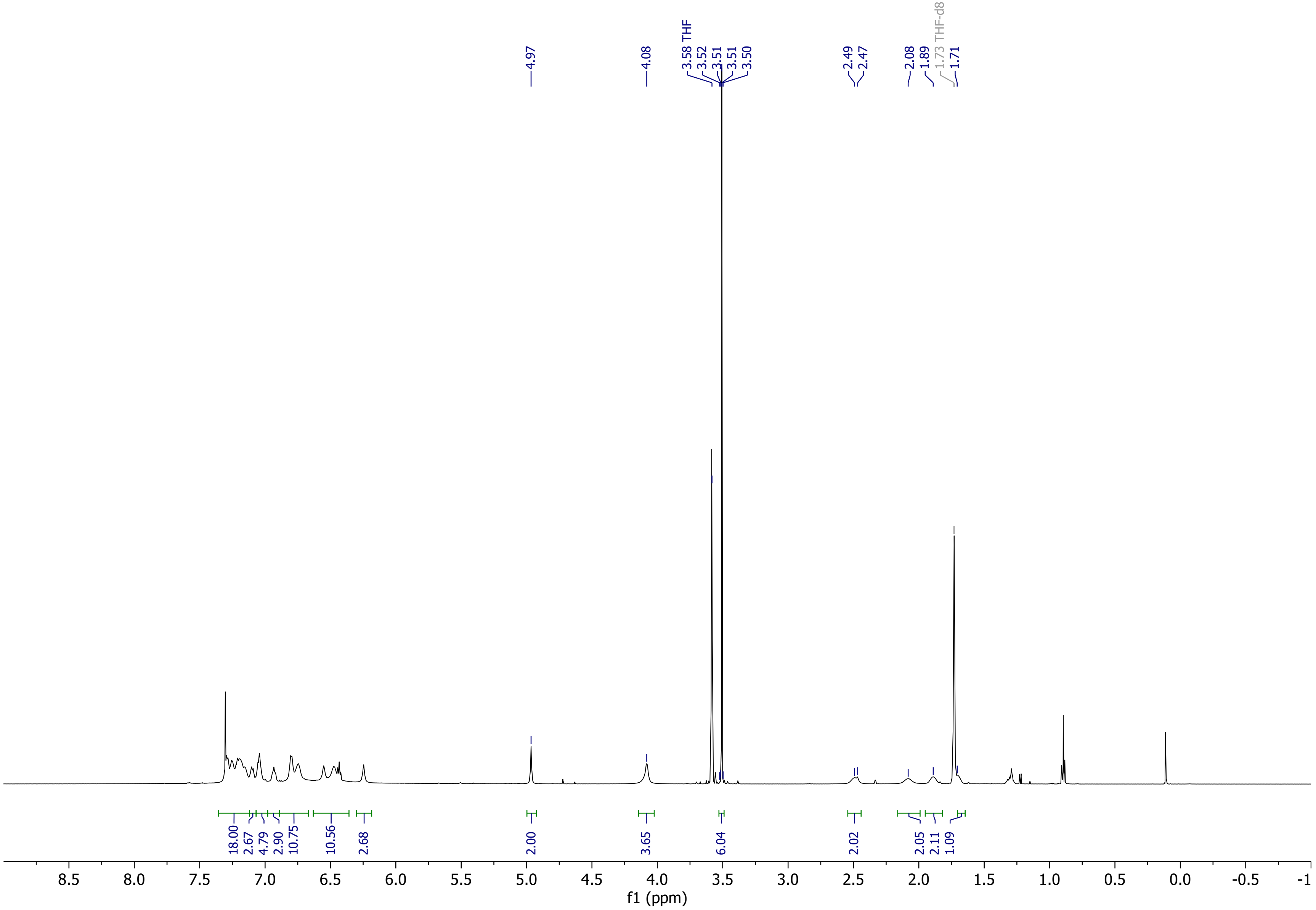

Figure S89: ^{1}H-NMR spectrum of **4** (500 MHz, THF-d_8).

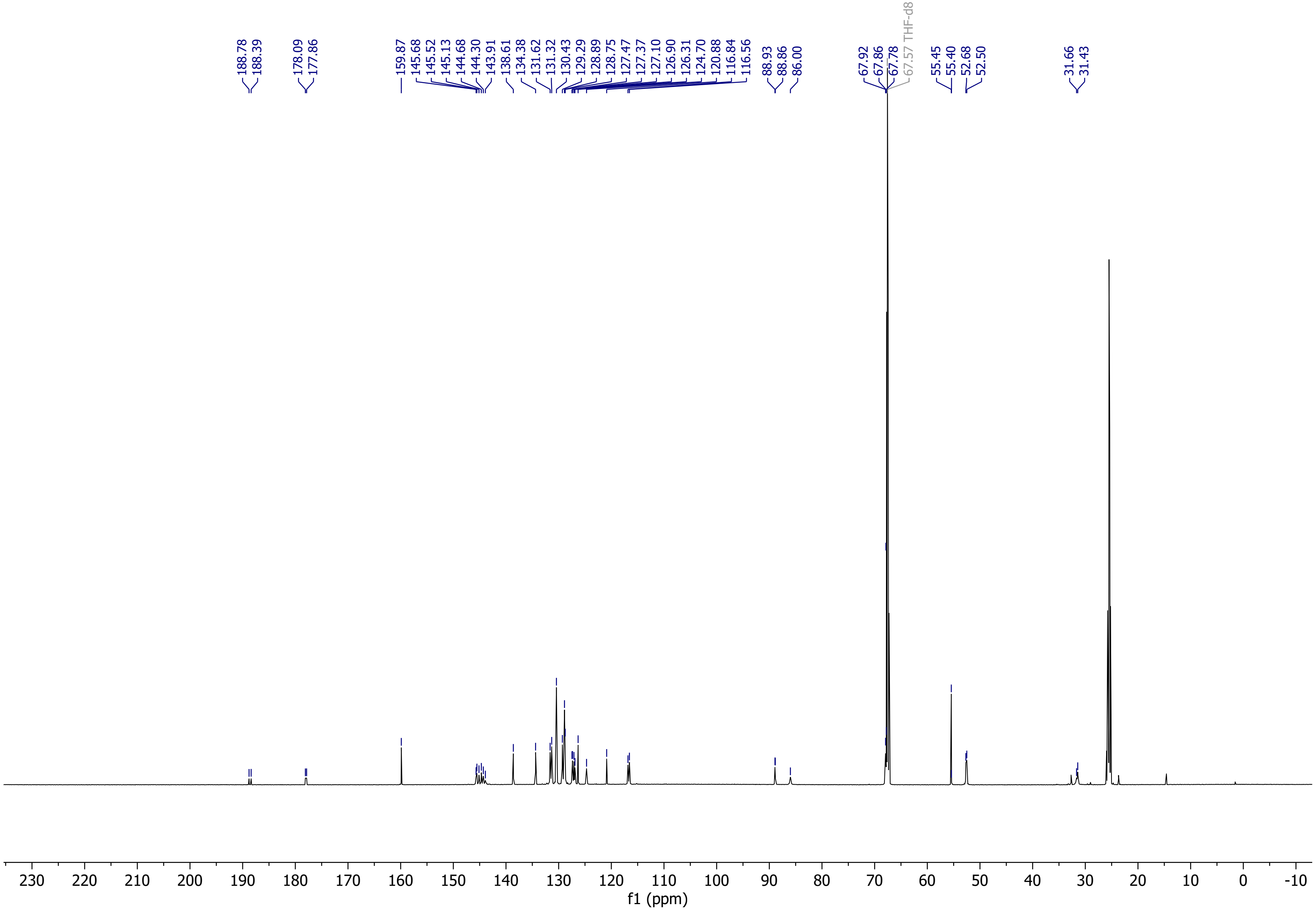

Figure S90: ${}^{13}C\{{}^{1}H\}$-NMR spectrum of **4** (126 MHz, THF-d_8).

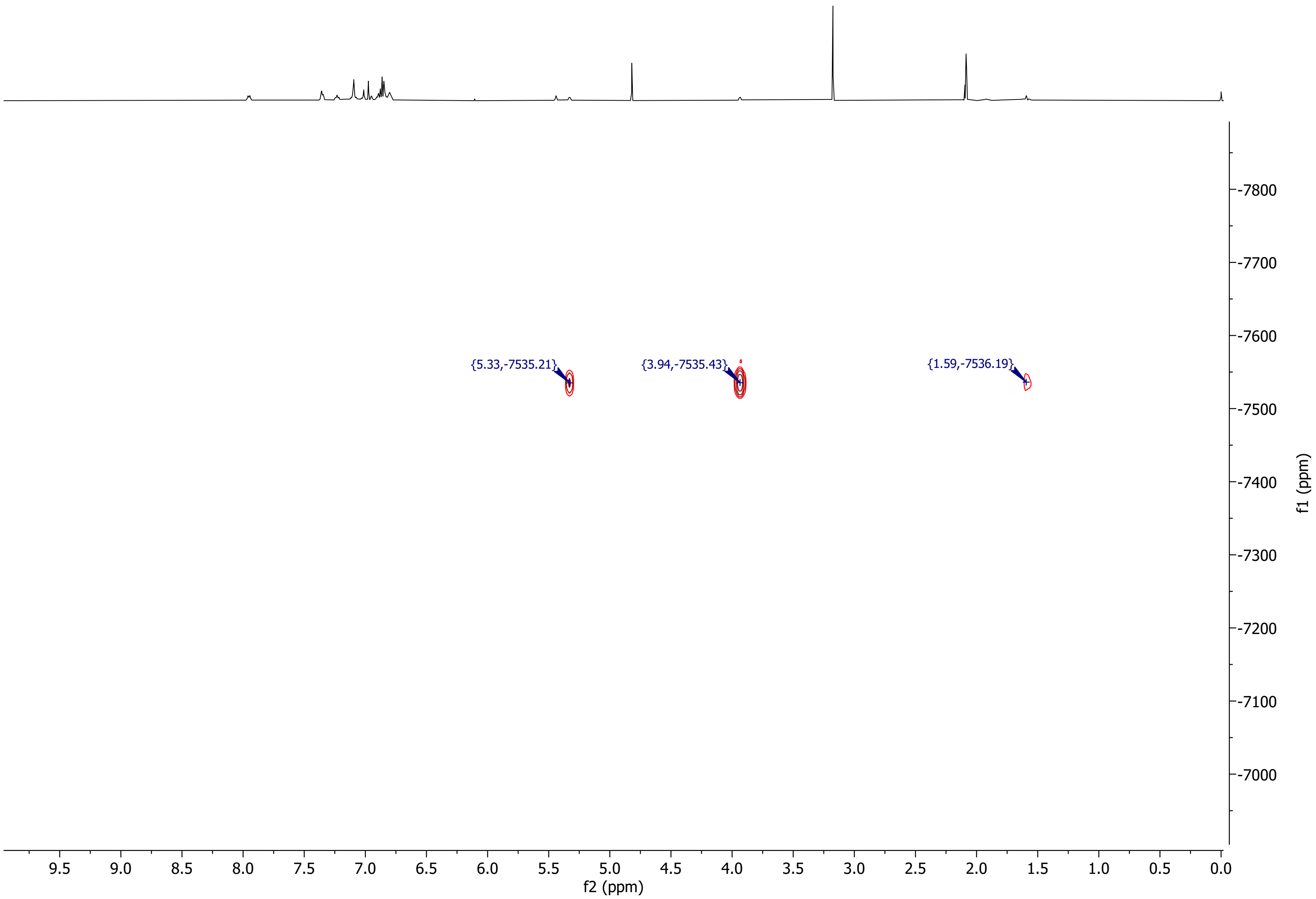

Figure S91: ^{1}H-^{103}Rh HMBC spectrum of **4** (16 MHz, THF-d_8).

DOSY experiments

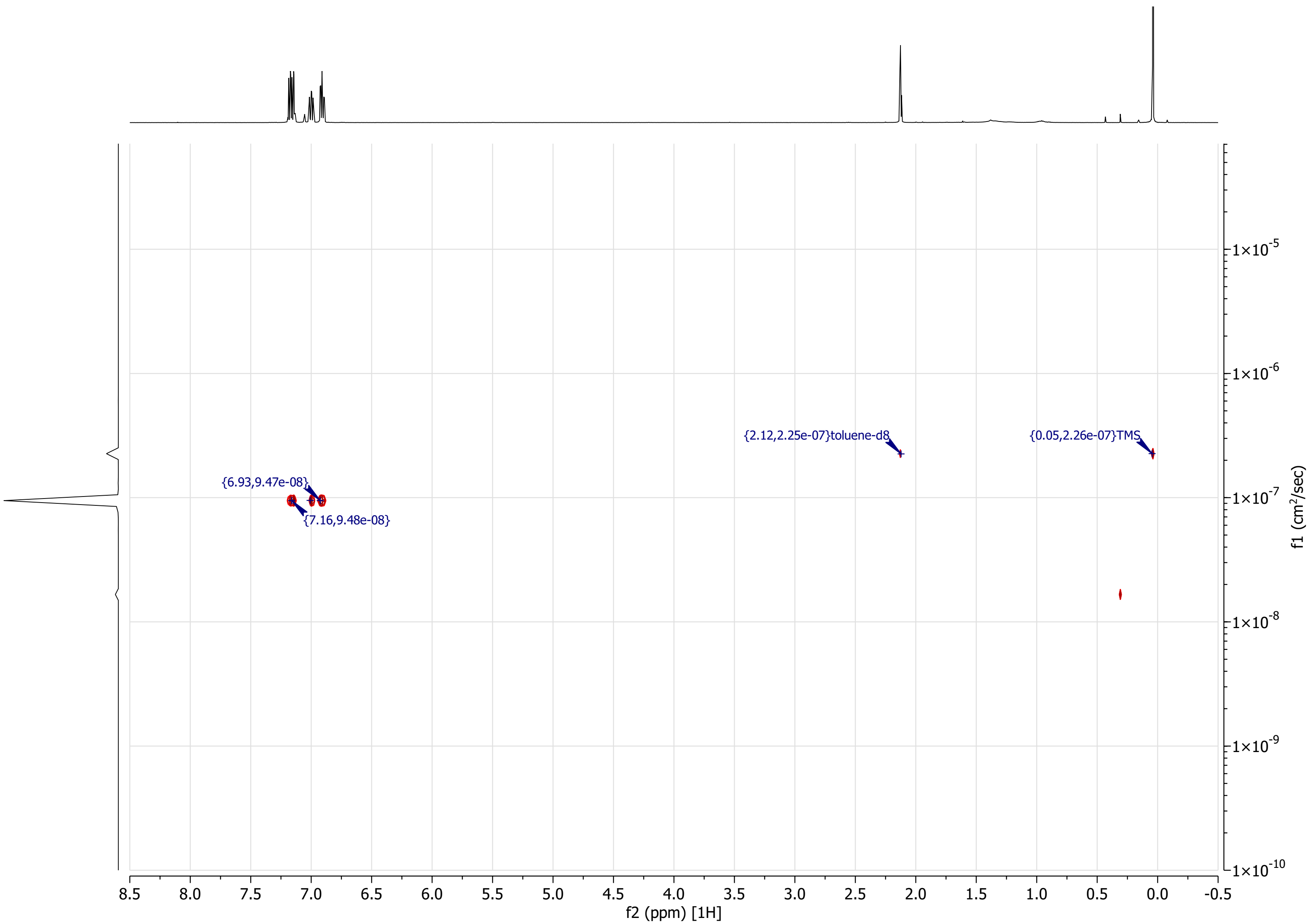

Figure S92: DOSY-^{1}H-NMR spectrum of $Au(PPh_3)Cl$ (500 MHz, toluene-d_8).

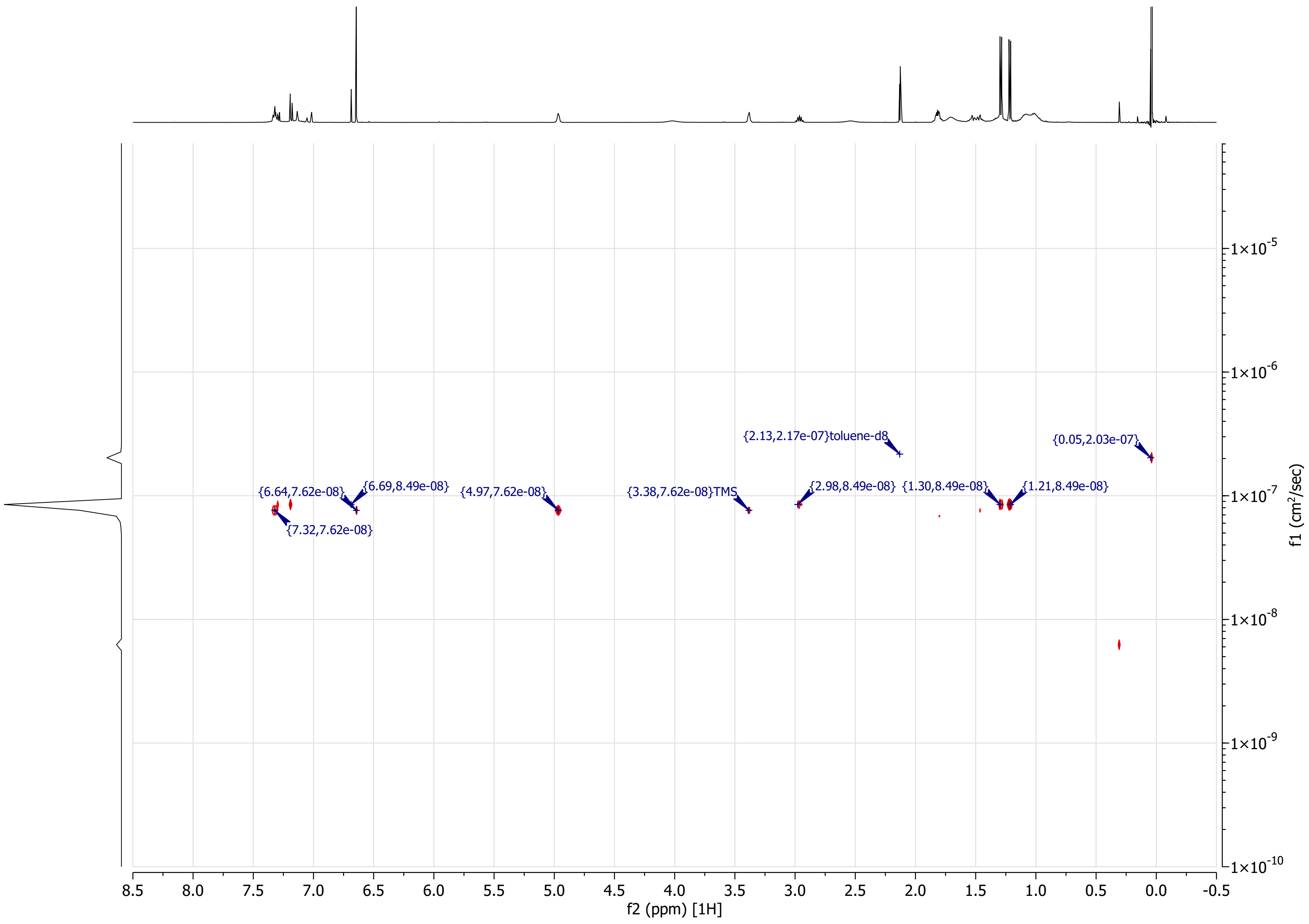

Figure S93: DOSY-^{1}H-NMR spectrum of complex Rh(IPr)(COD)Cl (**S2**) (500 MHz, toluene-d_8).

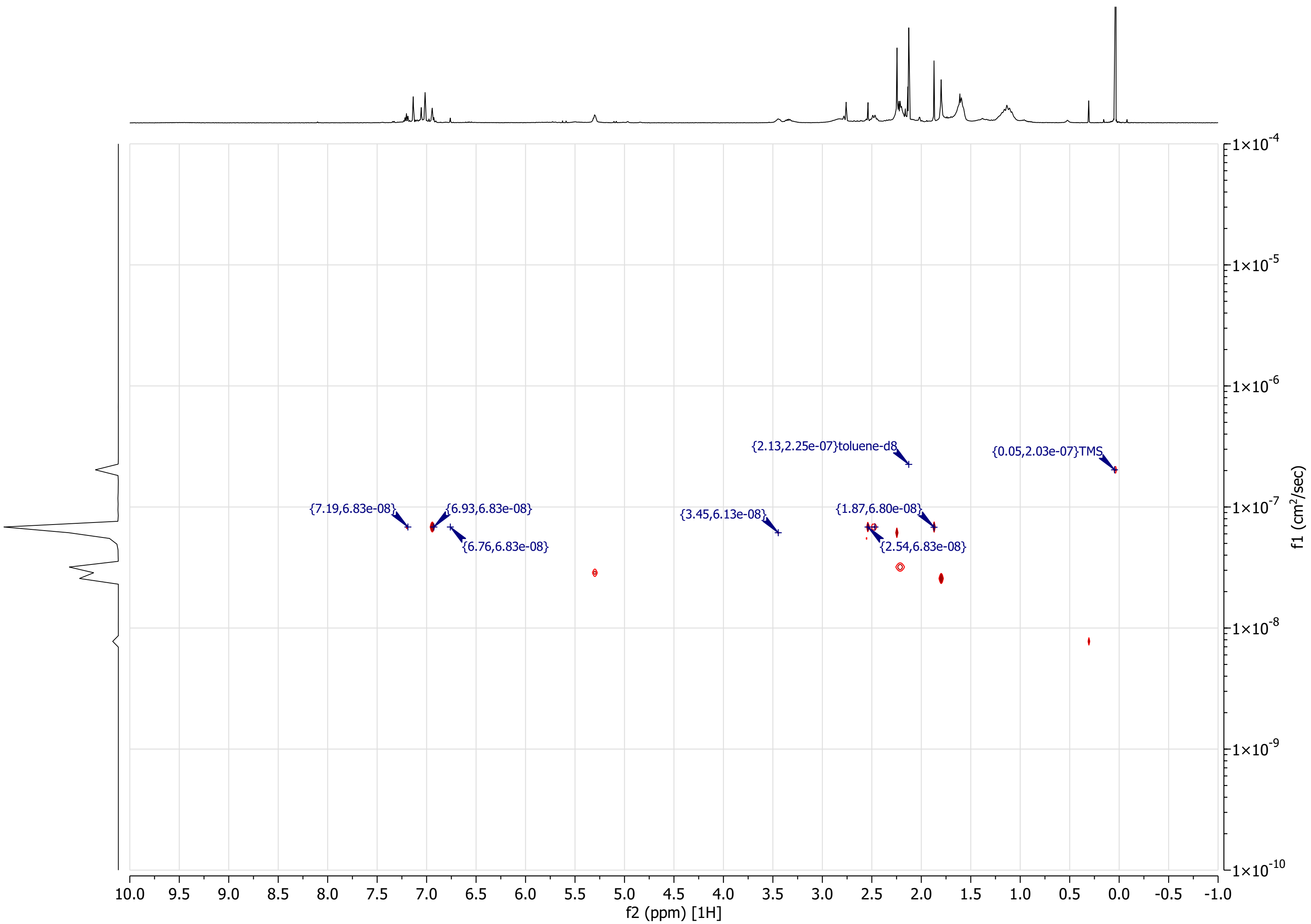

Figure S94: DOSY-^{1}H-NMR spectrum of Grubbs 2nd generation catalyst (500 MHz, toluene-d_8).

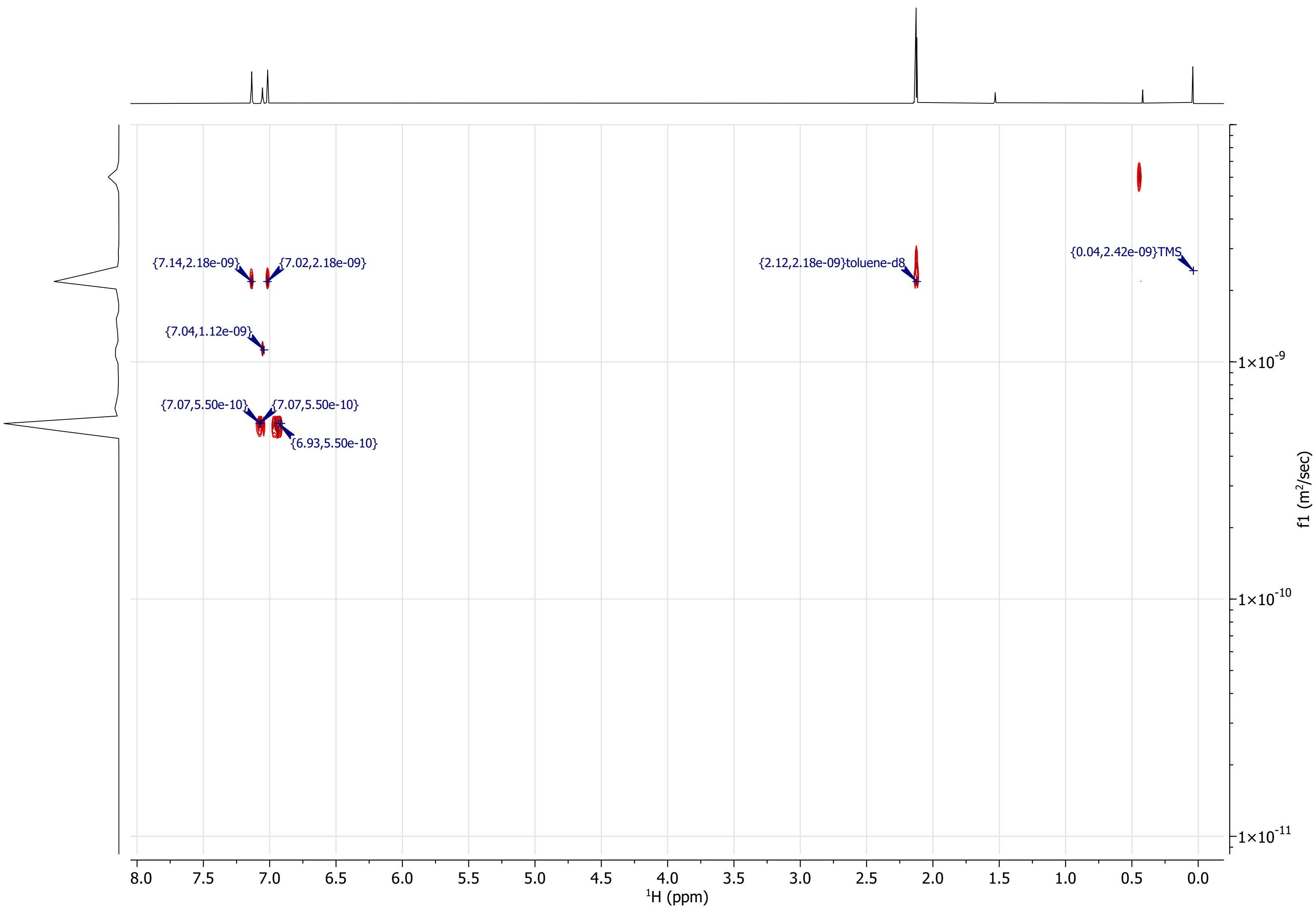

Figure S95: DOSY-^{1}H-NMR spectrum of complex $Ir(C_5Me_5)I_2$ (500 MHz, toluene-d_8).

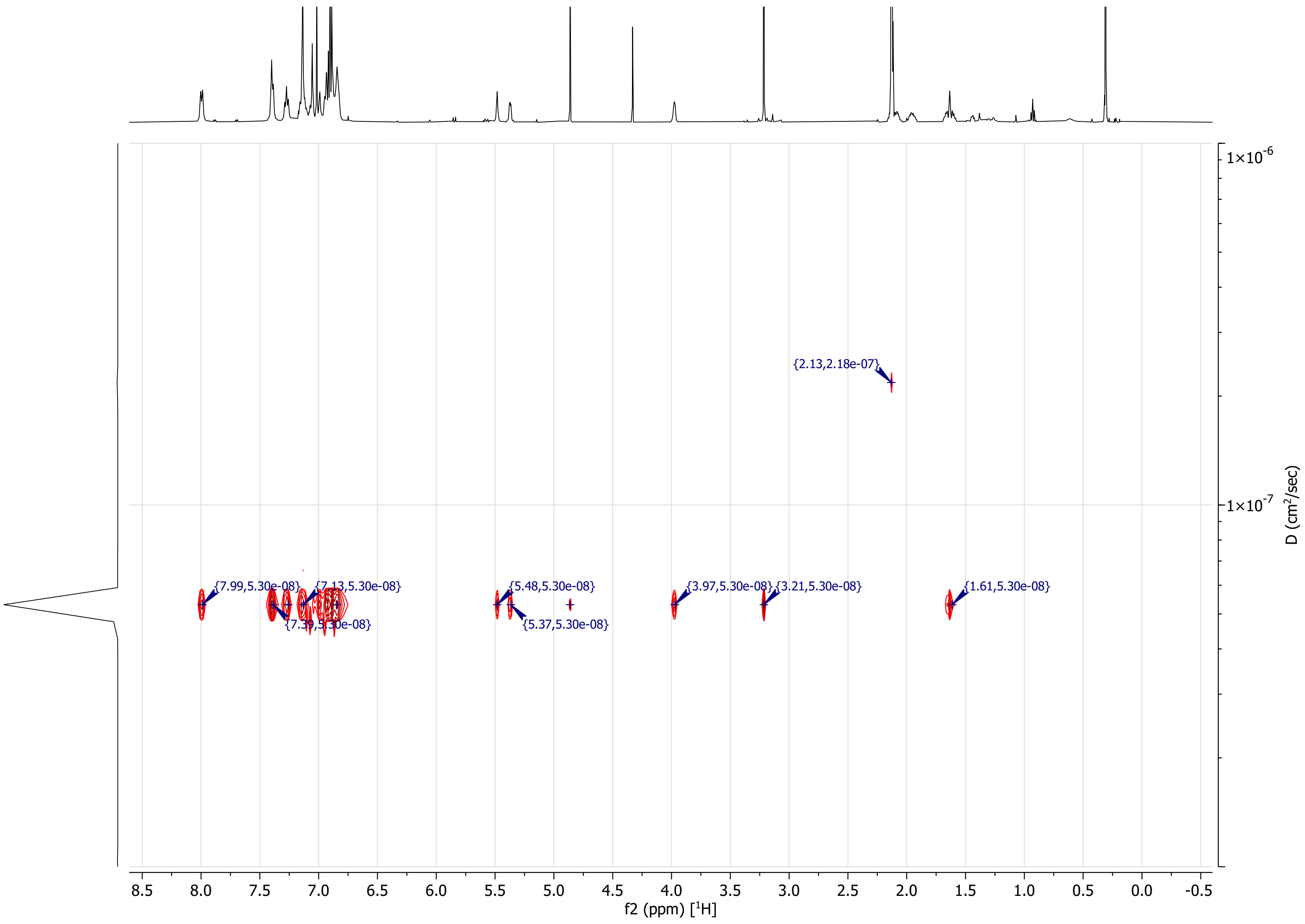

Figure S96: DOSY-^{1}H-NMR spectrum of complex **1** (500 MHz, toluene-d_8).

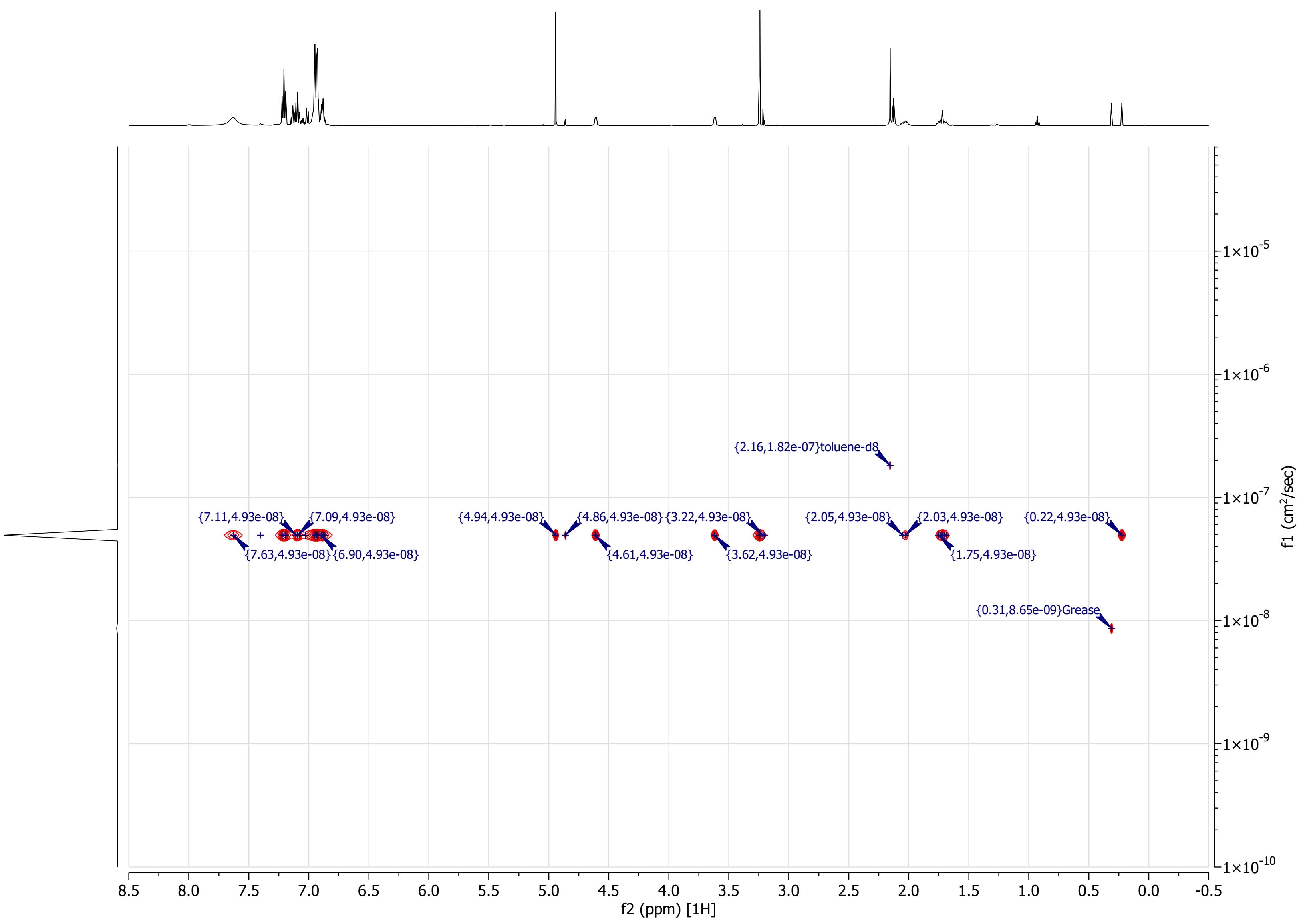

Figure S97: DOSY-^{1}H-NMR spectrum of complex **S1** (500 MHz, toluene-d_8).

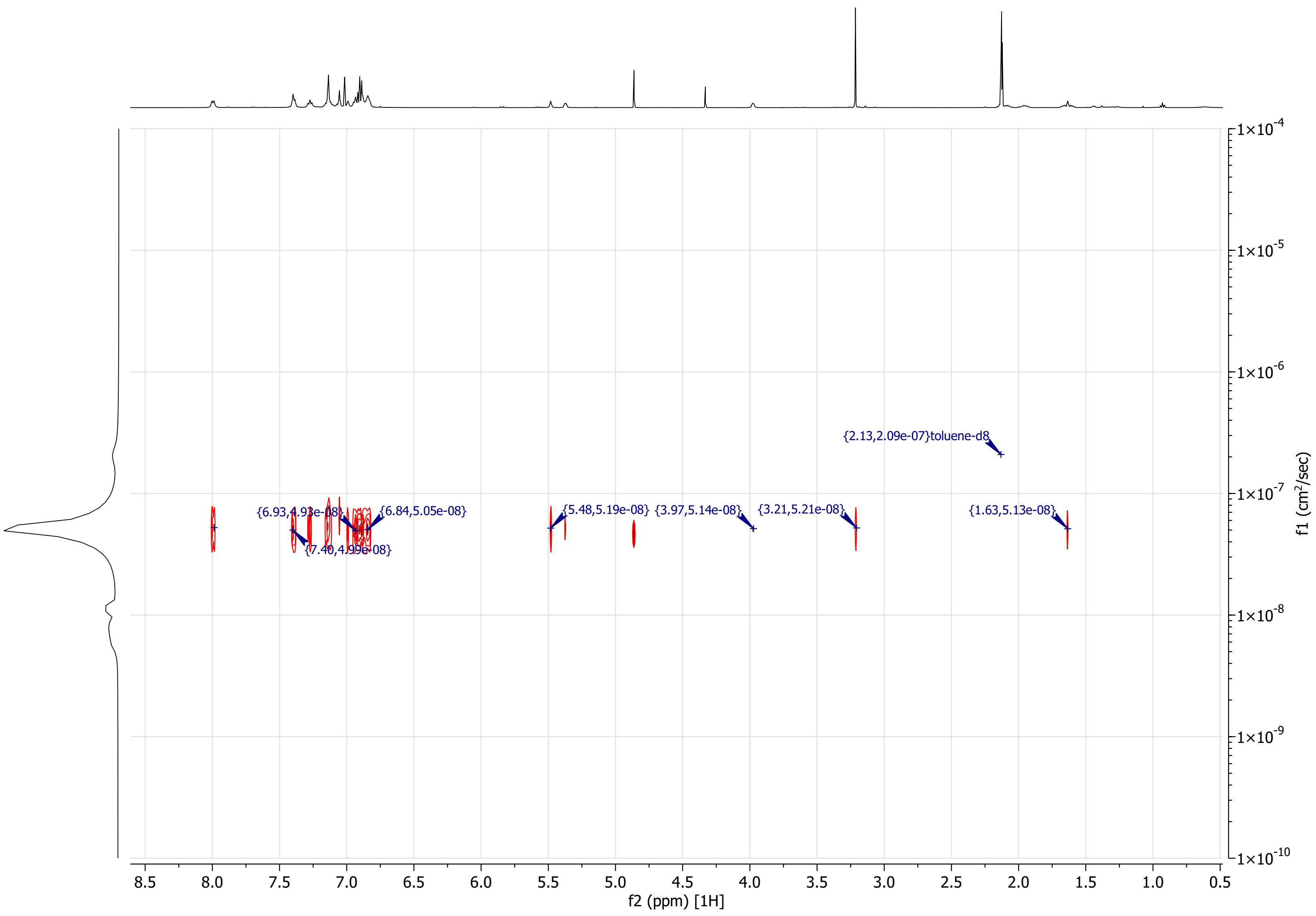

Figure S98: DOSY-^{1}H-NMR spectrum of complex **4** (500 MHz, toluene-d_8).

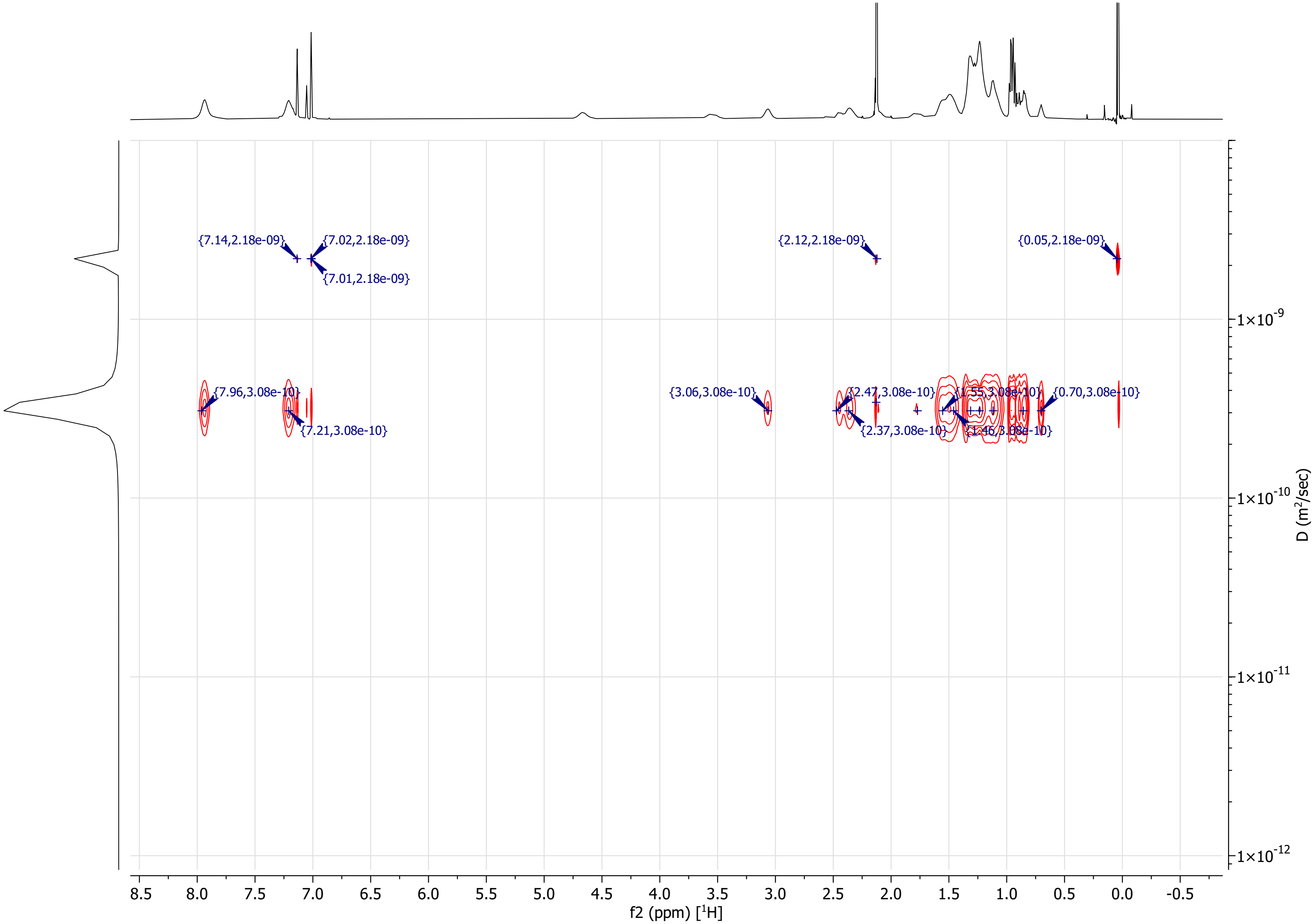

Figure S99: DOSY-^{1}H-NMR spectrum of complex $Rh_2(DOSP)_4$ (500 MHz, toluene-d_8).

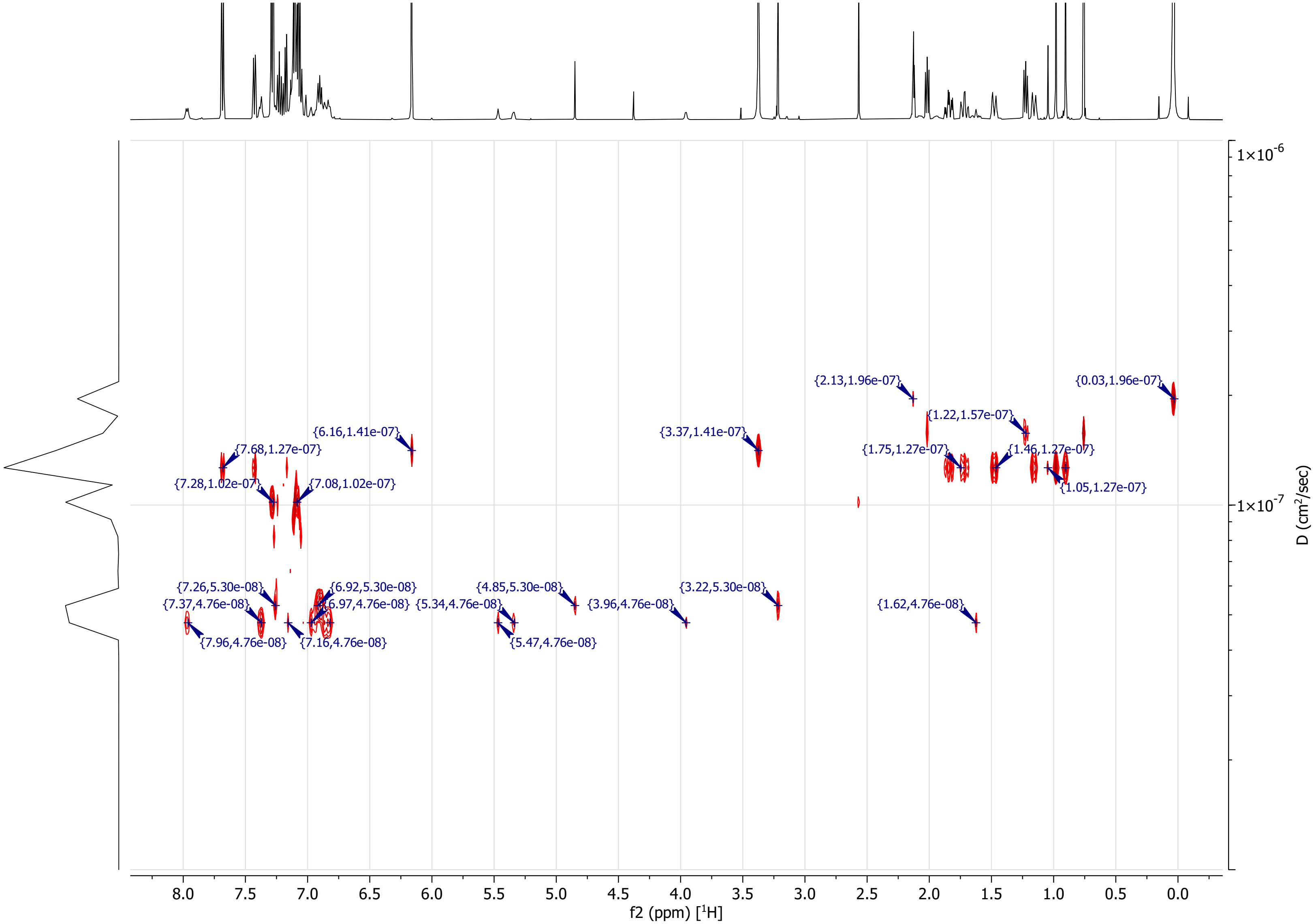

Figure S100: DOSY-^{1}H-NMR spectrum of the resting state **RS-1** in a catalytic reaction (500 MHz, toluene-d_8).

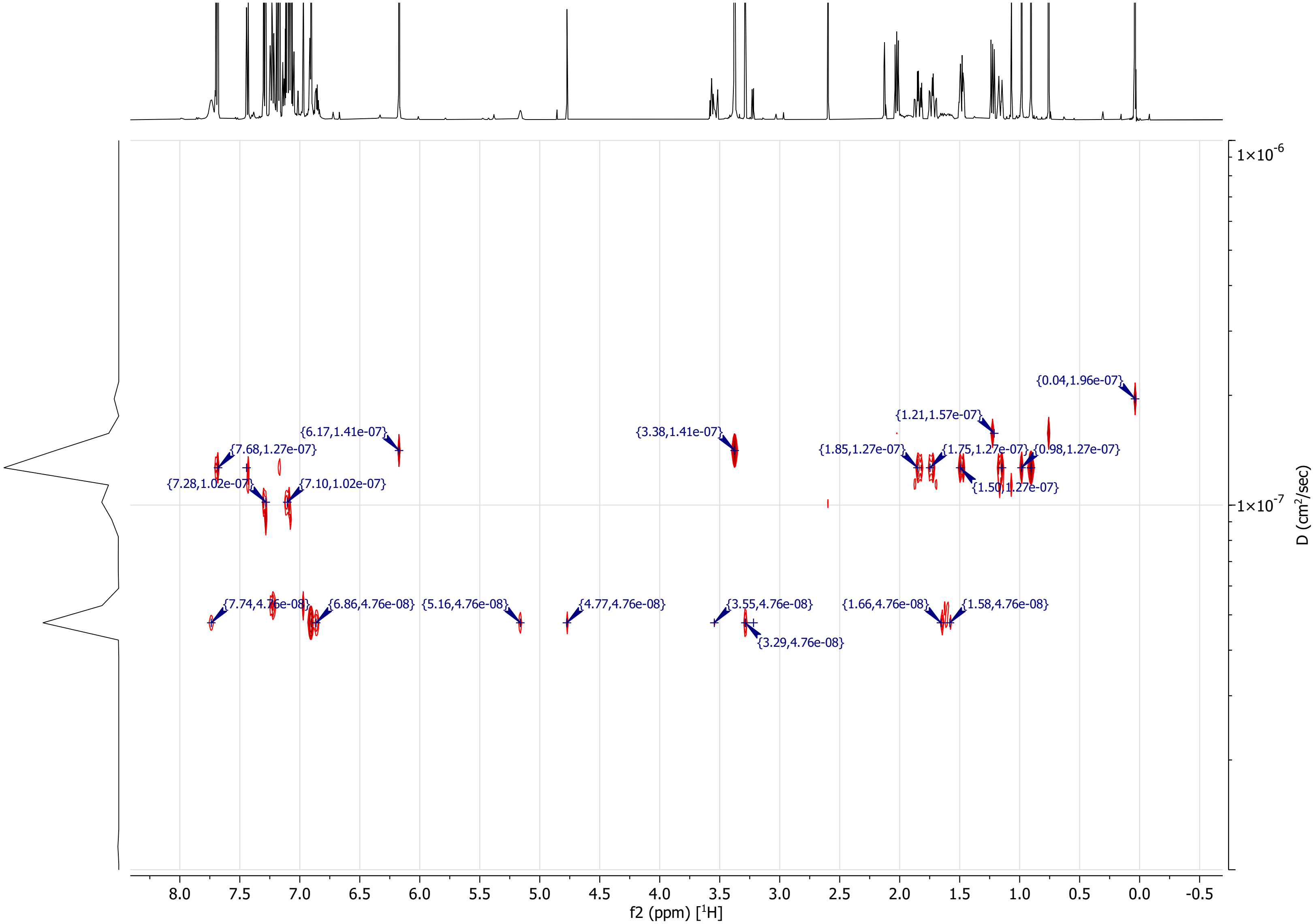

Figure S101: DOSY-^{1}H-NMR spectrum of the resting state **RS-3** in a catalytic reaction (500 MHz, toluene-d_8).
